# Structurally Defined Water‐Soluble Metallofullerene Derivatives towards Biomedical Applications

**DOI:** 10.1002/anie.202211704

**Published:** 2022-12-02

**Authors:** Yanbang Li, Rohin Biswas, William P. Kopcha, Thierry Dubroca, Laura Abella, Yue Sun, Ryan A. Crichton, Christopher Rathnam, Letao Yang, Yao‐Wen Yeh, Krishnendu Kundu, Antonio Rodríguez‐Fortea, Josep M. Poblet, Ki‐Bum Lee, Stephen Hill, Jianyuan Zhang

**Affiliations:** ^1^ Department of Chemistry and Chemical Biology, Rutgers The State University of New Jersey 123 Bevier Rd Piscataway NJ 08854 USA; ^2^ National High Magnetic Field Laboratory (NHMFL) Florida State University 1800 E. Paul Dirac Dr. Tallahassee FL 32310 USA; ^3^ Departament de Química Física i Inorgànica Universitat Rovira i Virgili Marcel⋅lí Domingo 1 43007 Tarragona Spain; ^4^ Department of Physics and Astronomy, Rutgers The State University of New Jersey 136 Frelinghuysen Rd Piscataway NJ 08854 USA; ^5^ Department of Physics Florida State University Tallahassee FL 32306 USA

**Keywords:** Contrast Agents, Endohedral Fullerenes, Rare Earth Compounds, Supramolecular Chemistry, Synthesis Design

## Abstract

Endohedral metallofullerenes (EMFs) are excellent carriers of rare‐earth element (REE) ions in biomedical applications because they preclude the release of toxic metal ions. However, existing approaches to synthesize water‐soluble EMF derivatives yield mixtures that inhibit precise drug design. Here we report the synthesis of metallobuckytrio (MBT), a three‐buckyball system, as a modular platform to develop structurally defined water‐soluble EMF derivatives with ligands by choice. Demonstrated with PEG ligands, the resulting water‐soluble MBTs show superb biocompatibility. The Gd MBTs exhibit superior *T*_1_ relaxivity than typical Gd complexes, potentially superseding current clinical MRI contrast agents in both safety and efficiency. The Lu MBTs generated reactive oxygen species upon light irradiation, showing promise as photosensitizers. With their modular nature to incorporate other ligands, we anticipate the MBT platform to open new paths towards bio‐specific REE drugs.

## Graphical abstract

A modular metallobuckytrio (MBT) platform provides a family of structurally defined, water‐soluble metallofullerene derivatives that can safely carry rare‐earth elements in tailorable molecules, showing promise in biomedicines including MRI contrast agents and photosensitizers.

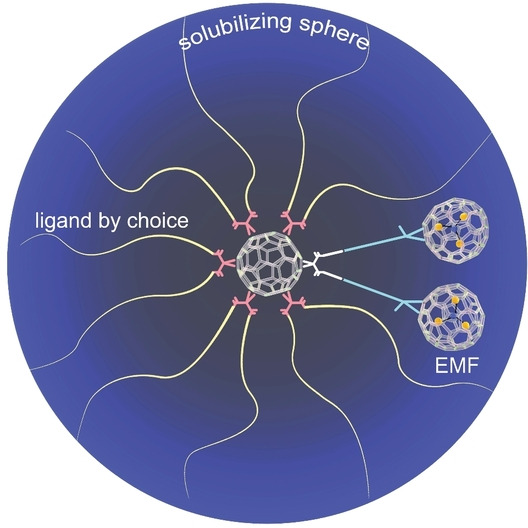

## Introduction

Rare‐earth elements (REEs) are valuable components for medicines due to their distinctive properties.[^1^, ^2^, ^3^, ^4^] For example, the high Z number of lanthanides makes them great X‐ray contrast agents in computed tomography (CT); their heavy atom effect to facilitate intersystem crossing (ISC) is important for photosensitizers in photodynamic therapy (PDT); the characteristic radioactivity of many REE isotopes (^90^Y, ^177^Lu, ^166^Ho, etc.) is very useful in both diagnostic (e.g. positron emission tomography) and therapeutic (e.g. brachytherapy) applications; the ideal paramagnetism of the Gd^3+^ ion crowns it the most desirable element for in MRI contrast agents. To realize the potential of REEs in medicine, the first and foremost challenge is to safely confine the toxic metal ions to ensure they do not leak into the physiological environment. The dilemma of gadolinium‐based contrast agents (GBCAs) for MRI^[5]^ is a perfect case in point. Current clinical contrast‐enhanced MRI exams are using GBCAs based on chelating complexes,^[6]^ which have high coordination constants, but the Gd^3+^ ions can still escape from the chelates under complex conditions. The metal leak caused significant safety concerns, including kidney dysfunction (nephrogenic systemic fibrosis)[^7^, ^8^] and long‐term Gd^3+^ deposition in the brain[^9^, ^10^] which led to the suspension of a few approved GBCAs in Europe.^[11]^ In the past decades, numerous efforts have been devoted to preventing the Gd release in GBCAs.[^5^, ^6^]

**Figure 1:**
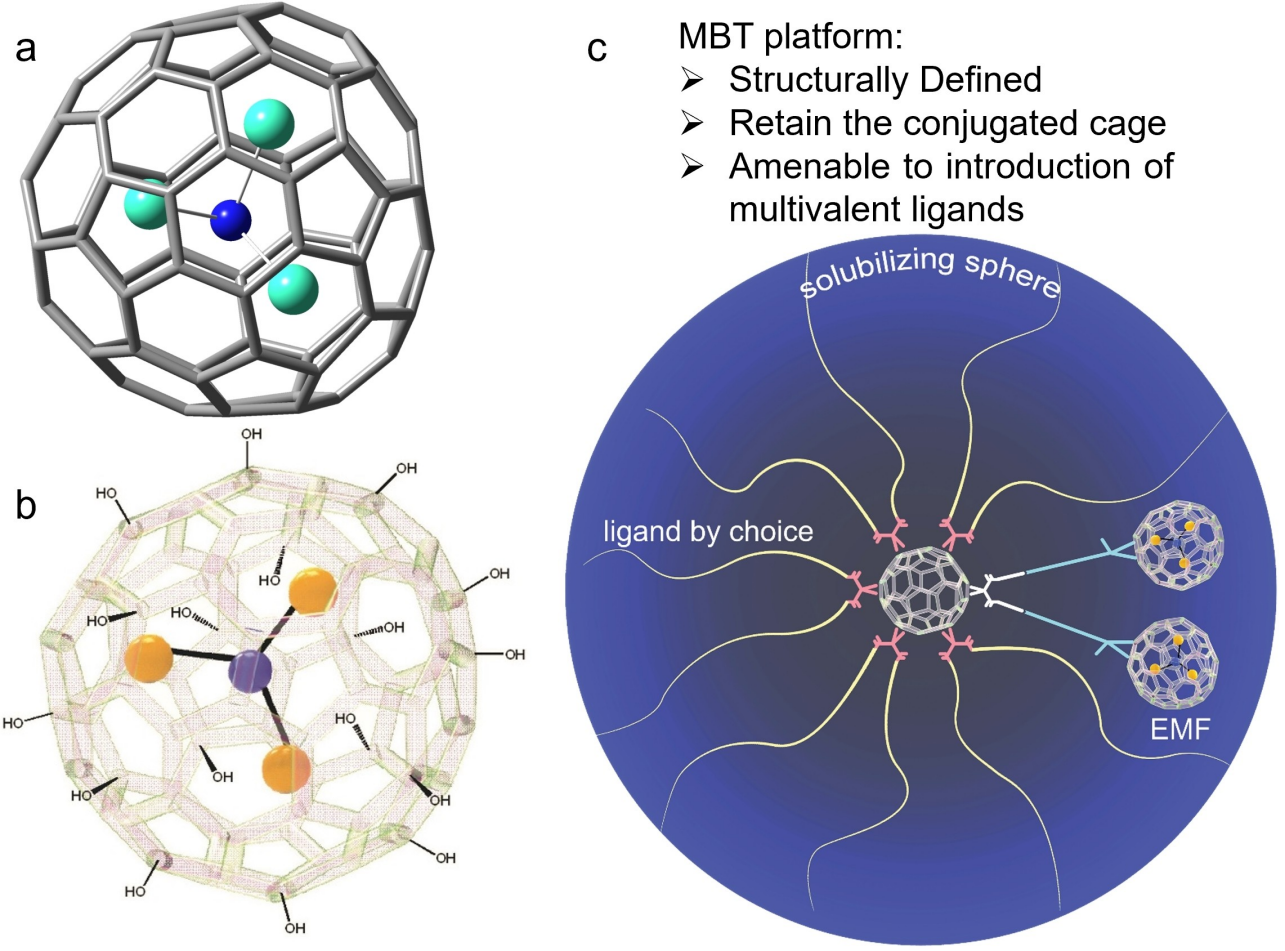
Overview of the MBT design. a) The structure of M_3_N@C_80_ used in this work, as an example of EMF. b) Metallofullerenol, a typical example of water‐soluble EMF derivative with hydrophilic groups of uncertain numbers and positions. c) Schematic representation of the MBT design, and its key advantages compared to other water‐soluble EMFs.

The unique structure of endohedral metallofullerenes (EMFs),^[12]^ i.e., a metal ion or cluster in a carbon cage (Figure 1a), provides the inherent protection of Gd or other REEs in both ways: the preservation of the magnetic properties of ions, and the complete prevention of metal leakage. While the former opens paths to applications such as spin qubits,[^13^, ^14^] single molecular magnets,[^15^, ^16^, ^17^, ^18^, ^19^, ^20^] and dynamic nuclear polarization,^[21]^ the latter is a critically desired feature for REE biomedicines. Indeed, many water‐soluble Gd EMF derivatives have shown great promise with presumed safety and much higher relaxivity than commercial GBCAs.[^22^, ^23^, ^24^, ^25^, ^26^, ^27^, ^28^, ^29^, ^30^, ^31^, ^32^, ^33^] One fundamental issue, however, arises. To solubilize hydrophobic EMFs in water, extensive surface modification with multiple (≈10–40) hydrophilic groups is required, but existing approaches[^32^, ^33^] result in mixtures with heterogeneous number and random regiochemistry of the hydrophilic groups (Figure 1b), posing serious challenges in characterization and reproducibility: the mixtures lack a definitive structure to be well‐characterized by NMR or mass‐spectrometry (MS), so X‐ray photoelectron spectroscopy (XPS) and IR assumed the burden as a compromise. Additionally, their hydrogen‐bond‐driven aggregation in solutions[^24^, ^27^, ^28^, ^29^] would conceal the functional groups, which hinders the further introduction of biological ligands (marked by low yields^[30]^). Meanwhile, the broken π‐conjugation causes the loss of electronic and photophysical properties of the EMF cage, voiding certain potential applications such as photosensitizers. Collectively, these limitations seriously inhibit robust quality control, drug design, and future administrative approval of EMF derivatives for biomedicines. While precise non‐ionic water‐soluble derivatives of fullerene C_60_ were achieved by multiadditions,[^34^, ^35^] efficient and precise EMF multiadditions with a large number of functional groups have yet to be developed, although there are promising recent work that added 2–4 functional groups regioselectively.[^36^, ^37^, ^38^, ^39^]

In a paradigm shift, here we report a three‐buckyball “metallobuckytrio” (MBT) platform to systematically develop structurally well‐defined water‐soluble EMF derivatives (Figure 1c). The MBT design decouples the functionalization of the EMF and the introducing of solubilizing groups into separate operations. Instead of making multiadducts, monoadducts of EMF are connected to the core of a C_60_ hexakisadduct^[40]^ that can take 10 other ligands for both aqueous solubility and desired functions. Compared to previously reported water‐soluble EMF derivatives, the MBT design has several advantages: 1) it is molecularly precise, with no batch‐to‐batch variations; 2) it retains the conjugated structure of the EMF cage, bringing its electronic and photophysical properties to the aqueous phase; 3) it is modular to take 10 functional ligands, opening vast design space. We demonstrate the synthetic platform with oligo‐ and polyethylene glycol (PEG) ligands. In vitro studies on the resulting water‐soluble MBTs also affirm their excellent biocompatibility in three representative cell lines. The Gd MBTs show significantly higher *T*_1_ relaxivity values than commercial MRI contrast agents. Moreover, with retained conjugated EMF cage MBTs generated Type I and Type II reactive oxygen species (ROS) under light irradiation. With the proof‐of‐concept work, we envision the MBTs becoming a general interface for organic and biological operations on sealed REEs in water with ligands of choice.

## Results and Discussion

### Synthesis and Characterization of the MBT Platform

**Scheme 1:**
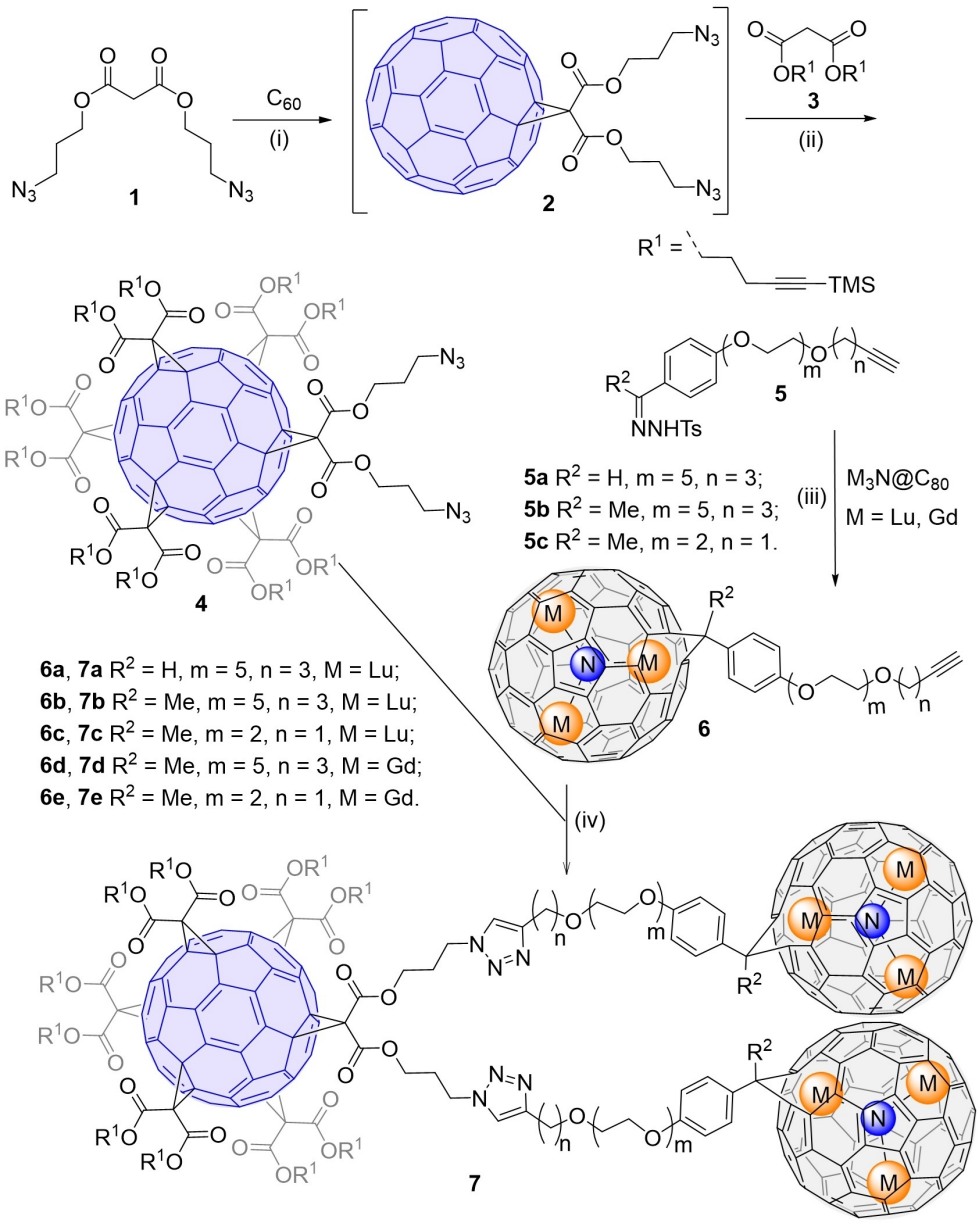
Synthesis of the MBT Platform **7**. Reagents and conditions: (i) C_60_, CBr_4_, DBU, toluene, r. t.; (ii) CBr_4_, DBU, toluene, r. t.; (iii) NaOMe, Pyridine, *o*‐DCB, r. t. to 70 °C (for **6** **a**–**c**) or 55 °C (for **6** **d**–**e**); (iv) CuBr⋅SMe_2_, sodium ascorbate, Cu^0^, *o*‐DCB, r. t. (for **7** **a**) or 40 °C (for **7** **b**–**e**).

As shown in Scheme 1, the basic MBT structure is formed by connecting two key building blocks: a C_60_ hexakisadduct and two molecules of EMF monoadducts. Hexakis‐addition of C_60_ via the Bingel‐Hirsch reaction provides excellent isomerically pure scaffolds for molecular materials construction.[^35^, ^41^, ^42^, ^43^, ^44^, ^45^, ^46^, ^47^, ^48^, ^49^] In our work, C_60_ was reacted with 1 eq of malonate **1** to afford **2**, which was purified and then directly used in the reaction with a large excess of malonate **3** to afford the C_60_ hexakisadduct **4** (characterizations in Figures S1–S4). Meanwhile, EMFs M_3_N@C_80_ (M=Lu, Gd) reacted with **5** (synthesis in SI, characterization of precursors in Figures S5–S18) in a well‐established diazo addition to yield monoadducts **6** **a**–**e** with [6,6]‐open structures,[^50^, ^51^] which were purified by flash chromatography, and characterized by high‐performance liquid chromatography (HPLC), matrix‐assisted laser desorption ionization time‐of‐flight (MALDI‐TOF) MS, and UV/Vis spectroscopy, as well as ^1^H, ^13^C, distortionless enhancement by polarization transfer (DEPT) −135 ^13^C, and heteronuclear multiple quantum coherence (HMQC) NMR for diamagnetic Lu EMF derivatives **6** **a**–**c** (Figures S19–S47). For Gd_3_N@C_80_ derivatives **6** **d**–**e**, in which the paramagnetic Gd^3+^ prevented meaningful NMR study, MALDI‐TOF confirmed the molecular mass, and the HPLC and UV/Vis results were carefully compared to the Lu counterparts, as well as literature reporting other [6,6]‐open M_3_N@C_80_ derivatives to check the structure.[^50^, ^51^, ^52^] Finally, **4** and **6** were mixed in 1 : 3 ratio under customized CuAAC conditions to form MBTs platforms **7**. Notably, in the whole synthetic route, we were able to use exclusively flash chromatography purifications, which is a scalable and universal approach that allows researchers to reproduce our synthesis without specialized HPLC columns.

**Figure 2:**
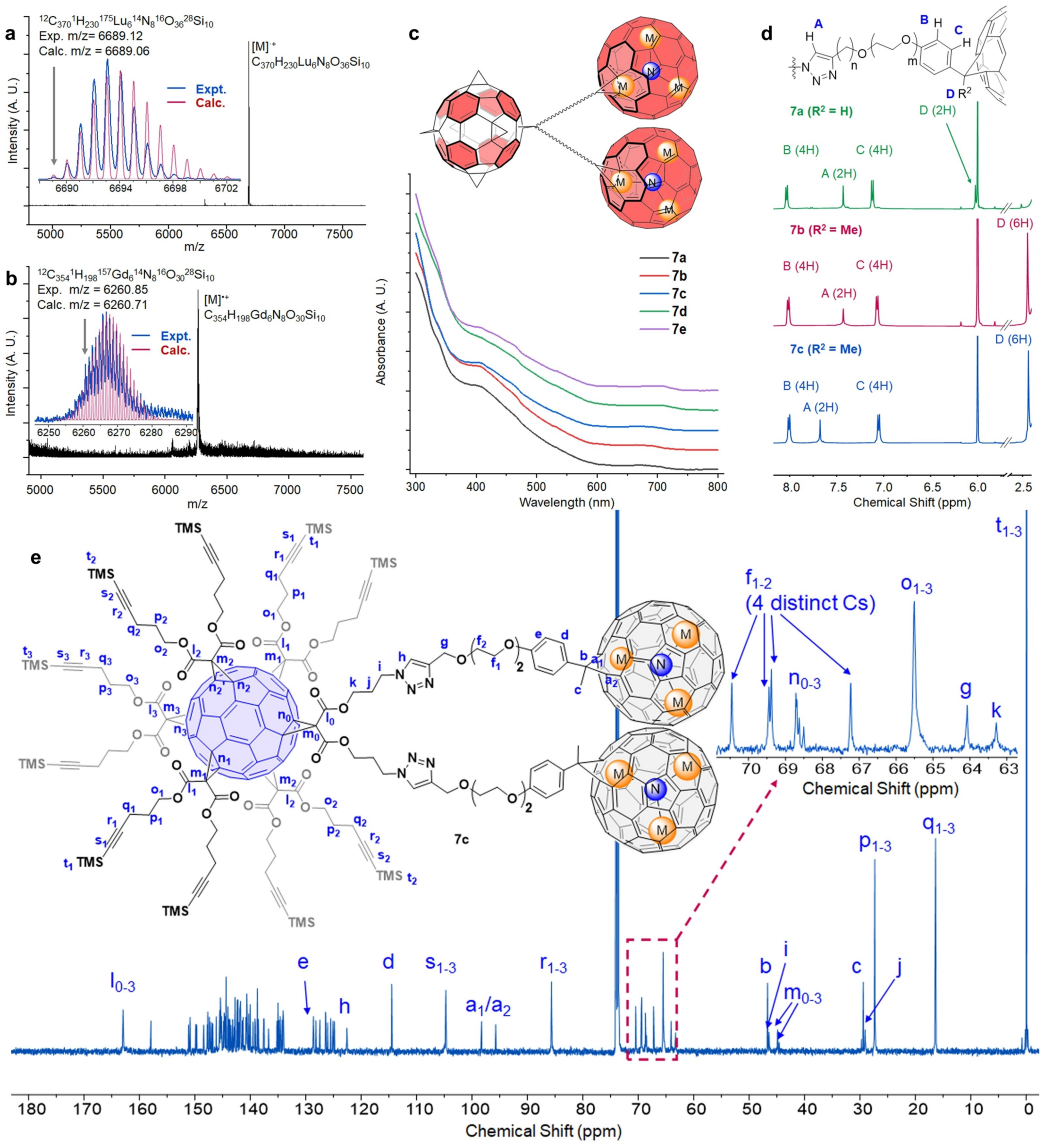
Characterization of MBTs **7**. a, b) MALDI‐TOF MS of Lu MBT **7** **b** (a) and Gd MBT **7** **e** (b). The molecular ion peaks corresponding to the most abundant isotope and the overall isotopic distributions matched the calculated results. c) UV/Vis spectra of **7** **a**–**e**. The MBTs have the same extinction peak positions, suggesting that they share the same conjugated aromatic structure, as highlighted in red. d) Selected region in the ^1^H NMR of **7** **a**–**c**, which shows the ratio of the four characteristic protons A/B/C/D as 2H/4H/4H/2H for **7** **a**, and 2H/4H/4H/6H for **7** **b**, **7** **c**. e) ^13^C NMR of **7** **c**, with peak assignments.

Precedents of linking EMF and C_60_ are rare,[^53^, ^54^] and due to the challenge in the synthesis and purification, some key characterizations such as ^13^C NMR were not achieved for the EMF‐C_60_ conjugates in the earlier work. The MBT platforms were extensively characterized by MALDI‐TOF MS, UV/Vis spectra, and various NMR approaches (for diamagnetic **7** **a**–**c**), including ^1^H, ^13^C, DEPT‐135 ^13^C, and HMQC spectra (Figure S48–73). Despite the large size and proneness to fragmentation, molecular ion peaks for **7** **a**–**e** were all observed in MALDI‐TOF (Figure 2a–b, Figures S48, S62, S69), which provides solid initial evidence of the structures. Meanwhile, the UV/Vis spectra of **7** **a**–**e** (S49, S56, S63, S70, S73) overlapped in extinction peak positions (Figure 2c), which supports that, as expected, all MBTs **7** **a**–**e** share the same conjugated aromatic structure (highlighted in red in Figure 2c). This observation allowed us to focus on the structural elucidation of the 3 diamagnetic Lu MBTs as structural probes with NMR. Peaks were assigned in the ^1^H NMR of **7** **a**–**c** (Figures S50, S57, S64), and the integrations of four characteristic protons (A–D in Figure 2d) signals, namely, proton A from the triazole ring, protons B and C from the phenyl ring, and proton(s) D from the hydrogen (**7** **a**) or methyl group (**7** **b**, **c**) attached to the geminal bridgehead carbon adjacent to the C_80_ cage, were closely examined. The results showed the A/B/C/D integrations of 2/4/4/2 for **7** **a**, and 2/4/4/6 for **7** **b** and **7** **c**, while the TMS peaks at 0.17–0.20 ppm showed combined integrations of ≈90 protons in all, which are consistent with the drawn MBT structures.

Further detailed structural evidence is provided by the ^13^C NMR spectra (Figures 2e, S51, S58, S65). Using **7** **c** as an example, all the carbon nuclei on the functional groups, and the *sp* ^*3*^ carbon nuclei on the fullerene and EMF cages, were assigned as a‐t in Figure 2e based on the DEPT‐^13^C, HMQC spectra, and the comparison with precursor molecules **4**, **6** **c**, and MBTs **7** **a**–**b**. Due to the different regiochemical locations relative to the two EMF functional groups, the *sp* ^*3*^ carbon atoms on the C_60_ (n_0_, n_1_, n_2_, n_2′_, n_3_), as well as the carbon nuclei at the corresponding locations on the ligands, are non‐equivalent to each other. However, with finite instrumental capacity, only some were resolved (l_0‐3_, m_0‐3_, n_0‐3_, s_1‐3_), while others (o_1‐3_, p_1‐3_, q_1‐3_, r_1‐3_, t_1‐3_) showed as one peak. In the same vein, theoretically **7** **c** should have 89 different *sp* ^*2*^ carbon nuclei that do not have hydrogen attachments (78 from the EMF,^[50]^ 2 from the phenyl rings, 1 from the triazole ring, and 8 from the C_60_ core), while 85 distinct peaks were identified in the region between 120 and 160 ppm, excluding the assigned e and h that have proton attachments. Fully resolving the theoretically 78 peaks on an asymmetric [6,6]‐open C_80_ cage is ultimately challenging;[^50^, ^55^, ^56^, ^57^, ^58^] however, the number of ^13^C NMR signals (85 out of 89) still clearly shows the symmetry of the molecule and corroborates the structural elucidation.

### Synthesis and Supramolecular Interactions of the Water‐soluble MBTs

**Scheme 2:**
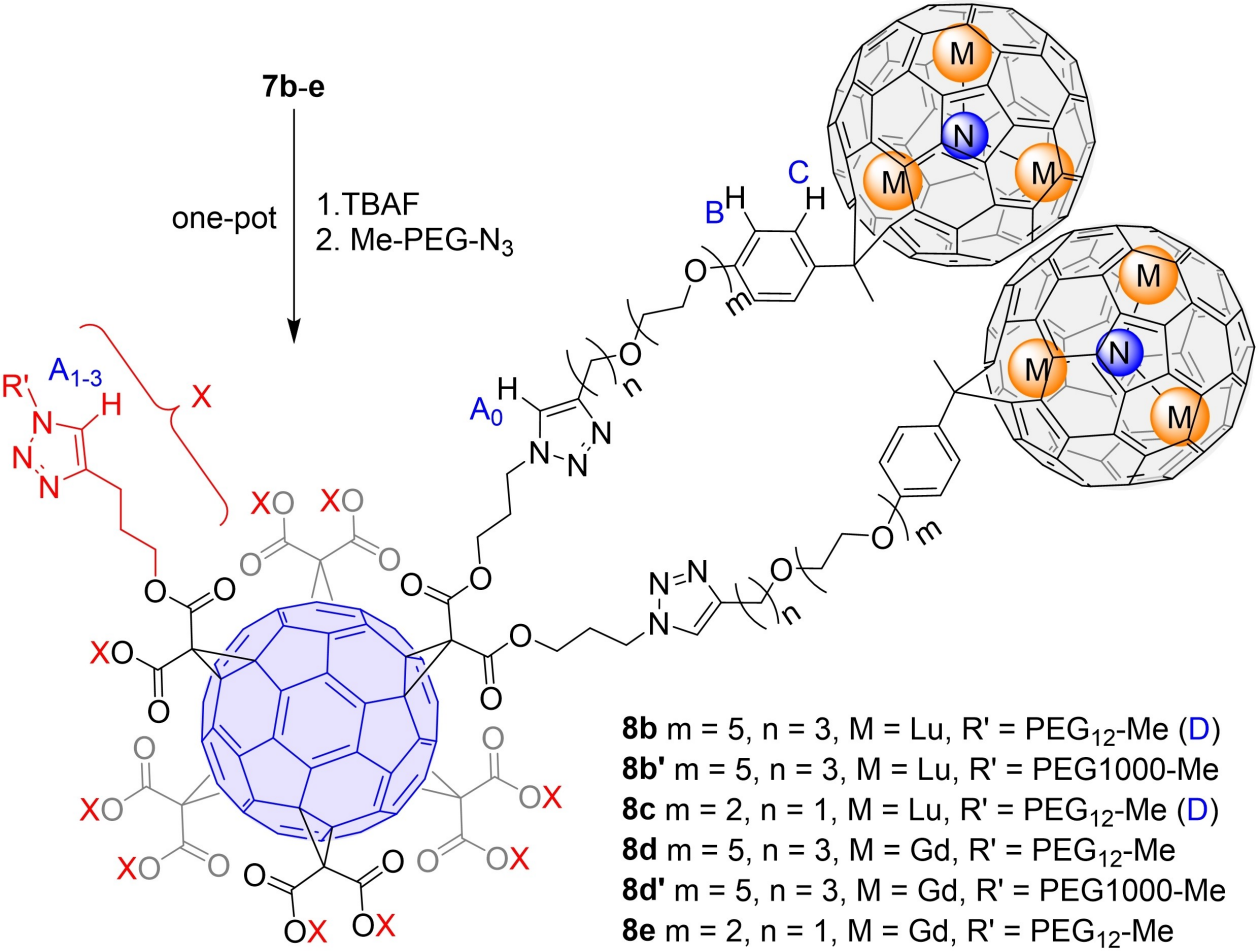
Functionalization of MBTs with PEG ligands via CuAAC click chemistry. The products **8** are all water‐soluble.

MBT **7** is a versatile platform ready for ligand installation. In this work, we use azido dodecaethylene glycol (PEG_12_, commercial) and polyethylene glycol (PEG1000) ligands (2 step synthesis in SI, characterization see Figures S74–77) to dissolve them in water. As shown in Scheme 2, N_3_‐PEG‐OMe ligands were clicked onto MBTs **7** **b**–**e** to yield the water‐soluble MBTs **8**. All the products were easily purified with straightforward dialysis thanks to the water‐solubility and large molecular weight (>10k) to afford pure products (characterization Figures S78–S97), among which **8** **b**, **8** **c**, **8** **d**, and **8** **e** are the first examples of molecularly precise water‐soluble EMF derivatives, while **8** **b′**, **8** **d′** are imprecise only because of the slight polydispersity of PEG1000. We do note that the concept of connecting monoadducts of EMF to a larger biocompatible system and therefore achieve water solubility with defined functionalization has been achieved in a “bioshuttle” system, although the metallofullerene samples used therein were mixtures.[^59^, ^60^] The UV/Vis spectra of the compounds **8** were compared with their precursors and with each other to confirm the intactness of the conjugated backbones (Figures S95–S97). To verify the successful attachment of 10 ligands, peaks in the ^1^H NMR of the two precise Lu MBTs **8** **b** and **8** **c** were carefully assigned and four characteristic proton signals (labeled in Scheme 2) A_0‐3_ (from the triazole) B, C (both from the phenyl), D (from the terminal methyl group of the PEG_12_ chains) were integrated, showing an integration of 12/4/4/30 (Figures S78, S86). The results show that **8** **b** and **8** **c** each have 12 triazole rings from the click reactions (2 from **7** **b**, **7** **c**, 10 from new ligand attachment), and 10 PEG_12_ chains (30H from terminal methyl groups), which suggest the reactions were complete with 10 ligands. The precarious MS characterization of the water‐soluble MBTs requires carefully striking a balance between low laser power to suppress fragmentation and high gain voltage of the dual‐stage reflector to increase signal strength, which was only accomplished for shorter linker versions **8** **c** and **8** **e** with limited signal‐to‐noise ratio (Figures S85, S91), but nevertheless provide additional solid evidence for the successful introduction of 10 ligands with a defined molecular weight.

**Figure 3:**
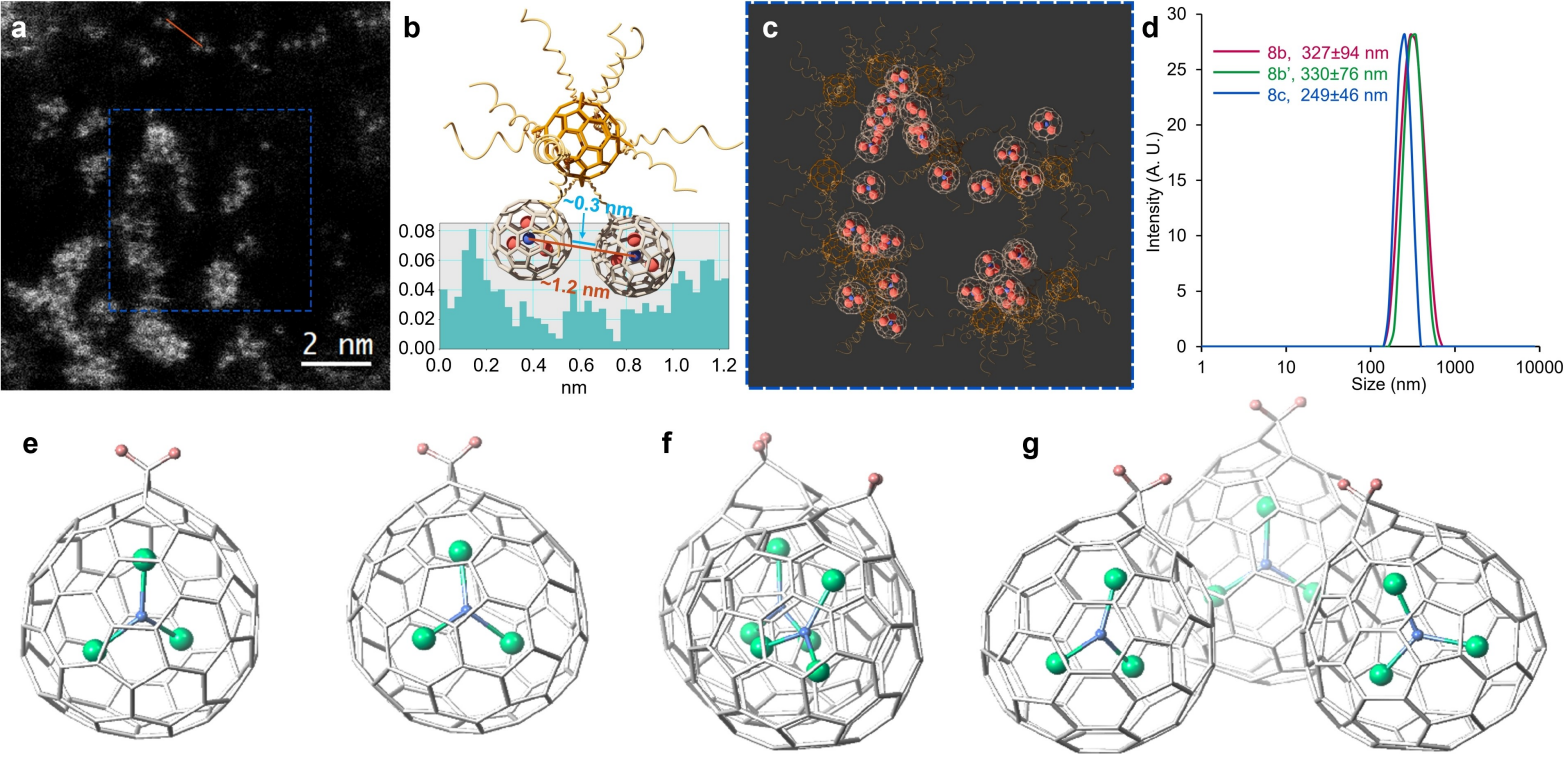
Investigation of the aggregation and supramolecular interactions of MBTs **8**. a) High‐angle annular dark‐field (HAADF) STEM image of **8** **e**. Gd atoms appear to be the only visible species in the image as their intensities overshadow the light elements in the molecule. b) Schematic representation of one **8** **e** molecule and histogram corresponding to the orange line in a). The N−N distance from two adjacent clusters is ≈1.2 nm, which translates to an EMF cage edge‐to‐edge distance of ≈0.3 nm after subtracting the EMF cage diameter. c) Schematic representation of the area in the blue dotted line in a), exemplifying the possible positions of the C_60_ core and ligands that are barely visible in the HAADF image. d) DLS spectra of **8** **b**, **8** **b′** and **8** **c**, 1 μM solution in water. e) “side” and f) “superimposed” views of the most realistic DFT optimized structure obtained for (Lu_3_N@C_80_‐CH_2_)_2_ dimer. g) optimized structure for the (Lu_3_N@C_80_‐CH_2_)_3_ trimer.

With the EMF cages in **8** remaining aromatic and hydrophobic, the molecules are amphiphilic, and we hypothesize they will aggregate driven by hydrophobic and/or π‐π interactions.^[34]^ To verify the hypothesis, a drop‐cast sample of **8** **e** was studied with a scanning transmission electron microscope (STEM) with atomic resolution (Figure 3a). The brightness of the Gd atoms (due to high Z‐number) overshadow all lighter atoms,^[61]^ which made Gd practically the only visible elements in the image. However, the positions of Gd atoms are sufficient to reveal the positions of the EMFs. Most molecules exist in large aggregates across the imaging areas, while Figure 3a was captured at the edge of an aggregate to show discrete Gd_3_N clusters. In pairs of neighboring Gd_3_N clusters that are in the same focus plane, presumably from the same molecule, the center‐to‐center (N−N) distance (orange line in Figure 3a) was typically ≈1.2 nm, which, after subtracting the cage diameter, translates to an edge‐to‐edge distance of approximately 0.3 nm (Figure 3b), within the effective range for π‐π interactions. This finding suggests the strong intramolecular interaction between the two EMFs outcompetes the entropic punishment from the conformational restriction of the flexible linkers. Of the same nature, intermolecular interactions among EMF cages will provide a strong driving force for aggregation as seen in Figure 3a and, partially depicted in Figure 3c indicating possible positions of the “invisible” buckyballs and ligands. In an aqueous solution, such aggregation would eventually lead to the formation of micelles. Dynamic light scattering (DLS) on **8** **b**, **8** **b′** and **8** **c** revealed that the MBTs exist in large 150–500 nm micelles in water (Figure 3d), among which **8** **c** (≈250 nm) forms smaller micelles than **8** **b** and **8** **b′** (≈330 nm) presumably due to the shorter linker between C_60_ and EMFs that induces higher steric hindrance against EMF interactions.

To better understand the aggregation of these molecules, we used computational approach to analyze the strength of the interactions between nearby EMFs, using a simplified model of a dimer (Lu_3_N@C_80_‐CH_2_)_2_ where the substituted methano group is replaced by CH_2_. Geometry optimizations using the PBE functional (computational details in the Supporting Information) for different relative orientations of the two Lu_3_N@C_80_‐CH_2_ units within the dimer show significant interaction energies that range between −10 and −15 kcal mol^−1^ (Figure S98). A few orientations that present the highest interaction energies in the simplified models are not compatible with the constraints imposed by the actual experimental MBTs where the EMFs are linked to the C_60_ hexakisadduct. For that reason, we find more likely a relative orientation of the two dimers as the one shown in Figure 3e/f (also Ori7 in Figure S98) with an interaction energy of −12.4 kcal mol^−1^. In this optimized structure, the shortest C⋅⋅⋅C distance between the two Lu_3_N@C_80_‐CH_2_ cages is 3.26 Å and the N⋅⋅⋅N distance is 11.0 Å, in excellent agreement with experiments. At 3.26 Å, fullerene‐fullerene interactions are far from being negligible. When increasing the shortest C⋅⋅⋅C distance in our model dimer, the interaction energy decreases to a value of −4.3 kcal mol^−1^ at 6.0 Å (Table S2), which is still significant. We have also estimated the interaction energy for the next step in the aggregation process, i.e., adding a third EMF to the dimer. When the three EMFs are placed in a triangular arrangement with shortest C⋅⋅⋅C distances of 3.35 Å (Figure 3g and Figure S99), the interaction energy increases up to −27 kcal mol^−1^. By increasing the distance between the EMFs the interaction energy does not decay abruptly (Table S3), similarly to the case of the dimer. Finally, to get more insight into the nature of this interaction, we have compared model systems with (i) different amounts of formal charge transfer keeping the same carbon cage; and (ii) different cage sizes. When comparing (Lu_3_N@C_80_‐CH_2_)_2_, and the hypothetical (Lu_2_O@C_80_‐CH_2_)_2_ and empty (C_80_‐CH_2_)_2_, with formal charge transfers of six, four and zero, respectively,[^12^, ^62^, ^63^] the interaction energies decrease from −12.4 to −10.8 and −9.3 kcal mol^−1^ (Table S4). These results clearly show that the charge transfer from the cluster to the cage enhances the well‐known π‐π interaction between fullerene cages.^[64]^ It is also relevant to remark the effect of the fullerene size. If we compare (C_80_‐CH_2_)_2_ and (C_60_‐CH_2_)_2_ the interaction energy is slightly larger for the cage that allows higher surface contact, i.e. C_80_, (−9.3 vs −7.1 kcal mol^−1^, Table S4 and Figure S100). Indeed, the different supramolecular chemistry exhibited by empty fullerenes and EMFs is the basis of the selective encapsulation and purification of EMFs. In this context, the nature of the cluster and the shape of the fullerene are highly relevant.[^65^, ^66^] In summary, the supramolecular π‐π interactions between Lu_3_N@C_80_‐CR_2_ entities in MBTs are significant and likely the driving force for the observed aggregation, even though hydrophobic interactions may also help.

#### Biomedical Properties of the Water‐Soluble MBTs

**Figure 4:**
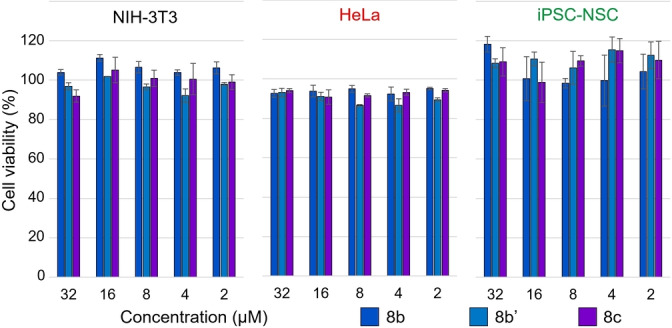
Cell viability tests of MBTs **8** **b**, **8** **b′** and **8** **c** at concentrations of 2–32 μM against NIH‐3T3, HeLa, and iPSC‐NSC cell lines. Error bars indicate standard deviation of individual cell viability data set.

The safety of water‐soluble MBTs was first established. First, we used inductively coupled plasma mass spectrometry (ICP‐MS) with a detection limit <0.1 ppb to confirm the EMF cage confinement of the metal ions (procedure in the Supporting Information). Then their cytotoxicity towards three cell lines, namely, normal cell line NIH‐3T3 from mouse fibroblast, cancer HeLa cells of cervical cancer origin, and stem cell line induced pluripotent stem cell—neural stem cell (iPSC‐NSC),[^67^, ^68^] were studied using a standard resazurin‐based cell metabolic assay. From these comprehensive cytotoxicity tests, MBTs were found to be generally non‐toxic across the board (Figure 4). The results suggest MBT structures have high biocompatibility and biosafety when used for a variety of applications including in vivo animal imaging studies, common cancer diagnostic applications, or as imaging probes in stem cell research.

**Figure 5:**
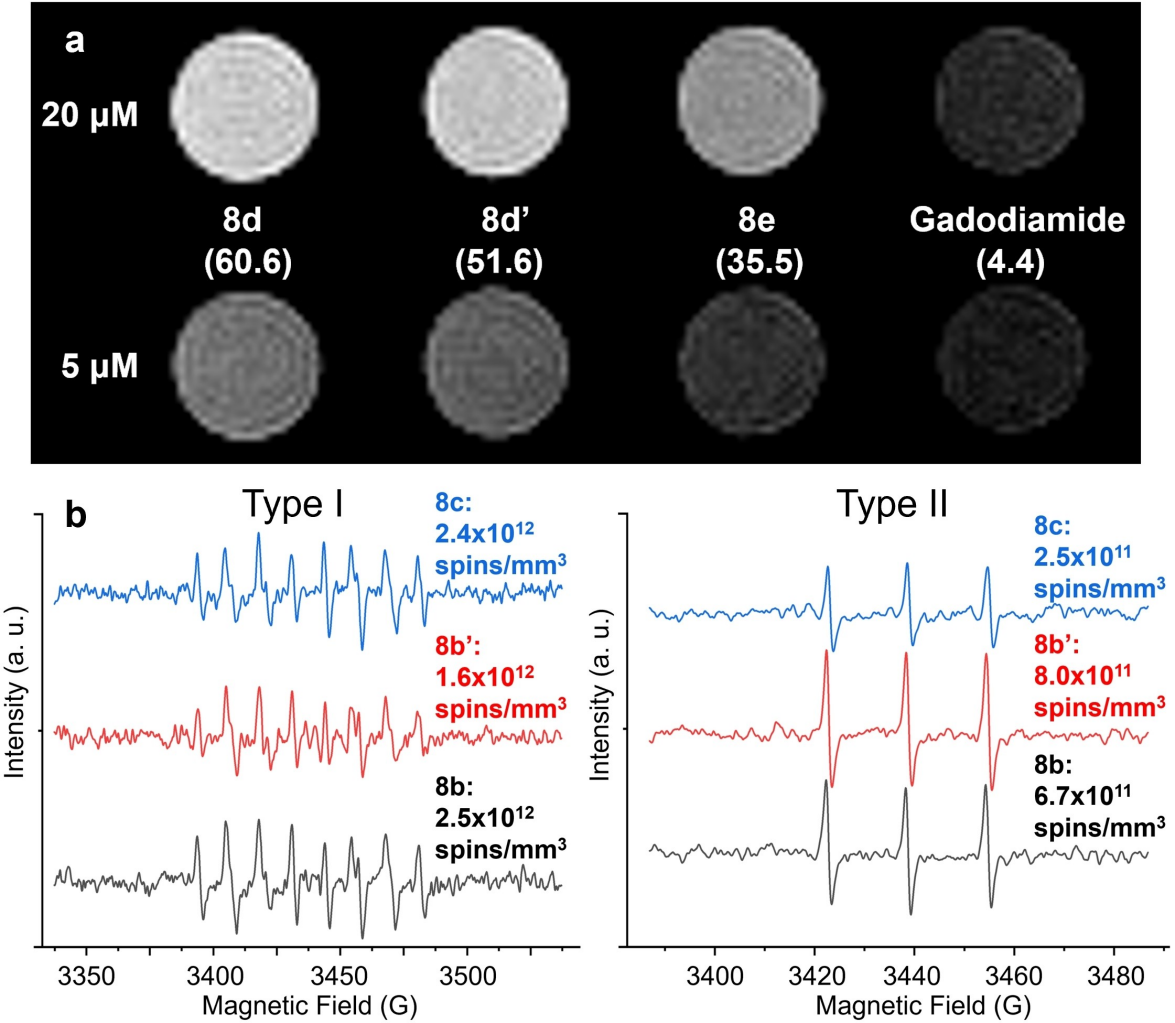
Potential biomedical applications of the water‐soluble MBTs. a) MR imaging of Gd MBT solutions on a 1.0 T scanner. The measured *r*_1_ values at 1.4 T are written in parentheses under each compound, in the unit of mM^−1^⋅s^−1^. b) EPR spectra of the stable spin traps measuring the Type I (left) and Type II (right) ROS generation upon light irradiation of Lu MBTs.

The *T*_1_ relaxivity values of water‐soluble Gd MBTs were tested on 60 MHz (1.4 T) and 300 MHz (7.1 T) NMR spectrometers (Table S1), together with a representative commercial GBCA gadodiamide (i.e., Omniscan^TM^). At 1.4 T, the more clinically relevant field strength, **8** **d**, **8** **d′**, **8** **e** exhibited *r*_1_ of 60.6, 51.6, and 35.5 mM^−1^ s^−1^, respectively, one order of magnitude higher than an *r*_1_ of 4.4 mM^−1^ s^−1^ for gadodiamide. The enhanced contrast was directly visualized on a 1.0 T MRI scanner, with 20 μM and 5 μM **8** **d**, **8** **d′**, **8** **e**, and gadodiamide (Figure 5a), and the imaging contrast is consistent with the measured relaxivity values. The preclusion of ion leakage by the robust EMF cage dictates that the real limiting factor of MBT contrast agents is the toxicity of the MBT structure, not individual Gd^3+^ ions, which means the molecular relaxivity of the MBTs is the more appropriate and relevant parameter for clinical evaluations. Meanwhile, even on a per Gd basis, the MBTs still show significantly higher relaxivity than gadodiamide at 1.4 T, despite the lack of direct hydration of the Gd^3+^ ion, likely due to the large hydrodynamic size of the aggregates, which increase the rotational correlation time of the contrast agents.[^24^, ^28^, ^29^] The precise design enabled the fundamental understanding extracted from the comparison among **8** **d**, **8** **d′** and **8** **e**, which suggests the linker lengths between the C_60_ core and the EMFs are crucial. Within a few ethylene glycol units, longer linkers considerably increase *r*_1_. We rationalize the enhancement with two factors. First, as mentioned above, with longer linkers **8** **d** and **8** **d′** form larger micelles thanks to the lower steric hindrance. Second, and more importantly, despite the 10 solubilizing ligands at the periphery, these linkers are the direct hydrophilic contributor in the proximity of the Gd EMF cages, making them the main force to attract water molecules near the EMFs for Gd^3+^‐H_2_O interaction. On the other hand, the length of the peripheral ligands did not substantially affect the relaxivity based on the comparison between **8** **d** and **8** **d′**, which promises vast freedom to “dial‐in” desirable biochemical ligands (e.g., peptides, aptamers) for targeted GBCAs with little concern for relaxivity loss, within the lower thousand Dalton range. Notably, the MBT system provide important insight of ^1^H relaxation of Gd^3+^ ions in a carbon cage without protic functional groups (e.g., −OH, NH_2_, −COOH) on it, which is also vital for future design of GBCAs based on other precise EMF derivatives (e.g., mono‐, bisadducts) or host–guest systems containing pristine EMFs.

Another key feature of the MBT design distinct from traditional water‐soluble EMF mixtures is the mostly preserved conjugated EMF cage. With safely encapsulated heavy atoms that facilitate ISC, they are very promising photosensitizers. To confirm this, the Lu MBTs **8** **b**, **8** **b′**, **8** **c** solutions were irradiated using a green LED, and the generated ROS were quantitatively converted to stable radicals by spin traps, and quantified with EPR signals, following an established approach.^[69]^ For Type I ROS, i.e., superoxide radical anion O_2_⋅^−^, spin trap 5‐(diethoxyphosphoryl)‐5‐methyl‐1‐pyrroline‐N‐oxide (DEPMPO) was used with NADH, diethylenetriaminepentaacetic acid, and L‐histidine, which is a known singlet oxygen blocker^[70]^ to correct for indirectly generated superoxide anion from singlet oxygen. For Type II ROS, i.e., singlet oxygen, spin trap 2,2,6,6‐tetramethyl‐4‐piperidone (4‐oxo‐TEMP) was used. As shown in Figure 5b, all MBTs showed a strong preference for the Type I pathway (≈70–90 % spin counts). Meanwhile, **8** **b** showed highest overall ROS (Type I+Type II) generation, while its paramagnetic counterpart **8** **d** has the highest relaxivity. These results indicate that the factor that is important for relaxivity, i.e., accessibility of water (containing O_2_), is likely also a critical factor in ROS generation, although the influence is not as direct as in the case of ^1^H relaxivity. Remarkably, a recent study showing that lack of water access contributed to an ultra‐long‐lived triplet state from a C_60_ hexakisadduct based giant molecule,^[49]^ confirming that the photoreaction pathways and ROS generation can be tuned by the linker between the EMF and C_60_ core, and the external ligands. More photophysical behavior of the MBT series, and their utility in PDT are currently being investigated.

## Conclusion

We have established the MBT platform that enabled the development of a series of structurally defined water‐soluble EMF derivatives. As revealed by both experimental and computational studies, the resulting water‐soluble MBTs form large aggregates with strong π‐π interactions among EMF cages. The MBTs have definitive metal seal and excellent biocompatibility. The Gd MBTs are efficient MRI contrast agents with higher *T*_1_ relaxivity and enhanced imaging contrast compared to the well‐established GBCA gadodiamide. The preserved EMF cages in MBTs are capable of ROS generation under light irradiation, which shows promise as photosensitizers for PDT. Both the MRI contrast enhancement and ROS generation show a dependence on the molecular structural parameters, suggesting further optimization for water access can be an important future direction. With the built‐in capacity to take multivalent biological ligands by the user's choice, MBT represents a new strategy towards safe and bio‐specific REE drugs with EMFs.

## Conflict of interest

The authors declare no conflict of interest.

1

## Supporting information

As a service to our authors and readers, this journal provides supporting information supplied by the authors. Such materials are peer reviewed and may be re‐organized for online delivery, but are not copy‐edited or typeset. Technical support issues arising from supporting information (other than missing files) should be addressed to the authors.

Supporting Information

## Data Availability

The data that support the findings of this study are available in the supplementary material of this article.
