# Supplementary material for: Structurally Defined Water‐Soluble Metallofullerene Derivatives towards Biomedical Applications
Source: Angew Chem Int Ed Engl. 2022 Dec 2;62(3):e202211704. doi: 10.1002/anie.202211704 (PMC9983306; doi:10.1002/anie.202211704)
Supplement: Supplementary file 1 — Supporting Information [file ANIE-62-0-s001.pdf]

Angewandte Chemie
Eine Zeitschrift der Gesellschaft Deutscher Chemiker

Supporting Information

Structurally Defined Water-Soluble Metallofullerene Derivatives towards Biomedical Applications

*Y. Li, R. Biswas, W. P. Kopcha, T. Dubroca, L. Abella, Y. Sun, R. A. Crichton, C. Rathnam, L. Yang, Y.-W. Yeh, K. Kundu, A. Rodríguez-Fortea, J. M. Poblet, K.-B. Lee, S. Hill, J. Zhang**

Table of Contents

1. Materials & instruments S2

2. Supplemental experimental procedures for compounds 4-8 S3-S19

2.1 Synthesis of [60]fullerene hexakis-adduct [5:1] S3-S4

2.2 Synthesis of endohedral metallofullerene monoderivatives S4-S10

2.3 Synthesis of Metallobuckytrios (MBTs) S11-S19

3. Supplemental procedures for experiments and computations including:

stem imaging, dynamic light scattering (DLS) Measurements, inductively coupled plasma-mass spectrometry (ICP-MS), metal ion leaking experiment, cell viability study, relaxivity measurements, magnetic resonance imaging, ROS generation and measurements, DFT calculation S20-S22

4. Supplemental characterization data S23-S86

5. Supplemental references S87

1. Materials & instruments

General chemicals and solvents were used as received from common vendors including Sigma Aldrich, Fisher Scientific, TCI America, etc. Some solvents are purchased in anhydrous grade sealed in an inert atmosphere if anhydrous solvent is noted in the procedures. ICP-MS metal standards were purchased from High Purity Standards. C_{60} was purchased from Henan Fullerene Co. Ltd. Endohedral metallofullerenes were purchased from Luna Innovations Inc. TEM grids were purchased from Ted Pella. Dialysis membranes were purchased from Spectra/Por. iPSC-derived neural stem cells, cells were derived from human iPSCs (WT126 clone 8; and WT33 clone 1) and grown in proliferation media. Other cell lines are cultured in DMEM from commercial cell lines: NIH-3T3 (Sigma), HeLa (ATCC).

All synthetic steps were carried out under atmosphere conditions unless otherwise noted. The NMR spectra were obtained at 25 °C with 400, 500 or 600 MHz spectrometers. Chemical shifts are given in ppm relative to TMS, calibrated with either TMS peak or known chemical shifts of $CDCl_3$ or $CDCl_2CDCl_2$. HPLC was performed on Agilent Infinity 1220 or Infinity 1260, with 5-PBB, 5-PYE, Buckyprep-D columns as noted in the chromatograms. MALDI-TOF MS spectra were recorded on a Bruker UltraFlex III. Chromatographic purifications were carried out with standard 230-400 mesh silica gel.

STEM Imaging was performed using a Nion UltraSTEM 100 with an aberration corrector and at an operating voltage of 60 kV.

Dynamic light scattering experiments were performed using a Malvern Zetasizer Nano ZS90 instrument with a standard laser source (4mW, 633nm).

Dialysis membrane: Spectra/Por standard grade regenerated cellulose dialysis membranes (MWCO: 1 kD and 3.5 kD) were stored in 0.5% sodium azide solution. Prior to use, dialysis bags of desired sizes were soaked in DI water for at-least 30 minutes before loading the sample.

Inductively Coupled-Plasma Mass Spectrometry (ICP-MS) elemental analysis was performed using a Thermo Scientific TM iCAP Q instrument.

T_1 relaxation time measurements were performed at two different magnetic fields using Nanalysis NMReady-60e desktop 60 MHz (1.4 T) instrument and Varian^{TM} Oxford VNMRS 300 MHz (7.1 T) instrument.

The ROS generation was induced by green light from an LED strip affixed in an ellicoidal pattern inside the cylindrical enclosure. The LED strip was purchased from Bestledstrip, Santa Ana, California, USA.

EPR spectra were recorded on a Bruker EMX nano X-band spectrometer.

Note: common impurities such as residue solvent toluene and grease from silica gel are sometimes impossible to be removed from the fullerene derivatives by routine flash column chromatography. Further purification by a diffusion-precipitation procedure was usually required to obtain pure samples. The diffusion-precipitation solvents are carbon disulfide (CS_2)/acetonitrile (CH_3CN) or CS_2/ethanol.

2. Supplemental experimental procedures for compounds 4-8

2.1 Synthesis of [60]fullerene hexakis-adduct [5:1]

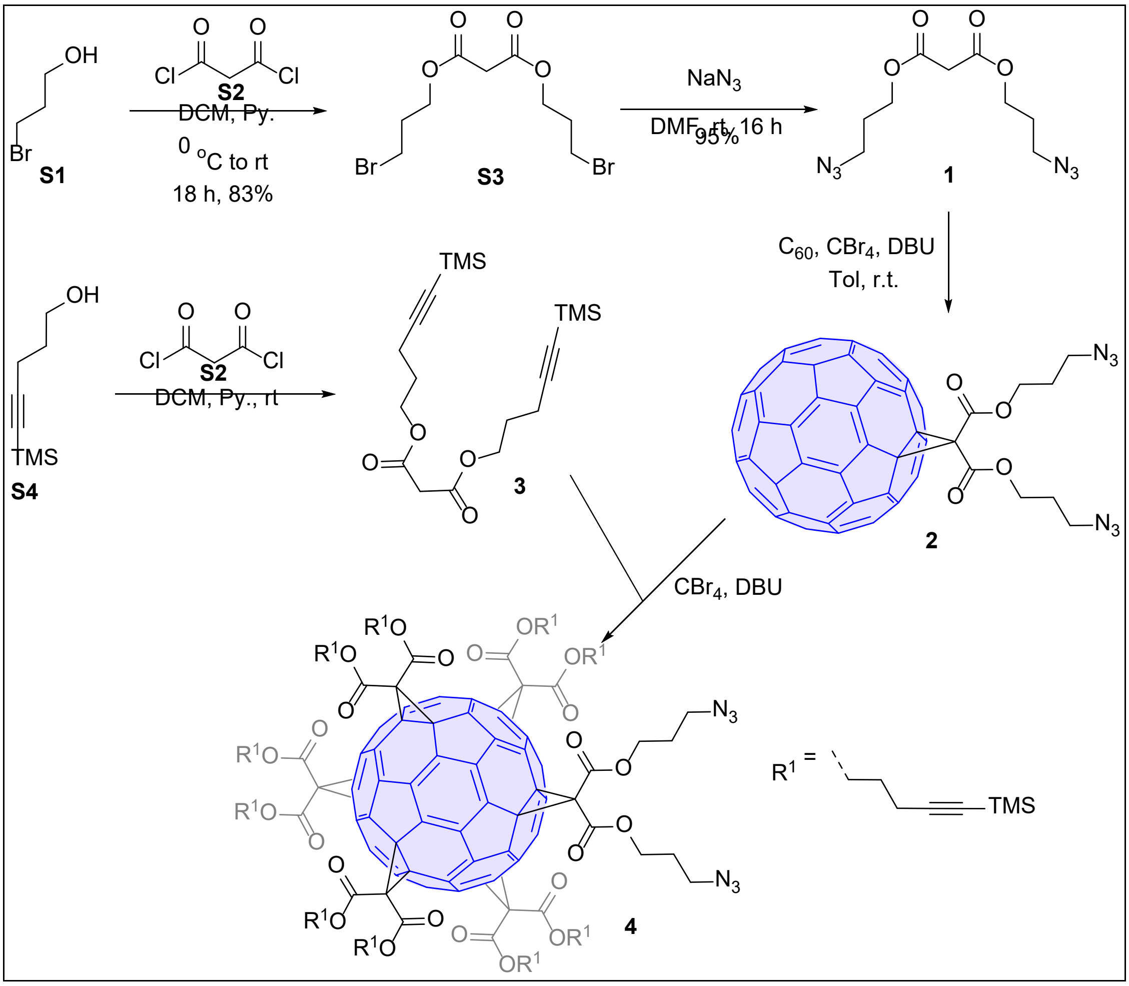

Scheme S1: Synthesis of [60]fullerene hexakis-adduct [5:1]

In Scheme S1, compound **1** was synthesized from compound **S1** via two steps based on the literatures[1-2].

In Scheme S1, compound **3** was synthesized from compound **S4** based on the literatures[3-4].

Preparation of compound 4:

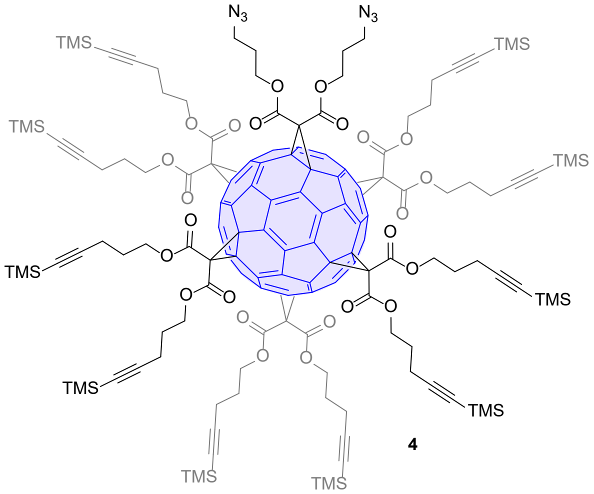

A solution of C_{60} (300 mg, 0.416 mmol), bis(3-azidopropyl) malonate **1** (112 mg, 0.416 mmol, 1 eq.) and CBr_4 (138 mg, 0.416 mmol, 1.0 eq.) in 85 mL of toluene was bubbled with nitrogen for 20 minutes. Subsequently, 1,8-Diazabicyclo[5.4.0]undec-7-ene (DBU, 93 μL, 0.624 mmol, 1.5 eq.) was added and the then flask was sealed with a septum. The resulting solution was stirred for 3 hours at room temperature, after which it was applied directly to a silica gel column and eluted with toluene. The first band (purple, unreacted C_{60}) was not collected, and the second (maroon, compound **2**) was concentrated under vacuum to a volume of approximately 150 mL. This solution was used without

further purification to produce **4** by adding bis(5-trimethylsilyl-4-pentynyl) malonate **3** (792 mg, 2.08 mmol, 5.0 eq.) and CBr_4 (6.898 g, 20.8 mmol, 50 eq.), bubbling with nitrogen, adding DBU (622 μL, 4.16 mmol, 10.0 eq.), and sealing with a septum. This mixture was stirred for 4 days at room temperature, during which time the solution changed from maroon to orange and a tan precipitate could be seen collecting on the sides of the reaction flask. The solution was concentrated under vacuum, applied to a silica column, and eluted with CS_2/ethyl acetate (EA) (gradient increase from 100 : 2 to 100 : 3 to 100 : 4). Product **4** was isolated as an orange, glassy solid (142 mg, 0.050 mmol, 12%).

Characterization data for 4:

^{1}H NMR (400 MHz, $CDCl_3$) δ 4.43-4.30 (m, 24H, OC**H$_2$**CH$_2$CH$_2$N$_3$ and OC**H$_2$**CH$_2$CH$_2$CCTMS), 3.42-3.37 (m, 4H, OCH$_2$CH$_2$C**H$_2$**N$_3$), 2.41-2.26 (m, 20H, OCH$_2$CH$_2$C**H$_2$**CCTMS), 2.03-1.84 (m, 24H, OCH$_2$C**H$_2$**CH$_2$N$_3$ and OCH$_2$C**H$_2$**CH$_2$CCTMS), 0.17-0.13 (m, 90H, TMS).

^{13}C NMR (126 MHz, $CDCl_3$) δ 163.57-163.52 (**C**OO), 145.89-145.74 (sp^2 **C** of the fullerene cage), 141.06-140.90 (the other sp^2 **C** of the fullerene cage), 105.10 (sp **C** of alkyne), 85.76 (sp **C** of alkyne), 69.08-69.04 (sp^3 **C** of the fullerene cage), 65.69-65.65 (O**C**H$_2$CH$_2$CH$_2$CCTMS), 63.76 (O**C**H$_2$CH$_2$CH$_2$N$_3$), 47.86 (OCH$_2$CH$_2$**C**H$_2$N$_3$), 45.22 (bridge **C** of malonate), 28.02 (OCH$_2$**C**H$_2$CH$_2$N$_3$), 27.50 (OCH$_2$CH$_2$**C**H$_2$CCTMS), 16.51 (OCH$_2$**C**H$_2$CH$_2$CCTMS), 0.13-0.11 (TMS).

MALDI-TOF-Positive $C_{73}H_{18}NO_4$ ([M]$^{\bullet+}$) m/z calculated 2878.9, found 2879.1.

2.2 Synthesis of endohedral metallofullerene monoderivatives

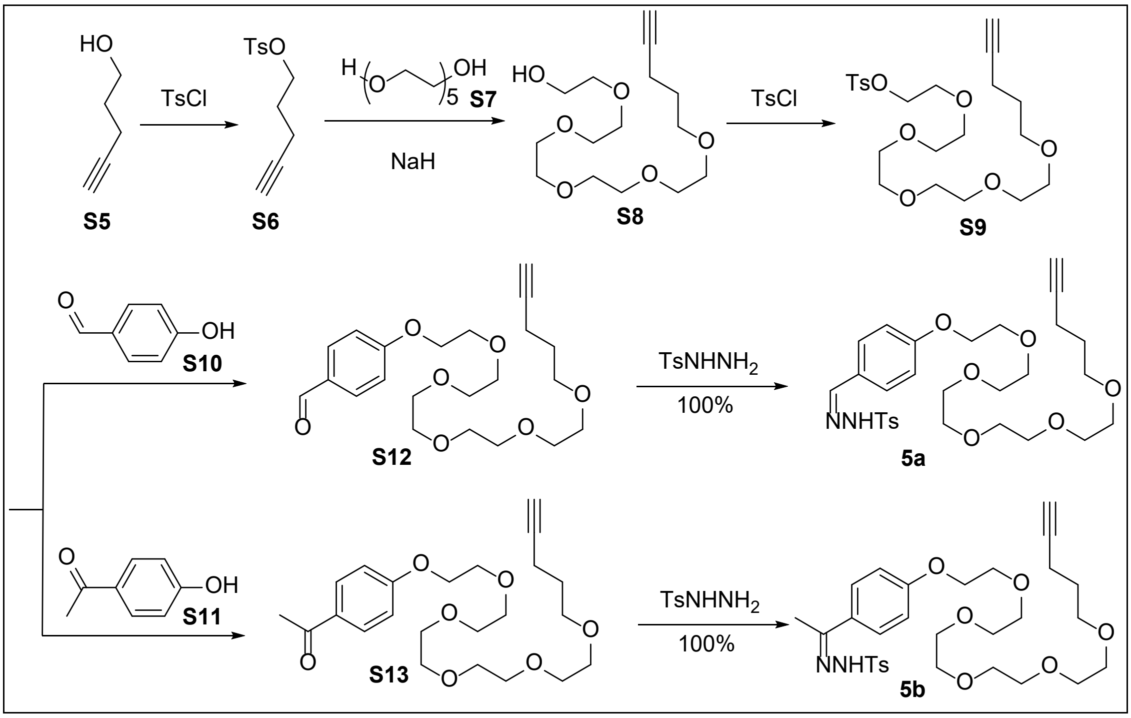

Scheme S2: Synthesis of precursors **5a** and **5b**

In Scheme S2, compound **S6** was synthesized from compound **S5** based on the literatures.[5-6]

Preparation of Compound S9:

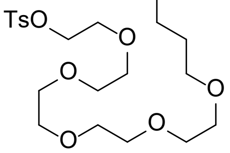

S9

To a solution of **S7** (8.81 g, 36.93 mmol, 1.0 eq.) in 190 mL of anhydrous tetrahydrofuran (THF), NaH (1.33 g, 33.6 mmol, 0.9 eq., 60% in oil) was slowly added under nitrogen atmosphere at 0 °C. The resulting solution was stirred for 1 hour and then a solution of **S6** (8.0 g, 33.6 mmol, 0.9 eq.) in 10 mL anhydrous THF was added. The solution was stirred at room temperature overnight and then quenched with water. The mixture was extracted by dichloromethane (DCM, 3 x 200 mL). The organic layers were combined, washed with water (200 mL) and brine (200 mL), dried over Na_2SO_4,

filtered and concentrated under vacuum. The crude **S8** was used in the next step without further purification. A solution of crude **S8** in 12 mL of THF was prepared and added to a solution of NaOH (2.76 g, 69.0 mmol) in 10 mL of water. p-Toluenesulfonyl chloride (9.92 g, 52.0 mmol) was dissolved in 10 mL anhydrous THF and added to the above solution dropwise at 0 °C. The resulting solution was stirred overnight at room temperature. The reaction was quenched by adding ice-cold water (30 mL). The mixture was extracted by DCM (3 x 30 mL). The organic layers were combined, washed with water (90 mL) and brine (90 mL), dried over Na_2SO_4, filtered and concentrated under vacuum. The mixture was loaded in a silica gel column. Upon elution using DCM/EA (4 : 1), gave compound **S9** as a light yellow oil (5.36 g, 11.70 mmol, 35% based on **S6**).

Characterization data for S9:

^{1}H NMR (500 MHz, $CDCl_3$) *δ* 7.81-7.78 (m, 2H), 7.35-7.33 (m, 2H), 4.16-4.15 (m, 2H), 3.70-3.58 (m, 18H), 3.55 (t, *J* = 6.3 Hz, 2H), 2.44 (s, 3H), 2.28 (td, *J* = 2.7, 7.1 Hz, 2H), 1.93 (t, *J* = 2.7 Hz, 1H), 1.81-1.76 (m, 2H).

^{13}C NMR (126 MHz, $CDCl_3$) *δ* 144.76, 133.02, 129.80, 127.97, 83.98, 70.74, 70.60, 70.56, 70.51, 70.22, 69.56, 69.22, 68.66, 68.42, 28.51, 21.62, 15.16.

Preparation of Compound S12:

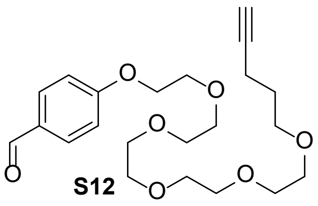

S12

A solution of **S9** (2.02 g, 4.36 mmol, 1.0 eq.) in 25 mL CH_3CN was added 4-hydroxybenzaldehyde (0.81 g, 6.55 mmol, 1.5 eq.), K_2CO_3 (1.81 g, 13.08 mmol, 3.0 eq.) and KI (0.05 g, 0.33 mmol, 0.08 eq.) under nitrogen atmosphere. Reaction mixture was stirred at 80 °C overnight. Upon complete consumption of **S9** based on TLC, the reaction mixture was filtered, and the solvent was removed under vacuum. The residue was redissolved in DCM and loaded in a silica gel column. Upon elution in DCM/EA (7:3), gave compound **S12** as a yellow oil (1.1 g, 2.7 mmol, 62 %).

Characterization data for S12:

^{1}H NMR (500 MHz, $CDCl_3$) *δ* 9.86 (s, 1H), 7.82-7.79 (m, 2H), 7.01-6.99 (m, 2H), 4.21-4.19 (m, 2H), 3.88-3.86 (m, 2H), 3.72-3.70 (m, 2H), 3.67-3.60 (m, 12H), 3.58-3.56 (m, 2H), 3.53 (t, *J* = 6.2 Hz, 2H), 2.26 (td, *J* = 7.1, 2.7 Hz, 2H), 1.92 (t, *J* = 2.7 Hz, 1H), 1.80-1.74 (m, 2H).

^{13}C NMR (126 MHz, $CDCl_3$) *δ* 190.74, 163.81, 131.90, 130.00, 114.85, 83.95, 70.87, 70.60, 70.56, 70.53, 70.20, 69.53, 69.43, 68.45, 67.74, 28.48, 15.14.

Preparation of Compound S13:

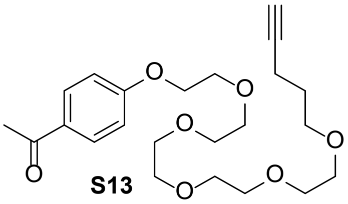

S13

A solution of **S9** (1.1 g, 2.2 mmol, 1.0 eq.) in 12 mL CH_3CN was added 4-hydroxyacetophenone (0.45 g, 3.27 mmol, 1.5 eq.), K_2CO_3 (0.92 g, 6.66 mmol, 3.0 eq.) and KI (0.03 g, 0.16 mmol, 0.08 eq.) under nitrogen atmosphere. Reaction mixture was stirred at 80 °C overnight. Upon the complete consumption of **S9** based on TLC, the reaction mixture was filtered, and the solvent was removed under vacuum. The residue was redissolved in a small amount of DCM and loaded in a silica gel column. Upon elution in DCM/EA (3:2), gave compound **S12** as a yellow oil (0.77 g, 1.8 mmol, 83 %).

Characterization data for S13:

^{1}H NMR (500 MHz, $CDCl_3$) *δ* 7.93-7.91 (m, 2H), 6.95-6.93 (m, 2H), 4.20-4.18 (m, 2H), 3.89-3.87 (m, 2H), 3.73-3.71 (m, 2H), 3.69-3.62 (m, 12H), 3.59-3.58 (m, 2H), 3.54 (t, *J* = 6.2 Hz, 2H), 2.55 (s, 3H), 2.27 (td, *J* = 7.1, 2.7 Hz, 2H), 1.93 (t, *J* = 2.7 Hz, 1H), 1.80-1.77 (m, 2H).

^{13}C NMR (126 MHz, $CDCl_3$) δ 196.78, 162.73, 130.56, 130.46, 114.28, 84.00, 70.91, 70.65, 70.61, 70.59, 70.25, 69.59, 69.54, 68.46, 67.63, 28.52, 26.36, 15.19.

Preparation of Compound 5a:

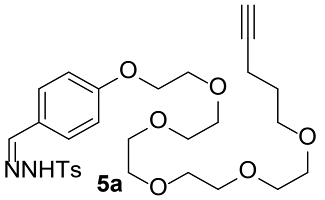

S12 (1.1 g, 2.7 mmol, 1.0 eq.) was dissolved in 100 mL of 1,2-dichloroethane, then $TsNHNH_2$ (3.0 g, 16.1 mmol, 6.0 eq.) was added. The resulting solution was stirred at 100 °C under N_2 atmosphere overnight and cooled down after TLC showed no further change. The solution was then concentrated under vacuum and loaded onto a silica gel column. Eluting with DCM/EA (4 : 1) removed the unreacted $TsNHNH_2$. Eluting with DCM/EA (2 : 1) gave compound **5a** (1.55 g, 2.7 mmol, 100%).

Characterization data for 5a:

^{1}H NMR (500 MHz, $CDCl_3$) δ 7.91 (s, 1H), 7.87-7.85 (m, 2H), 7.71 (s, 1H), 7.49-7.46 (m, 2H), 7.31-7.29 (m, 2H), 6.86-6.83 (m, 2H), 4.11-4.09 (m, 2H), 3.84-3.82 (m, 2H), 3.72-3.69 (m, 2H), 3.68-3.60 (m, 12H), 3.59-3.56 (m, 2H), 3.54 (t, J = 6.3 Hz, 2H), 2.40 (s, 3H), 2.27 (td, J = 7.1, 2.7 Hz, 2H), 1.93 (t, J = 2.7 Hz, 1H), 1.80-1.75 (m, 2H).

^{13}C NMR (126 MHz, $CDCl_3$) δ 160.66, 148.12, 144.08, 135.45, 129.60, 128.90, 127.93, 126.03, 114.71, 83.97, 70.83, 70.60, 70.58, 70.54, 70.20, 69.58, 69.56, 68.45, 67.46, 28.47, 21.57, 15.15.

Preparation of Compound 5b:

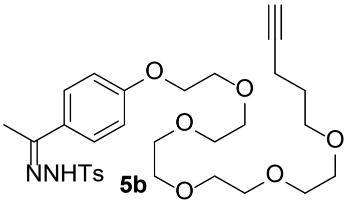

S13 (775 mg, 1.8 mmol, 1.0 eq.) was dissolved in 80 mL of 1,2-dichloroethane, then $TsNHNH_2$ (2.0 g, 10.7 mmol, 6.0 eq.) was added. The resulting solution was stirred at 100 °C under N_2 atmosphere overnight and cooled down after TLC showed no further change. The solution was then concentrated under vacuum and loaded onto a silica gel column. Eluting with DCM/EA (4 : 1) removed the unreacted $TsNHNH_2$. Eluting with DCM/EA (1 : 1) gave compound **5b** (1.08 g, 1.8 mmol, 100%).

Characterization data for 5b:

^{1}H NMR (500 MHz, $CDCl_3$) δ 7.92-7.90 (m, 2H), 7.60-7.53 (m, 2H), 7.53 (s, 1H), 7.33-7.30 (m, 2H), 6.87-6.84 (m, 2H), 4.12 (dd, J = 5.7, 4.0 Hz, 2H), 3.86-3.84 (m, 2H), 3.73-3.71 (m, 2H), 3.68-3.61 (m, 12H), 3.59-3.57 (m, 2H), 3.54 (t, J = 6.3 Hz, 2H), 2.41 (s, 3H), 2.27 (td, J = 7.1, 2.7 Hz, 2H), 2.11 (s, 3H), 1.93 (t, J = 2.7 Hz, 1H), 1.81-1.76 (m, 2H).

^{13}C NMR (126 MHz, $CDCl_3$) δ 160.00, 152.68, 144.01, 135.46, 129.87, 129.50, 128.11, 127.67, 114.24, 83.96, 70.81, 70.57, 70.53, 70.19, 69.59, 69.53, 68.42, 67.39, 28.46, 21.56, 15.13, 13.23.

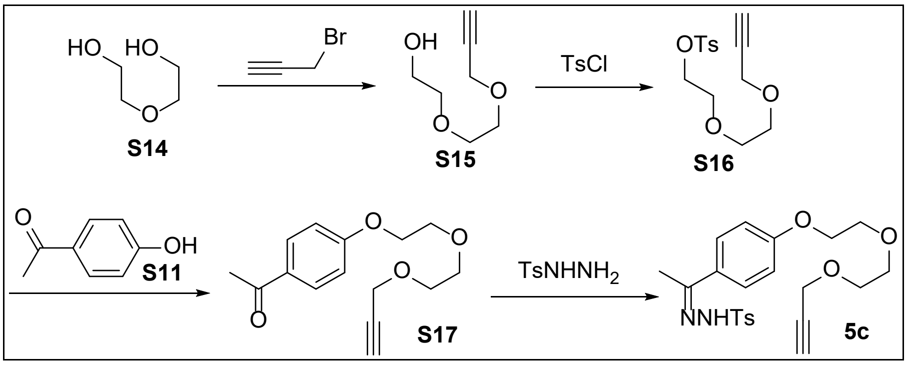

Scheme S3: Synthesis of precursor **5c**

In Scheme S3, compound **S16** was synthesized from compound **S14** based on the literatures.[7-8]

Preparation of Compound S17:

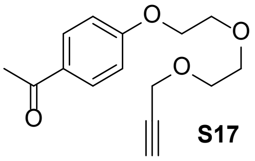

S17

A solution of **S16** (1.9 g, 6.5 mmol, 1.0 eq.) in 35 mL CH_3CN was added to a mixture of 4-hydroxyacetophenone (1.3 g, 9.7 mmol, 1.5 eq.), K_2CO_3 (2.7 g, 19.4 mmol, 3.0 eq.) and KI (0.08 g, 0.5 mmol, 0.075 eq.) and bubbled with nitrogen for 15 minutes and then this reaction mixture was stirred at 80 °C overnight. Upon complete consumption of **S16** based on TLC, reaction mixture was filtered, and the solvent was removed under vacuum. The residue was redissolved in a small amount of DCM and loaded in a silica gel column. Upon elution in DCM/EA (9:1), gave compound **S17** as an yellow oil (0.813 g, 3.1 mmol, 48 %).

Characterization data for S17:^{1}H NMR (500 MHz, $CDCl_3$) δ 7.93-7.91 (m, 2H), 6.96-6.94 (m, 2H), 4.21, 4.21, 4.21, 4.21-4.19 (m, 4H), 3.90-3.88 (m, 2H), 3.77-3.72 (m, 4H), 2.55 (s, 3H), 2.43 (t, J = 2.4 Hz, 1H).

^{13}C NMR (126 MHz, $CDCl_3$) δ 196.77, 162.66, 130.52, 130.42, 114.24, 79.52 74.61, 74.59, 70.67, 69.53, 69.09, 67.56, 58.43, 26.32.

Preparation of Compound 5c:

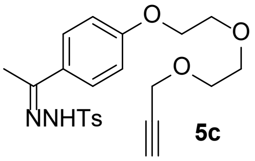

5c

S17 (813 mg, 3.1 mmol, 1.0 eq.) was dissolved in 120 mL of 1,2-dichloroethane, then $TsNHNH_2$ (3.5 g, 18.8 mmol, 6.0 eq.) was added. The resulting solution was stirred at 100 °C under N_2 atmosphere overnight and cooled down after TLC showed no further change. The solution was concentrated under vacuum and loaded onto a silica gel column. Eluting with DCM to DCM/EA (40 : 1), the unreacted $TsNHNH_2$ was removed partially and a mixture of compound **5c** and $TsNHNH_2$ with a rough ratio of 1:2.5 (based on ^{1}H NMR). Crude compound **5c** (530.9 mg) was isolated by the preparative TLC, which was directly used in next steps without further purification.

Characterization data for 5c:

There are some short peaks of impurities in ^{1}H NMR and ^{13}C NMR spectra of compound **5c.** Only the peaks of compound **5c** were shown below.

^{1}H NMR (500 MHz, $CDCl_3$) δ 8.21 (s, 1H), 7.91-7.89 (m, 2H), 7.53-7.51 (m, 2H), 7.28-7.27 (m, 2H), 6.80-6.77 (m, 2H), 4.17-4.16 (m, 2H), 4.08-4.05 (m, 2H), 3.83-3.80 (m, 2H), 3.73-3.69 (m, 4H), 2.42 (t, J = 2.4 Hz, 2H), 2.36 (s, 3H), 2.09 (s, 3H).

^{13}C NMR (126 MHz, $CDCl_3$) δ 159.70, 152.69, 143.82, 135.37, 129.87, 129.36, 127.94, 127.50, 114.04, 79.42, 74.57, 70.42, 69.47, 68.90, 67.19, 58.22, 21.41, 13.27.

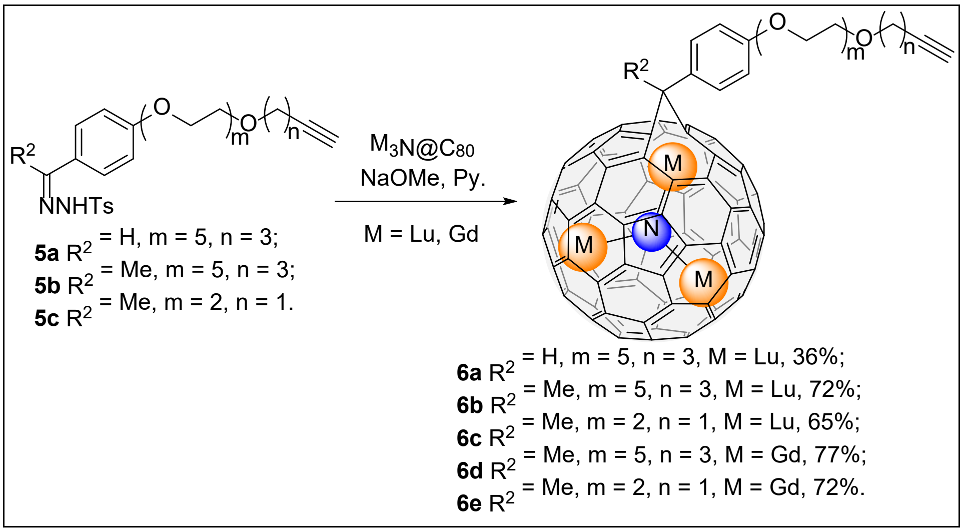

Scheme S4: Synthesis of EMF partners

Preparation of Compound 6a:

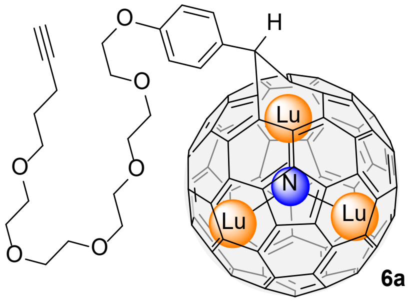

6a

5a (19.2 mg, 0.033 mmol, 5.0 eq.) and NaOMe (2.88 mg, 0.053 mmol, 8.0 eq.) were dissolved in 3 mL of pyridine under N_2 atmosphere. The solution was stirred at room temperature for 20 minutes. A solution of $Lu_3N@C_{80}$ (10 mg, 0.0067 mmol, 1.0 eq.) in 20 mL of *o*-dichlorobenzene was degassed by freeze-pump-thaw process and refilled with N_2, and then injected to the abovementioned pyridine solution. The resulting solution was stirred at 70 °C for 4 hours (the reaction was tracked by TLC and terminated when there was no further change). The solution was loaded onto a silica gel column eluting with hexanes and then CS_2 to remove *o*-dichlorobenzene and unreacted $Lu_3N@C_{80}$. Eluting with CS_2/EA (6 : 1) gave the brown band as compound **6a** (4.5 mg, 0.0024 mmol, 36%).

Characterization data for 6a:

^{1}H NMR (500 MHz, CS_2/CD_2Cl_2) δ 8.04-8.02 (m, 2H), 7.12-7.10 (m, 2H), 6.02 (s, 1H), 4.22-4.20 (m, 2H), 3.89-3.87 (m, 2H), 3.73-3.71 (m, 2H), 3.66-3.64 (m, 2H), 3.64-3.56 (m, 12H), 3.54 (t, J = 6.1 Hz, 2H), 2.28 (td, J = 7.1, 2.7 Hz, 2H), 1.94 (t, J = 2.6 Hz, 1H), 1.81-1.76 (m, 2H).

^{13}C NMR (126 MHz, CS_2/CD_2Cl_2) δ 159.60, 151.96, 151.87, 150.62, 150.50, 148.98, 148.95, 148.77, 148.59, 148.42, 148.22, 148.13, 147.33, 147.13, 146.42, 146.36, 145.63, 145.60, 145.56, 145.48, 145.33, 145.24, 145.18, 144.98, 144.91, 144.57, 144.21, 144.08, 144.05, 144.00, 143.64, 143.59, 143.36, 143.31, 143.15, 143.09, 143.05, 142.78, 142.69, 142.52, 142.42, 141.62, 141.12, 140.99, 140.94, 140.59, 140.55, 140.14, 139.72, 139.63, 139.56, 138.55, 138.51, 136.19, 136.02, 135.96, 135.90, 135.51, 135.48, 135.34, 135.12, 134.95, 129.89 (**C**H of benzene ring), 128.88, 128.52, 128.46, 127.28, 127.01, 126.80, 126.58, 126.15, 115.51 (**C**H of benzene ring), 93.21 (1C, bridge **C** of fullerene cage), 88.94 (1C, bridge **C** of fullerene cage), 84.60 (sp **C** of alkyne), 71.54, 71.32, 71.29, 71.27, 71.23, 70.98, 70.30, 70.08, 69.13 (sp **C** of alkyne), 68.29, 45.52 (1C, bridge carbon), 29.51, 15.92.

MALDI-TOF-Positive $C_{102}H_{32}Lu_3NO_6$ ($[M]^{\bullet+}$) m/z calculated 1891.0, found 1891.5.

Preparation of Compound 6b:

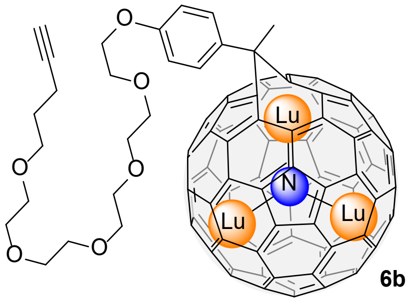

6b

5b (59 mg, 0.1 mmol, 5.0 eq.) and NaOMe (13 mg, 0.24 mmol, 12.0 eq.) were dissolved in 9 mL of pyridine under N_2 atmosphere. The solution was stirred at room temperature for 20 minutes. A solution of $Lu_3N@C_{80}$ (30 mg, 0.02 mmol, 1.0 eq.) in 60 mL of *o*-dichlorobenzene was degassed by freeze-pump-thaw process and refilled with N_2, and then injected to the abovementioned pyridine solution. The resulting solution was stirred at 70 °C for 4 hours (the reaction was tracked by TLC and terminated when there was no further change). The solution was loaded onto a silica gel column eluting with hexanes and then chloroform ($CHCl_3$) to remove *o*-dichlorobenzene and unreacted $Lu_3N@C_{80}$. Eluting with $CHCl_3$/EA (5 : 1) removed unreacted **5b**. Eluting with CS_2/EA (gradient increase from 6 : 1 to 2 : 1) gave the brown band as compound **6b** (27.5 mg, 0.014 mmol, 72%).

Characterization data for 6b:

^{1}H NMR (500 MHz, $CS_2/CDCl_2CDCl_2$) δ 8.03-8.01 (m, 2H), 7.08-7.07 (m, 2H), 4.23-4.21 (m, 2H), 3.93-3.91 (m, 2H),

3.77-3.75 (m, 2H), 3.71-3.60 (m, 14H), 3.58 (t, J = 6.1 Hz, 2H), 2.46 (s, 3H), 2.32 (td, J = 7.1, 2.7 Hz, 2H), 1.96 (t, J = 2.6 Hz, 1H), 1.85-1.80 (m, 2H).

^{13}C NMR (126 MHz, $CS_2/CDCl_2CDCl_2$) δ 157.98, 151.13, 150.85, 149.83, 149.64, 148.40, 147.76, 147.67, 147.64, 147.33, 147.22, 146.95, 146.75, 146.12, 145.49, 145.16, 144.80, 144.69, 144.59, 144.48, 144.35, 144.33, 144.02, 143.99, 143.82, 143.65, 143.46, 143.19, 143.13, 142.89, 142.74, 142.72, 142.33, 142.22, 142.20, 142.04, 141.86, 141.77, 141.72, 141.43, 141.33, 141.26, 140.80, 140.26, 140.12, 140.02, 139.48, 139.36, 139.12, 139.06, 138.70, 138.52, 137.57, 137.48, 136.61, 135.18, 134.98, 134.95, 134.79, 134.57, 134.24, 134.06, 134.02, 133.97, 128.55, 128.15, 127.41, 126.40, 126.37, 126.04, 125.50, 125.09, 124.81, 114.47, 98.25 (1C, bridge **C** of fullerene cage), 95.70 (1C, bridge **C** of fullerene cage), 83.85 (sp **C** of alkyne), 70.57, 70.32, 70.29, 70.25, 69.97, 69.85, 69.38 (sp **C** of alkyne), 69.15, 68.65, 67.23, 46.66 (1C, bridge carbon), 29.39 (1C, **C**H_3), 28.51, 15.12.

MALDI-TOF-Positive $C_{103}H_{34}Lu_3NO_6$ ($[M]^{\bullet+}$) m/z calculated 1905.1, found 1905.4.

Preparation of Compound 6c:

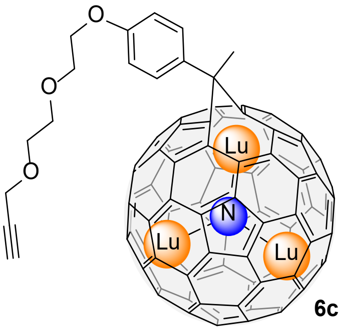

6c

5c (42.9 mg, 0.010 mmol, 5.0 eq.) and NaOMe (12.9 mg, 0.24 mmol, 12.0 eq.) were dissolved in 9 mL of pyridine under N_2 atmosphere. The solution was stirred at room temperature for 20 minutes. A solution of $Lu_3N@C_{80}$ (30 mg, 0.02 mmol, 1.0 eq.) in 60 mL of *o*-dichlorobenzene was degassed by freeze-pump-thaw process and refilled with N_2, and then injected to the abovementioned pyridine solution. The resulting solution was stirred at 70 ^{o}C for 5 hours (the reaction was tracked by TLC and terminated when there was no further change). The solution was loaded onto a silica gel column eluting with hexanes and then CS_2 to remove *o*-dichlorobenzene and unreacted $Lu_3N@C_{80}$. Eluting with CS_2/EA (40 : 1) removed unreacted **5c**. Eluting with CS_2/EA (20 : 1) gave the brown band as compound **6c** (22.5 mg, 0.013 mmol, 65%).

Characterization data for 6c:

^{1}H NMR (500 MHz, $CS_2/CDCl_2CDCl_2$) δ 8.03-8.00 (m, 2H), 7.09-7.06 (m, 2H), 4.24 (d, J = 2.4 Hz, 2H), 4.23-4.21 (m, 2H), 3.93-3.91 (m, 2H), 3.79-3.74 (m, 4H), 2.50 (t, J = 2.4 Hz, 1H), 2.44 (s, 3H).

^{13}C NMR (126 MHz, $CS_2/CDCl_2CDCl_2$) δ 157.97, 151.19, 150.93, 149.90, 149.72, 148.46, 147.85, 147.72, 147.69, 147.42, 147.29, 147.02, 146.84, 146.19, 145.56, 145.25, 144.87, 144.75, 144.68, 144.54, 144.41, 144.10, 144.08, 143.90, 143.72, 143.53, 143.24, 143.21, 142.94, 142.82, 142.80, 142.43, 142.40, 142.29, 142.24, 142.10, 141.93, 141.85, 141.80, 141.78, 141.51, 141.41, 141.35, 140.86, 140.33, 140.21, 140.10, 139.58, 139.46, 139.18, 138.80, 138.61, 137.69, 137.60, 136.80, 135.28, 135.08, 135.07, 134.91, 134.68, 134.63, 134.34, 134.16, 134.09, 128.66, 128.26, 127.51, 126.49, 126.45, 126.11, 125.59, 125.15, 114.55, 98.37 (1C, bridge **C** of fullerene cage), 95.80 (1C, bridge **C** of fullerene cage), 79.68 (sp **C** of alkyne), 74.63 (sp **C** of alkyne), 70.35, 69.46, 68.94, 67.30, 58.11, 46.75 (1C, bridge carbon), 29.47(1C, **C**H_3).

MALDI-TOF-Negative $C_{95}H_{18}Lu_3NO_3$ ($[M]^{\bullet-}$) m/z calculated 1745.0, found 1745.4.

Preparation of Compound 6d:

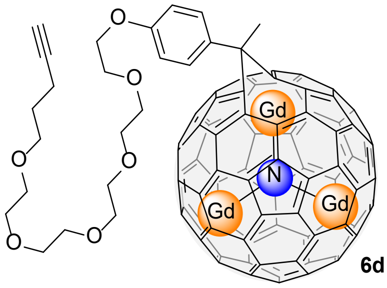

6d

5b (13.0 mg, 0.022 mmol, 4.0 eq.) and NaOMe (3.0 mg, 0.056 mmol, 10.0 eq.) were dissolved in 2.4 mL of pyridine under N_2 atmosphere. The solution was stirred at room temperature for 15 minutes. A solution of $Gd_3N@C_{80}$ (8.0 mg, 0.0055 mmol, 1.0 eq.) in 16 mL of *o*-dichlorobenzene was degassed by freeze-pump-thaw process and refilled with N_2, and then injected to the abovementioned pyridine solution. The resulting solution was stirred at 55 °C for 8 hours (the reaction was tracked by TLC and terminated when there was no further change). The solution was loaded onto a silica gel column eluting with hexanes and then $CHCl_3$ to remove *o*-dichlorobenzene and unreacted $Gd_3N@C_{80}$. Eluting with $CHCl_3$/EA (5 : 1) removed unreacted **5b**. Eluting with CS_2/EA (5 : 1 to 2 : 1) gave the brown band as compound **6d** (7.9 mg, 0.0043 mmol, 77%).

Characterization data for 6d:

MALDI-TOF-Positive $C_{103}H_{34}Gd_3NO_6$ ($[M]^{\bullet+}$) m/z calculated 1853.2, found 1853.0.

Preparation of Compound 6e:

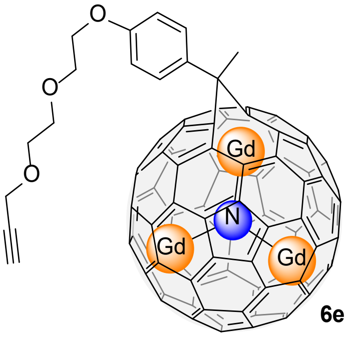

6e

5c (9.5 mg, 0.022 mmol, 4.0 eq.) and NaOMe (3.0 mg, 0.056 mmol, 10.0 eq.) were dissolved in 2.4 mL of pyridine under N_2 atmosphere. The solution was stirred at room temperature for 15 minutes. A solution of $Gd_3N@C_{80}$ (8.0 mg, 0.0055 mmol, 1.0 eq.) in 16 mL of *o*-dichlorobenzene was degassed by freeze-pump-thaw process and refilled with N_2, and then injected to the abovementioned pyridine solution. The resulting solution was stirred at 55 °C for 9 hours (the reaction was tracked by TLC and terminated when there was no further change). The solution was loaded onto a silica gel column eluting with hexanes and then CS_2 to remove *o*-dichlorobenzene and unreacted $Gd_3N@C_{80}$. Eluting with CS_2/EA (20 : 1) gave the brown band as compound **6e** (6.7 mg, 0.0040 mmol, 72%).

Characterization data for 6e:

MALDI-TOF-Positive $C_{95}H_{18}Gd_3NO_3$ ($[M]^{\bullet+}$) m/z calculated 1693.9, found 1693.8.

2.3 Synthesis of metallobuckytrios (MBTs)

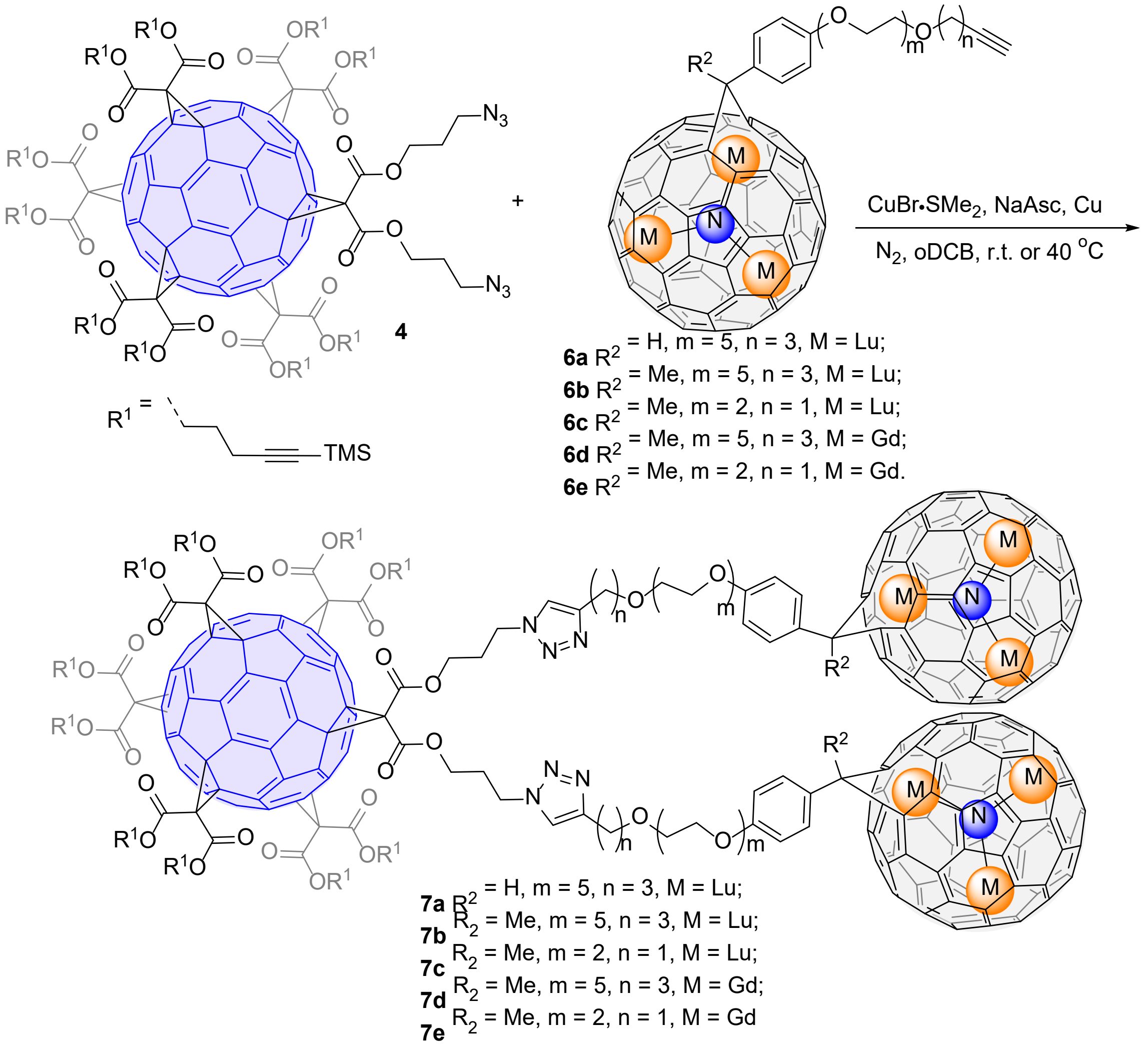

Scheme S5: Synthesis of basic MBT structures

Preparation of Compound 7a:

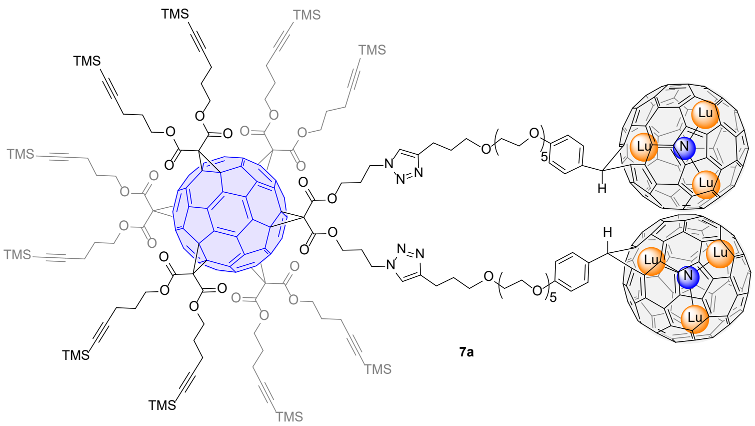

4 (10.7 mg, 0.0037 mmol, 1.0 eq.) and **6a** (21 mg, 0.011 mmol, 3.0 eq.) were dissolved in 9 mL of *o*-dichlorobenzene. $CuBr·SMe_2$ (4.6 mg, 0.022 mmol, 6.0 eq.), sodium L-ascorbate (NaAsc, 6.6 mg, 0.033 mmol, 9.0 eq.) and a piece of sanded copper metal wire were added to the solution. The resulting solution was degassed by freeze-pump-thaw process and refilled with N_2, and then stirred at room temperature for 3 days (the reaction was terminated when there was no further change). The solution was washed three times with saturated aqueous ethylenediaminetetraacetic acid (EDTA) solution and then three times by DI water. The solution was dried with anhydrous Na_2SO_4 and filtered, and concentrated under vacuum. The residue was loaded onto a silica gel column eluting with hexanes to remove *o*-dichlorobenzene. Eluting with CS_2/EA (2 : 1) gave the unreacted **6a** (9.2 mg, 0.0049 mmol). Eluting with $CHCl_3$/Methanol

(MeOH) (20 : 1) gave the brown band as compound **7a** (19.5 mg, 0.0029 mmol, 79%).

Characterization data for 7a:

^{1}H NMR (500 MHz, $CS_2/CDCl_2CDCl_2$) δ 8.04-8.02 (m, 4H), 7.43 (s, 2H), 7.13-7.11 (m, 4H), 6.02 (s, 2H), 4.46-4.37 (m, 28H), 4.24-4.22 (m, 4H), 3.92-3.90 (m, 4H), 3.76-3.74 (m, 4H), 3.70-3.58 (m, 28H), 3.53-3.50 (m, 4H), 2.80-2.77 (m, 4H), 2.39-2.33 (m, 24H), 1.97-1.94 (m, 24H), 0.21-0.17 (m, 90H).

^{13}C NMR (126 MHz, $CS_2/CDCl_2CDCl_2$) δ 163.05, 162.99, 162.93, 158.51, 151.01, 150.92, 149.68, 149.54, 148.03, 148.00, 147.84, 147.63, 147.43, 147.33, 147.29, 147.20, 146.36, 146.18, 145.58, 145.54, 145.47, 145.41, 145.34, 145.30, 144.67, 144.66, 144.60, 144.55, 144.51, 144.40, 144.29, 144.23, 144.03, 143.96, 143.63, 143.25, 143.12, 143.06, 142.70, 142.68, 142.68, 142.64, 142.42, 142.38, 142.22, 142.15, 142.11, 141.81, 141.72, 141.58, 141.47, 141.45, 140.66, 140.63, 140.58, 140.54, 140.35, 140.15, 140.05, 140.02, 139.64, 139.58, 139.18, 139.17, 138.77, 138.67, 138.60, 137.59, 137.54, 135.23, 135.06, 135.03, 134.93, 134.54, 134.51, 134.36, 134.17, 133.99, 128.98 (4C, **C**H of benzene ring), 127.92, 127.54, 127.50, 126.32, 126.06, 125.84, 125.59, 125.19, 120.76 (2C, **C** of 1,2,3-triazole ring), 114.71 (4C, **C**H of benzene ring), 104.77, 104.75, 104.72, 92.18, 87.94, 85.65, 70.55, 70.29, 70.25, 69.97, 69.85, 69.37, 68.74, 68.71, 68.65, 68.55, 67.30, 65.54, 63.47, 46.23, 44.93, 44.88, 44.59 (2C, bridge **C**), 29.16, 27.33, 22.06, 16.38, -0.05.

MALDI-TOF-Positive $C_{368}H_{227}Lu_6N_8O_{36}Si_{10}$ ($[M + H]^+$) m/z calculated 6662.0, found 6661.8.

Preparation of Compound 7b:

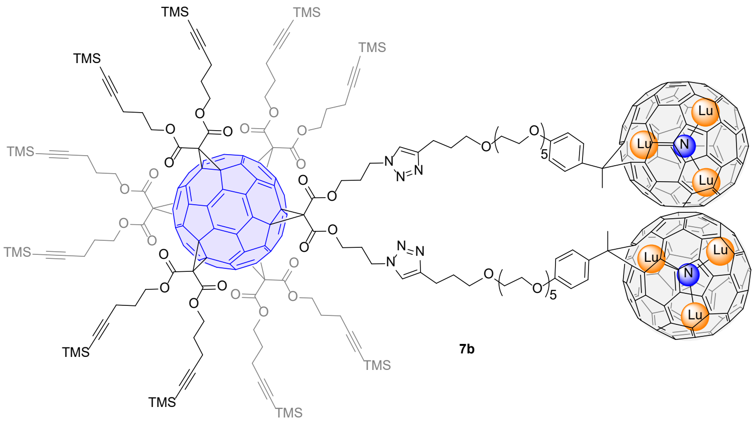

4 (25.0 mg, 0.0087 mmol, 1.0 eq.) and **6b** (49.6 mg, 0.026 mmol, 3.0 eq.) were dissolved in 9 mL of *o*-dichlorobenzene. $CuBr·SMe_2$ (10.7 mg, 0.052 mmol, 6.0 eq.), NaAsc (15.5 mg, 0.078 mmol, 9.0 eq.) and a piece of sanded copper metal wire were added to the solution. The resulting solution was degassed by a freeze-pump-thaw process, refilled with N_2, and then stirred at 40 °C for 4 days (the reaction was terminated when there was no further change). The solution was washed three times by saturated aqueous EDTA solution and then three times by DI water. The solution was dried by anhydrous Na_2SO_4 and filtered, concentrated under vacuum. The residue was loaded onto a silica gel column eluting with hexanes to remove *o*-dichlorobenzene. Eluting with CS_2/EA (2 : 1) gave the unreacted **6b** (24.4 mg, 0.013 mmol). Eluting with $CHCl_3$/MeOH (20 : 1) gave the brown band as compound **7b** (43.1 mg, 0.0064 mmol, 74%).

Characterization data for 7b:

^{1}H NMR (500 MHz, $CS_2/CDCl_2CDCl_2$) δ 8.03-8.00 (m, 4H), 7.43 (s, 2H), 7.08-7.06 (m, 4H), 4.48-4.32 (m, 28H), 4.22-4.20 (m, 4H), 3.91-3.89 (m, 4H), 3.75-3.74 (m, 4H), 3.70-3.58 (m, 28H), 3.52-3.50 (m, 4H), 2.80-2.77 (m, 4H), 2.44 (s, 6H), 2.36-2.33 (m, 24H), 1.97-1.94 (m, 24H), 0.20-0.17 (m, 90H).

^{13}C NMR (126 MHz, $CS_2/CDCl_2CDCl_2$) δ 163.04, 162.98, 162.92, 157.97, 151.15, 150.88, 149.86, 149.67, 148.42, 147.79, 147.68, 147.65, 147.37, 147.32, 147.25, 146.97, 146.78, 146.15, 145.57, 145.53, 145.40, 145.33, 145.29, 145.20, 144.83, 144.72, 144.63, 144.51, 144.37, 144.06, 144.03, 143.85, 143.67, 143.49, 143.20, 143.17, 142.91, 142.78, 142.76, 142.36, 142.24, 142.21, 142.07, 141.89, 141.81, 141.75, 141.74, 141.46, 141.37, 141.30, 140.83, 140.64, 140.57, 140.53, 140.35, 140.29, 140.16, 140.05, 139.52, 139.40, 139.17, 139.11, 138.74, 138.55, 137.62, 137.53, 136.67, 135.22, 135.01, 135.01, 134.84, 134.62, 134.59, 134.28, 134.10, 134.06, 134.02, 128.60 (4C, **C**H of benzene ring), 128.19, 127.46, 126.44, 126.41, 126.07, 125.54, 125.11, 124.84, 120.76 (2C, **C** of 1,2,3-triazole ring), 114.49 (4C, **C**H of benzene ring), 104.76, 104.74, 104.72, 98.31, 95.76, 85.65, 85.62, 70.53, 70.28, 70.25, 70.23, 69.96,

69.84, 69.37, 68.73, 68.70, 68.64, 68.54, 67.24, 65.53, 63.46, 46.70, 46.23, 44.92, 44.88, 29.43 (2C, **C**H_3), 29.15, 27.32, 22.04, 16.37, -0.05.

MALDI-TOF-Positive $C_{370}H_{231}Lu_6N_8O_{36}Si_{10}$ ($[M]^{\bullet+}$) m/z calculated 6689.1, found 6689.1.

Preparation of Compound 7c:

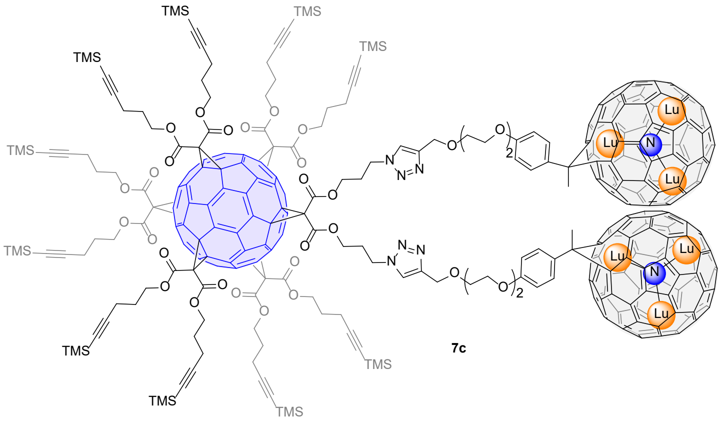

7c

4 (11.0 mg, 0.0038 mmol, 1.0 eq.) and **6c** (20.0 mg, 0.011 mmol, 3.0 eq.) were dissolved in 9 mL of *o*-dichlorobenzene. CuBr·SMe_2 (4.7 mg, 0.023 mmol, 6.0 eq.), NaAsc (6.8 mg, 0.034 mmol, 9.0 eq.) and a piece of sanded copper metal wire were added to the solution. The resulting solution was degassed by freeze-pump-thaw process, refilled with N_2, and then stirred at 40 °C for 3 days (the reaction was terminated when there was no further change). The solution was washed three times by saturated aqueous EDTA solution and then three times by DI water. The solution was dried by anhydrous Na_2SO_4 and filtered, concentrated under vacuum. The residue was loaded onto a silica gel column eluting with hexanes to remove *o*-dichlorobenzene. Eluting with CS_2/EA (20 : 1) gave the unreacted **6c** (6.2 mg, 0.0036 mmol). Eluting with $CHCl_3$/MeOH (100 : 1) gave the brown band as compound **7c** (23.3 mg, 0.0037 mmol, 93%).

Characterization data for 7c:

^{1}H NMR (500 MHz, $CS_2/CDCl_2CDCl_2$) δ 8.01 (d, J = 8.3 Hz, 4H), 7.68 (s, 2H), 7.05 (d, J = 8.8 Hz, 4H), 4.66-4.65 (m, 4H), 4.50-4.40 (m, 28H), 4.21-4.19 (m, 4H), 3.90-3.88 (m, 4H), 3.75-3.72 (m, 8H), 2.44 (s, 6H), 2.36-2.34 (m, 20H), 1.97-1.94 (m, 24H), 0.22-0.17 (m, 90H).

^{13}C NMR (126 MHz, $CS_2/CDCl_2CDCl_2$) δ 163.03, 162.94, 162.91, 162.87, 157.92, 151.13, 150.86, 149.85, 149.65, 148.41, 147.78, 147.66, 147.65, 147.36, 147.24, 146.95, 146.76, 146.13, 145.57, 145.52, 145.51, 145.39, 145.31, 145.27, 145.19, 144.82, 144.71, 144.66, 144.61, 144.50, 144.36, 144.04, 143.84, 143.65, 143.49, 143.19, 143.17, 142.89, 142.75, 142.35, 142.22, 142.06, 141.89, 141.86, 141.80, 141.75, 141.74, 141.45, 141.36, 141.29, 140.80, 140.64, 140.55, 140.52, 140.28, 140.27, 140.14, 140.02, 139.49, 139.38, 139.15, 139.08, 138.72, 138.53, 137.59, 137.51, 136.71, 135.20, 134.99, 134.82, 134.60, 134.57, 134.25, 134.09, 134.07, 134.00, 128.60 (4C, **C**H of benzene ring), 128.16, 127.44, 126.42, 126.39, 126.05, 125.51, 125.08, 124.82, 122.57 (2C, **C** of 1,2,3-triazole ring), 114.47 (4C, **C**H of benzene ring), 104.72, 98.27, 95.72, 85.65, 70.45, 69.45, 69.38, 68.71, 68.69, 68.63, 68.50, 67.23, 65.51, 64.07, 63.29, 46.69, 46.41, 44.91, 44.87, 29.41 (2C, **C**H_3), 29.08, 27.33, 16.38.

MALDI-TOF-Positive $C_{354}H_{199}Lu_6N_8O_{30}Si_{10}$ ($[M + H]^+$) m/z calculated 6369.8, found 6370.0.

Preparation of Compound 7d:

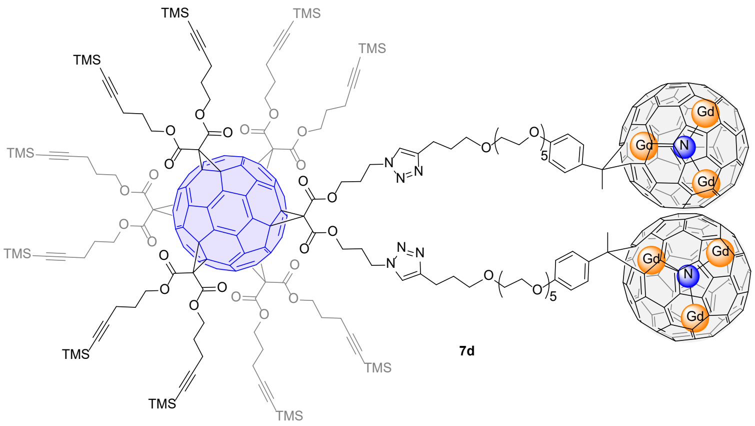

4 (5.9 mg, 0.0020 mmol, 1.0 eq.) and **6d** (11.3 mg, 0.0061 mmol, 3.0 eq.) were dissolved in 4.5 mL of *o*-dichlorobenzene. CuBr·SMe_2 (2.5 mg, 0.012 mmol, 6.0 eq.), NaAsc (3.6 mg, 0.018 mmol, 9.0 eq.) and a piece of sanded copper metal wire were added to the solution. The resulting solution was degassed by freeze-pump-thaw process and refilled with N_2, and then stirred at 40 °C for 4 days (the reaction was terminated when there was no further change). The solution was washed three times by saturated aqueous EDTA solution and then three times by DI water. The solution was dried by anhydrous Na_2SO_4 and filtered, concentrated under vacuum. The residue was loaded onto a silica gel column eluting with hexanes to remove *o*-dichlorobenzene. Eluting with CS_2/EA (2 : 1) gave the unreacted **6d** (6.5 mg, 0.0035 mmol). Eluting with $CHCl_3$/MeOH (20 : 1) gave the brown band as compound **7d** (8.0 mg, 0.0012 mmol, 59%).

Characterization data for 7d:

MALDI-TOF-Positive $C_{370}H_{230}Gd_6N_8O_{36}Si_{10}$ ($[M]^{\bullet+}$) m/z calculated 6587.0, found 6586.9.

Preparation of Compound 7e:

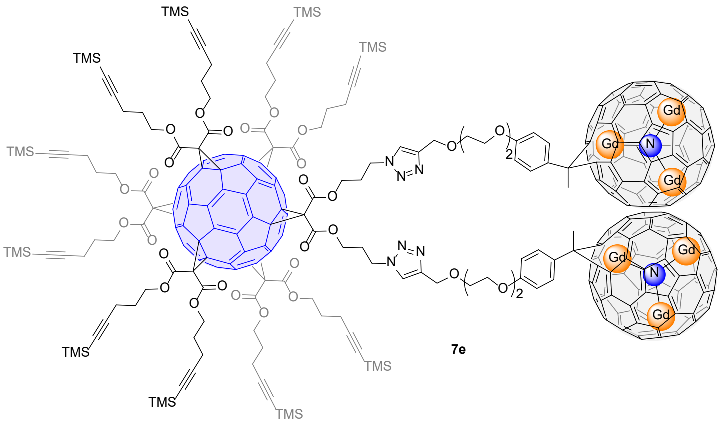

4 (9.0 mg, 0.0031 mmol, 1.0 eq.) and **6e** (15.8 mg, 0.0093 mmol, 3.0 eq.) were dissolved in 7.0 mL of *o*-dichlorobenzene. CuBr·SMe_2 (3.8 mg, 0.018 mmol, 6.0 eq.), NaAsc (5.5 mg, 0.028 mmol, 9.0 eq.) and a piece of sanded copper metal wire were added to the solution. The resulting solution was degassed by freeze-pump-thaw process and refilled with N_2, and then stirred at 40 °C for 6 days (the reaction was terminated when there was no further change). The solution was washed three times by saturated aqueous EDTA solution and then three times by DI water. The solution was dried by anhydrous Na_2SO_4 and filtered, concentrated under vacuum. The residue was loaded onto a silica gel column eluting with hexanes and CS_2 to remove *o*-dichlorobenzene. Eluting with CS_2/EA (20 : 1) gave the unreacted **6e** (5.2 mg, 0.0031 mmol). Eluting with $CHCl_3$/MeOH (75 : 1) gave the brown band as compound **7e** (11.6 mg, 0.0019 mmol, 59%).

Characterization data for 7e:

MALDI-TOF-Positive $C_{354}H_{198}Gd_6N_8O_{30}Si_{10}$ ($[M]^{\bullet+}$) m/z calculated 6266.7, found 6266.7.

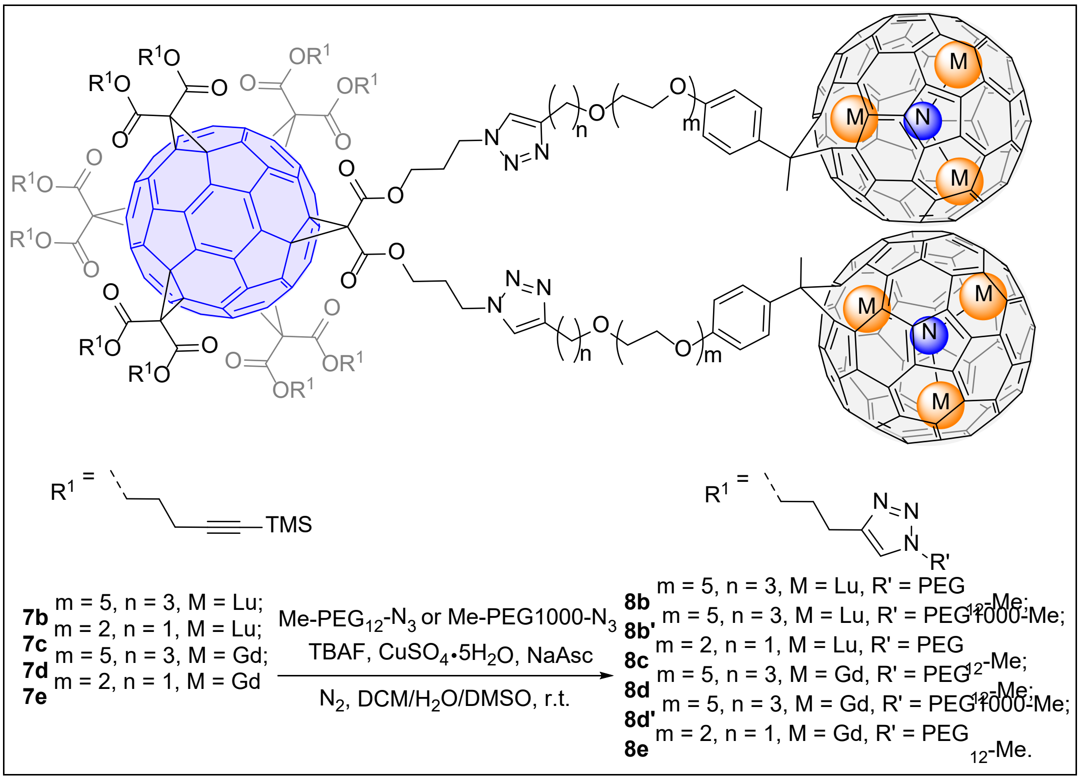

Scheme S6: Synthesis of water-soluble molecules

Preparation of Me-PEG1000-OTs:

To **PEG-1000 monomethyl ether** (5 g, ~5 mmol, 1.0 eq.) in 50 mL anhydrous CH_2Cl_2, was added p-toluenesulfonyl chloride (9.5 g, 50 mmol, 10.0 eq.). The solution was cooled to 0 °C in an ice-bath, followed by the addition of Et_3N (5.1 g, 50 mmol, 10.0 eq.) dropwise. After addition, the ice-bath was removed and the solution was stirred at room temperature for 24 hrs. Then, the reaction mixture was concentrated and directly loaded in a silica gel column. Upon elution in $CHCl_3$/MeOH (24:1), **Me-PEG1000-OTs** was obtained as a light yellow solid (2.57 g, ~2.2 mmol, 44%).

Characterization data for Me-PEG1000-OTs:

^{1}H NMR (500 MHz, $CDCl_3$) δ 7.81-7.79 (m, 2H), 7.35-7.33 (m, 2H), 4.17-4.15 (m, 2H), 3.69-3.60 (m, 80H), 3.58 (m, 4H), 3.56-3.54 (m, 2H), 3.38 (s, 3H), 2.45 (s, 3H).

^{13}C NMR (126 MHz, $CDCl_3$) δ 144.74, 132.98, 129.78, 127.94, 71.89, 70.70, 70.56, 70.52, 70.47, 69.19, 68.64, 59.00, 21.61.

Preparation of Me-PEG1000-N$_3$:

To **Me-PEG1000-OTs** (1.20 g, ~1.04 mmol, 1.0 eq.) in 5 mL CH_3CN, NaN_3 (0.68 g, 10.46 mmol, 10.0 eq.) was added and the reaction mixture was heated to 80 °C and stirred for 24 hrs until TLC showed no **Me-PEG1000-OTs** was left. Reaction mixture was filtered and concentrated. The residue was diluted in a small amount $CHCl_3$ and loaded in a silica gel column. Upon elution using $CHCl_3$/MeOH (24:1), **Me-PEG1000-N$_3$** was obtained and after concentration under vacuum gave a light yellow solid (0.600 g, ~0.60 mmol, 58%).

Characterization data for Me-PEG1000-N$_3$:

^{1}H NMR (500 MHz, $CDCl_3$) δ 3.78-3.58 (m, 84H), 3.55-3.53 (m, 2H), 3.39-3.37 (m and s, 5H), 1.87-1.85 (t, 2H).

^{13}C NMR (126 MHz, $CDCl_3$) δ 71.89, 70.66, 70.63, 70.59, 70.56, 70.53, 70.48, 69.99, 58.99, 50.64.

Preparation of Compound 8b:

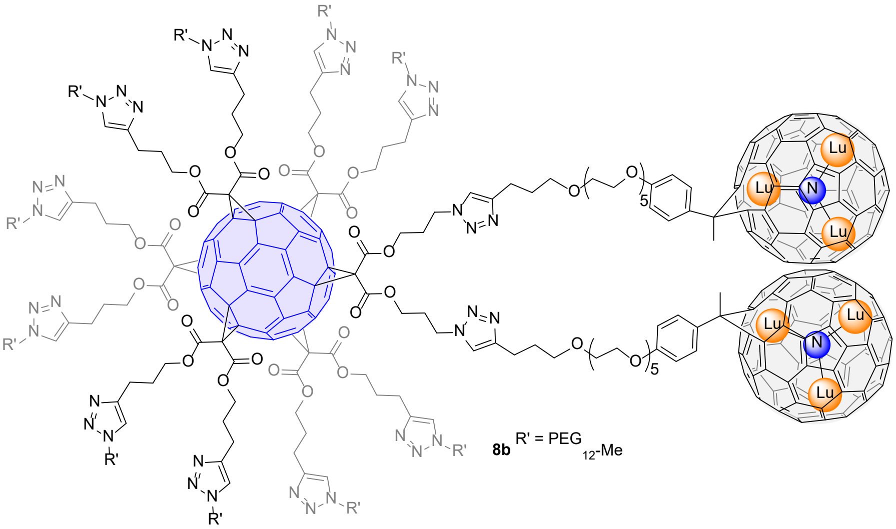

7b (10.0 mg, 0.0015 mmol, 1.0 eq.) and Me-PEG$_{12}$-N$_3$ (43.8 mg, 0.075 mmol, 50.0 eq.) were dissolved in 3 mL of DCM. Tetra-*n*-butylammonium fluoride (TBAF, 1 M solution in THF, 17.9 µL, 0.018 mmol, 12.0 eq.), a solution of $CuSO_4 \cdot 5H_2O$ (11.2 mg, 0.045 mmol, 30.0 eq.) and NaAsc (26.7 mg, 0.135 mmol, 90.0 eq.) in 2.25 mL of DI water, and 1.8 mL of DMSO were added sequentially to the above solution. The resulting solution was degassed by freeze-pump-thaw process and refilled with N_2, and then stirred at room temperature for 14 days. After that, it was kept in the hood without stirring for 7-10 days until all the suspended solids fully dissolved in the aqueous solution. The reaction was subsequently stirred for another 7 days to ensure complete reaction. The solution was loaded into a dialysis membrane bag (MWCO = 3.5 kD) and dialyzed in water for 72 h (water was changed every 12 hours) and lyophilized overnight to get a black solid as compound **8b** (17.0 mg, 0.0014 mmol, 96%).

Characterization data for 8b:

The structure of compound **8b** is complicated and the molecule is big, so no common characterization methods can be used for **8b**. The HNMR and CNMR were obtained. Only recognizable signals are shown below.

^{1}H NMR (500 MHz, $CDCl_3$) δ 7.94 (m, C**H** of benzene, 4H), 7.56-7.51 (m, C**H** of 1,2,3-triazole ring, 12H), 7.02 (m, C**H** of benzene, 4H), 4.49, 4.49, 4.46, 4.33, 4.17, 3.86, 3.85, 3.83, 3.64, 3.63, 3.62, 3.36 (m, terminal C**H$_3$**), 2.75, 2.75, 2.36, 2.08, 2.08, 2.02, 2.00, 1.99, 1.98, 1.94, 1.91.

^{13}C NMR (201 MHz, $CDCl_3$) δ 163.65, 158.34, 151.59, 151.21, 150.25, 150.16, 149.97, 148.81, 148.15, 147.96, 147.72, 147.58, 147.32, 147.13, 146.45, 146.22, 145.83, 145.72, 145.53, 145.13, 145.00, 144.80, 144.68, 144.36, 144.18, 144.00, 143.78, 143.49, 143.19, 143.08, 142.71, 142.57, 142.47, 142.38, 142.23, 142.10, 141.78, 141.69, 141.11, 140.59, 140.51, 140.38, 139.94, 139.79, 139.59, 139.17, 138.94, 138.04, 137.97, 137.09, 135.62, 135.43, 135.27, 135.01, 134.89, 134.68, 134.48, 128.93, 128.60, 127.83, 126.78, 126.32, 125.89, 125.40, 125.16, 122.14, 114.71, 98.72, 96.09, 77.16, 77.00, 76.84, 71.89, 70.52, 69.49, 67.48, 66.36, 58.99, 49.97, 47.03, 29.39, 28.07, 22.00, 14.06.

Preparation of Compound 8b′:

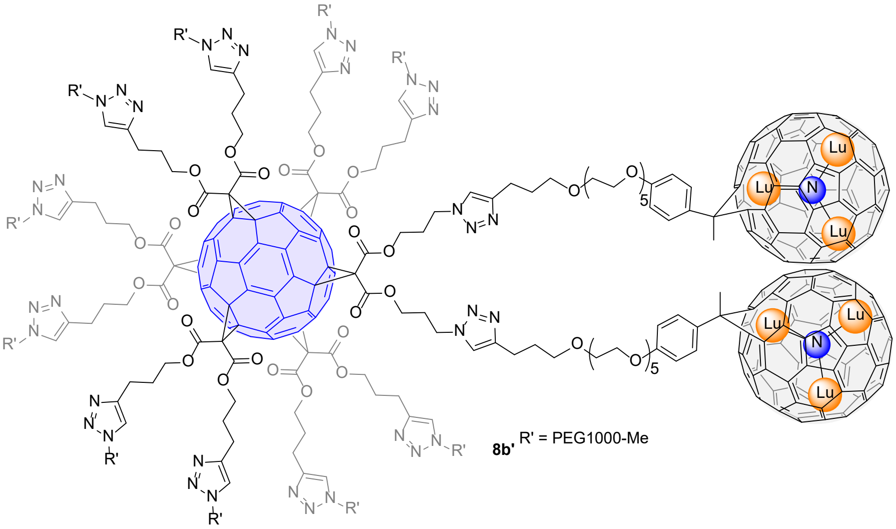

7b (10.0 mg, 0.0015 mmol, 1.0 eq.) and Me-PEG1000-N_3 (75.1 mg, 0.075 mmol, 50.0 eq.) were dissolved in 3 mL of DCM. Tetra-*n*-butylammonium fluoride (TBAF, 1 M solution in THF, 17.9 μL, 0.018 mmol, 12.0 eq.), a solution of $CuSO_4 \cdot 5H_2O$ (11.2 mg, 0.045 mmol, 30.0 eq.) and NaAsc (26.7 mg, 0.135 mmol, 90.0 eq.) in 2.25 mL of DI water, and 1.8 mL of DMSO were added sequentially to the above solution. The resulting solution was degassed by freeze-pump-thaw process and refilled with N_2, and then stirred at room temperature for 14 days. After that, it was kept in the hood without stirring for 7-10 days until all the suspended solids fully dissolved in the aqueous solution. The reaction was subsequently stirred for another 7 days to ensure complete reaction. The solution was loaded into a dialysis membrane bag (MWCO = 3.5 kD) and dialyzed in water for 72 h (water was changed every 12 hours) and lyophilized overnight to get a black solid as compound **8b′** (33.1 mg). The exact yield cannot be calculated due to inexact molecular mass. If assuming the M.W. of Me-PEG1000-N_3 =1000, yield is over 100%.

Characterization data for 8b′:

Similar as compound **8b**, no common characterization methods can be used for **8b′**. The HNMR was obtained. Only recognizable signals are shown below.

^{1}H NMR (500 MHz, $CDCl_3$) δ 7.96-7.95 (m, C**H** of benzene, 4H), 7.52 (m, C**H** of 1,2,3-triazole ring, 12H), 7.03 (m, C**H** of benzene, 4H), 4.50, 4.49, 4.49, 4.47, 4.42, 4.40, 4.39, 4.33, 4.33, 4.30, 4.27, 4.22, 4.21, 4.20, 4.19, 4.18, 4.17, 4.15, 3.88, 3.87, 3.85, 3.84, 3.83, 3.81, 3.80, 3.79, 3.78, 3.77 , 3.76, 3.75, 3.73, 3.72, 3.72, 3.71, 3.68, 3.67, 3.65, 3.65, 3.64, 3.63, 3.62, 3.60, 3.59, 3.55, 3.54, 3.54, 3.53, 3.51, 3.50, 3.49, 3.48, 3.45, 3.40, 3.39, 3.38, 3.37 (m, terminal C**H$_3$**), 3.22, 2.80, 2.78, 2.76, 2.75, 2.73, 2.36, 2.36, 2.34, 2.31, 2.15, 2.10, 2.08, 2.07, 2.05, 2.04, 2.02, 2.01.

Preparation of Compound 8c:

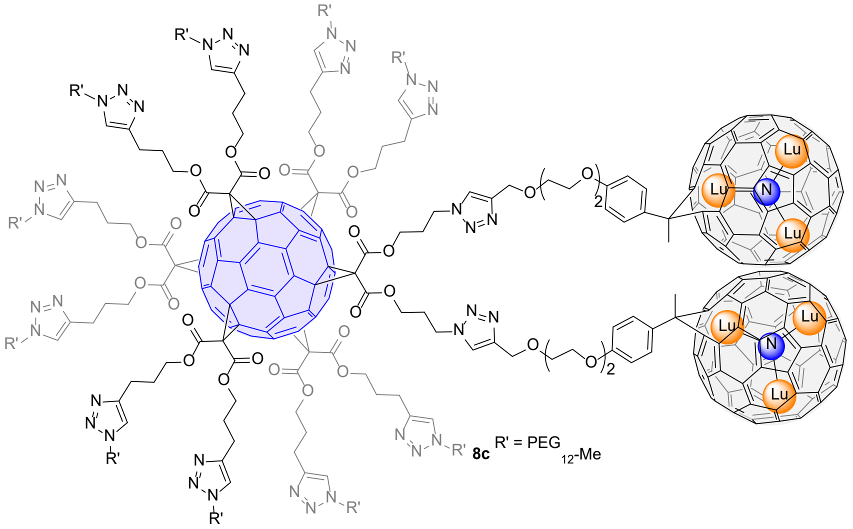

7c (10.0 mg, 0.0016 mmol, 1.0 eq.) and Me-PEG$_{12}$-N_3 (46.0 mg, 0.079 mmol, 50.0 eq.) were dissolved in 3 mL of DCM. Tetra-*n*-butylammonium fluoride (TBAF, 1 M solution in THF, 18.8 μL, 0.019 mmol, 12.0 eq.), a solution of $CuSO_4 \cdot 5H_2O$ (11.8 mg, 0.047 mmol, 30.0 eq.) and NaAsc (28.0 mg, 0.141 mmol, 90.0 eq.) in 2.25 mL of DI water, and 1.8 mL of DMSO were added sequentially to the above solution. The resulting solution was degassed by freeze-pump-thaw process and refilled with N_2, and then stirred at room temperature for 14 days. After that, it was kept in the hood without stirring for 7-10 days until all the suspended solids fully dissolved in the aqueous solution. The reaction was subsequently stirred for another 7 days to ensure complete reaction. The solution was loaded into a dialysis membrane bag (MWCO = 3.5 kD) and dialyzed in water for 72 h (water was changed every 12 hours) and lyophilized overnight to get a black solid as compound **8c** (18.6 mg, 0.0016 mmol, 100%).

Characterization data for 8c:

Similar as compound **8b** and **8b′**, no common characterization methods can be used for **8c**. The HNMR and MALDI-TOF MS were obtained. Only recognizable signals are shown below.

^{1}H NMR (500 MHz, $CDCl_3$) *δ* 7.98-7.91 (m, C**H** of benzene, 4H), 7.70-7.47 (m, C**H** of 1,2,3-triazole ring, 12H), 7.05-7.00 (m, C**H** of benzene, 4H), 4.66, 4.50, 4.49, 4.48, 4.47, 4.45, 4.43, 4.34, 4.32, 4.27, 4.26, 4.22, 4.20, 4.19, 4.17, 4.16, 4.16, 3.90, 3.88, 3.87, 3.86, 3.85, 3.85, 3.84, 3.78, 3.77, 3.76, 3.72, 3.72, 3.72, 3.71, 3.70, 3.70, 3.67, 3.65, 3.64,

3.63, 3.63, 3.63, 3.61, 3.61, 3.60, 3.55, 3.54, 3.54, 3.53, 3.51, 3.50, 3.49, 3.49, 3.37 (m, terminal CH_3), 3.23, 2.76, 2.76, 2.74, 2.37, 2.37, 2.36, 2.35, 2.32, 2.31, 2.29, 2.17, 2.13, 2.10, 2.09, 2.08, 2.04.
MALDI-TOF-Positive $C_{574}H_{628}Lu_6N_{38}O_{150}$ ($[M]^{\bullet+}$) m/z calculated 11501.9, found 11502.1.

Preparation of Compound 8d:

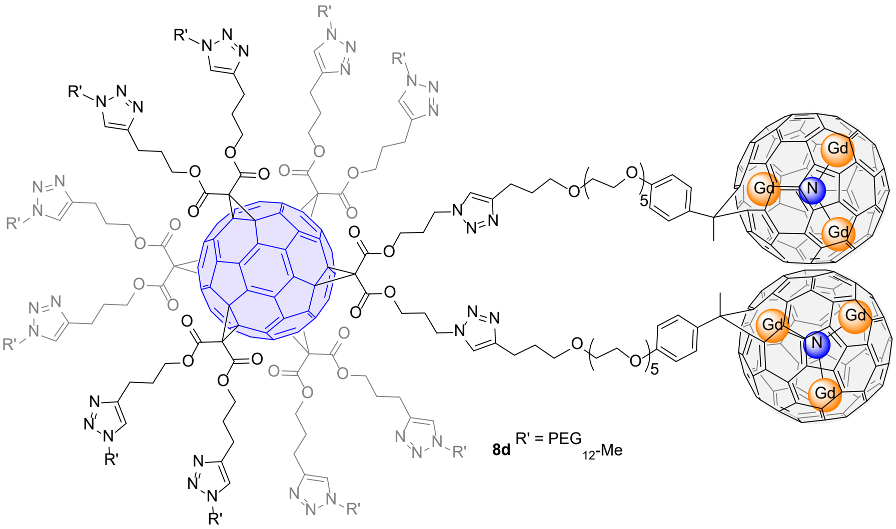

7d (4.0 mg, 0.00061 mmol, 1.0 eq.) and Me-PEG_{12}-N_3 (43.8 mg, 0.075 mmol, 123.0 eq.) were dissolved in 3 mL of DCM. Tetra-*n*-butylammonium fluoride (TBAF, 1 M solution in THF, 7.3 μL, 0.0073 mmol, 12.0 eq.), a solution of $CuSO_4\cdot5H_2O$ (11.2 mg, 0.045 mmol, 74.0 eq.) and NaAsc (26.7 mg, 0.135 mmol, 222.0 eq.) in 2.25 mL of DI water, and 1.8 mL of DMSO were added sequentially to the above solution. The resulting solution was degassed by freeze-pump-thaw process and refilled with N_2, and then stirred at room temperature for 14 days. After that, it was kept in the hood without stirring for 7-10 days until all the suspended solids fully dissolved in the aqueous solution. The reaction was subsequently stirred for another 7 days to ensure complete reaction. The solution was loaded into a dialysis membrane bag (MWCO = 3.5 kD) and dialyzed in water for 72 h (water is changed every 12 hours) to get an aqueous solution of compound **8d**. Due to the limited amount **8d** was not lyophilized. The yield was estimated to be quantitative based on our observation (UV-vis, ICP, etc.).

Preparation of Compound 8d′:

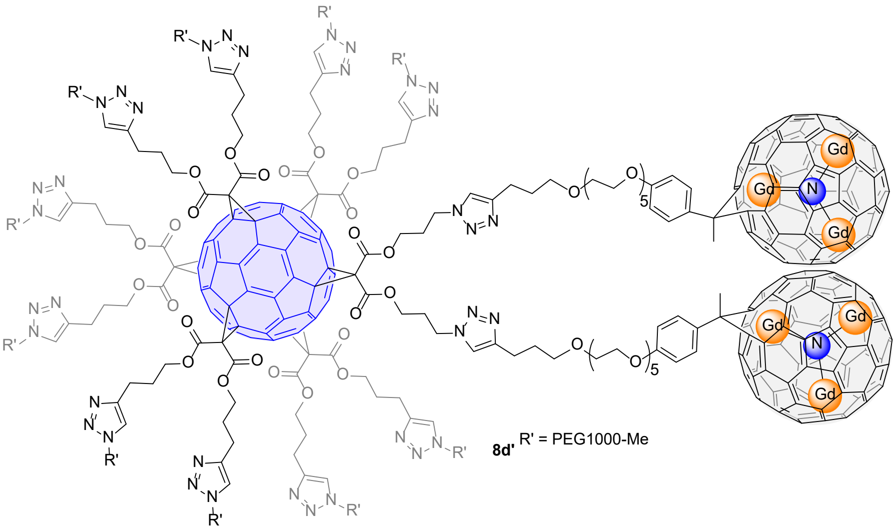

7d (4.5 mg, 0.00068 mmol, 1.0 eq.) and Me-PEG1000-N_3 (75.1 mg, 0.075 mmol, 110.0 eq.) were dissolved in 2 mL of DCM. Tetra-*n*-butylammonium fluoride (TBAF, 1 M solution in THF, 8.2 μL, 0.0082 mmol, 12.0 eq.), a solution of $CuSO_4\cdot5H_2O$ (11.2 mg, 0.045 mmol, 66.0 eq.) and NaAsc (26.7 mg, 0.135 mmol, 197.0 eq.) in 1.5 mL of DI water, and 1.2 mL of DMSO were added sequentially to the above solution. The resulting solution was degassed by freeze-pump-thaw process and refilled with N_2, and then stirred at room temperature for 14 days. After that, it was kept in the hood without stirring for 7-10 days until all the suspended solids fully dissolved in the aqueous solution. The reaction was

subsequently stirred for another 7 days to ensure complete reaction. The solution was loaded into a dialysis membrane bag (MWCO = 3.5 kD) and dialyzed in water for 72 h (water was changed every 12 hours) to get an aqueous solution of compound **8d'**. Due to the limited amount **8d'** was not lyophilized. The yield was estimated to be quantitative based on our observation (UV-vis, ICP, etc.).

Preparation of Compound 8e:

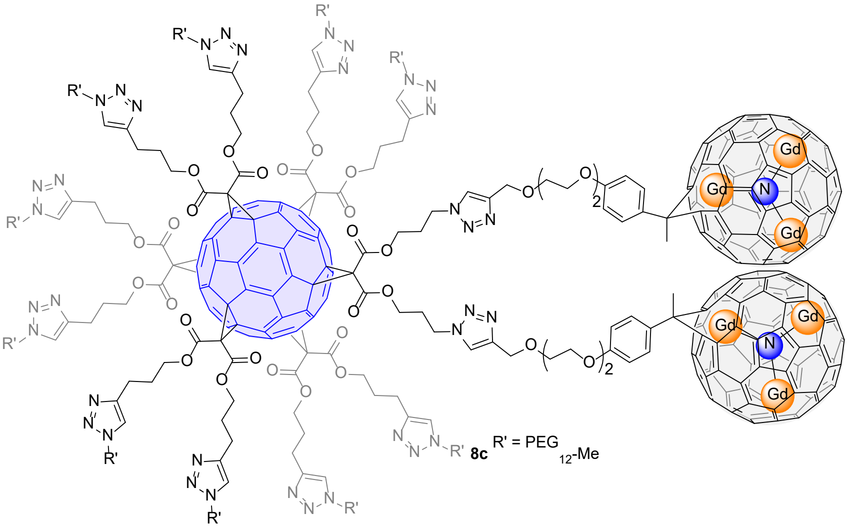

7e (5.6 mg, 0.00089 mmol, 1.0 eq.) and Me-PEG$_{12}$-N$_3$ (46.0 mg, 0.079 mmol, 88.0 eq.) were dissolved in 2.0 mL of DCM. Tetra-*n*-butylammonium fluoride (TBAF, 1 M solution in THF, 10.7 μL, 0.011 mmol, 12.0 eq.), a solution of $CuSO_4 \cdot 5H_2O$ (11.8 mg, 0.047 mmol, 53.0 eq.) and NaAsc (28.0 mg, 0.141 mmol, 158.0 eq.) in 1.5 mL of DI water and 1.2 mL of DMSO were added sequentially to the above solution. The resulting solution was degassed by freeze-pump-thaw process and refilled with N_2, and then stirred at room temperature for 14 days. After that, it was kept in the hood without stirring for 5 days until all the suspended solids fully dissolved in the aqueous solution. The reaction was subsequently stirred for another 7 days to ensure complete reaction. The solution was loaded into a dialysis membrane bag (MWCO = 3.5 kD) and dialyzed in water for 72 h (water is changed every 12 hours) to get an aqueous solution of compound **8e**. Due to the limited amount **8e** was not lyophilized. The yield was estimated to be quantitative based on our observation (UV-vis, ICP, etc.).

Characterization data for 8e:

MALDI-TOF-Positive $C_{574}H_{628}Gd_6N_{38}O_{150}$ ($[M]^{\bullet+}$) m/z calculated 11399.8, found 11399.5.

3. Supplemental procedures for experiments including:

STEM imaging

STEM Imaging was performed using a Nion UltraSTEM 100 with an aberration corrector and at an operating voltage of 60 kV. The imaging work was done using an electron probe with a convergence semi-angle of 35 mrad. This probe produces about 1-Å resolution with a non-monochromated beam. The transmitted high-angle scattered electrons were collected using a high-angle annular detector with inner and outer collection angles of 80 mrad and 200 mrad, respectively. Electron beam current for the image acquisitions were 15–25 pA. Additionally, both the high angle annular dark field (HAADF) and bright field (BF) were in combination to assist in increasing the visibility of the EMFs. Sample **8e** was dropcasted from water onto a PELCO® Single Layer Graphene TEM Support Film on Lacey Carbon, 300 Mesh Copper Grid from Ted Pella. Two to three drops of sample were placed on the TEM grid and left to air dry for two hours. Sample is then baked for overnight and transferred to the Nion UltraSTEM 100 for imaging.

DLS measurements:

Dynamic light scattering experiments were performed using a Malvern Zetasizer Nano ZS90 instrument with a standard laser source (4mW, 633nm), to measure the hydrodynamic size of the aggregates formed by the samples in solution. 1 uM sample concentrations were used for all the samples measured.

ICP-MS

Samples were digested for 2 hours at 85 °C in 70% ACS grade nitric acid and 30% hydrogen peroxide in a volumetric ratio of 1:2:1, then they were diluted to 2% concentration using 18 MΩ water in appropriate volumetric flasks. Along with the samples, several calibration standards (one for Lu and one for Gd) were prepared in the 0.1, 1, 5, 10, 50, 100 ppb range and diluted with 2% nitric acid solution into 100 mL volumetric flasks. Then 10 mL of sample and standards were transferred to clean 15 mL falcon tubes via a volumetric pipette.

Checking for metal ion leakage

Samples **8b**, **8b′** and **8c** (16 µM, or 16800 ppb Lu) were taken in dialysis bags (MWCO = 1kD) and dialyzed against 150 mL PBS 1x for 7 days without changing diasylate (outside solution in the beaker). The diasylate was collected for each sample and concentrated 30 folds and then analyzed with ICP-MS (detection limit < 0.1 ppb) using a quadrapole Thermo Scientific TM iCAP Q instrument to detect Lu concentration in the diasylate. With a detection limit < 0.1 ppb, no Lu3+ ions were detected in the three solutions, confirming the cage confinement of the metal ions.

Cell viability study:

Cell Culture: For cell experiments 5 different cell lines were used. For hiPSC-derived neural stem cells, cells were derived from human iPSCs (WT126 clone 8; and WT33 clone 1) and grown in proliferation media containing a DMEM/F12 (Invitrogen) supplemented with B27 (Invitrogen), N2 (Stemcell), and bFGF (20ng/mL) (Invitrogen). Cells between passages 5-10 were used for all experiments. For NIH-3T3 (Sigma), MDA-MB231 (ATCC), HeLa (ATCC), and U87MG (ATCC), all cells were grown in DMEM (Invitrogen) with 10% Fetal Bovine Serum supplemented with 1% penicillin/streptomycin.

Biocompatibility analysis: To study the viability of cells after treatment, all 5 cell lines were passaged into 96 well plates at a density of 10,000 cells/cm^2 and grown in growth media. Cells were cultured until approximately 75% confluency and then treated with varying concentrations of our particles (0-32 µM) for 24 hours. After cells were washed 3x with PBS (Invitrogen) the Presto Blue-based cytotoxicity assay (ThermoFisher) was used to measure cell viability. The ultraviolet-visible (UV-Vis) absorption at 570 nm was measured using a plate reader (Tecan Infinite M Plex) for quantifying cell viabilities following manufactures protocol.

Relaxivity measurements:

NMR relaxation measurements were performed at two different magnetic fields: using Nanalysis NMReady-60e desktop 60 MHz (1.4 T) instrument and Varian™ Oxford VNMRS 300 MHz (7.1 T) instrument. The inversion-recovery method

was used to measure T_1. The relaxivities were calculated from linear fits of plots of relaxation rate ($1/T_1$) vs concentrations of the MBT or gadodiamide (Omniscan) molecules. The concentration range of **8d** and **8d'** were in the range of 1.67-11.59 μM, that of **8e** was in the range 4.8-24.0 μM and for Omniscan it was 1.4-21 μM. The Gd^{3+} ion concentration was determined by ICP-MS. The table below summarizes the measurements.

Table S1: The T_1 relaxivity of water-soluble Gd MBTs on 60 MHz (1.4 T) and 300 MHz (7.1 T) NMR, together with a representative commercial GBCA gadodiamide.

Molecule	r_1 (1.4 T) $mM^{-1}S^{-1}$	r_1 (7.1 T) $mM^{-1}S^{-1}$
**8d**	60.6	20.8
**8d'**	51.6	19.4
**8e**	35.5	13.3
Gadodiamide (Omniscan)	4.4	3.9

Magnetic resonance imaging:

By the application of 1.0T Aspect M2 MRI System (Aspect Magnet Technologies Ltd., Netanya, Israel). FOV 45 cm, matrix size 256x256, slice thickness 2 mm, TR 6000 ms, TE 10.6 ms, and 9 inversion times TIs: 300, 500, 800, 1100, 1500, 1800, 2500, 3000, 4000 ms. All images were consistent in terms of the relative contrasts of samples, and the result with TI = 4000 ms was used as a representative image in the manuscript.

ROS generation and EPR measurements:

The two types of Reactive Oxygen Species were prepared as the following:

Type I -

Solution recipe: 40 μL MBT **8** solution, 12 μL NADH, 24μL buffer, 24 μL spin trap DEPMPO solution, 20 μL L-hystidine solution

MBT **8**: 90 μM **8b**, **8b'** or **8c** in mQ water.

NADH: 0.10 mM solution in mQ water.

Buffer: 5 mM DETAPAC in 300 mM phosphate buffer, pH 7.

Spin trap DEPMPO solution: 250 mM DEPMPO in DMSO.

L-hystidine solution: 60 mM L-histidine in mQ water.

Type II -

Solution recipe:

40 μL MBT **8** solution, 24μL buffer, 56 μL spin trap 4-oxo TEMP solution.

MBT **8**: 90 μM **8b**, **8b'** or **8c** in mQ water.

Buffer: 300 mM phosphate buffer, pH 7.

Spin trap 4-oxo TEMP solution: 171 mM 4-oxo-TEMP in mQ water.

Each solution type was saturated with oxygen by bubbling pure oxygen for one minute in the solution placed inside a 1 mL glass vial. Following this step, glass vials were placed inside a green light irradiation enclosure for 30 minutes. The green light was generated by a LED strip affixed in an ellicoidal pattern inside the cylindrical enclosure. The 300 LEDs, producing 2,360 lumens in total, were aimed toward the center of the enclosure. All solutions from the same set were irradiated at the same time. The geometry was such that the vials from the same set were placed on a 7 cm circle inside the enclosure at mid height. Enclusure dimensions: 15 cm diameter, 15 cm tall. The enclosure is made of a reflective metal to maximize the samples illumination.

Quantitative EPR measurements

10 μL aliquots of each irradiated solution were placed into an EPR quartz tube for analysis. A Bruker EMX nano X-band spectrometer was used to recorded the spectra and extract the radical spin densities via the built-in quantitative EPR

function. The EPR spectra were all acquired using the same settings (temperature 295 K, microwave frequency 9.65 GHz, microwave power 10 mW, receiver gain 40 dB, modulation amplitude 1.00 G, modulation frequency 100 kHz, sweep time 6.67 sec, 10 scans average).

Supplemental procedures for computations:

Kohn-Sham density functional theory (DFT) calculations were performed with the Amsterdam Density Functional (ADF, v. 2019) package[9] using the non-hybrid Perdew-Burke-Ernzerhof (PBE)[10] density functional, in conjunction with all-electron triple-ζ polarized (TZP) Slater-type orbital (STO) basis sets quality.[11,12] Scalar relativistic (SR) zero-order regular approximation (ZORA) was included for relativistic effects.[13] ‘D3’ dispersion corrections by Grimme were also considered,[14] which are strictly necessary to assess the fullerene-fullerene interactions (see Table S5).

4. Supplemental characterization data

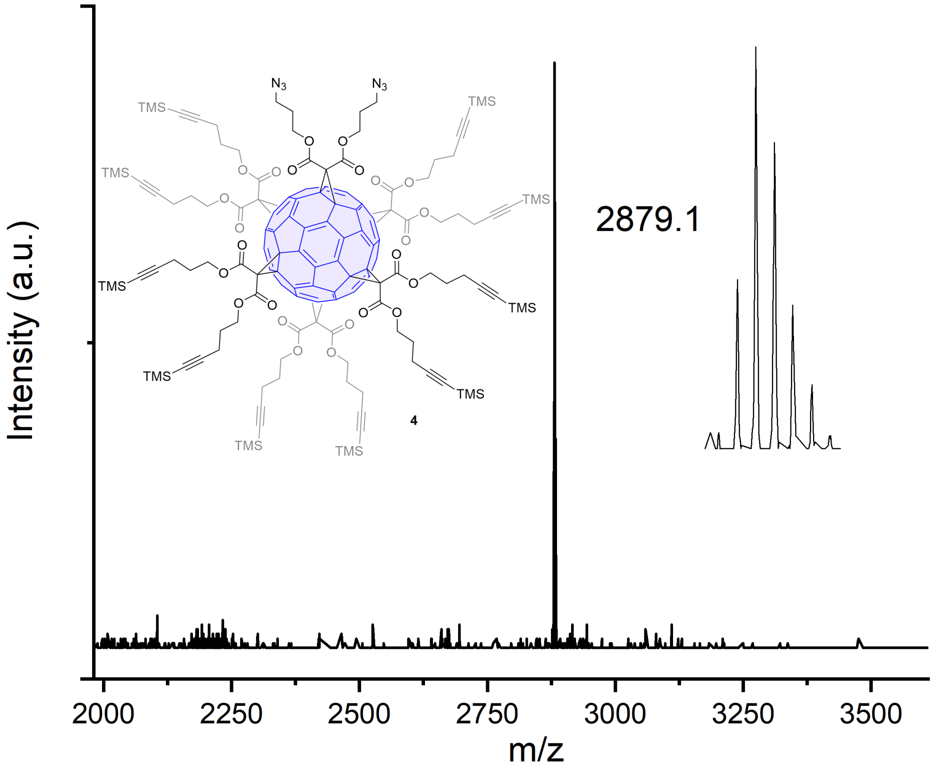

Figure S1. MALDI-TOF mass spectrometry of **4** (m/z calculated 2878.9, found 2879.1)

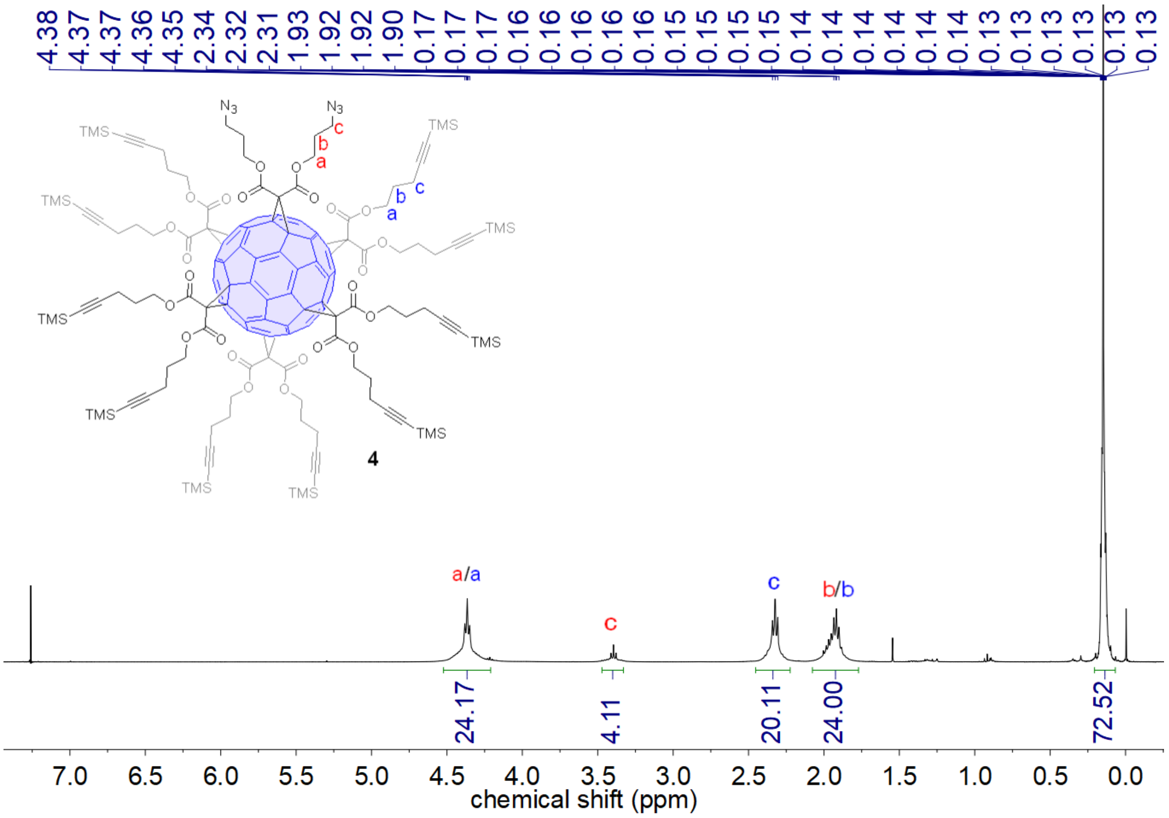

Figure S2. ^{1}H NMR spectrum of **4**.

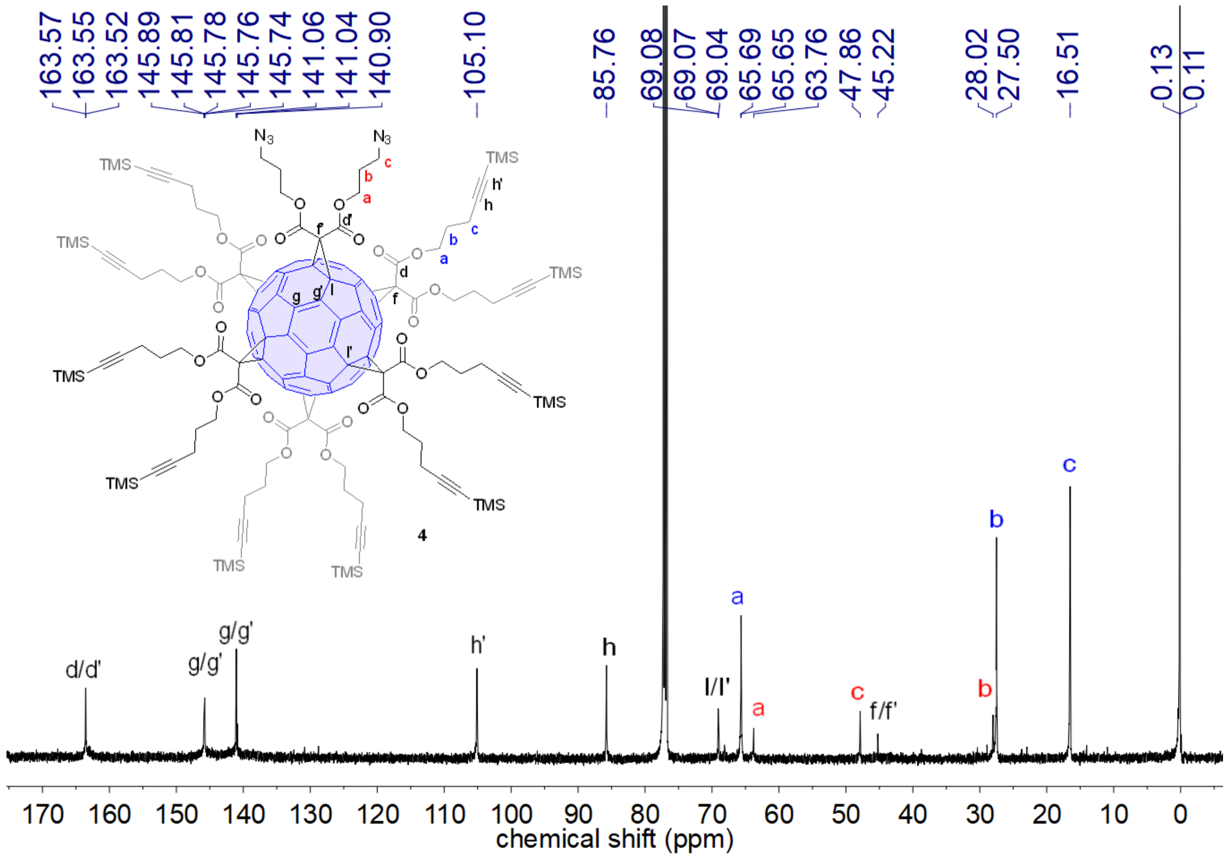

Figure S3. ^{13}C NMR spectrum of **4**.

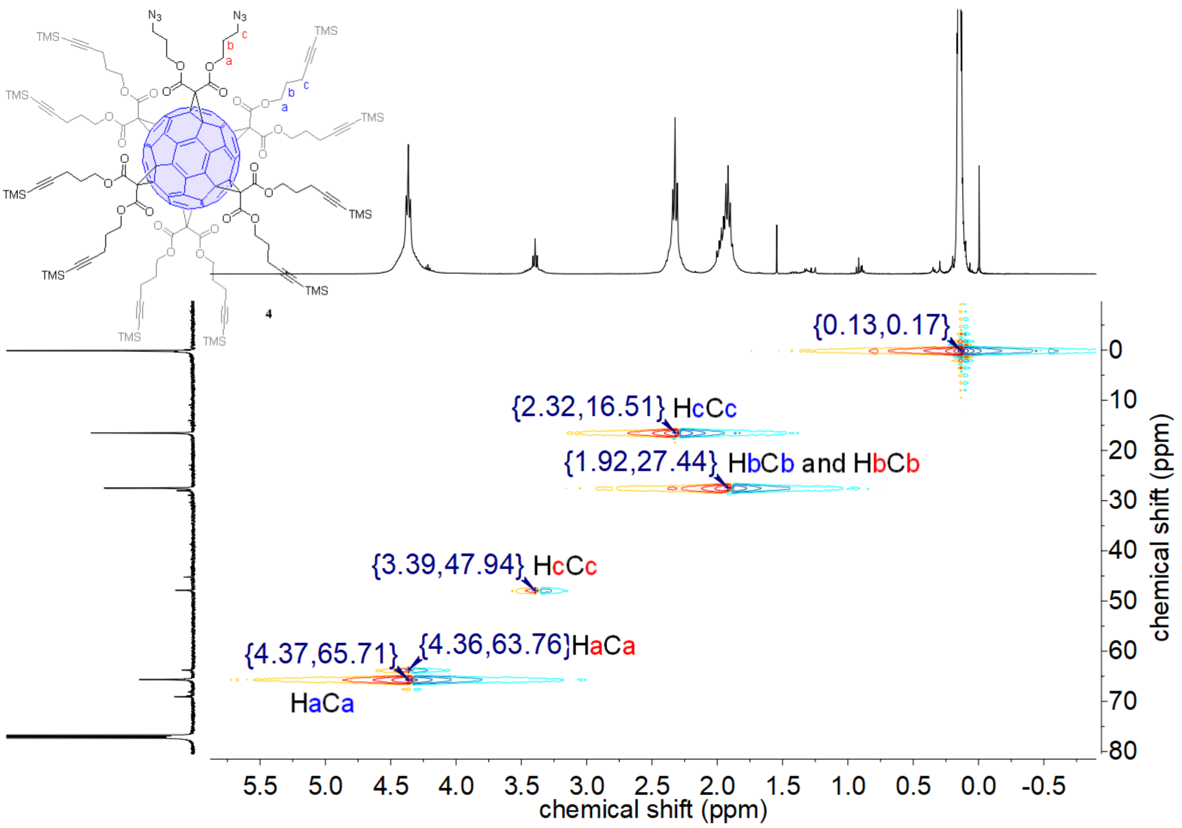

Figure S4. HMQC NMR spectrum of **4**.

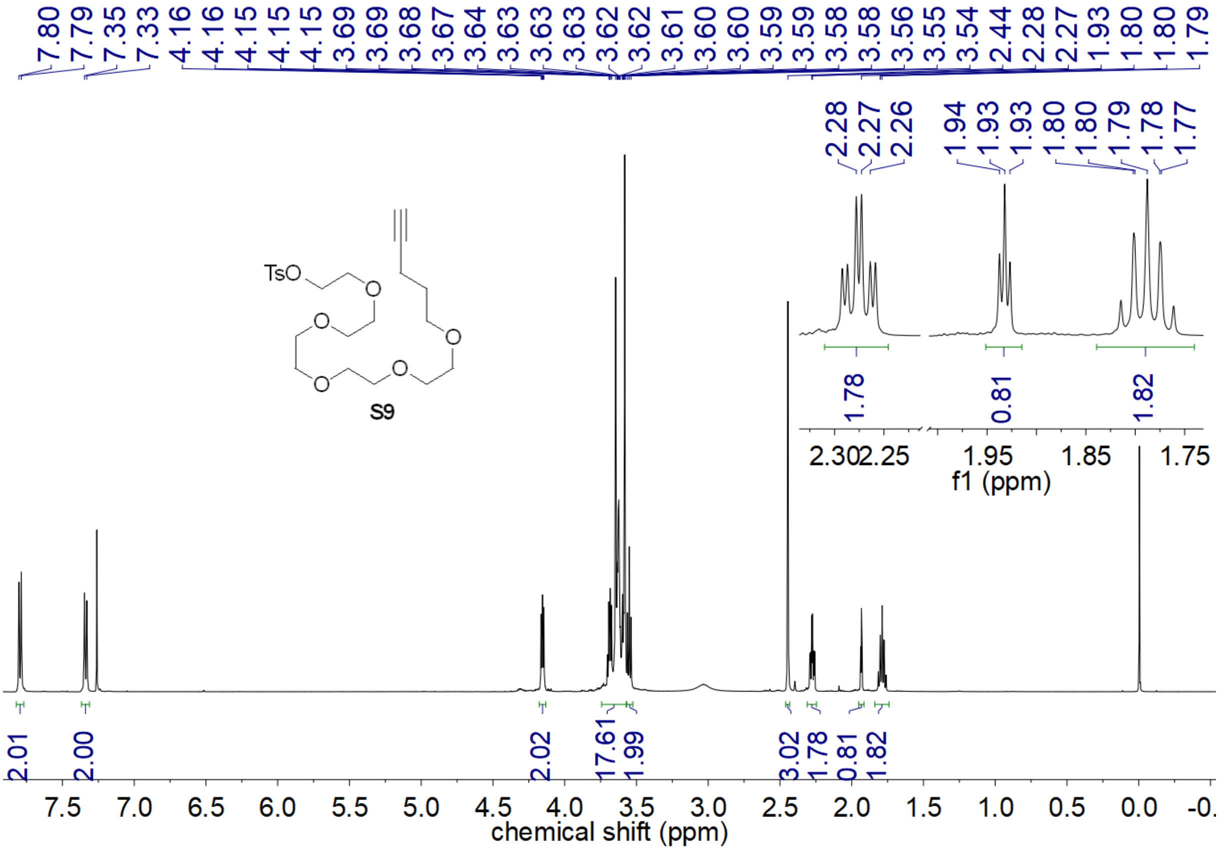

Figure S5. ^{1}H NMR spectrum of **S9**.

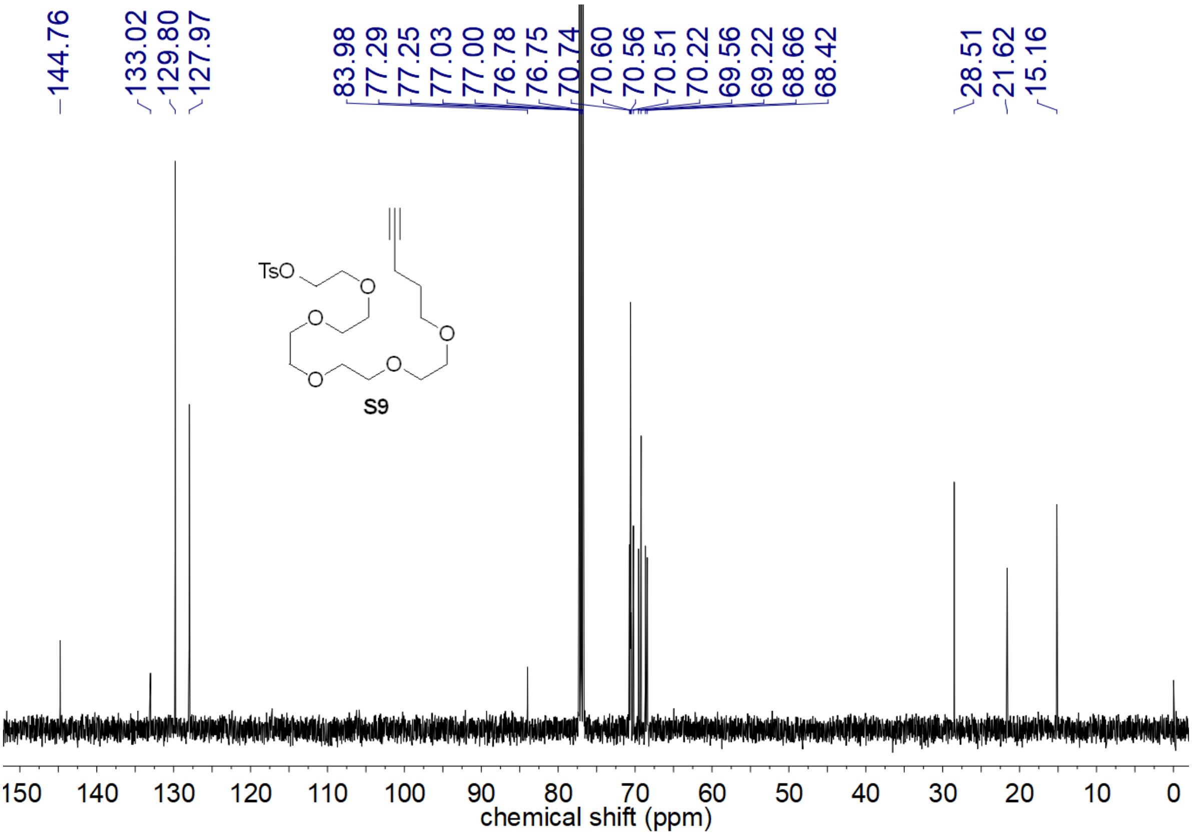

Figure S6. ^{13}C NMR spectrum of **S9**.

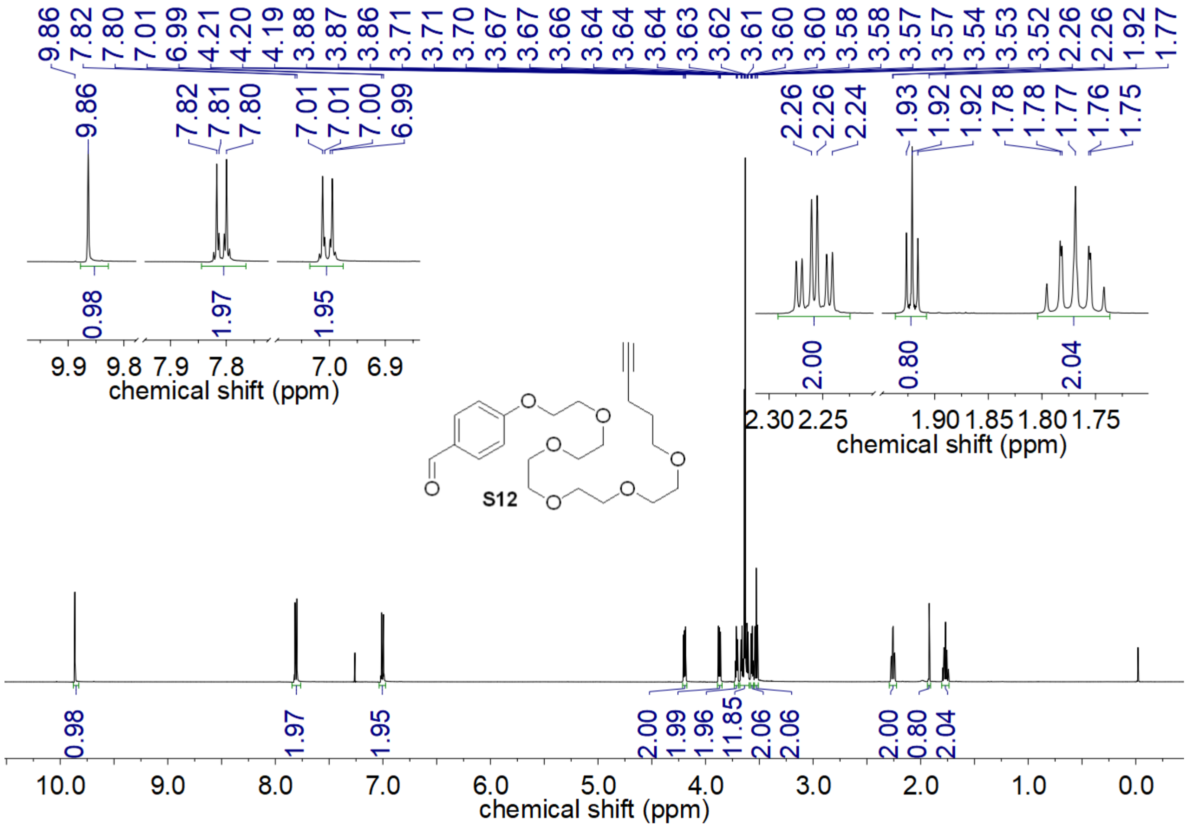

Figure S7. ^{1}H NMR spectrum of **S12**.

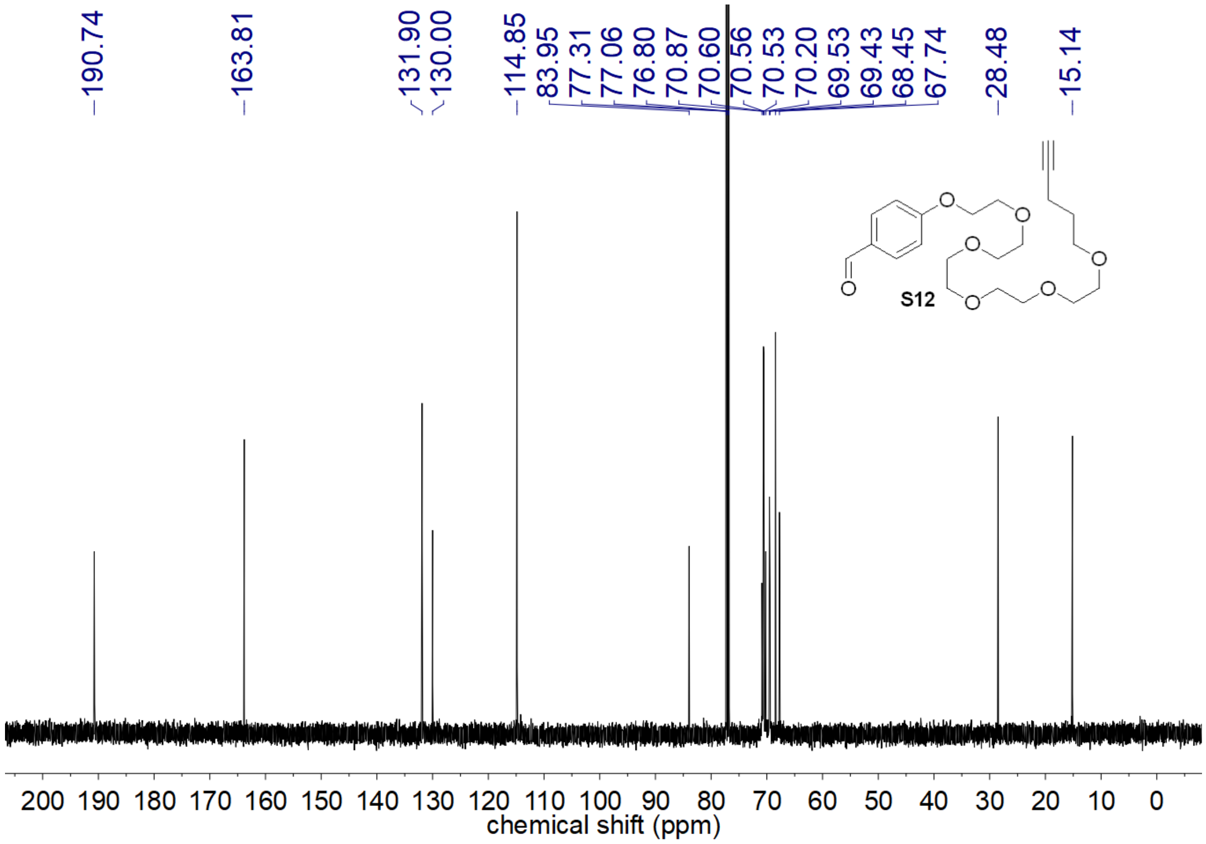

Figure S8. ^{13}C NMR spectrum of **S12**.

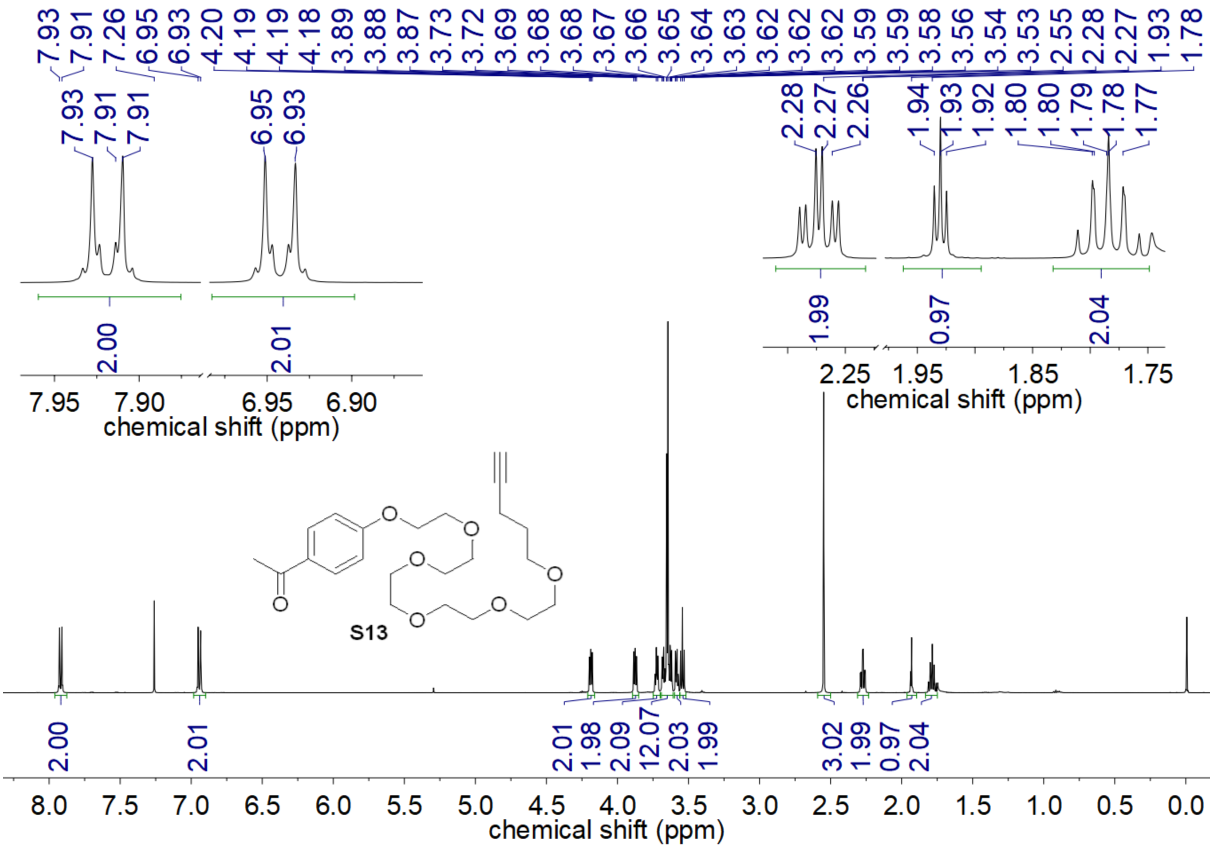

Figure S9. ^{1}H NMR spectrum of **S13**.

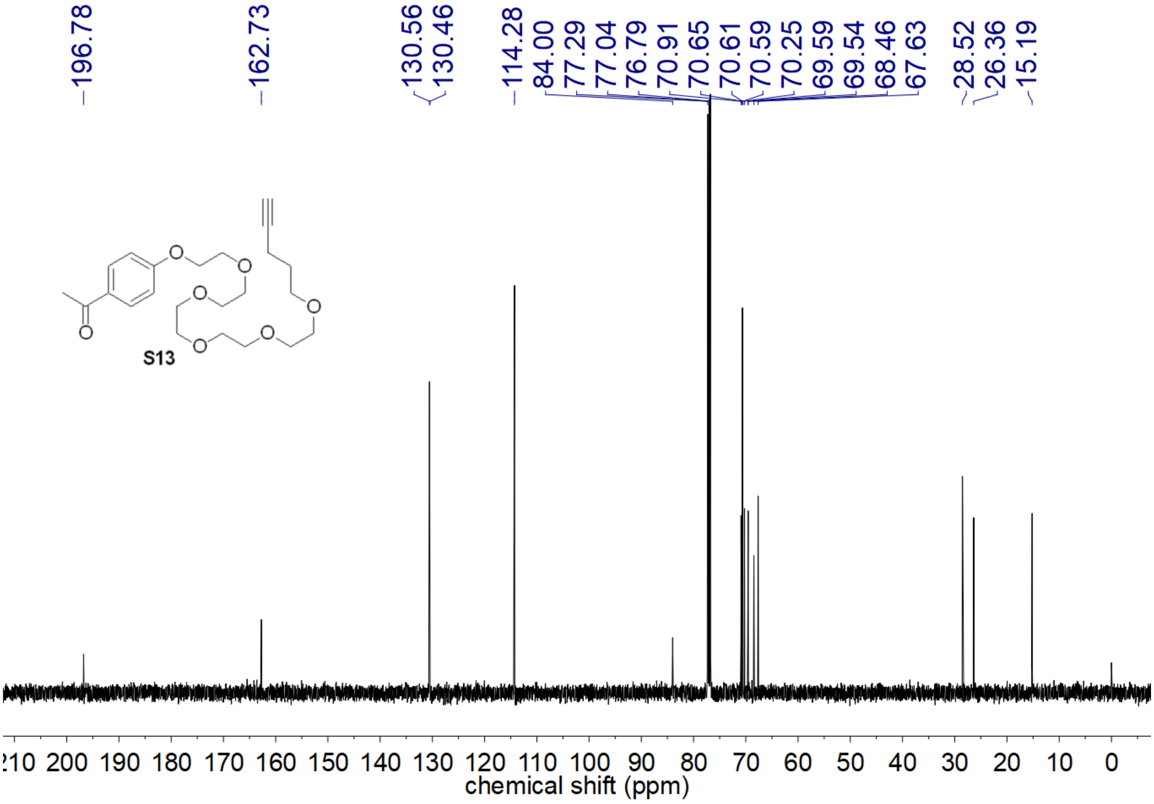

Figure S10. ^{13}C NMR spectrum of **S13**.

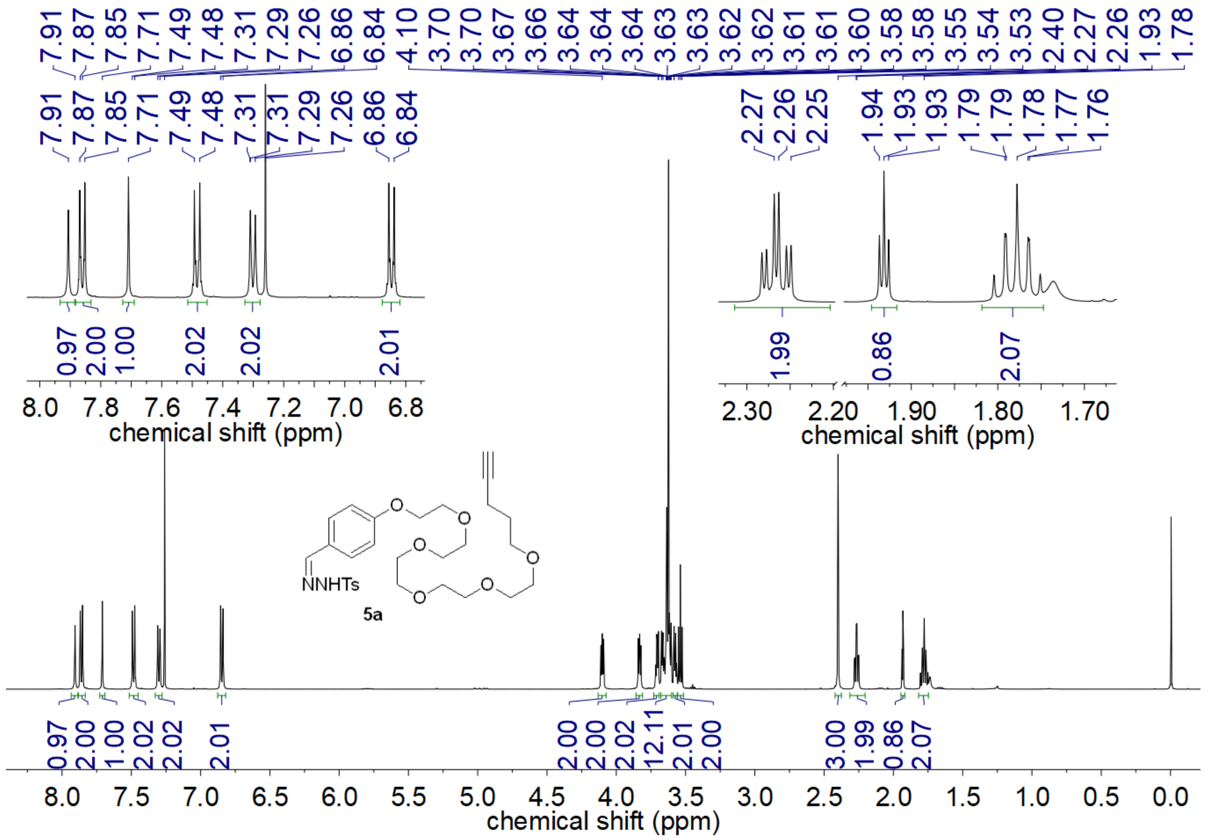

Figure S11. ^{1}H NMR spectrum of **5a**.

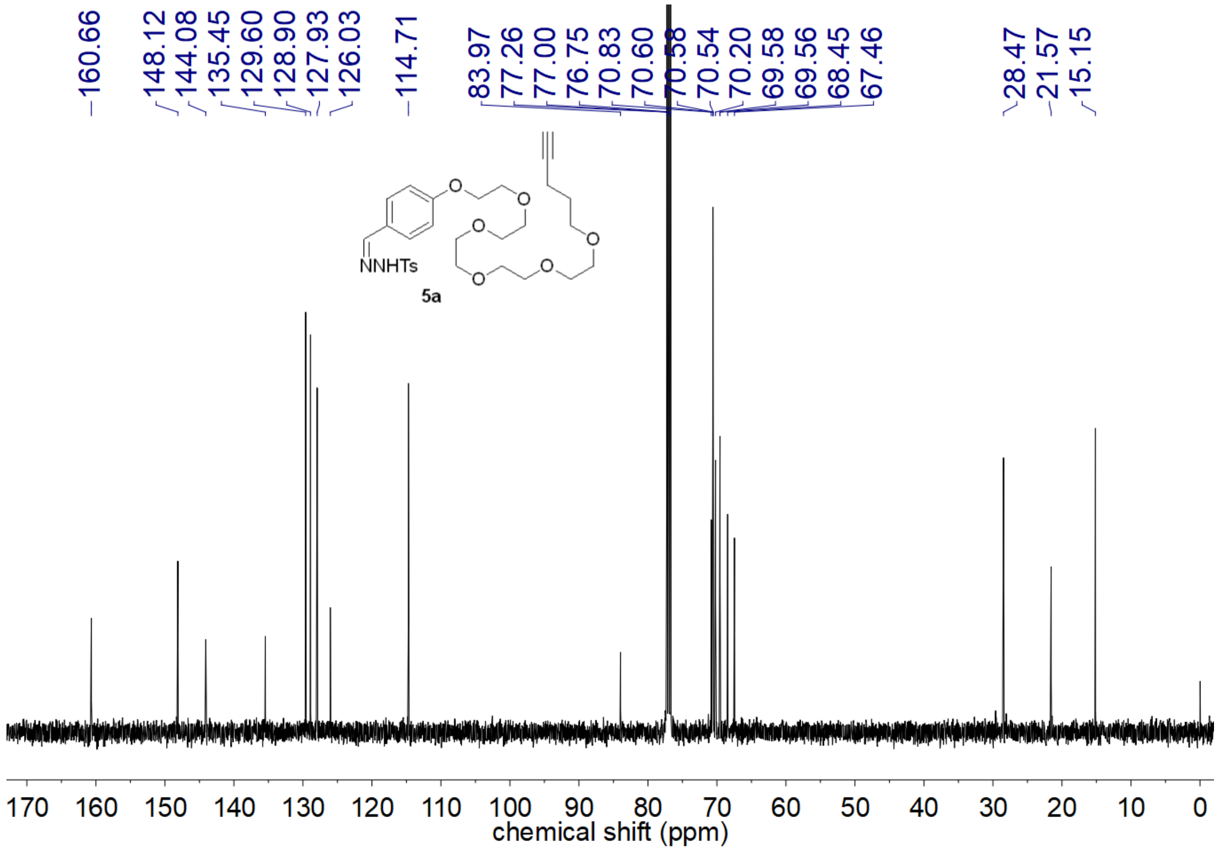

Figure S12. ^{13}C NMR spectrum of **5a**.

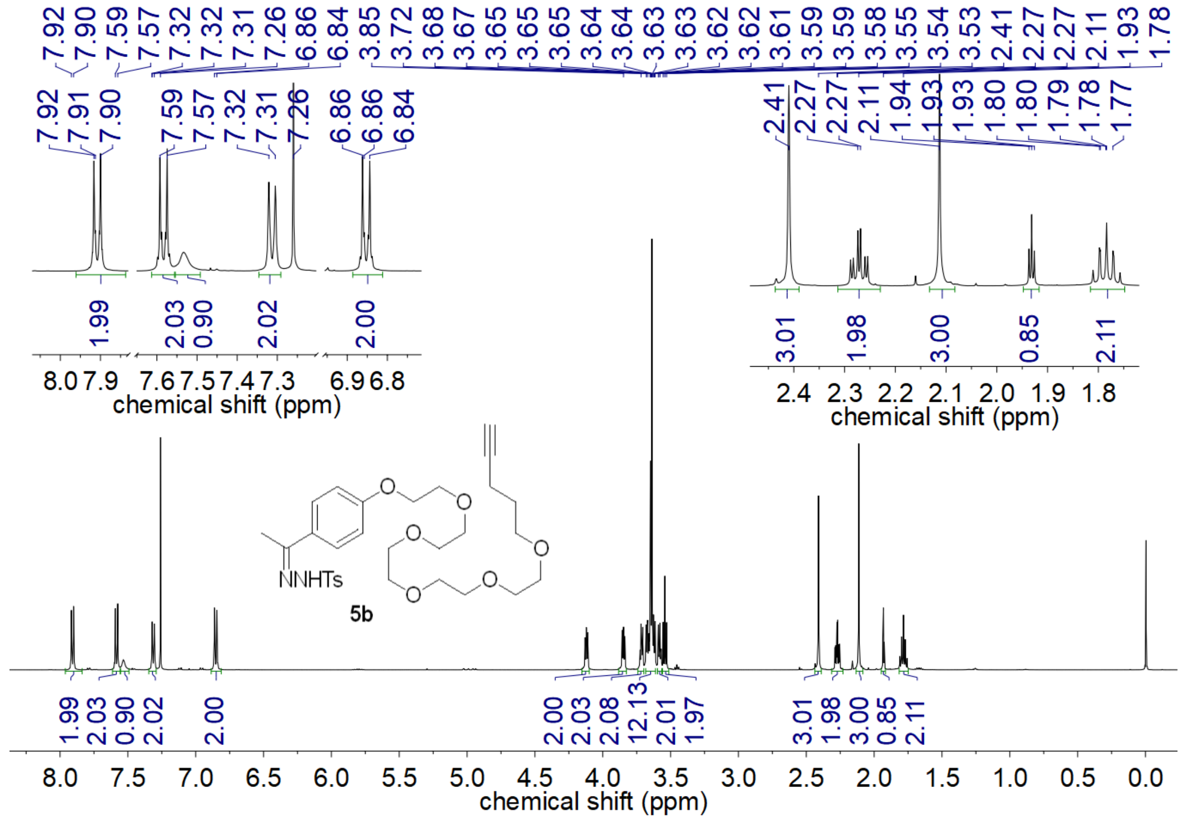

Figure S13. ^{1}H NMR spectrum of **5b**.

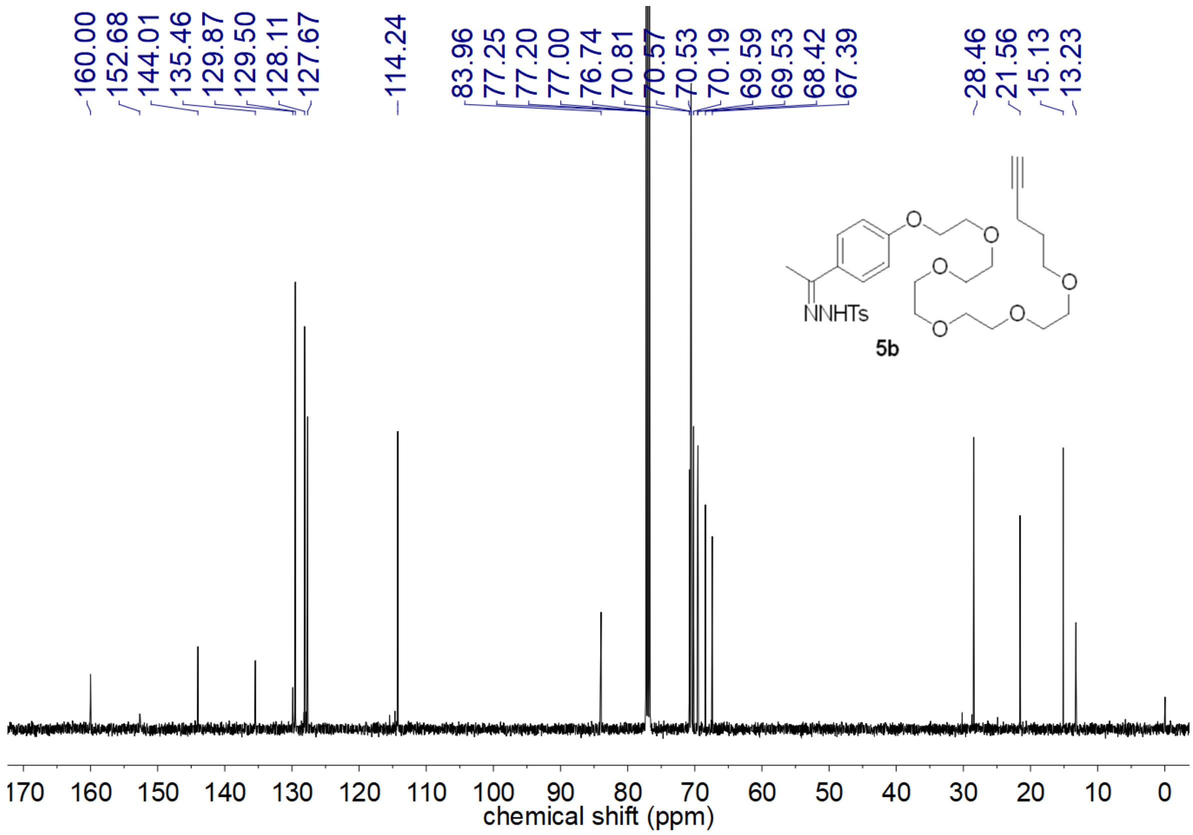

Figure S14. ^{13}C NMR spectrum of **5b**.

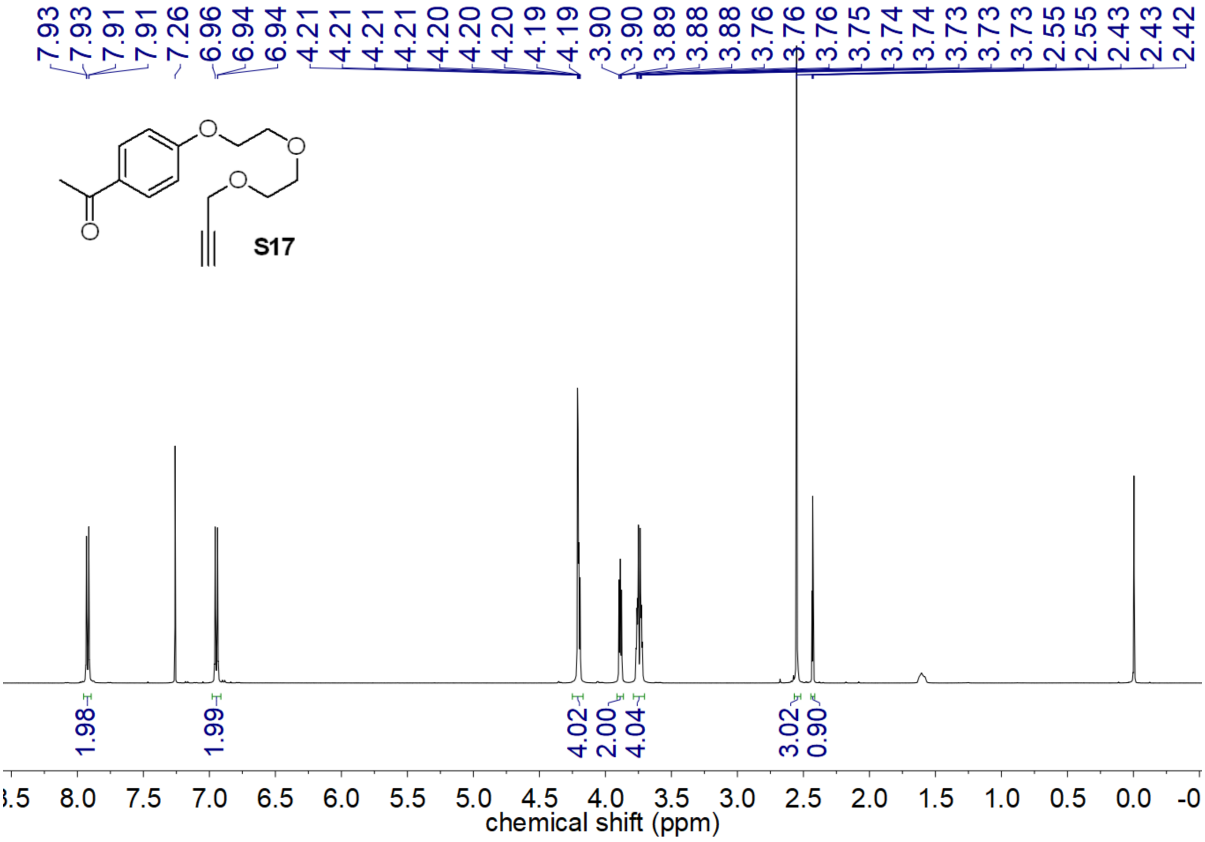

Figure S15. ^{1}H NMR spectrum of **S17**.

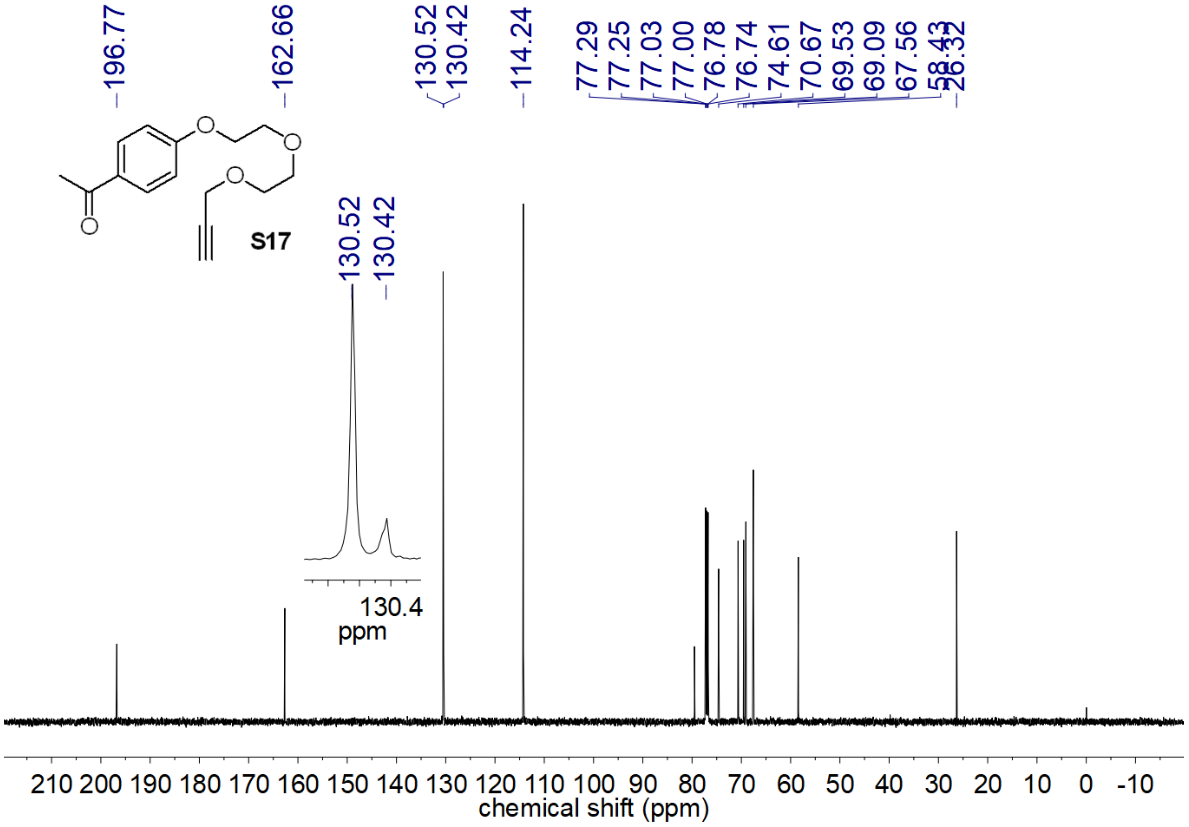

Figure S16. ^{13}C NMR spectrum of **S17**.

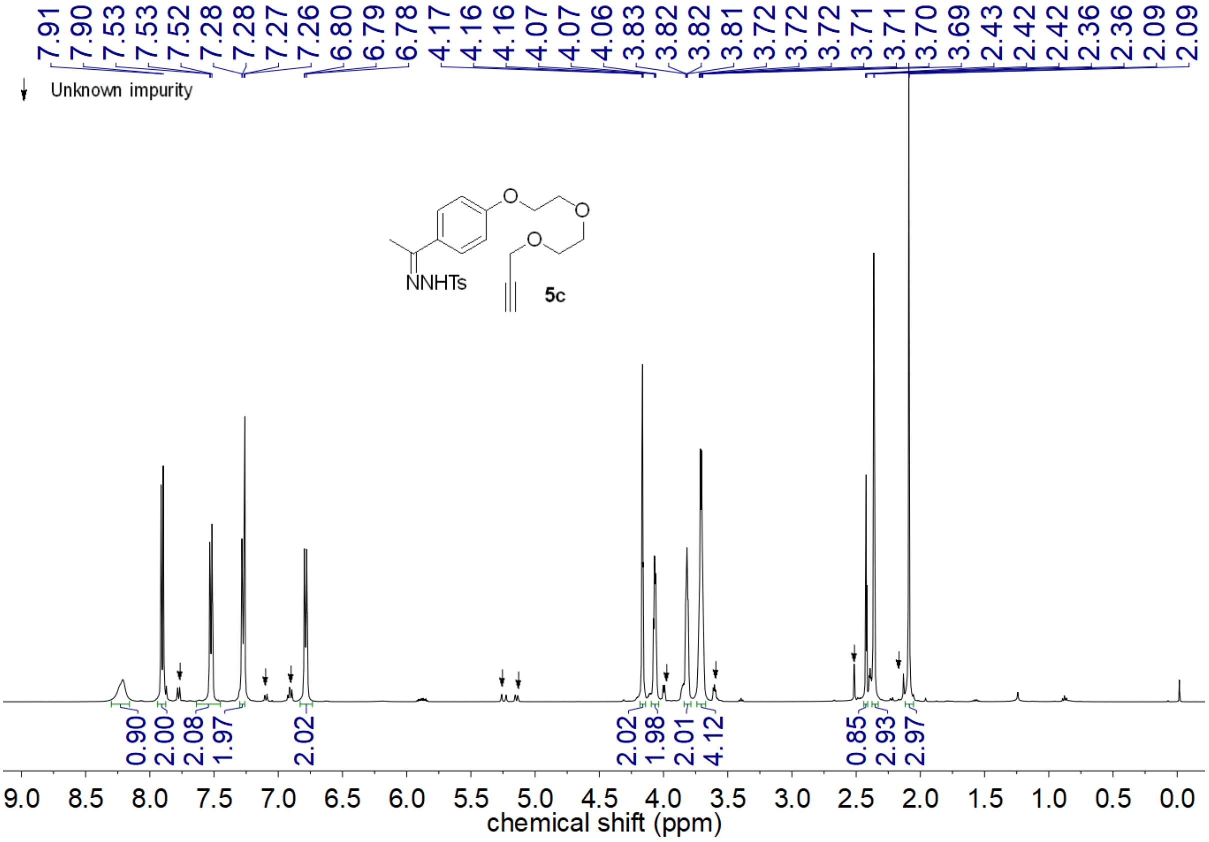

Figure S17. ^{1}H NMR spectrum of **5c**.

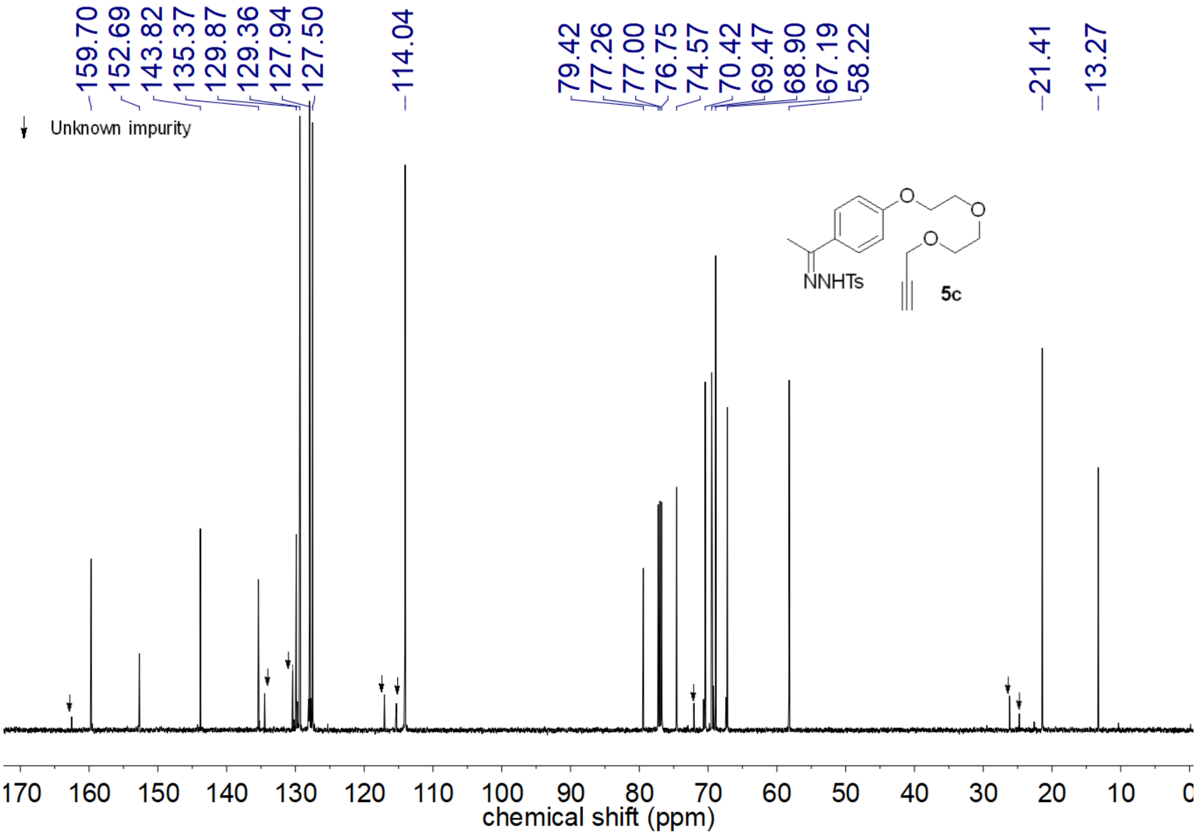

Figure S18. ^{13}C NMR spectrum of **5c**.

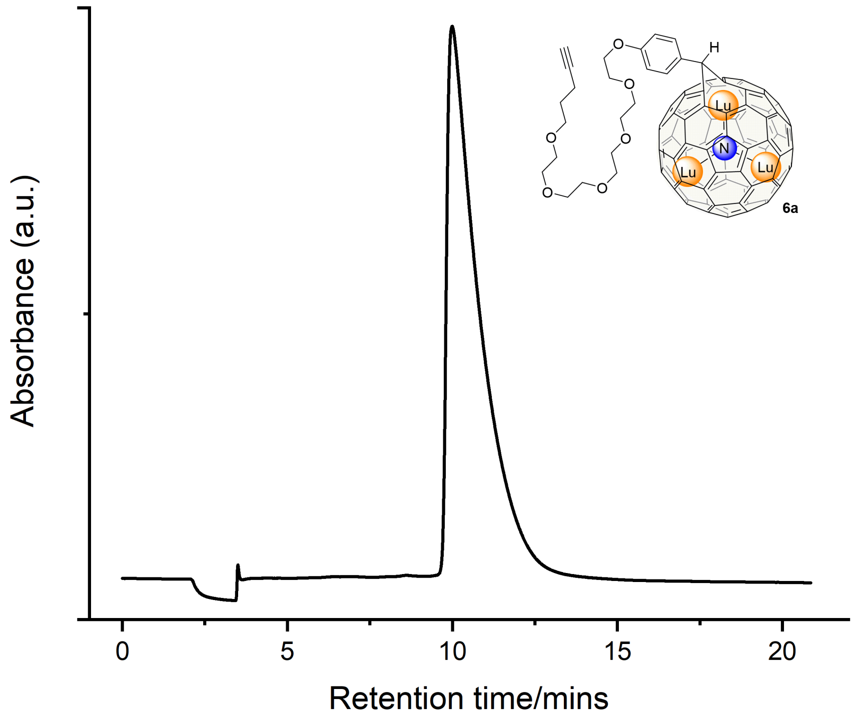

Figure S19. HPLC chromatogram (1.5 mL/minute, toluene : MeOH = 95 : 5, Buckyprep D) of **6a**.

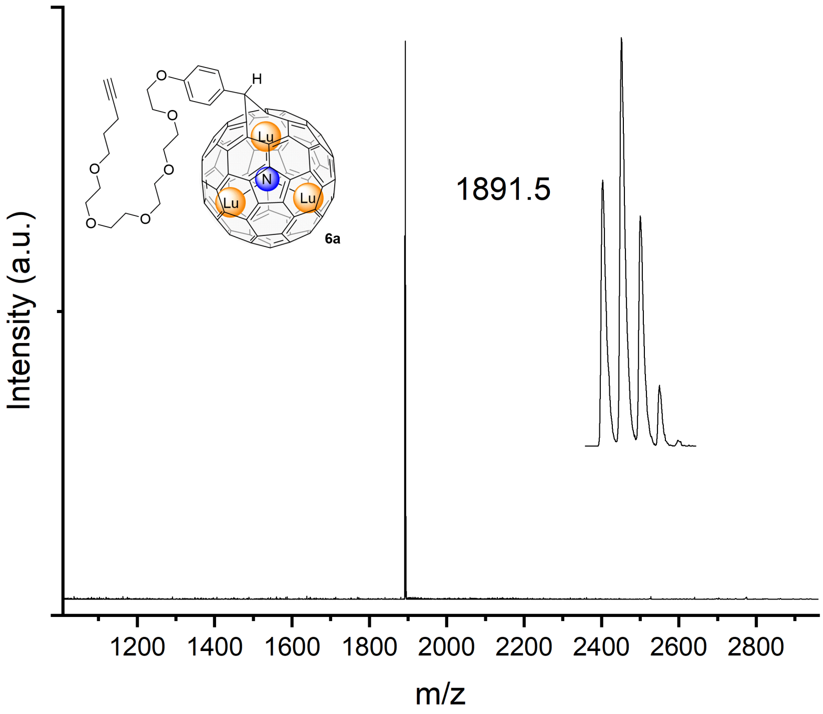

Figure S20. MALDI-TOF mass spectrometry of **6a** (m/z calculated 1891.0, found 1891.5)

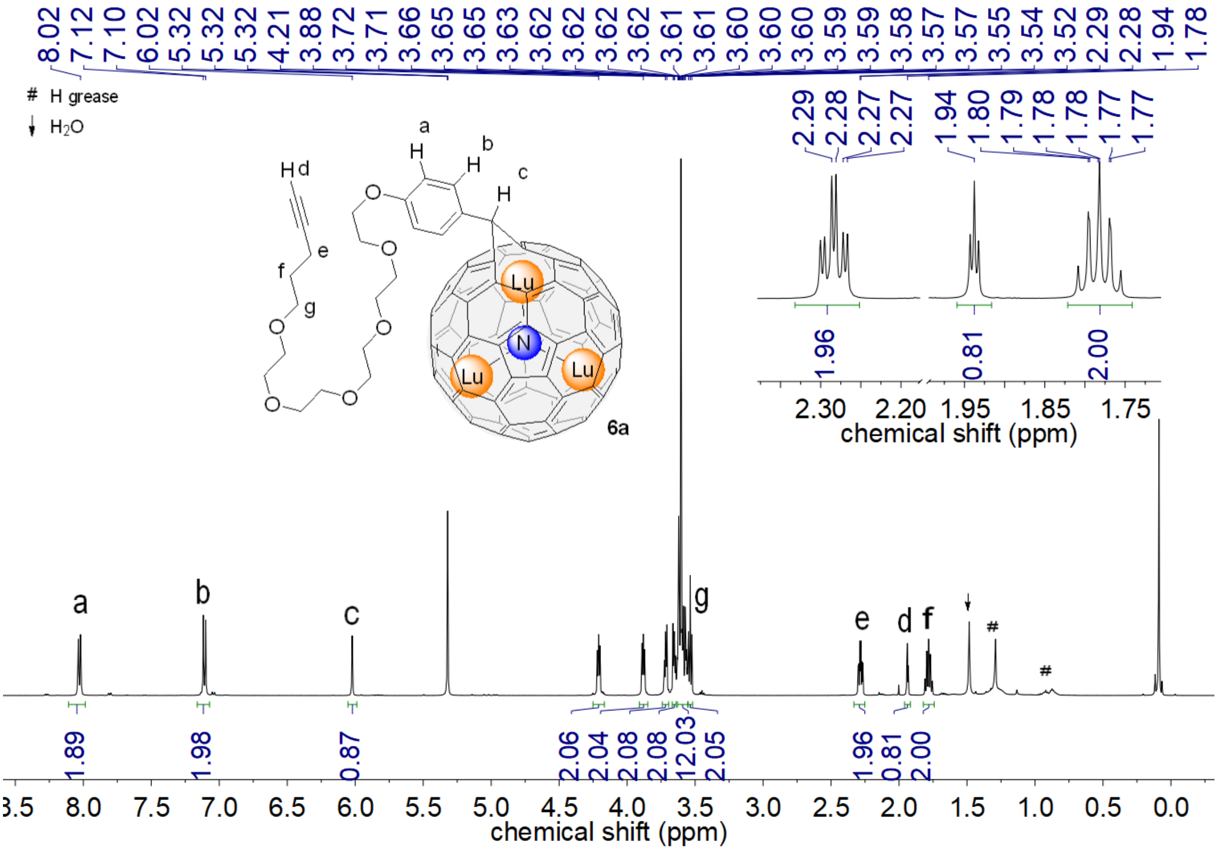

Figure S21. ¹H NMR spectrum of **6a**.

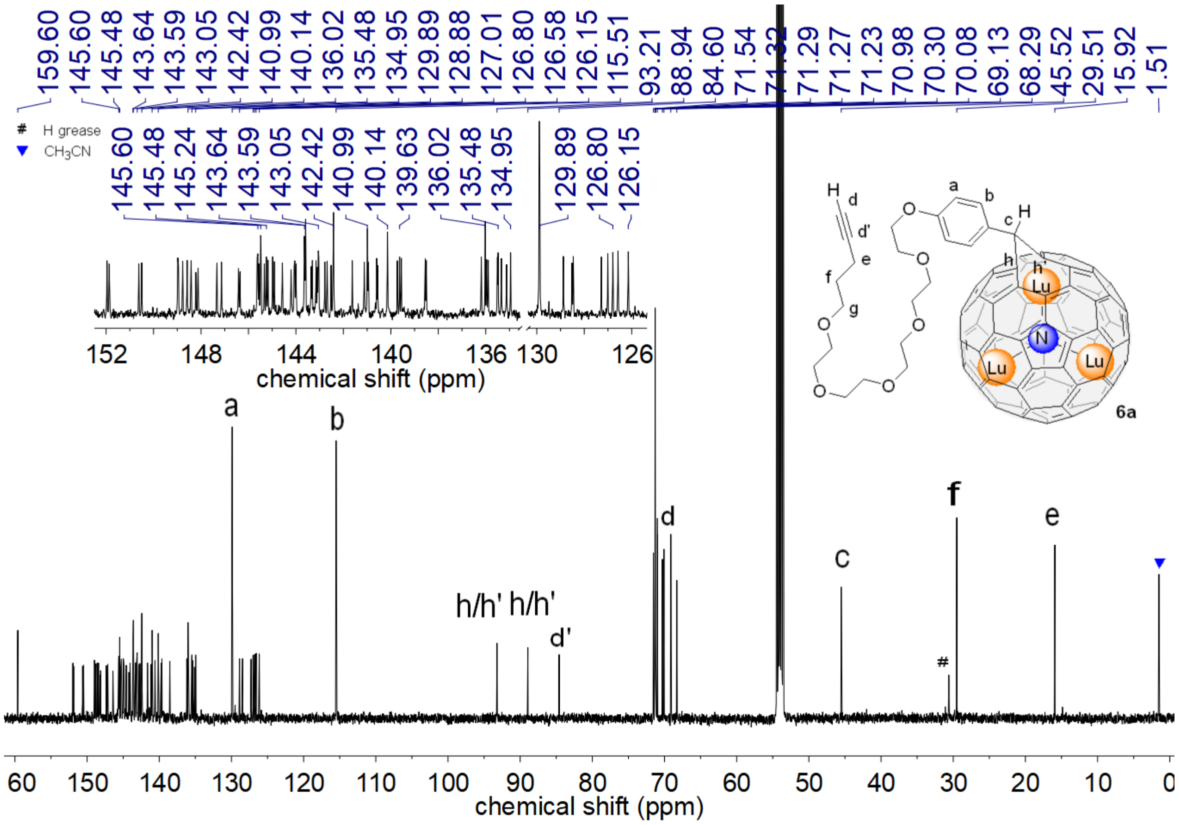

Figure S22. ¹³C NMR spectrum of **6a**.

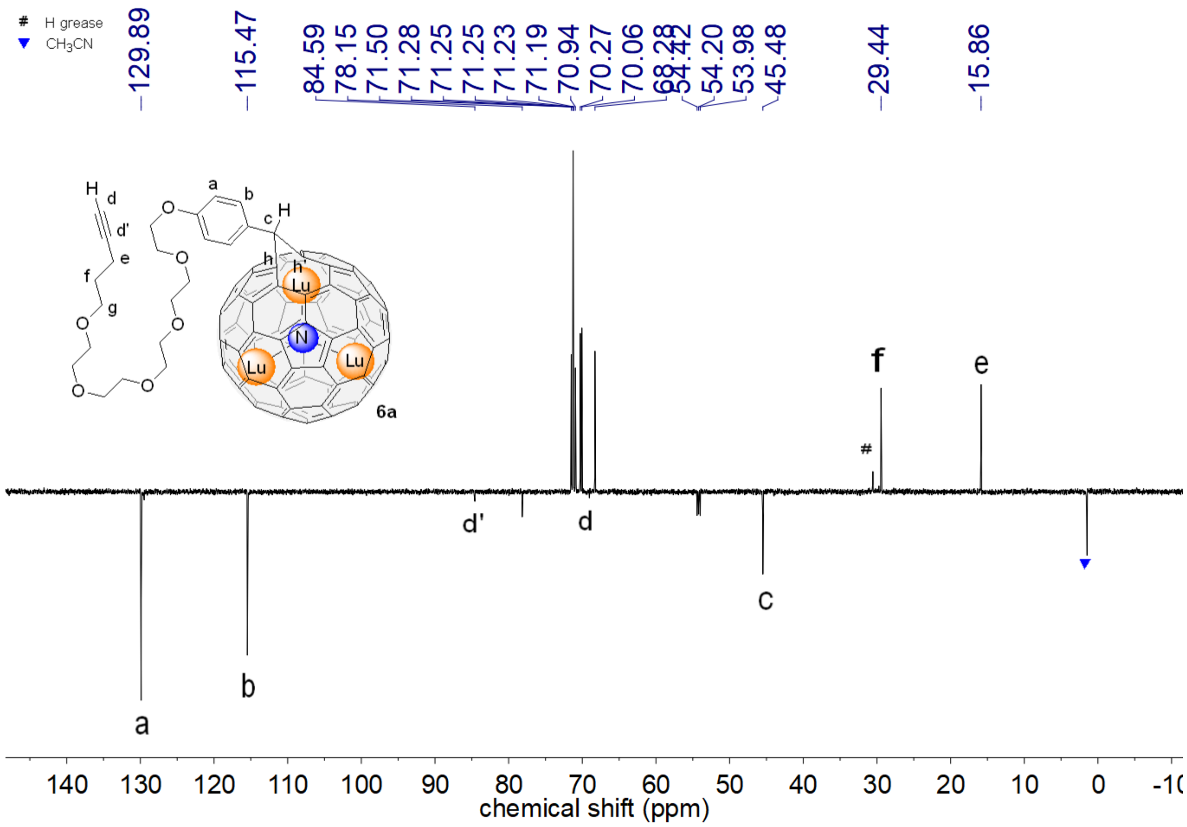

Figure S23. DEPT-135 spectrum of **6a**.

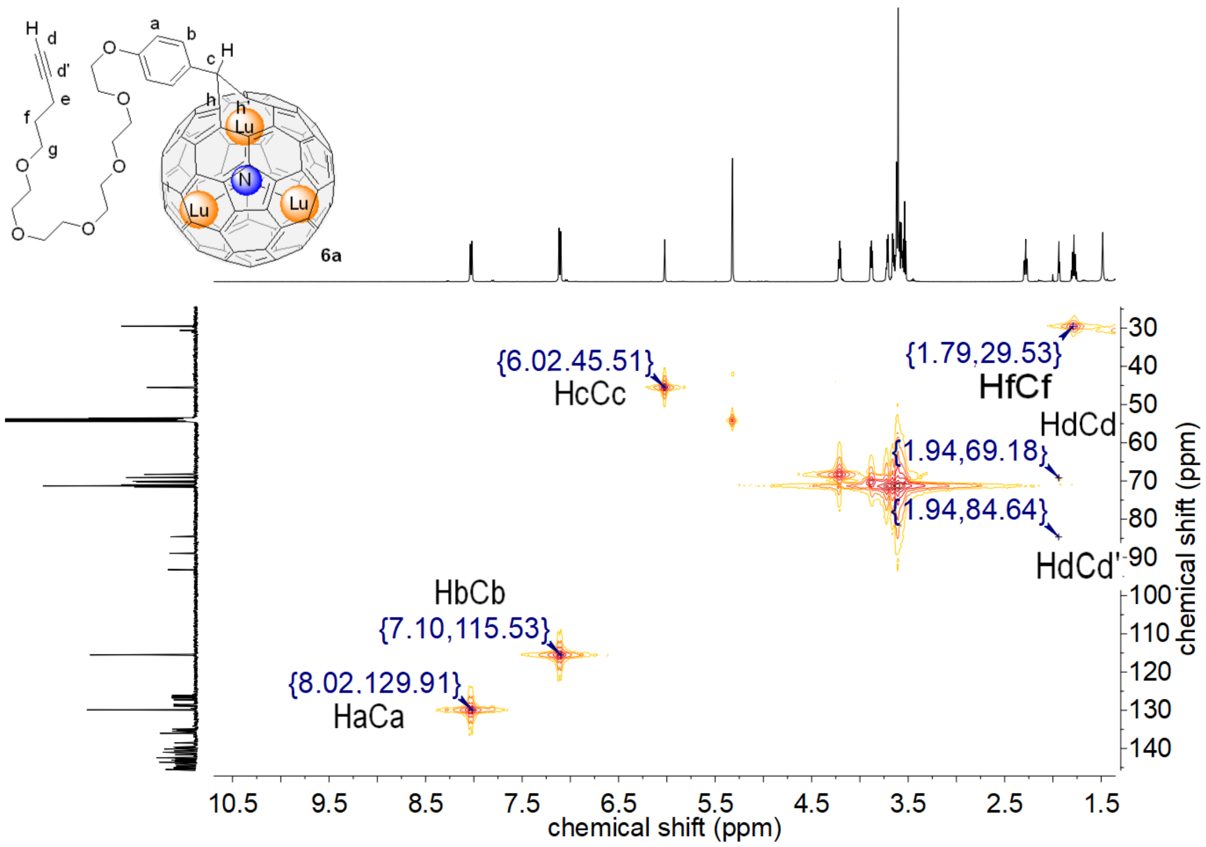

Figure S24. HMQC NMR spectrum of **6a**.

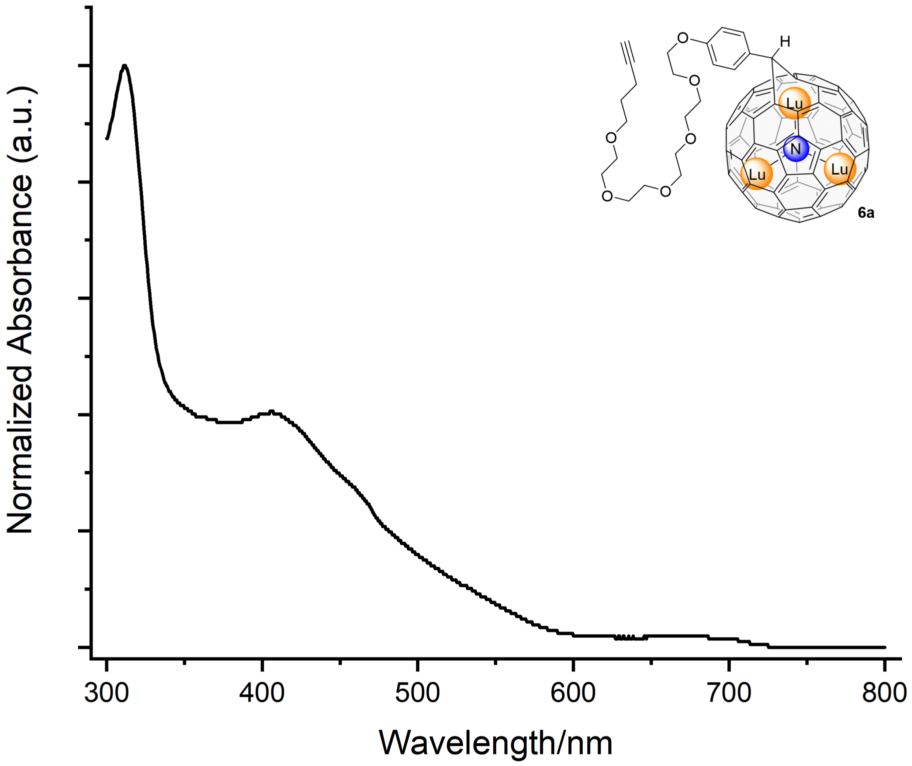

Figure S25. UV-vis spectrum of **6a** in $CHCl_3$.

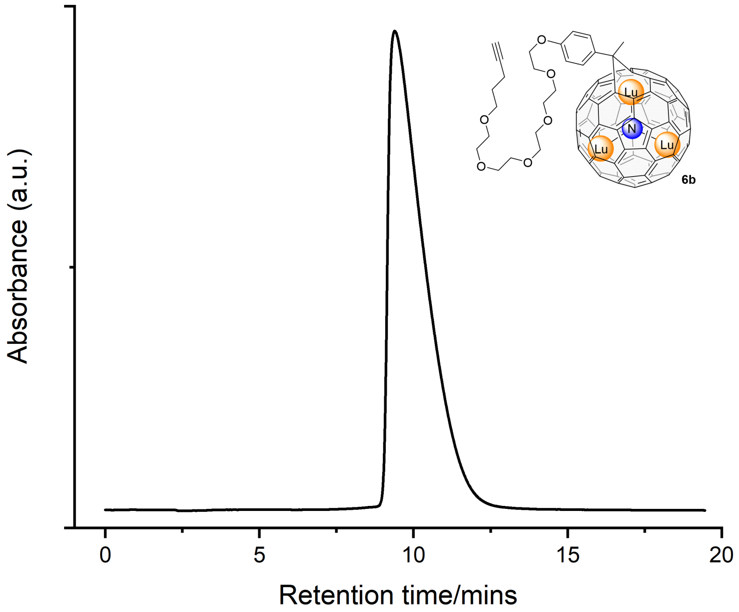

Figure S26. HPLC chromatogram (1.5 mL/minute, toluene : MeOH = 95 : 5, Buckyprep D) of **6b**.

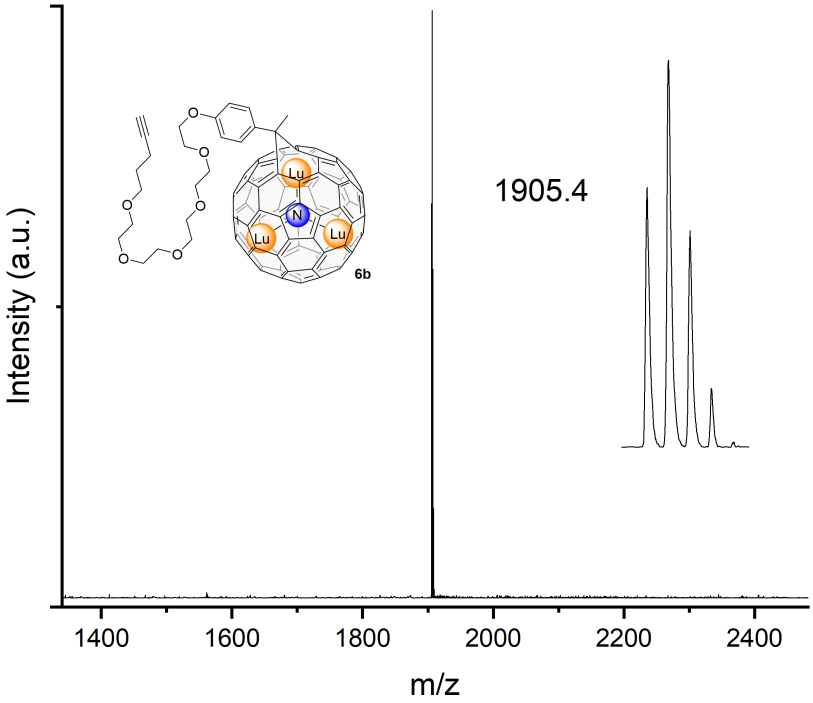

Figure S27. MALDI-TOF mass spectrometry of **6b** (m/z calculated 1905.1, found 1905.4)

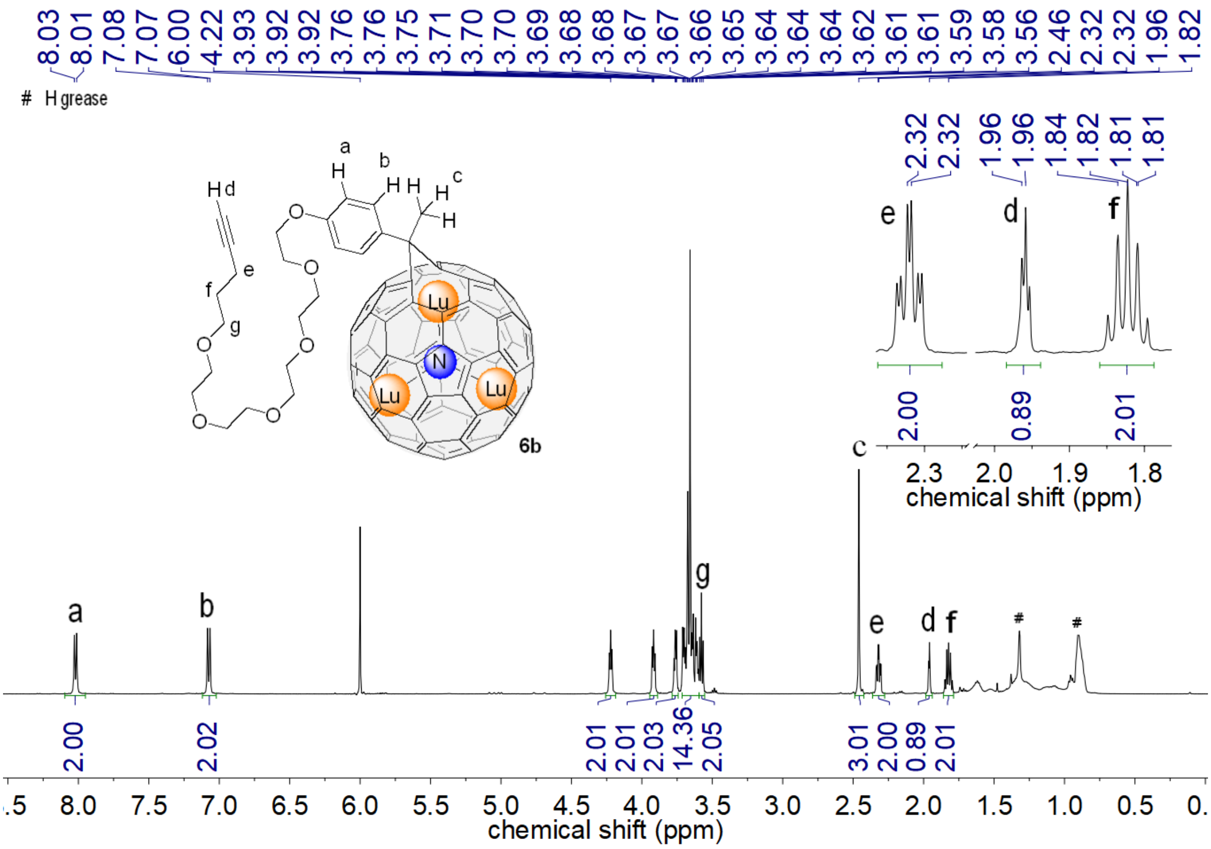

Figure S28. ^{1}H NMR spectrum of **6b**.

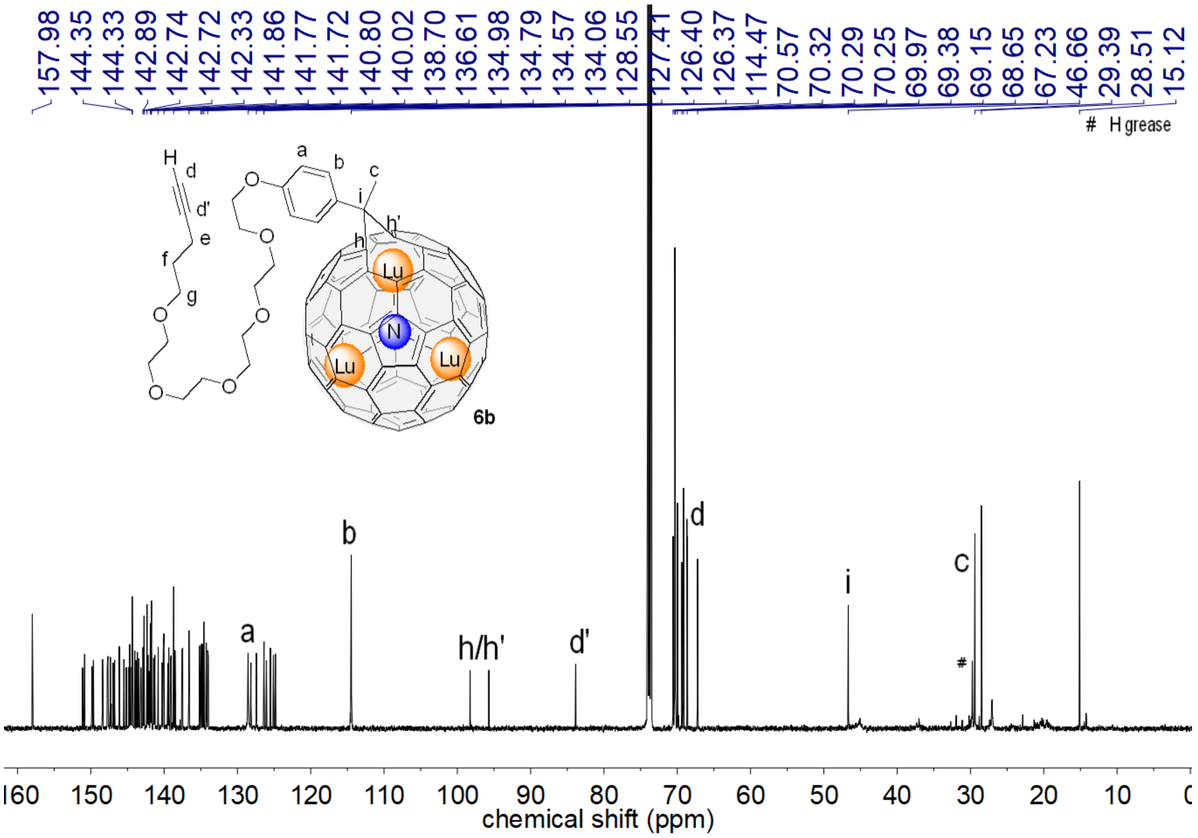

Figure S29. ^{13}C NMR spectrum of **6b**.

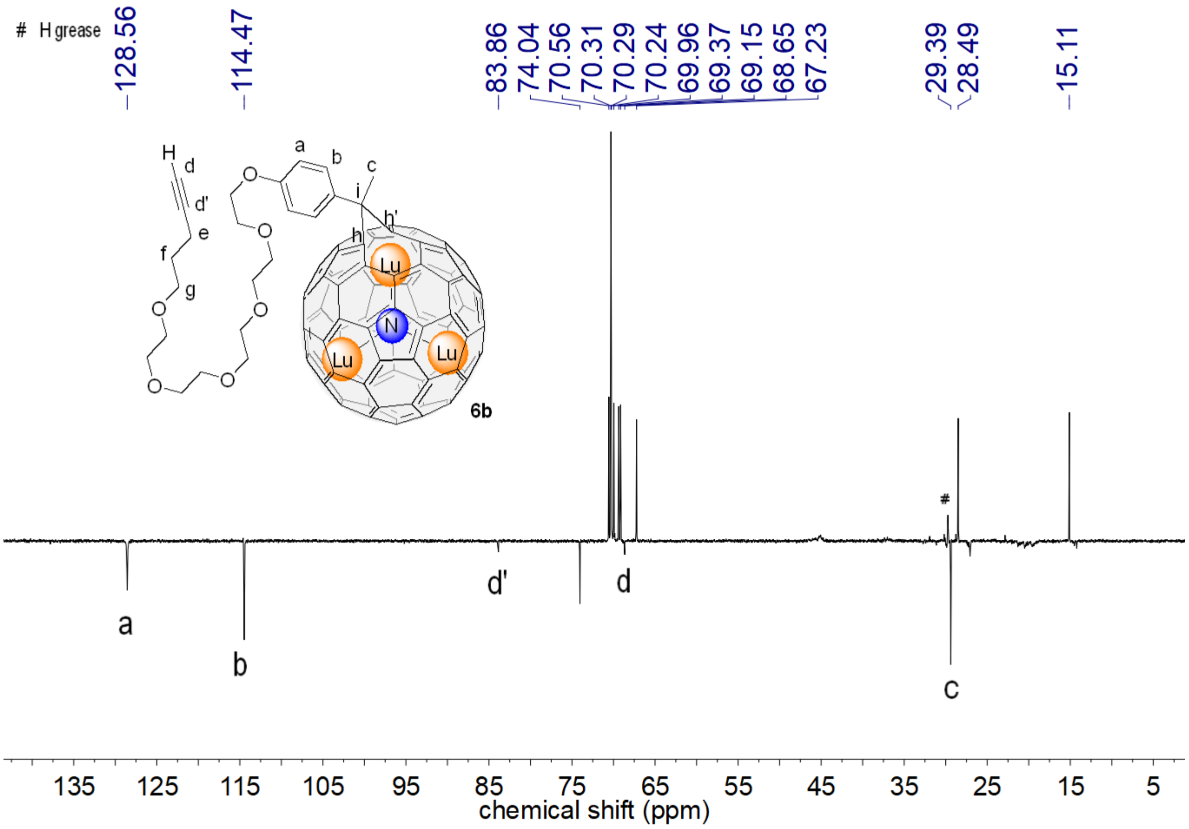

Figure S30. DEPT-135 spectrum of **6b**.

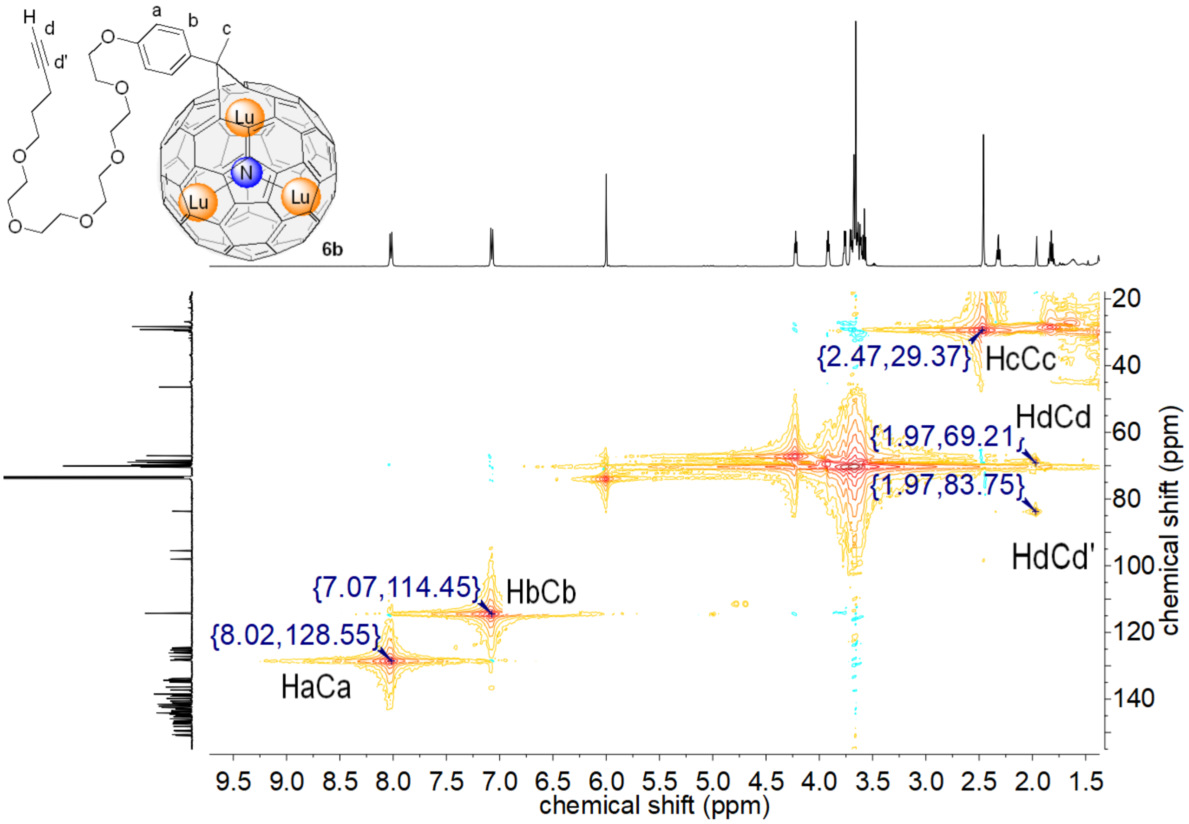

Figure S31. HMQC NMR spectrum of **6b**.

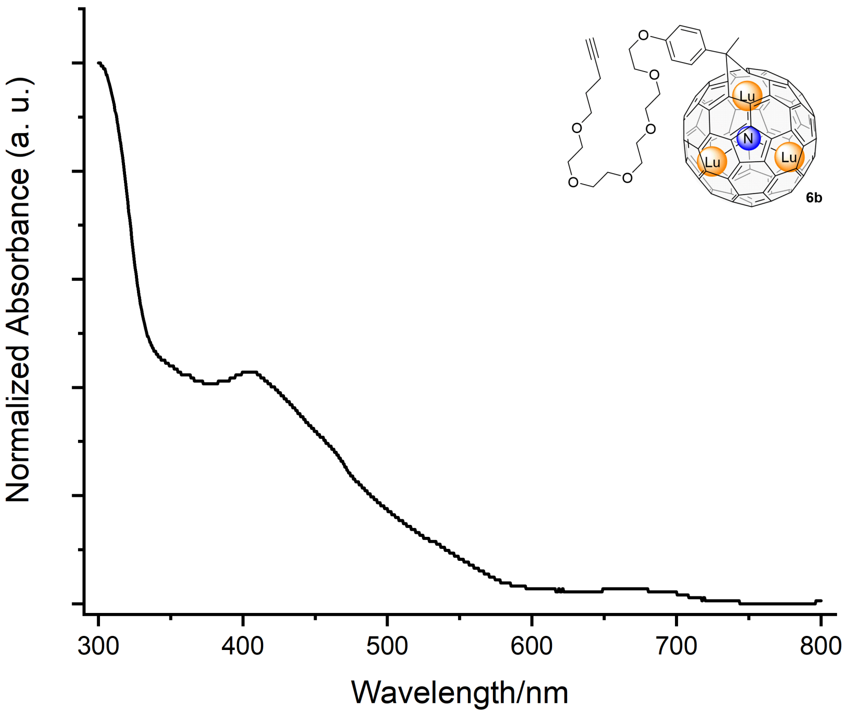

Figure S32. UV-vis spectrum of **6b** in $CHCl_3$.

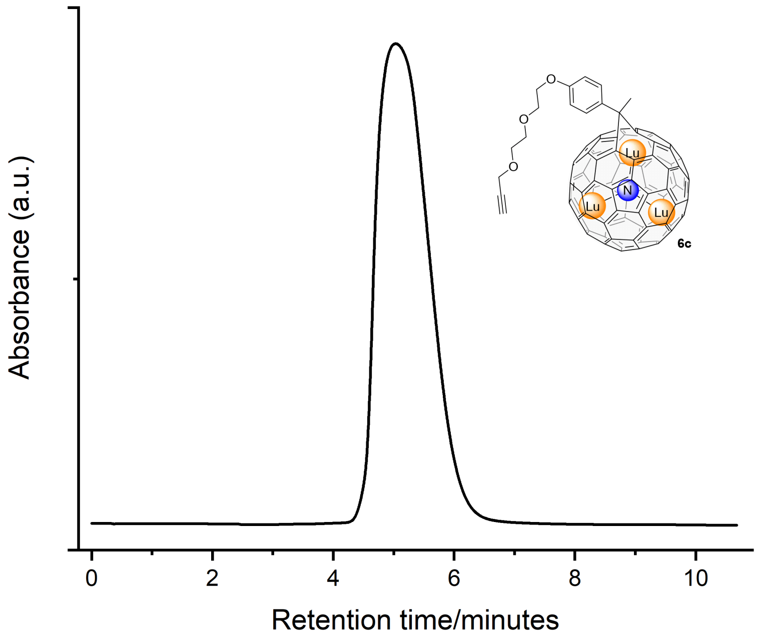

Figure S33. HPLC chromatogram (1.5 mL/minute, toluene : MeOH = 95 : 5, Buckyprep D) of **6c**.

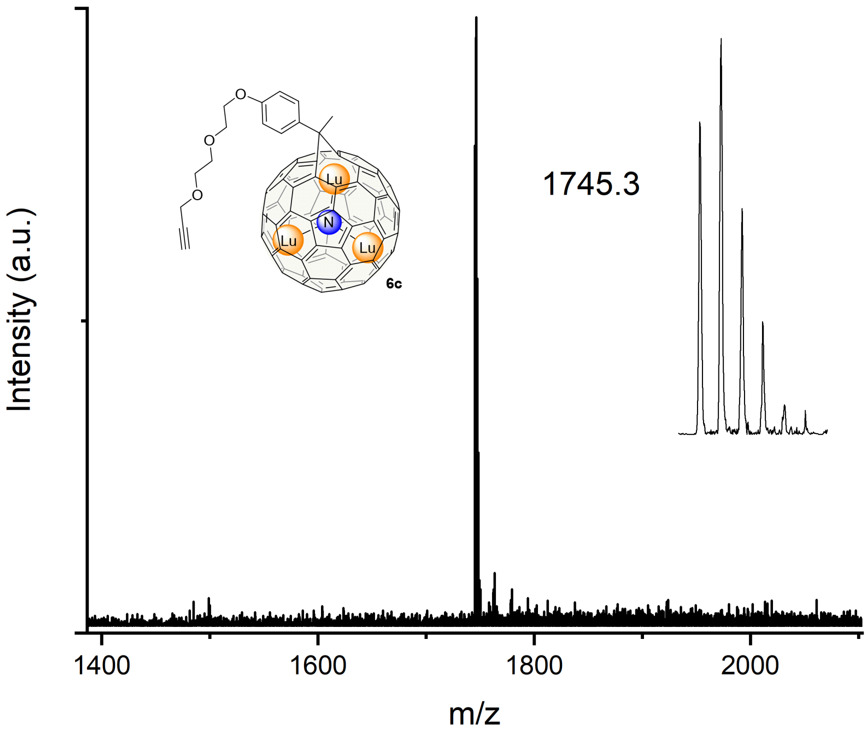

Figure S34. MALDI-TOF mass spectrometry of **6c** (m/z calculated 1745.0, found 1745.4)

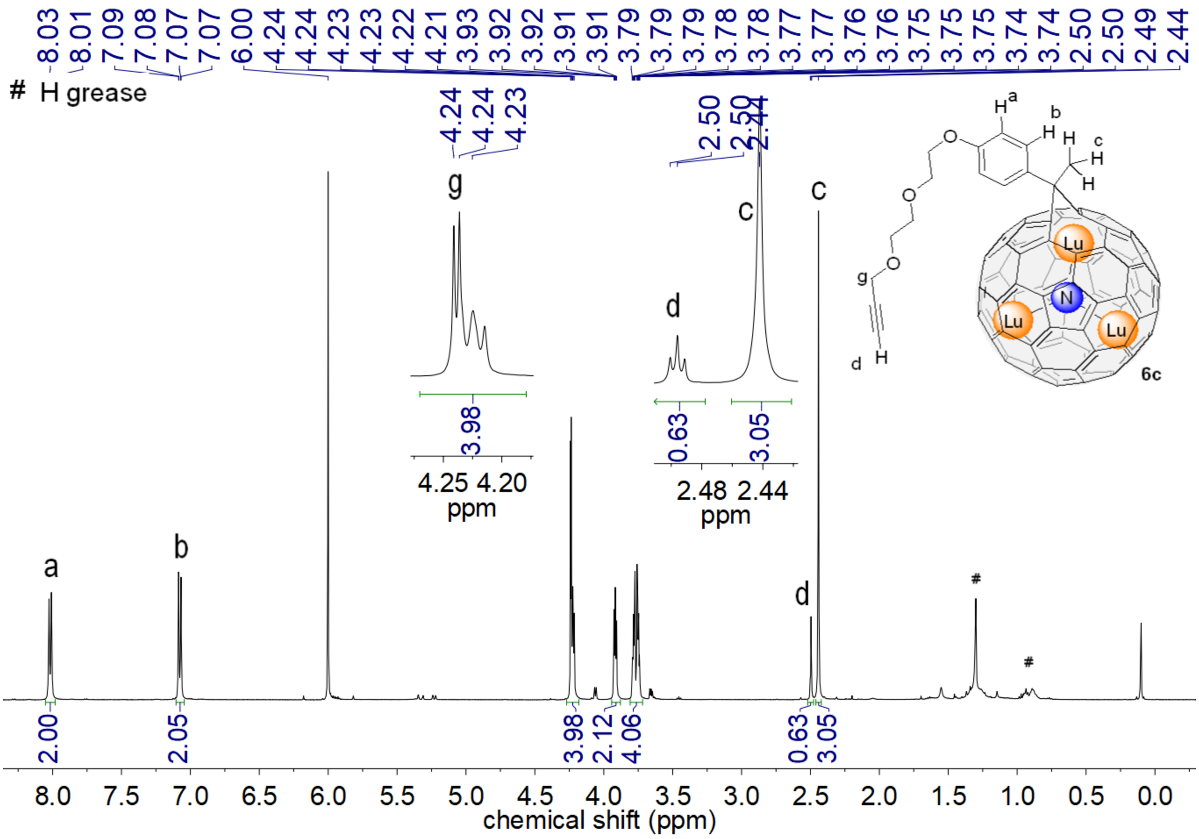

Figure S35. ^{1}H NMR spectrum of **6c**.

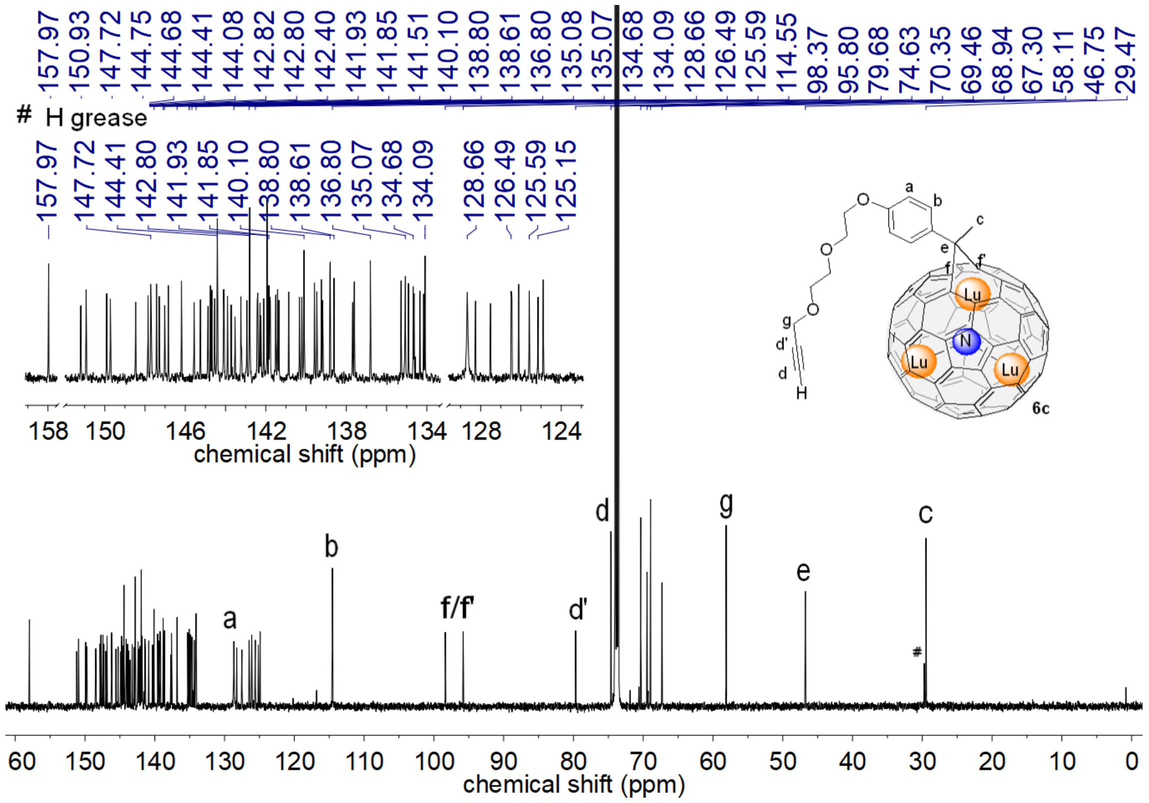

Figure S36. ^{13}C NMR spectrum of **6c**.

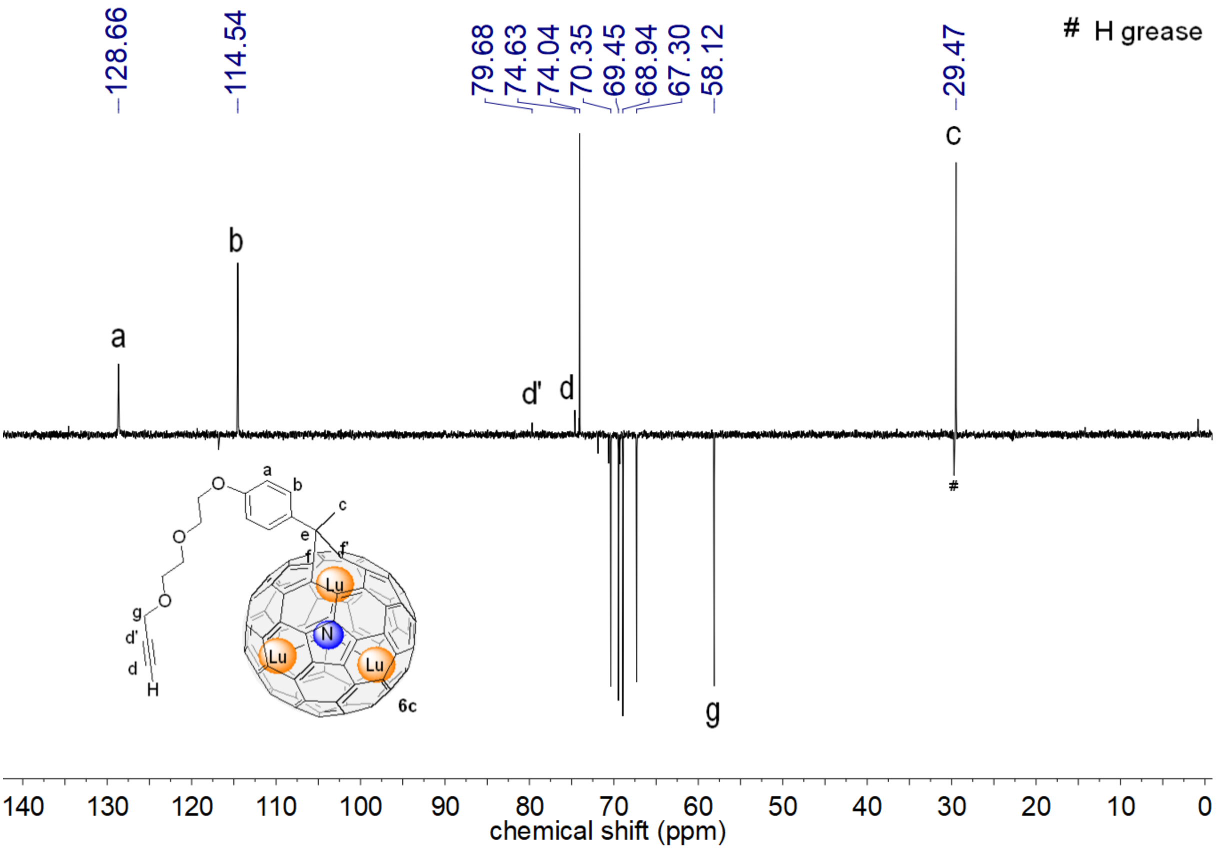

Figure S37. DEPT-135 spectrum of **6c**.

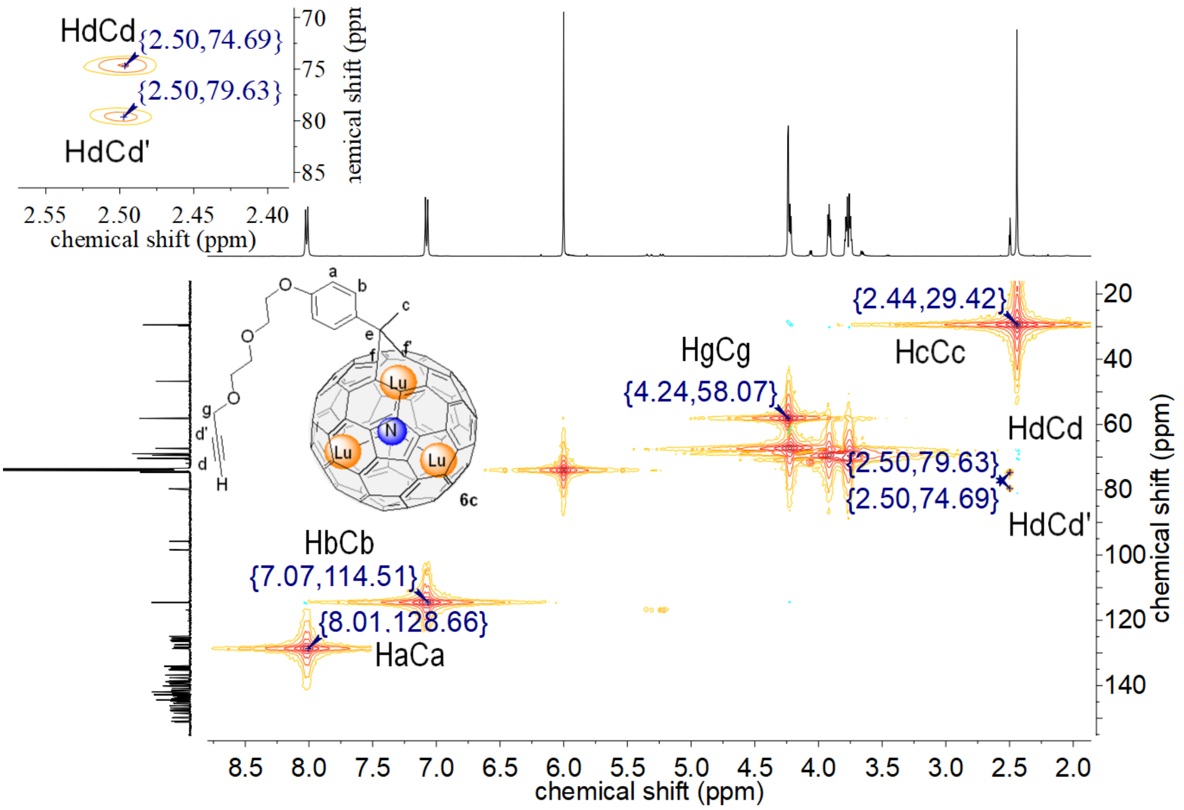

Figure S38. HMQC NMR spectrum of **6c**.

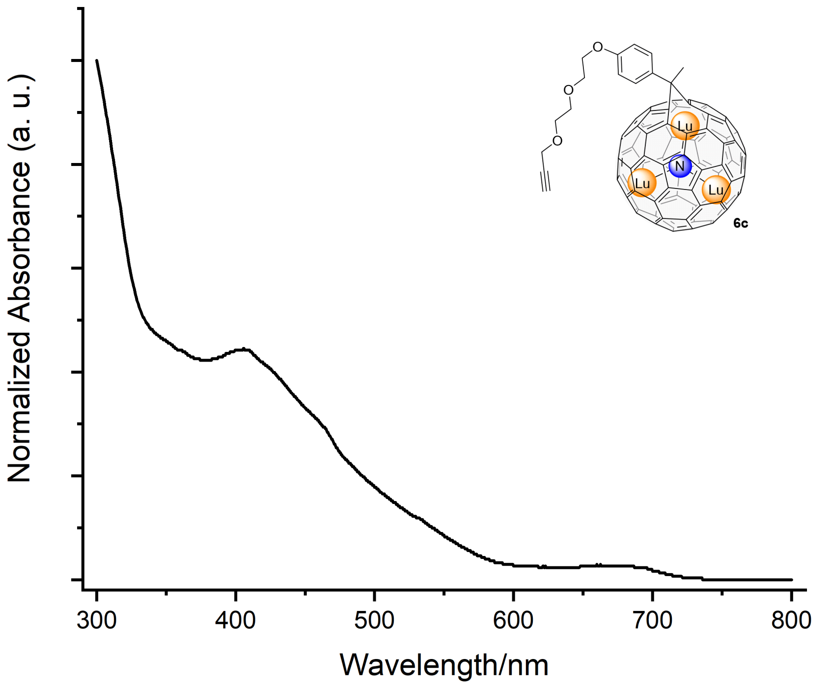

Figure S39. UV-vis spectrum of **6c** in $CHCl_3$.

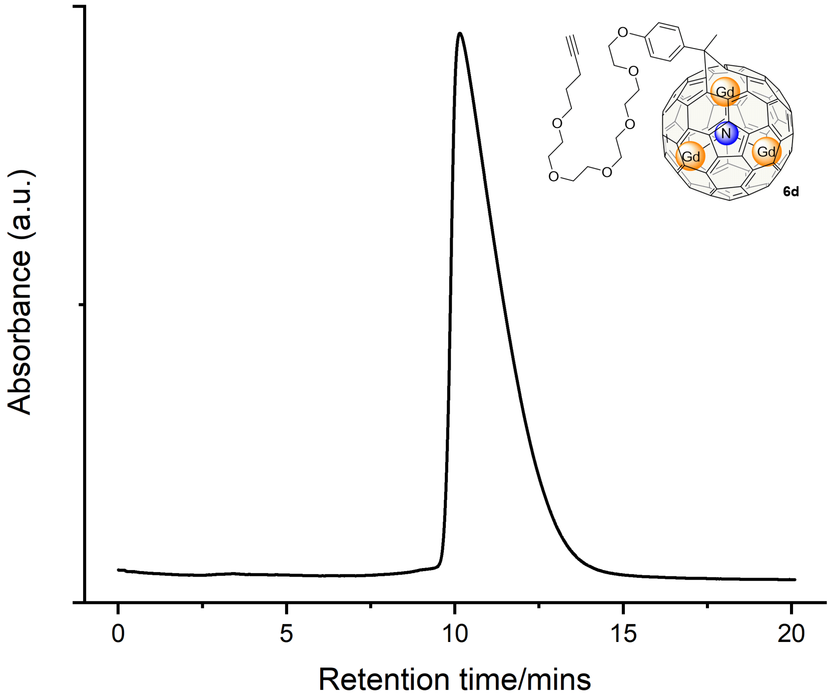

Figure S40. HPLC chromatogram (1.5 mL/minute, toluene : MeOH = 95 : 5, Buckyprep D) of **6d**.

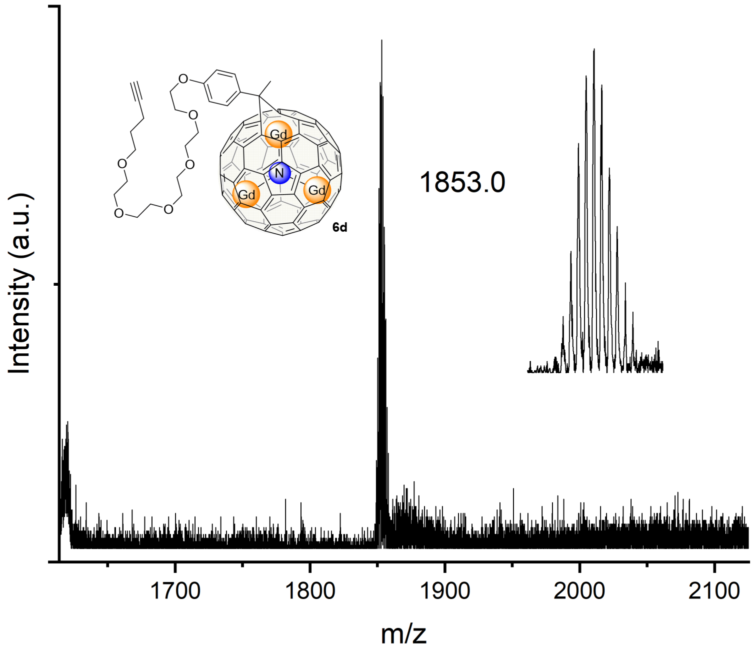

Figure S41. MALDI-TOF mass spectrometry of **6d** (m/z calculated 1853.2, found 1853.0)

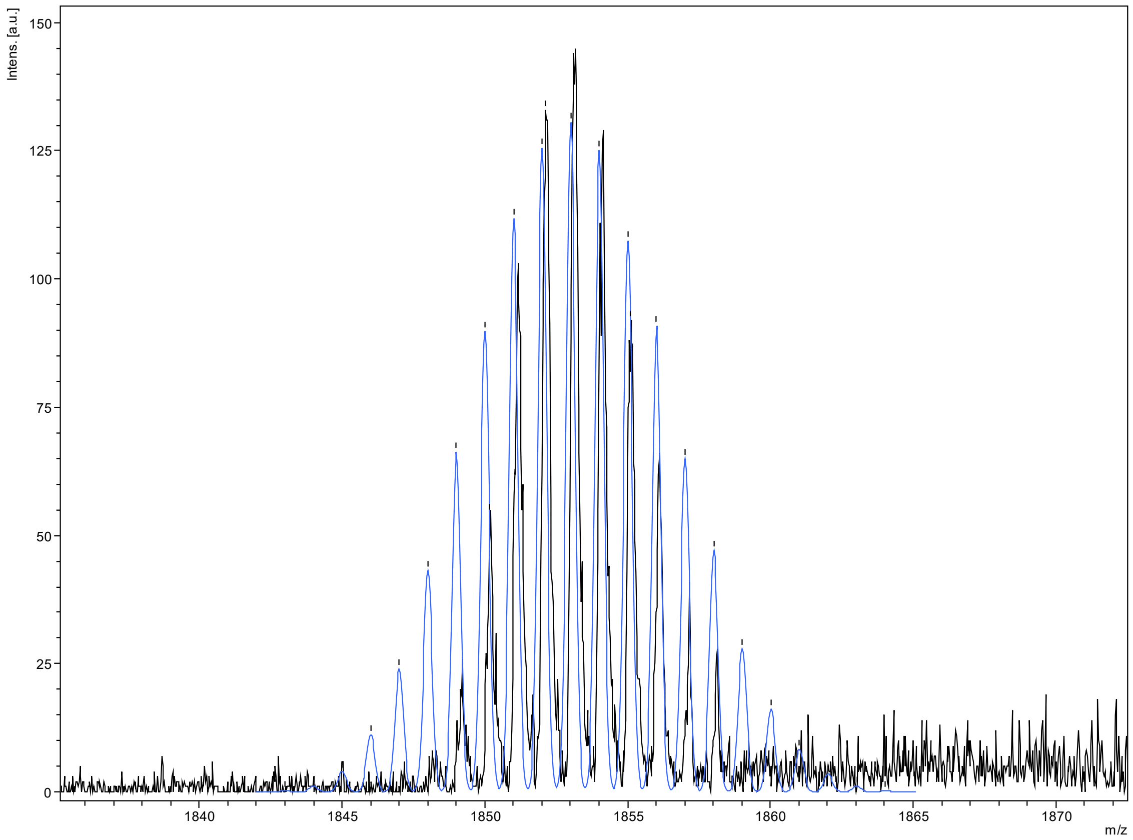

Figure S42. Calculated (blue) vs experimental (black) MALDI-TOF MS of of **6d**.

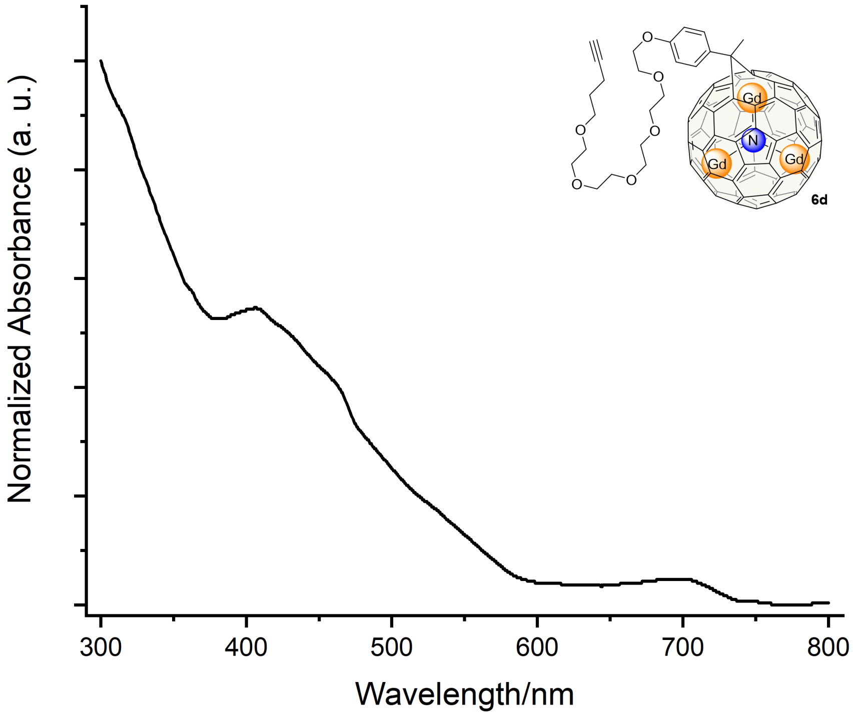

Figure S43. UV-vis spectrum of **6d** in $CHCl_3$.

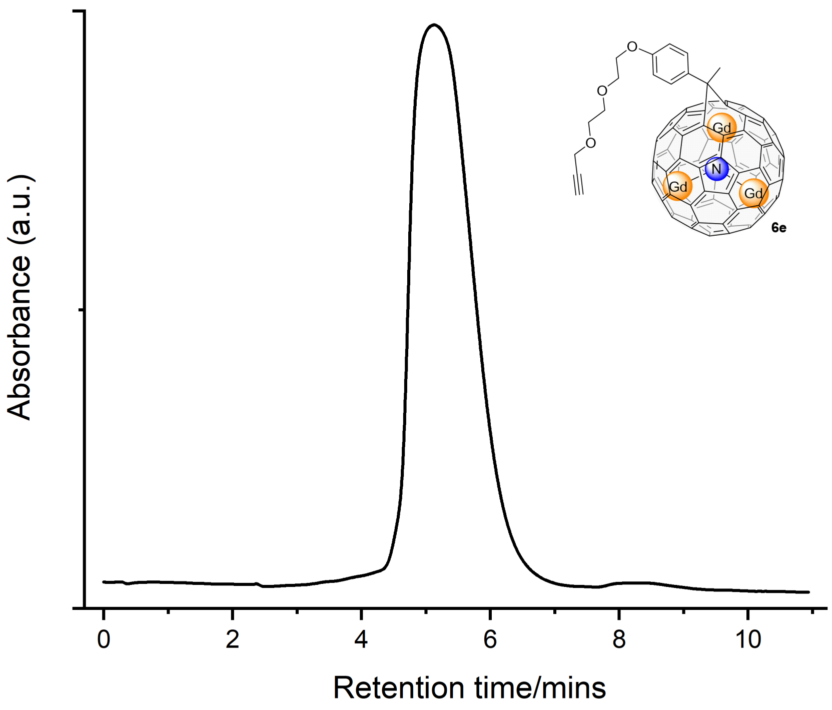

Figure S44. HPLC chromatogram (1.5 mL/minute, toluene : MeOH = 95 : 5, Buckyprep D) of **6e**.

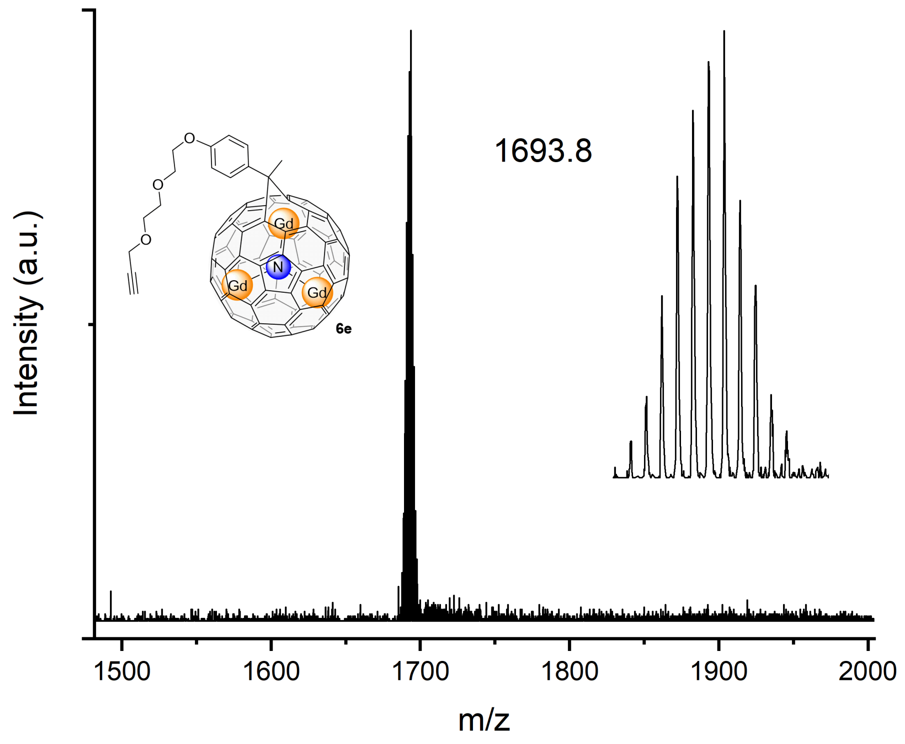

Figure S45. MALDI-TOF mass spectrometry of **6e** (m/z calculated 1693.9, found 1693.8)

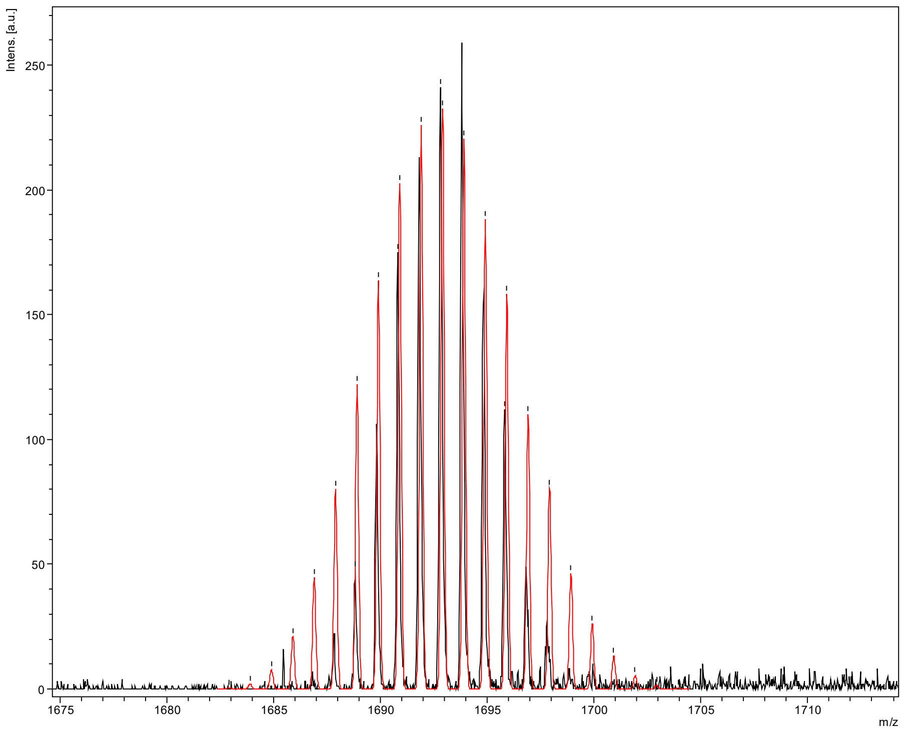

Figure S46. Calculated (red) vs experimental (black) MALDI-TOF MS of of **6e**.

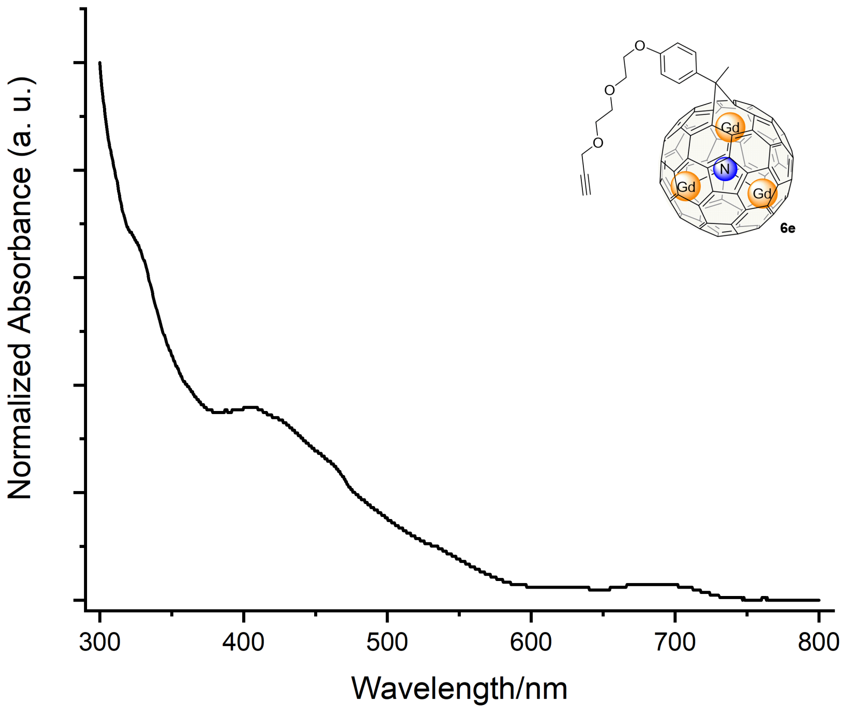

Figure S47. UV-vis spectrum of **6e** in $CHCl_3$.

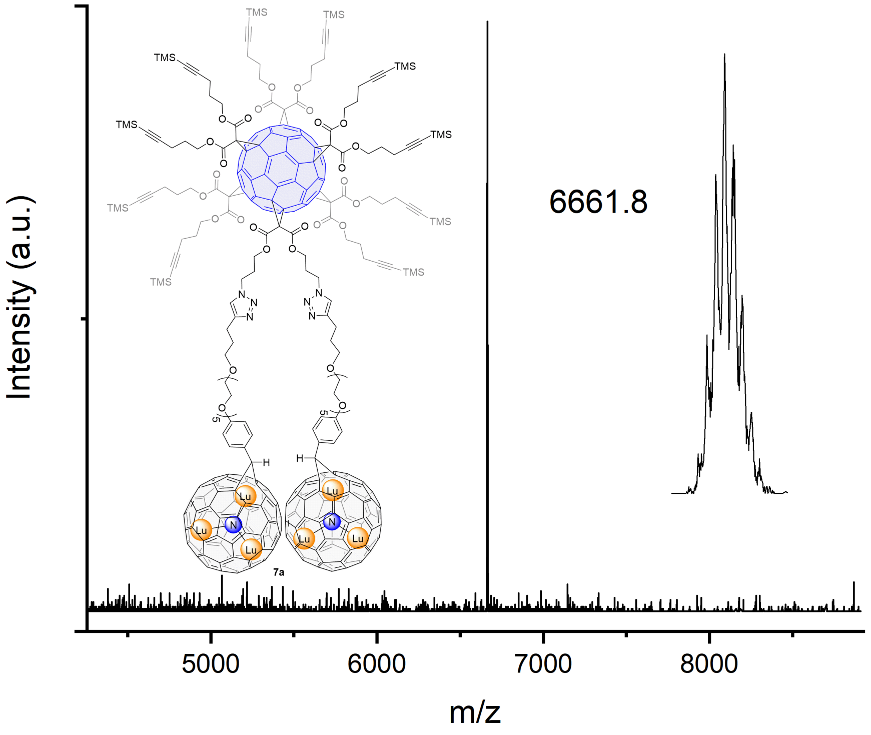

Figure S48. MALDI-TOF mass spectrometry of **7a** (average m/z calculated 6662.0, found 6661.8)

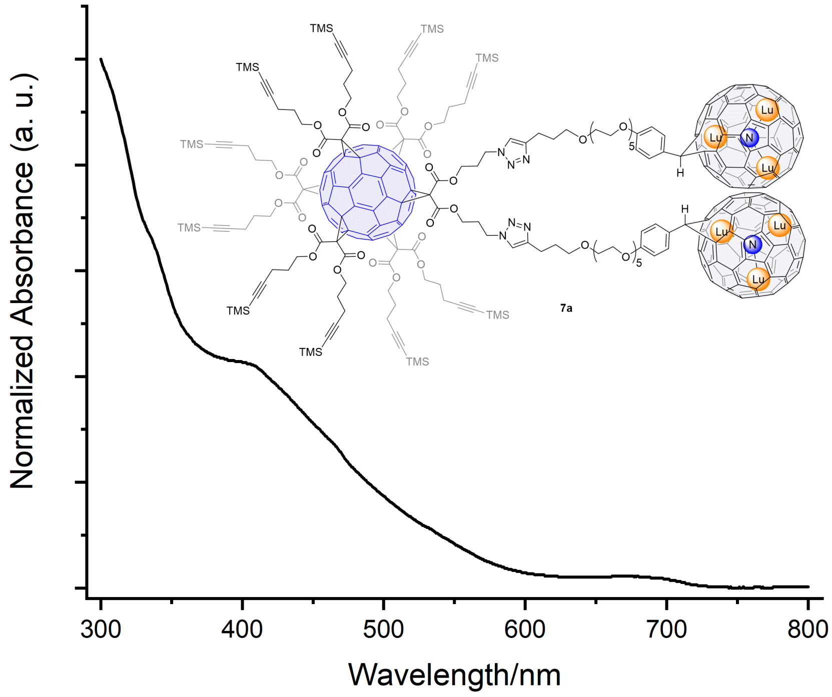

Figure S49. UV-vis spectrum of **7a** in $CHCl_3$.

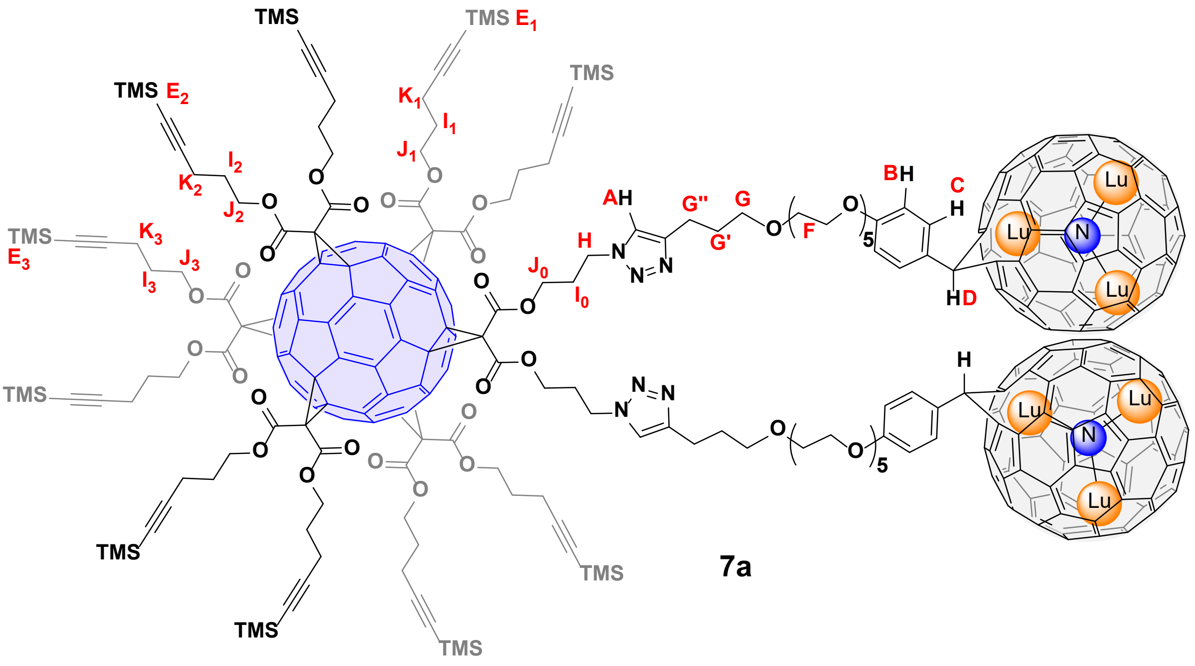

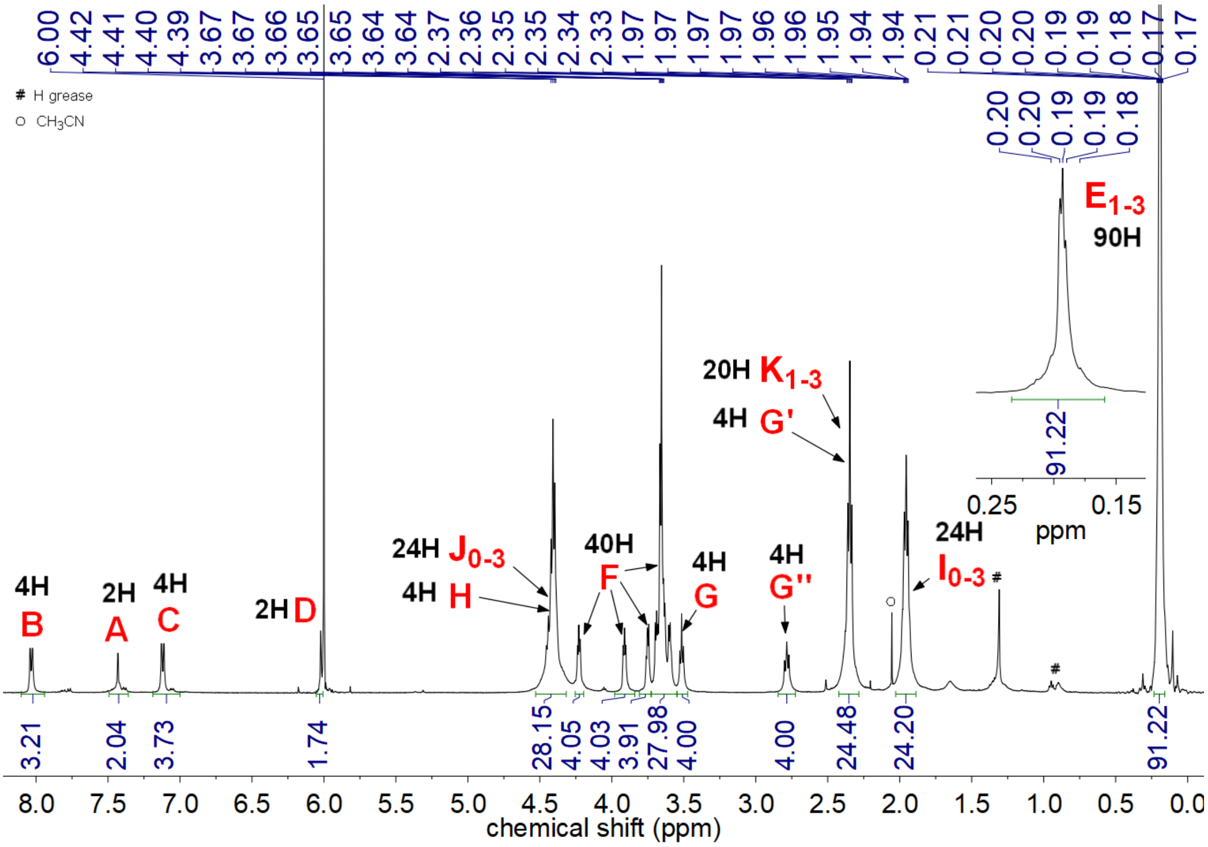

Figure S50. ^{1}H NMR spectrum of **7a**.

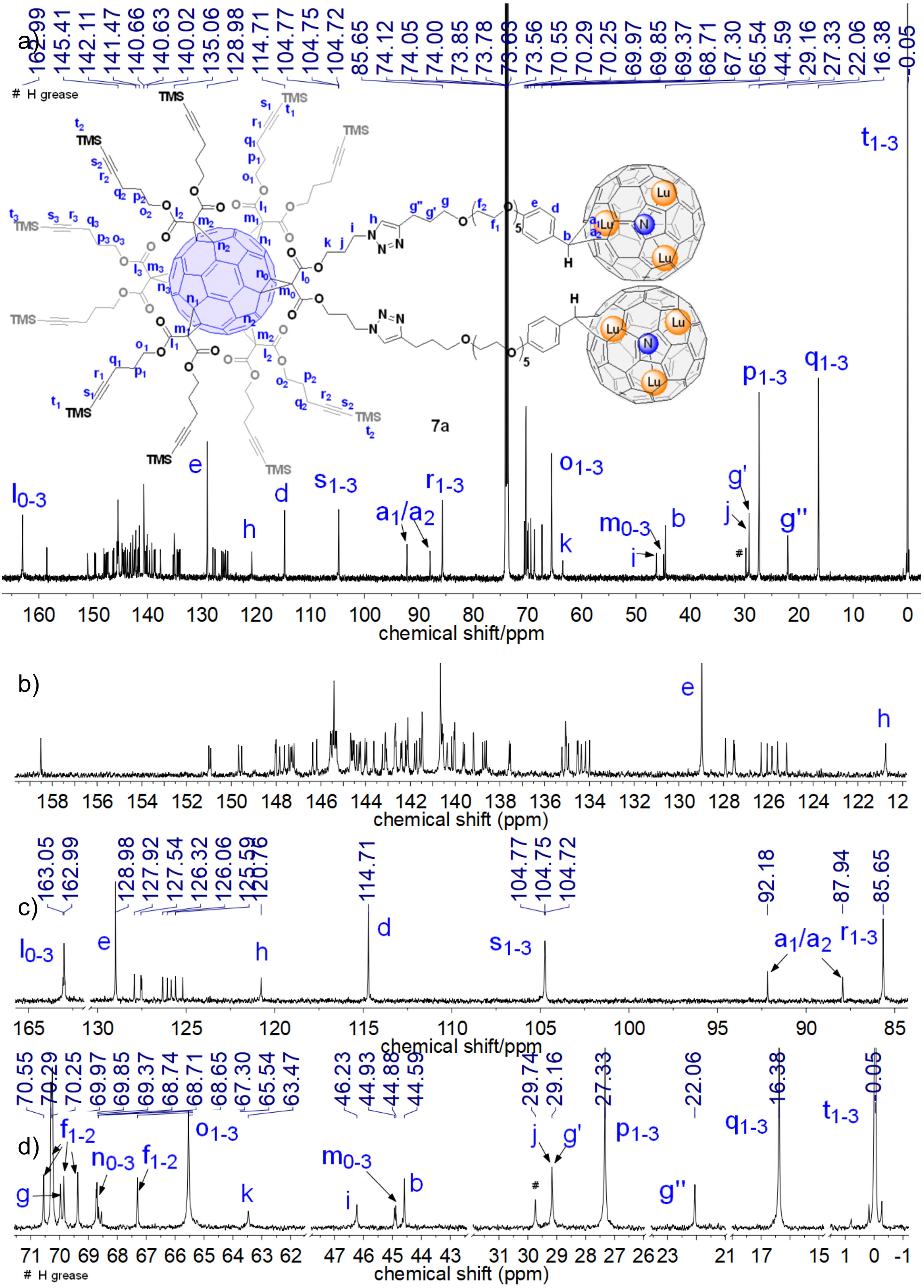

Figure S51. a) full ^{13}C NMR spectrum of **7a**; b) sp^2 carbon region from 120 to 160 ppm of **7a**; c) selected special carbon signals from 85 to 165 ppm of **7a**; d) selected special carbon signals from 0 to 71 ppm of **7a**.

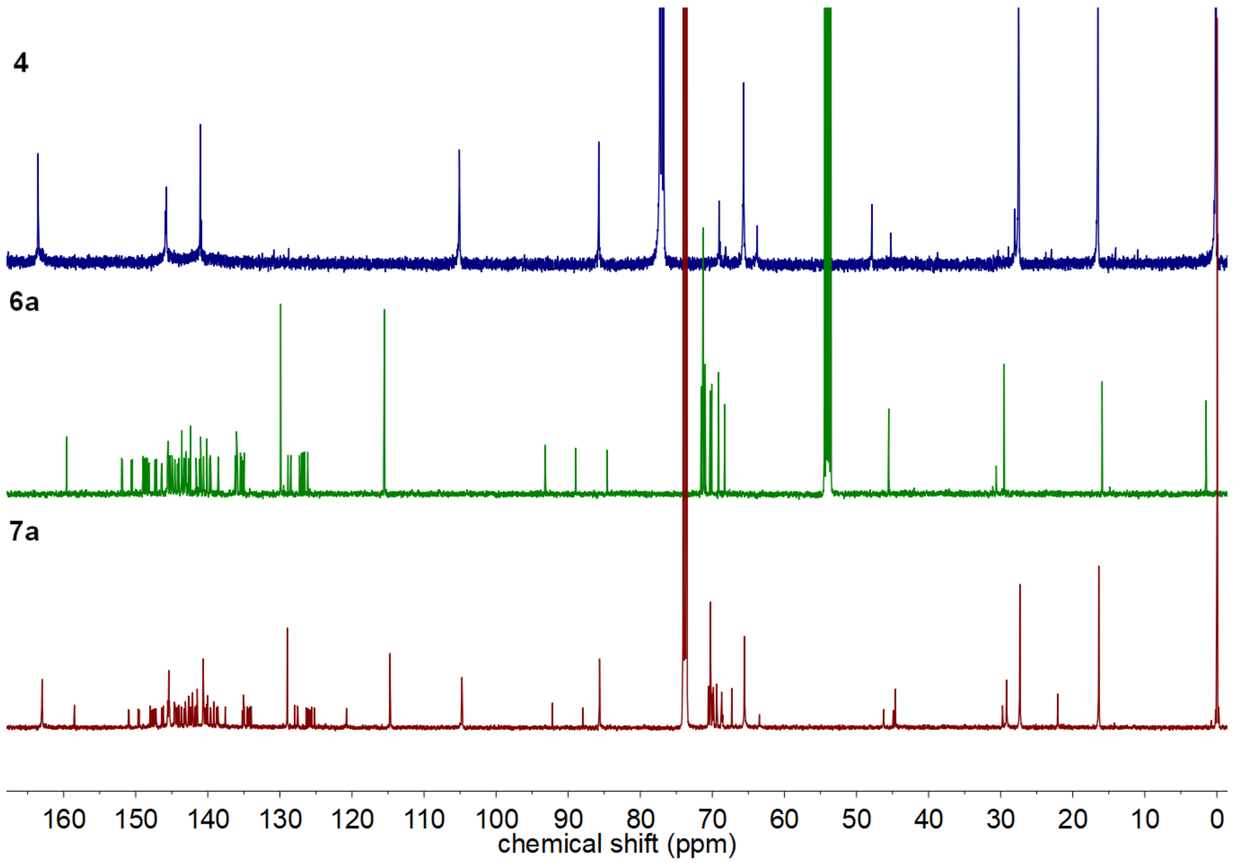

Figure S52. Comparision of ^{13}C NMR spectra of **4**, **6a** and **7a**

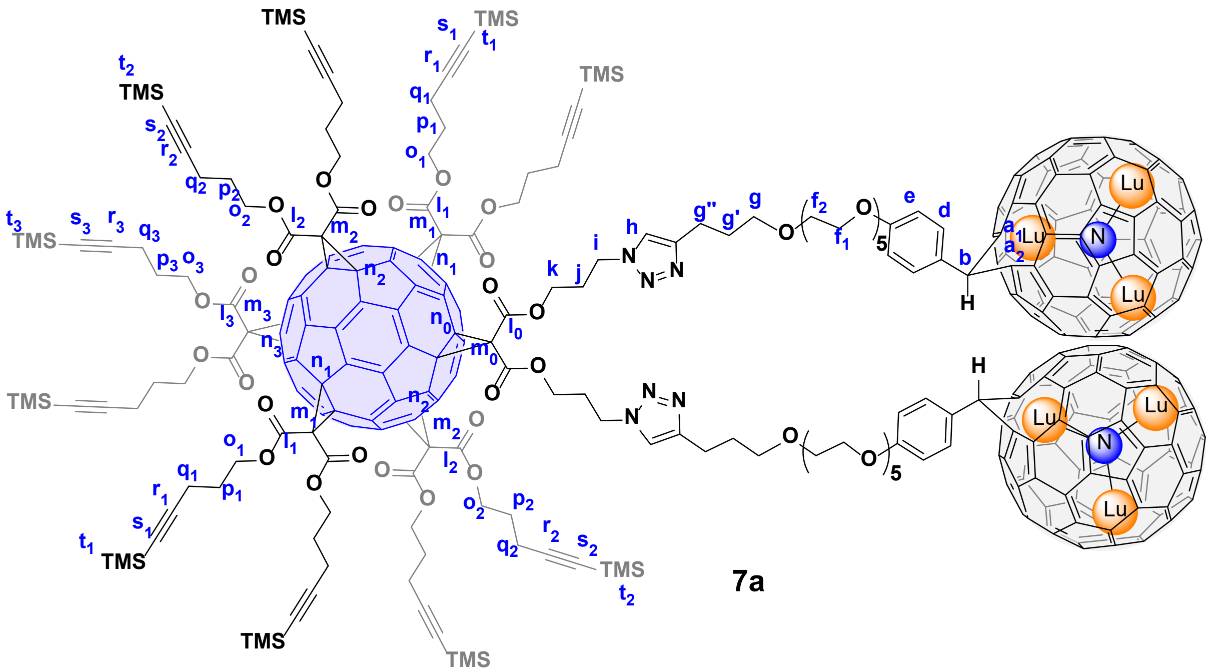

7a

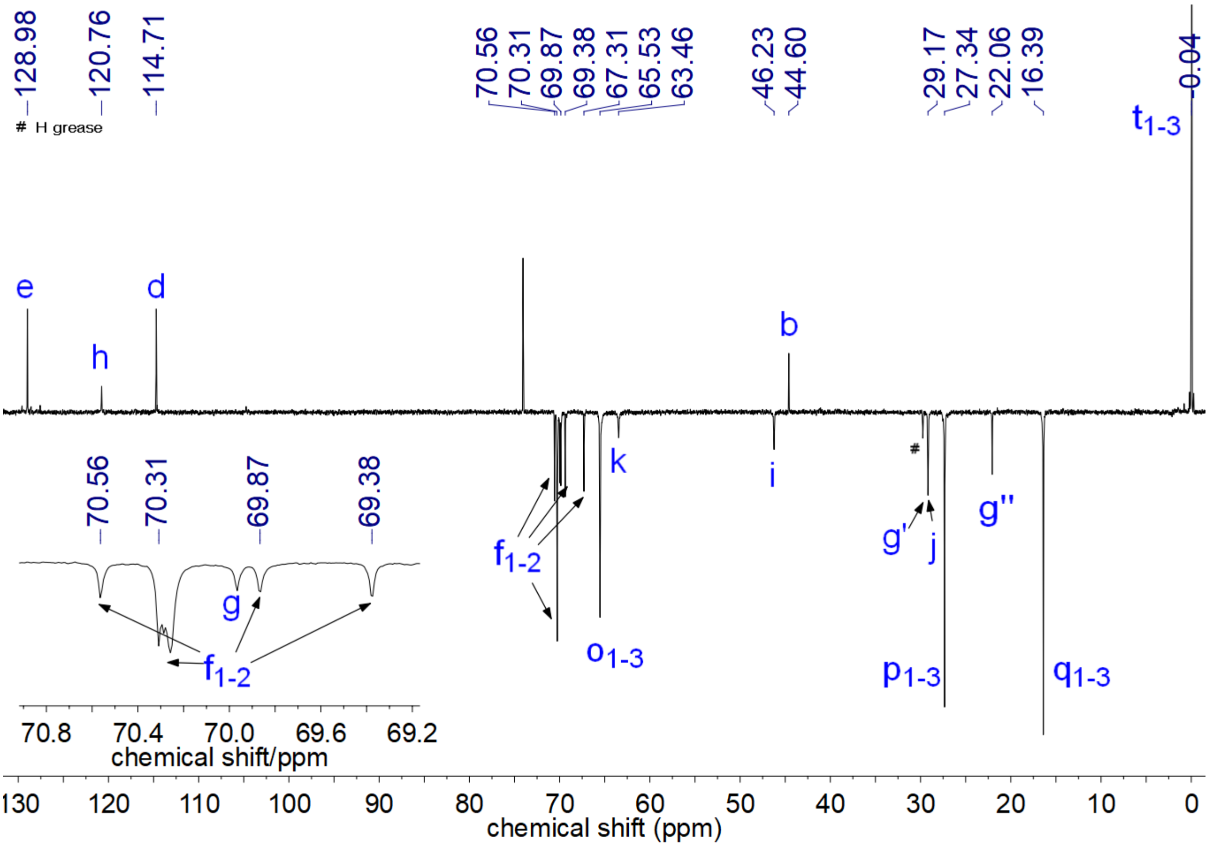

Figure S53. DEPT-135 spectrum of **7a**.

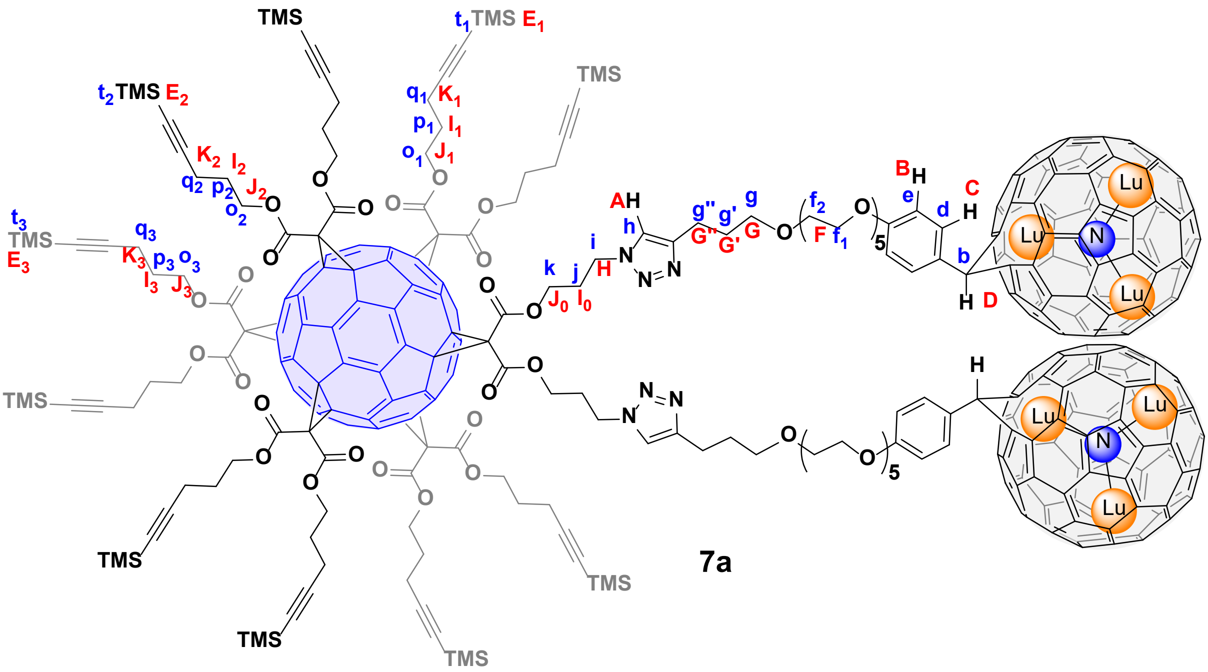

7a

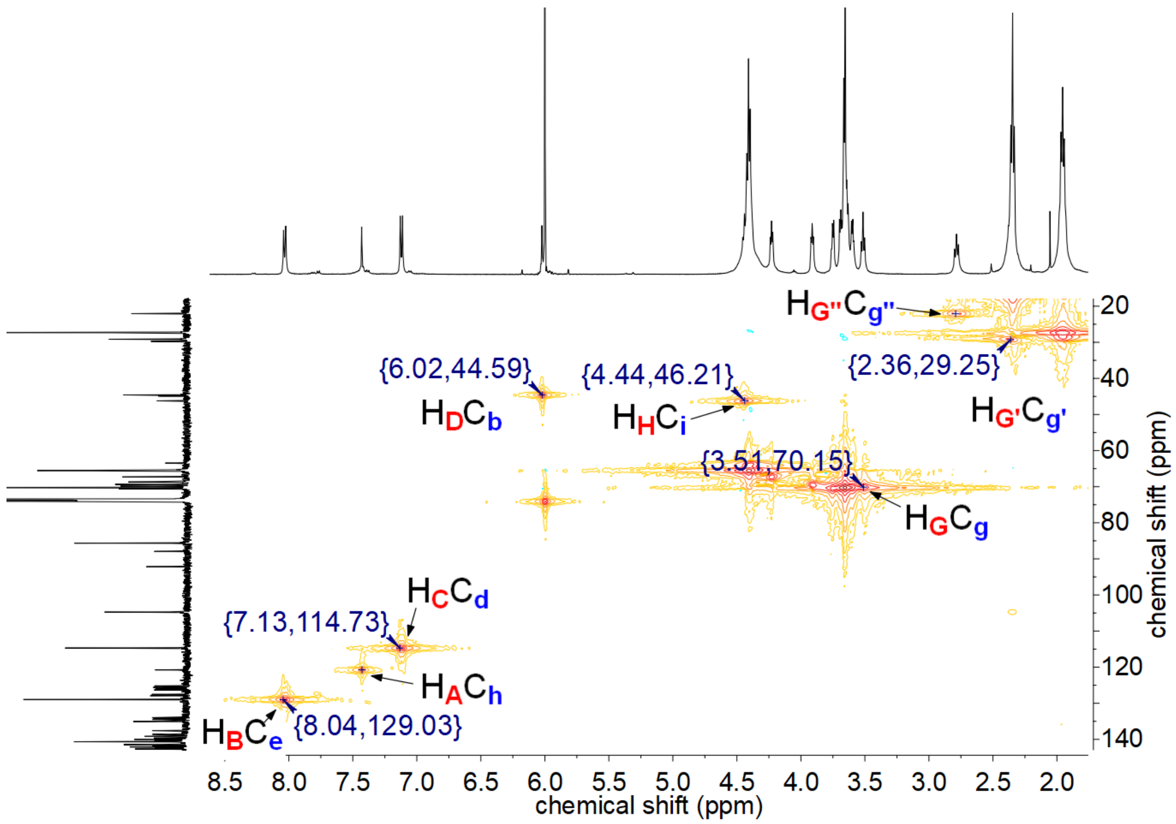

Figure S54. HMQC NMR spectrum of **7a**.

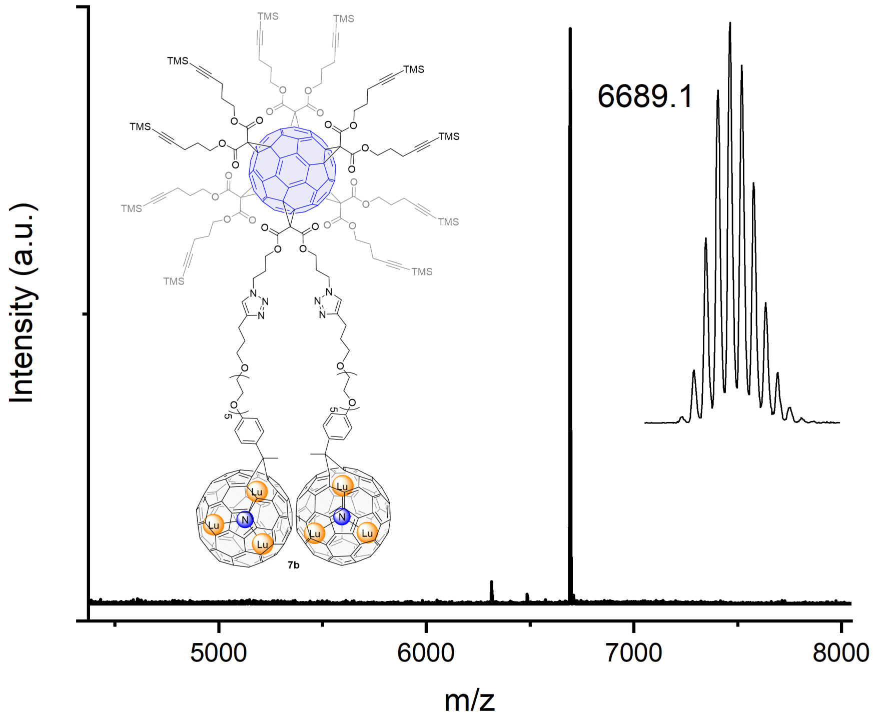

Figure S55. MALDI-TOF mass spectrometry of **7b** (average m/z calculated 6689.1, found 6689.1)

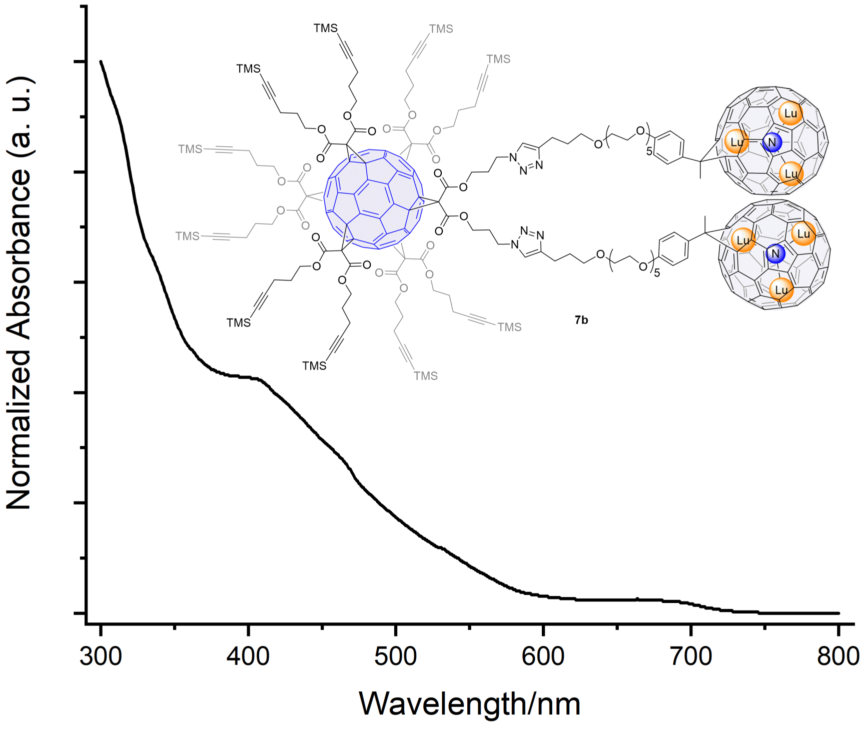

Figure S56. UV-vis spectrum of **7b** in $CHCl_3$.

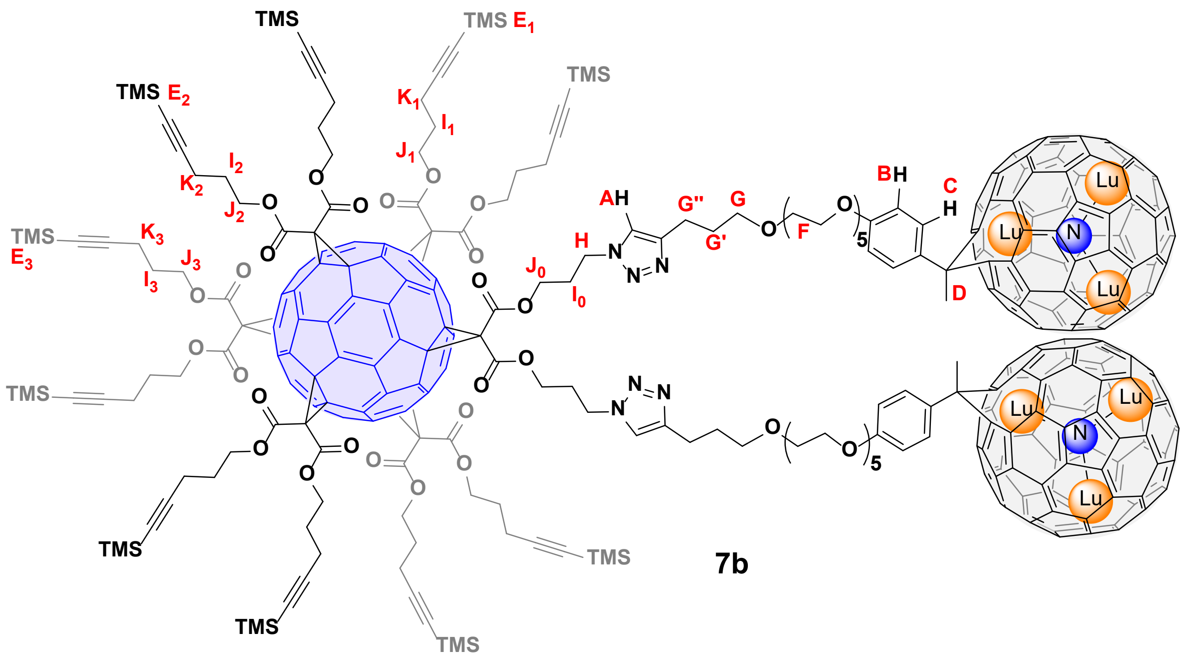

7b

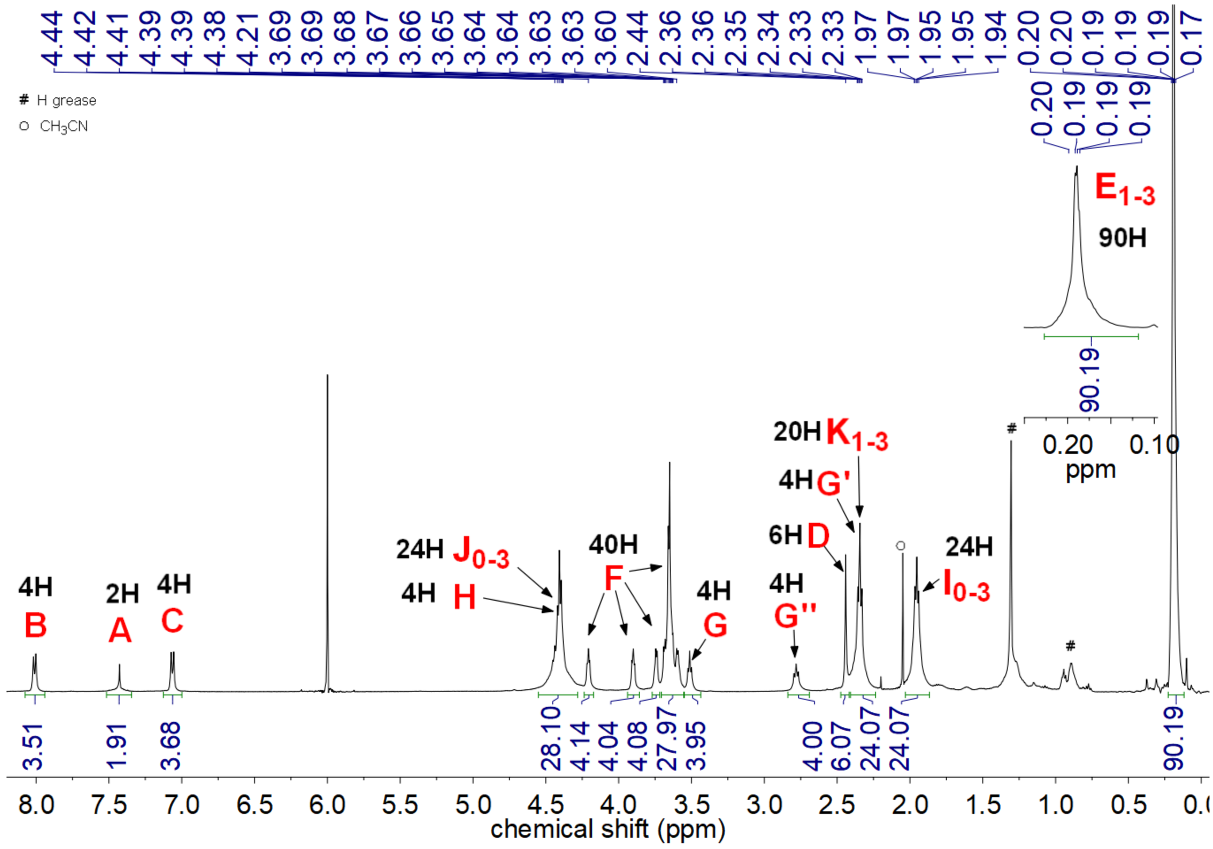

Figure S57. ^{1}H NMR spectrum of **7b**.

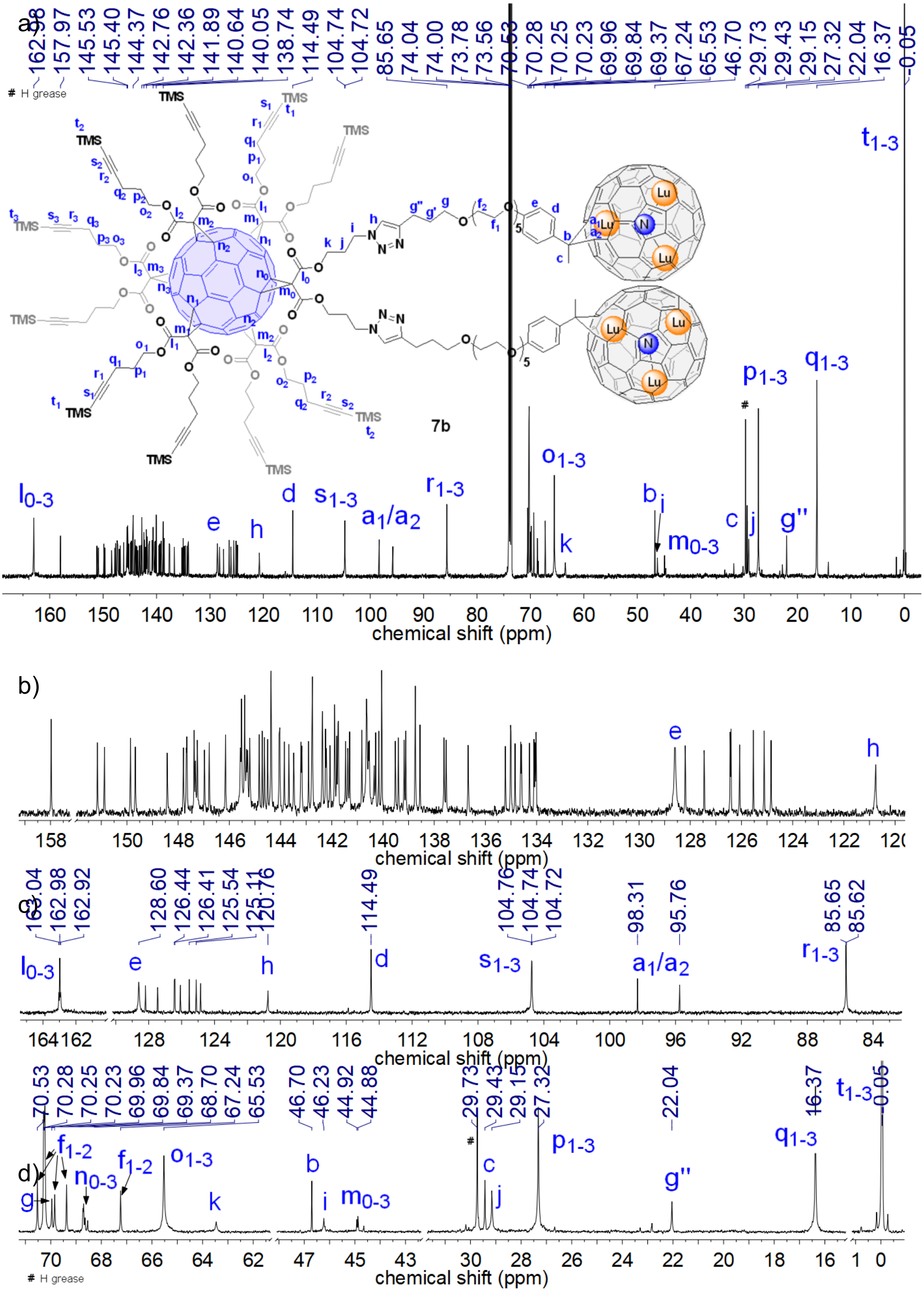

Figure S58. a) full ^{13}C NMR spectrum of **7b**; b) sp^2 carbon region from 120 to 160 ppm of **7b**; c) selected special carbon signals from 85 to 165 ppm of **7b**; d) selected special carbon signals from 0 to 71 ppm of **7b**.

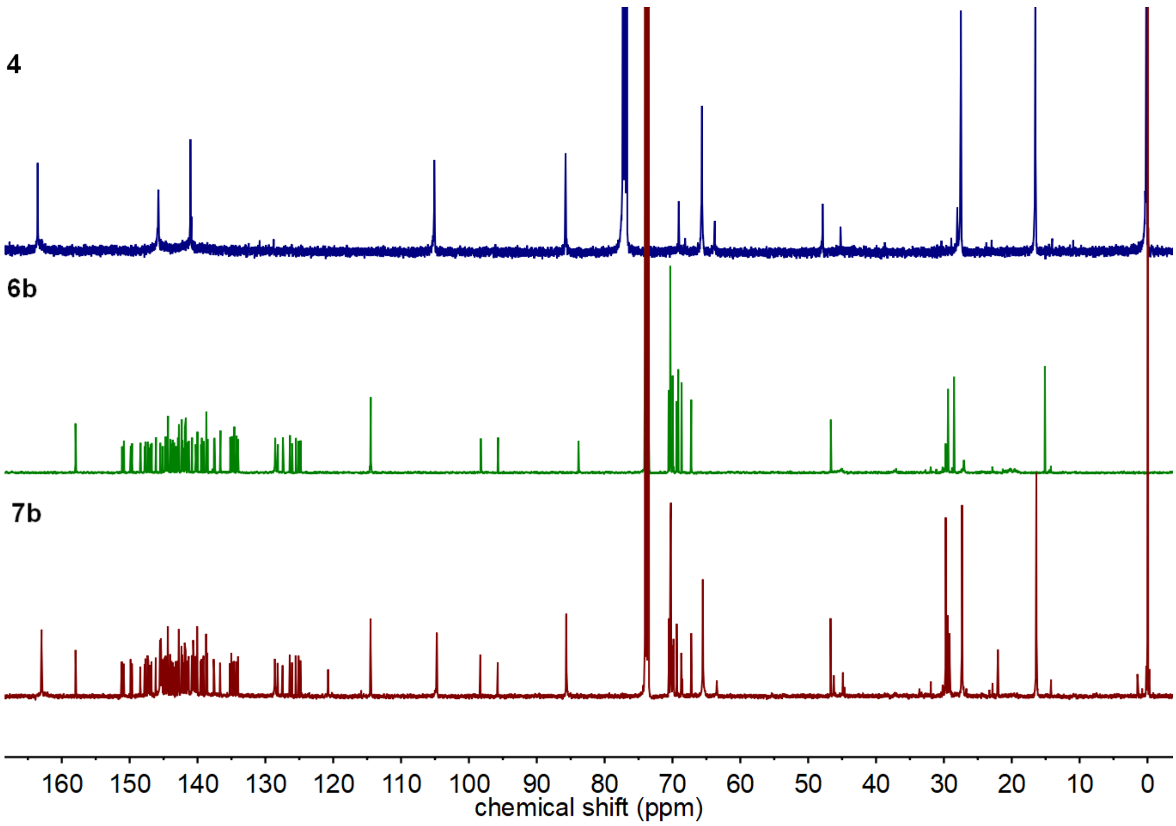

Figure S59. Comparision of ^{13}C NMR spectra of **4**, **6b** and **7b**

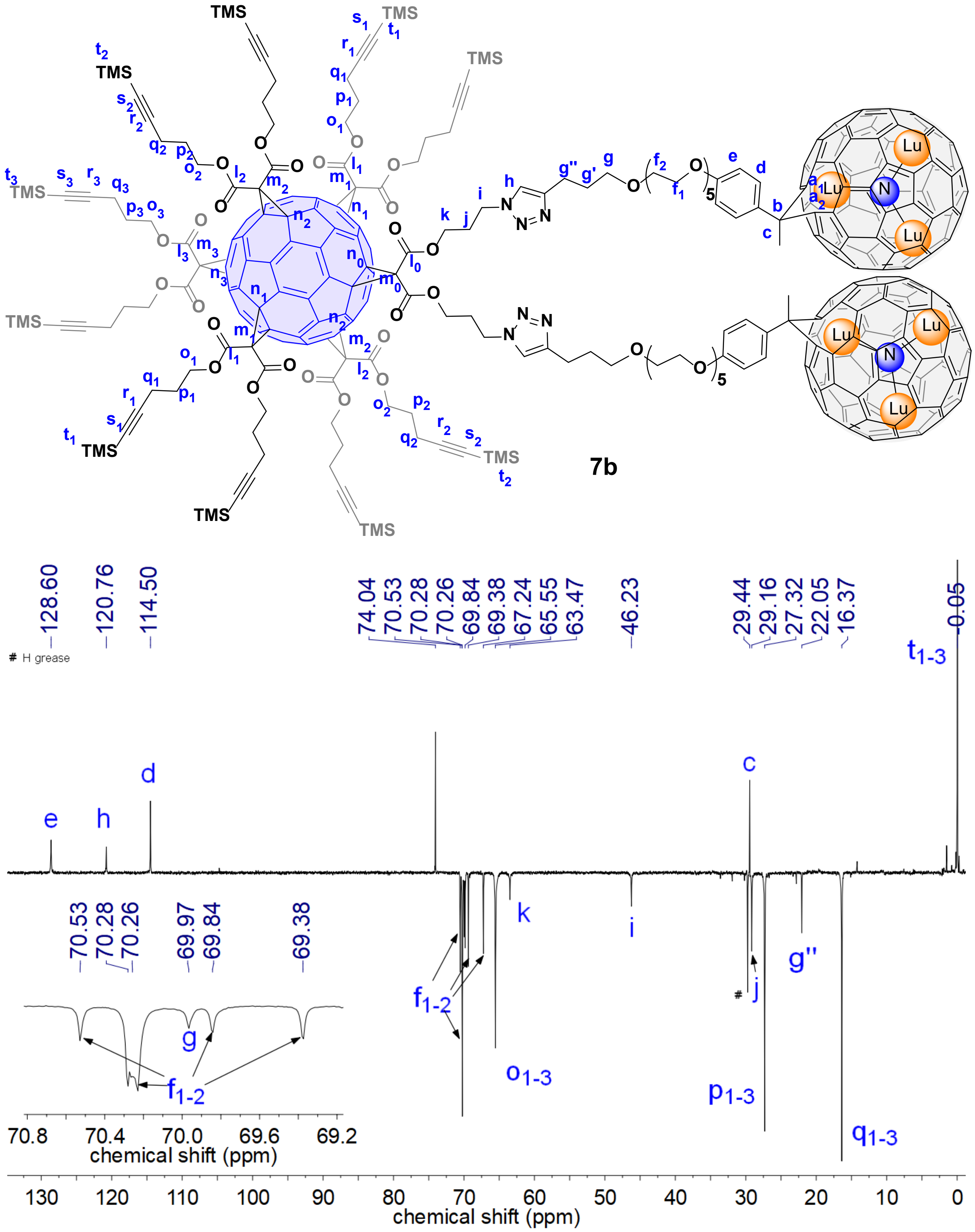

Figure S60. DEPT-135 spectrum of **7b**.

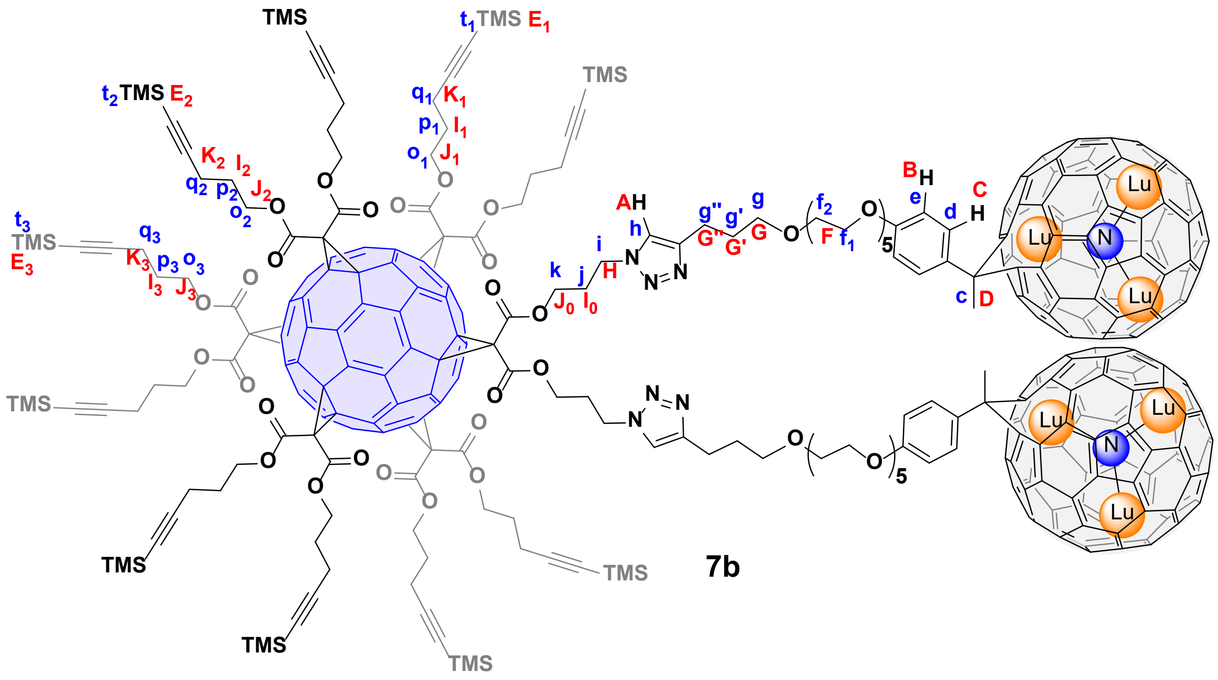

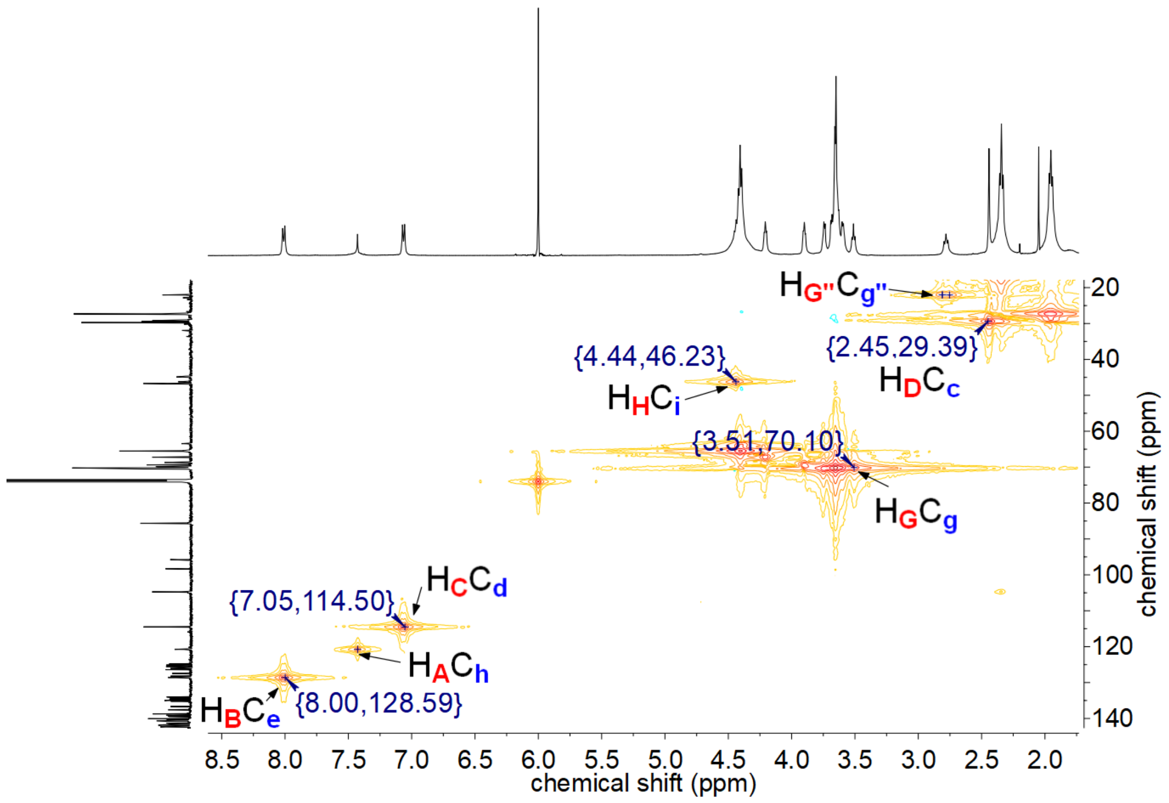

Figure S61. HMQC NMR spectrum of **7b**.

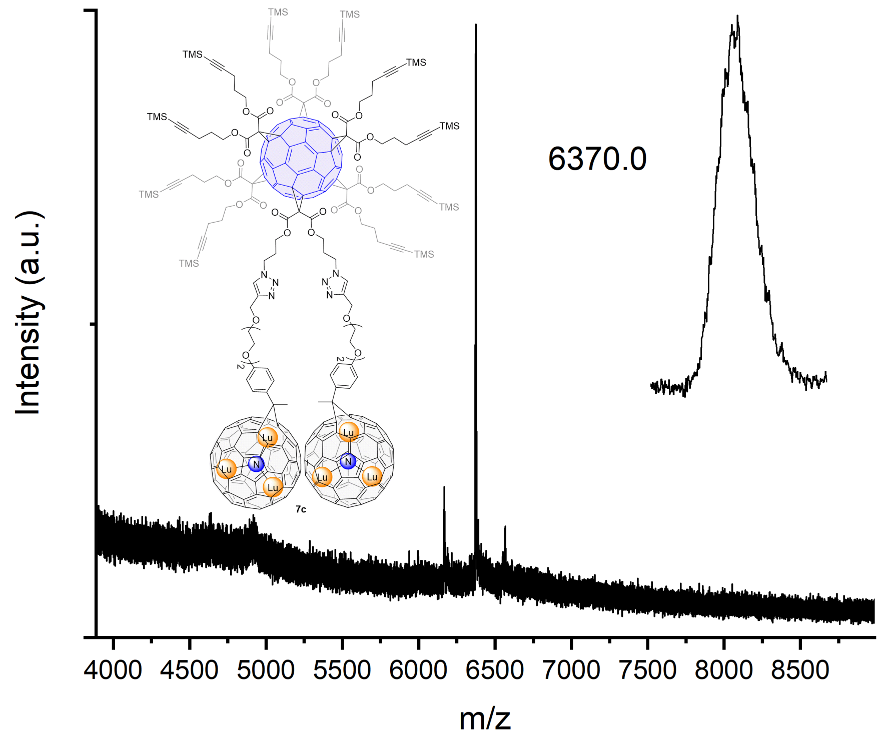

Figure S62. MALDI-TOF mass spectrometry of **7c** (average m/z calculated 6369.8, found 6370.0)

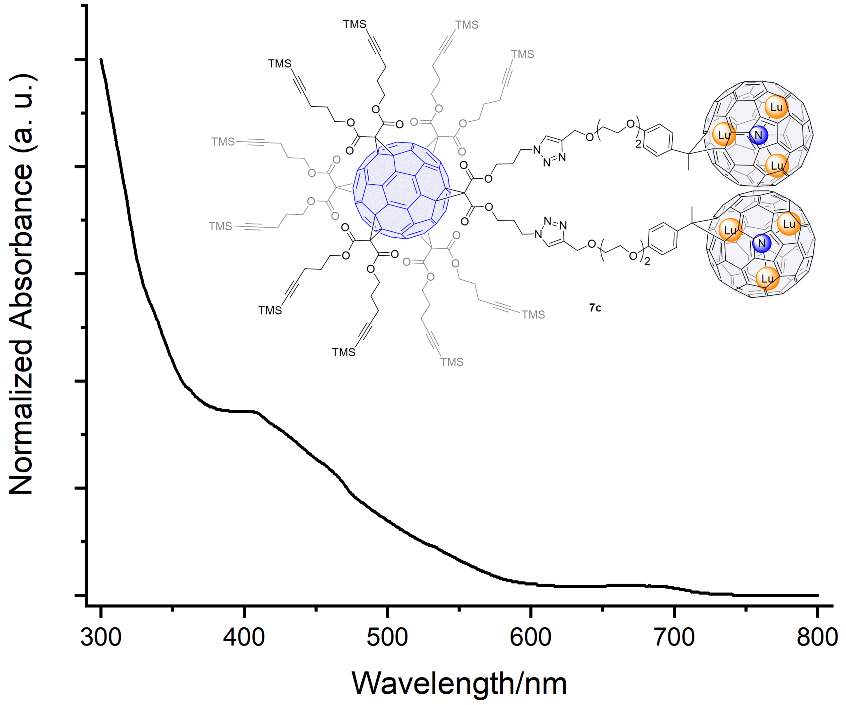

Figure S63. UV-vis spectrum of **7c** in $CHCl_3$.

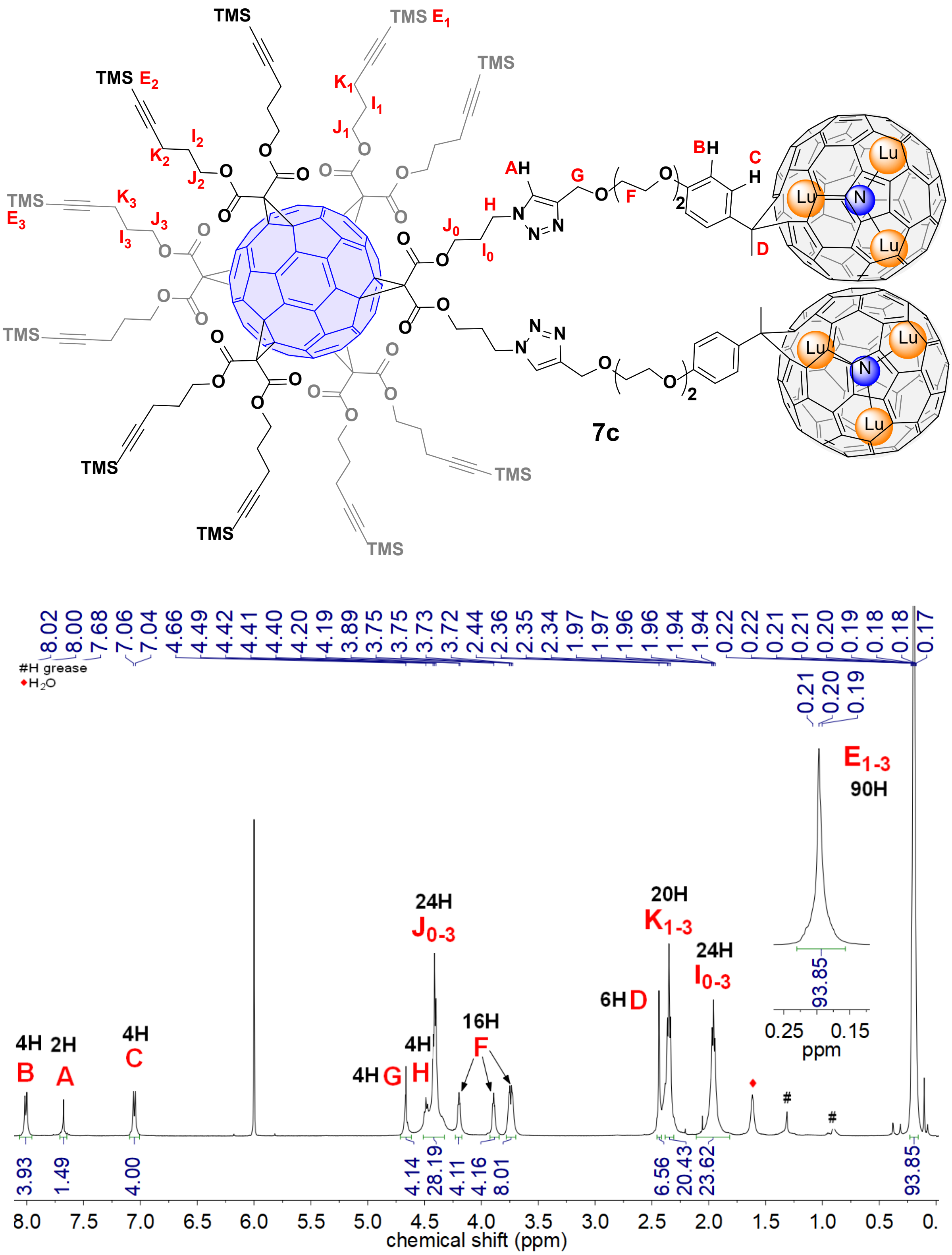

Figure S64. ^{1}H NMR spectrum of **7c**.

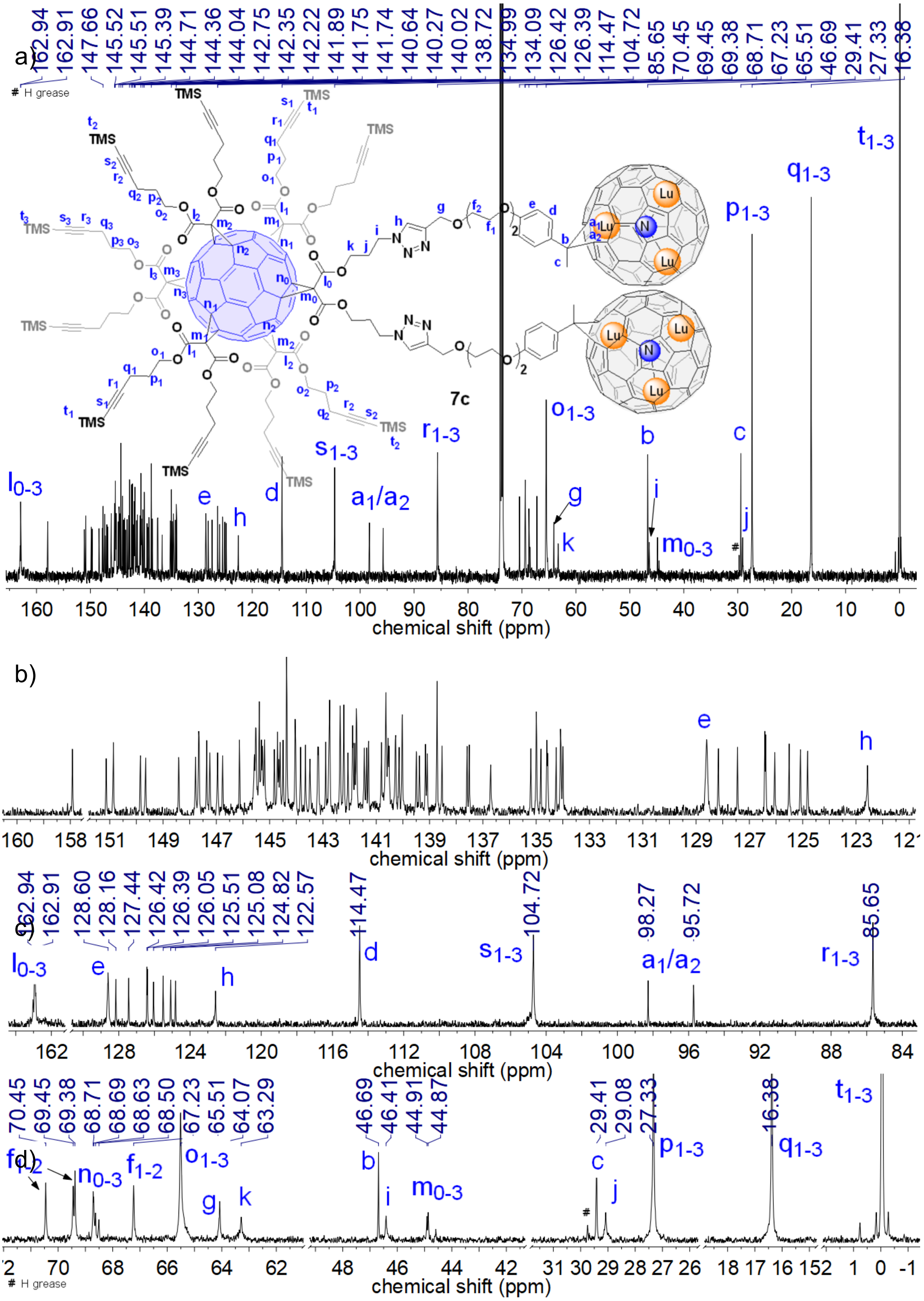

Figure S65. a) full ^{13}C NMR spectrum of **7c**; b) sp^{2} carbon region from 120 to 160 ppm of **7c**; c) selected special carbon signals from 85 to 165 ppm of **7c**; d) selected special carbon signals from 0 to 71 ppm of **7c**.

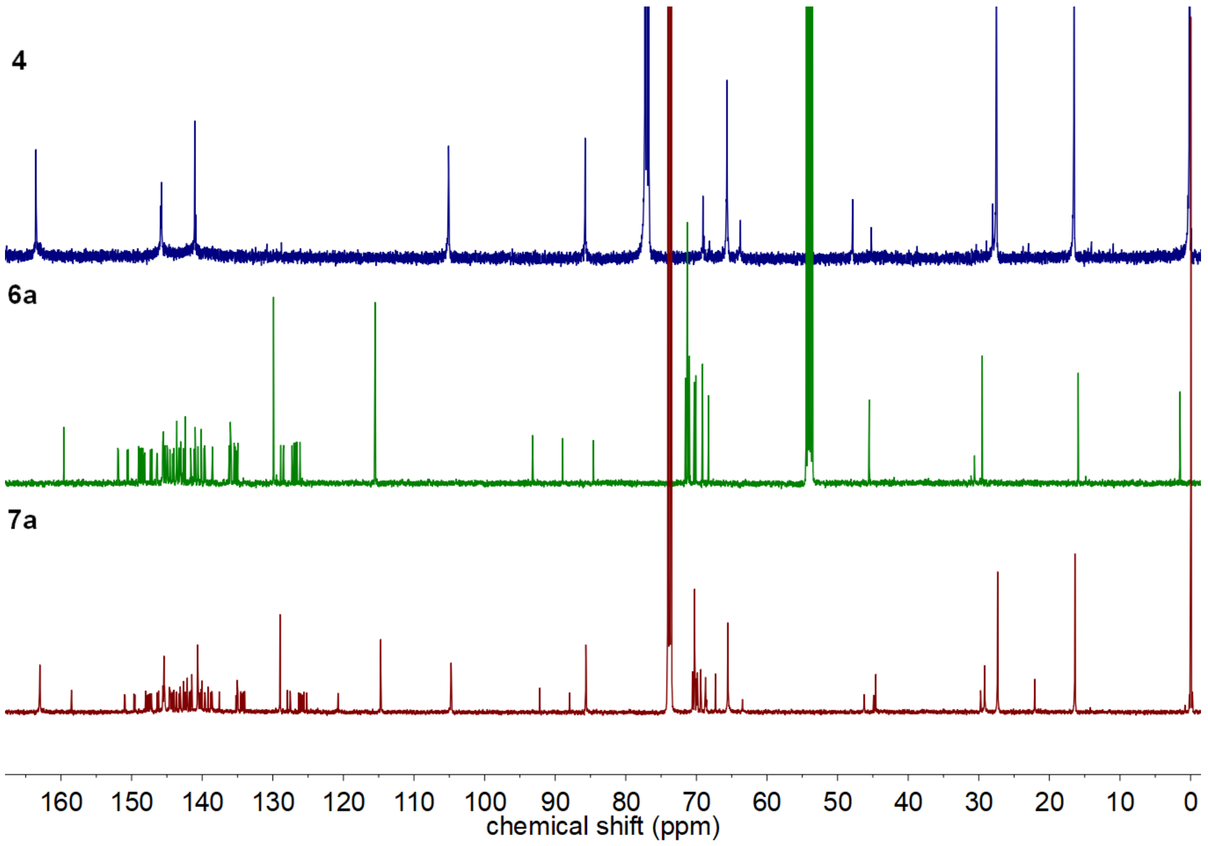

Figure S66. Comparision of ^{13}C NMR spectra of **4**, **6c** and **7c**

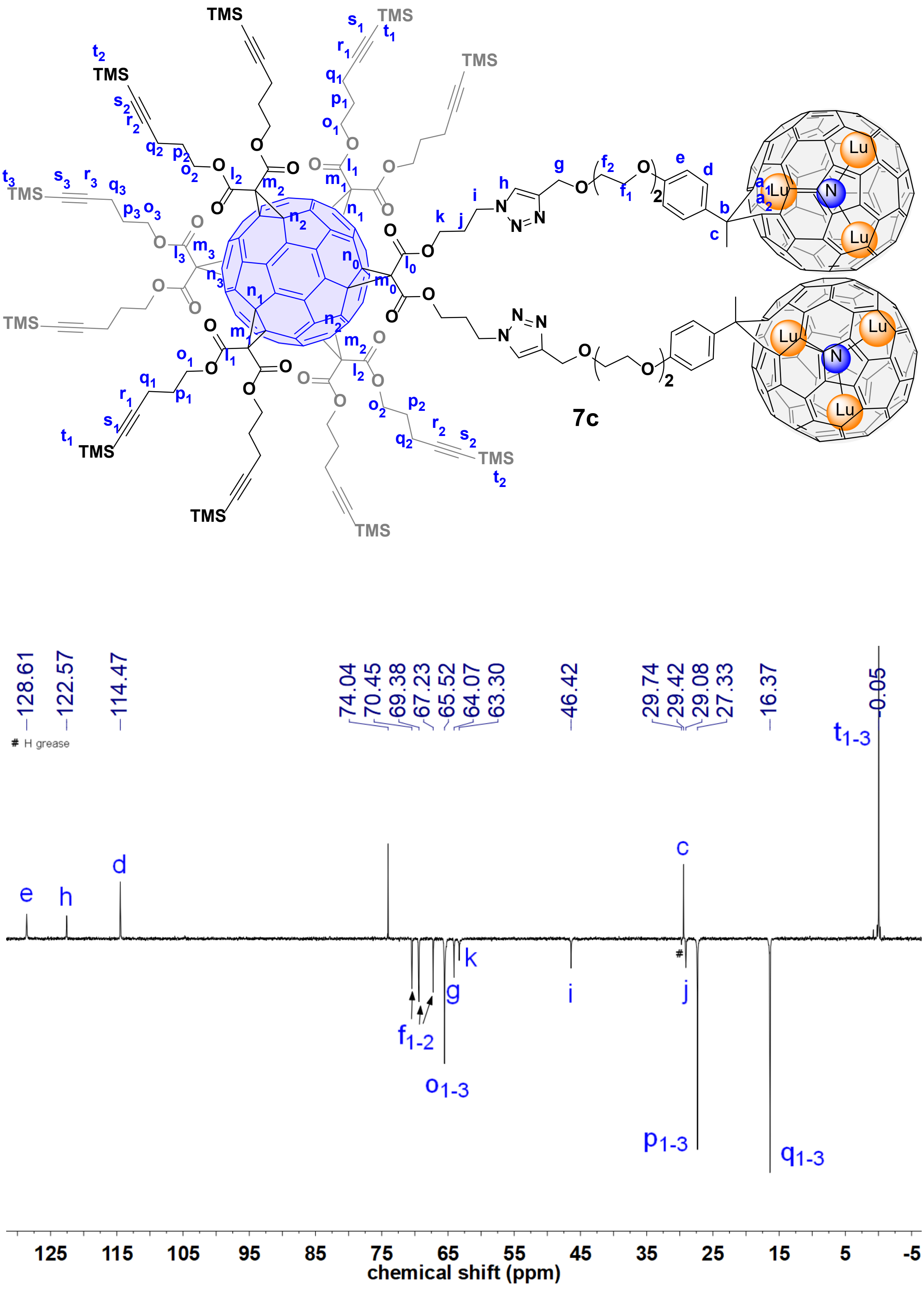

Figure S67. DEPT-135 spectrum of **7c**.

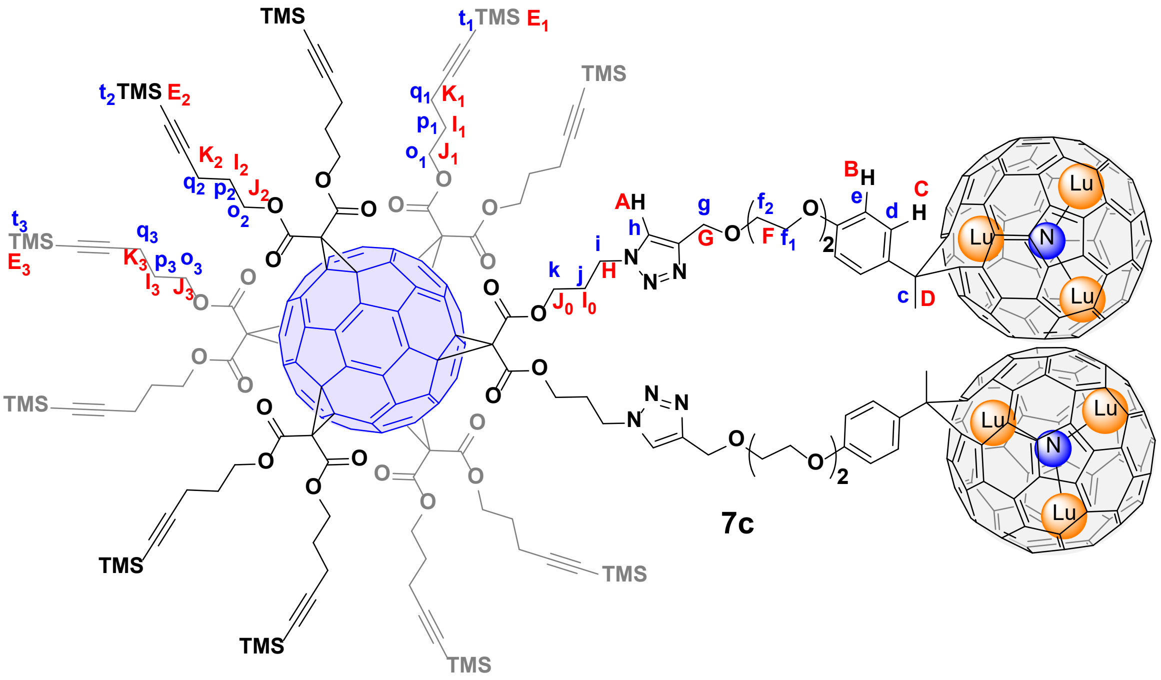

7c

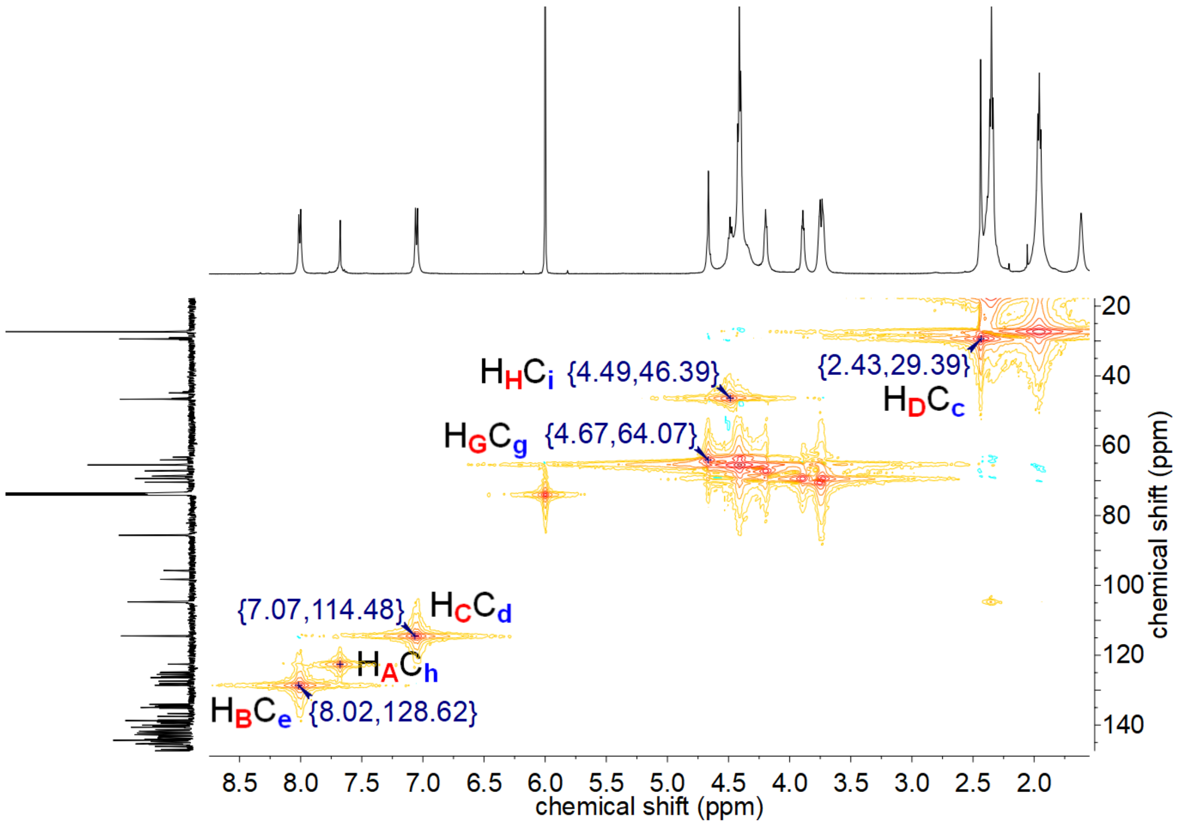

Figure S68. HMQC NMR spectrum of **7c**.

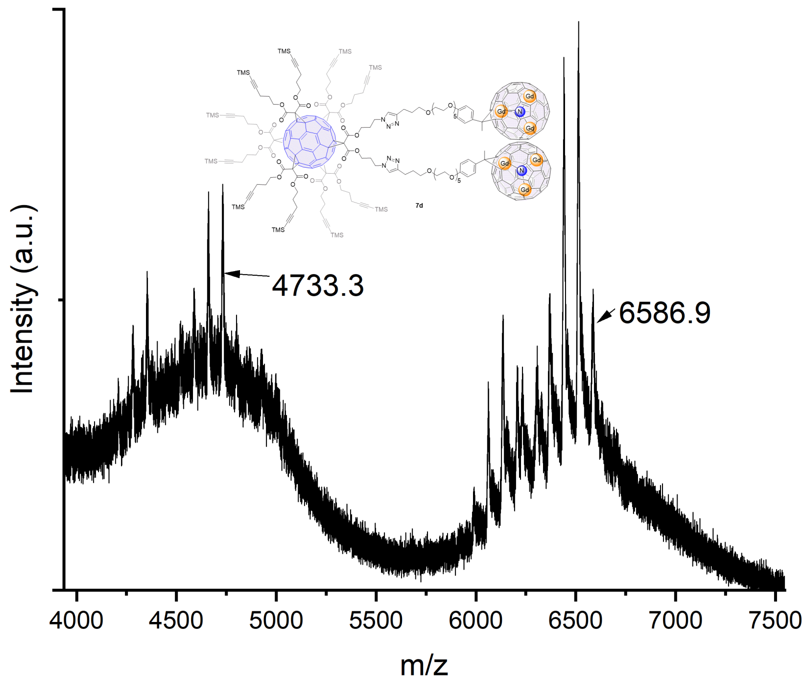

Figure S69. MALDI-TOF mass spectrometry of **7d** (average m/z calculated 6587.0, found 6586.9. The major fragment at 4733.3 is due to the loss of one metallofullerene derivative)

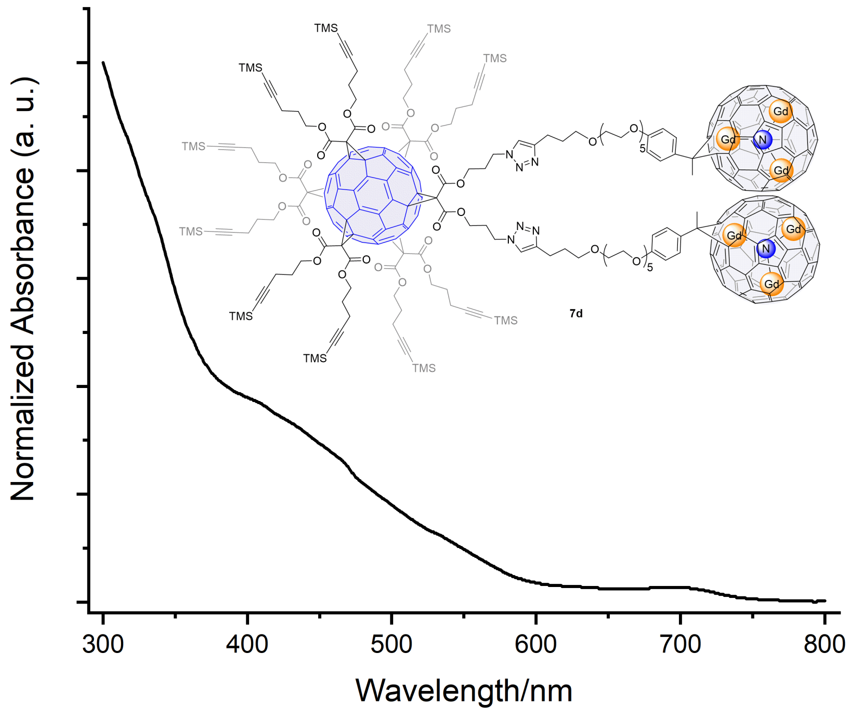

Figure S70. UV-vis spectrum of **7d** in $CHCl_3$.

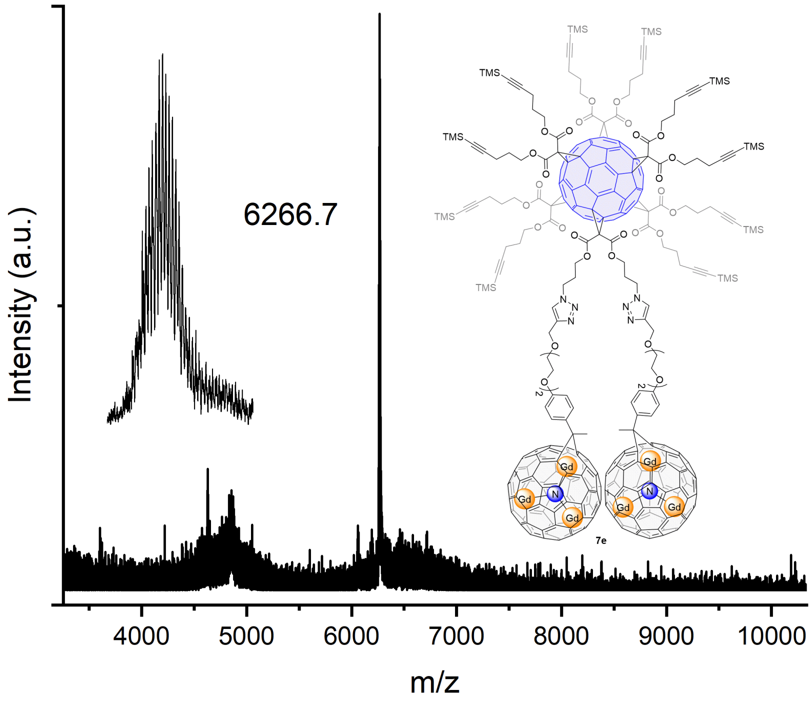

Figure S71. MALDI-TOF mass spectrometry of **7e** (average m/z calculated 6266.7, found 6266.7)

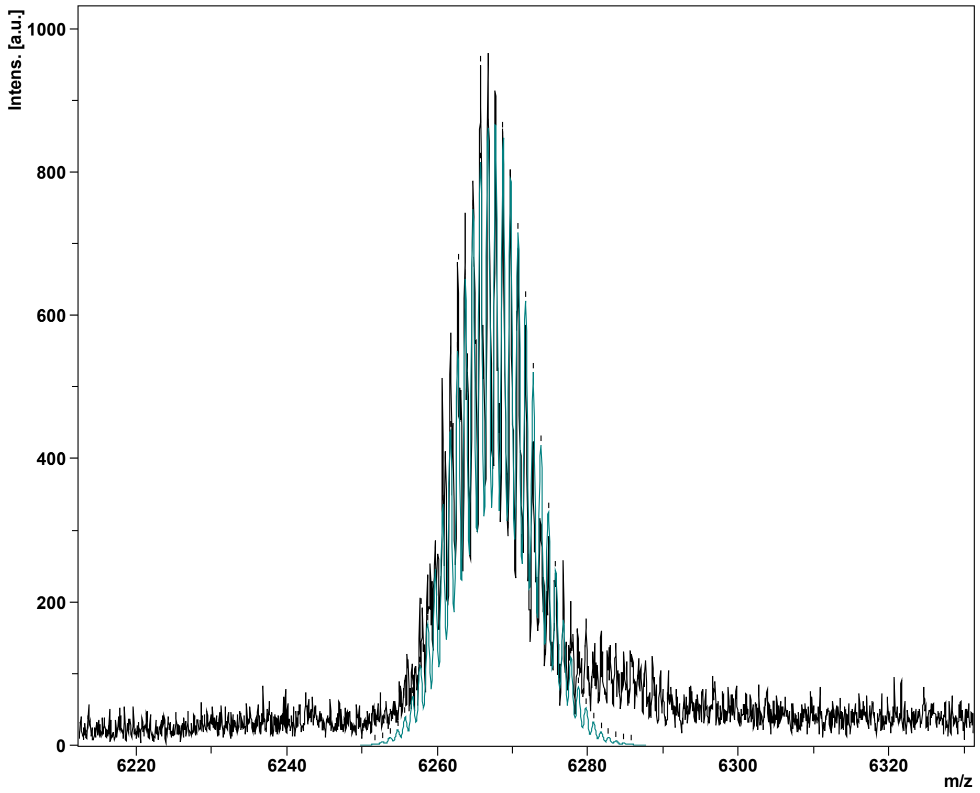

Figure S72. Calculated (green) vs experimental (black) MALDI-TOF MS of of **7e**.

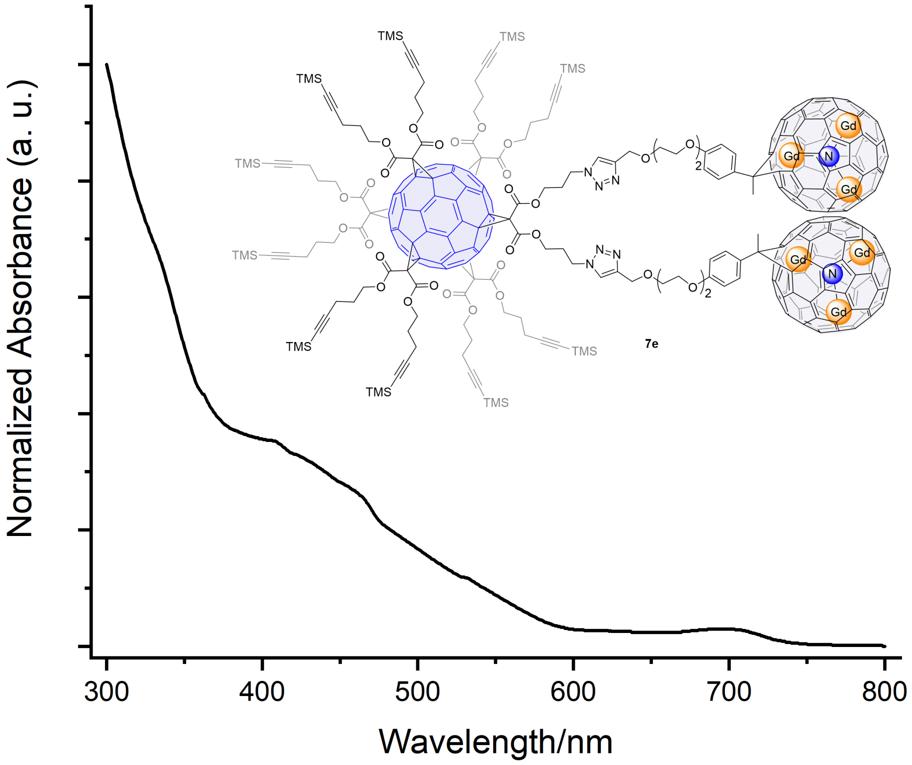

Figure S73. UV-vis spectrum of **7e** in $CHCl_3$.

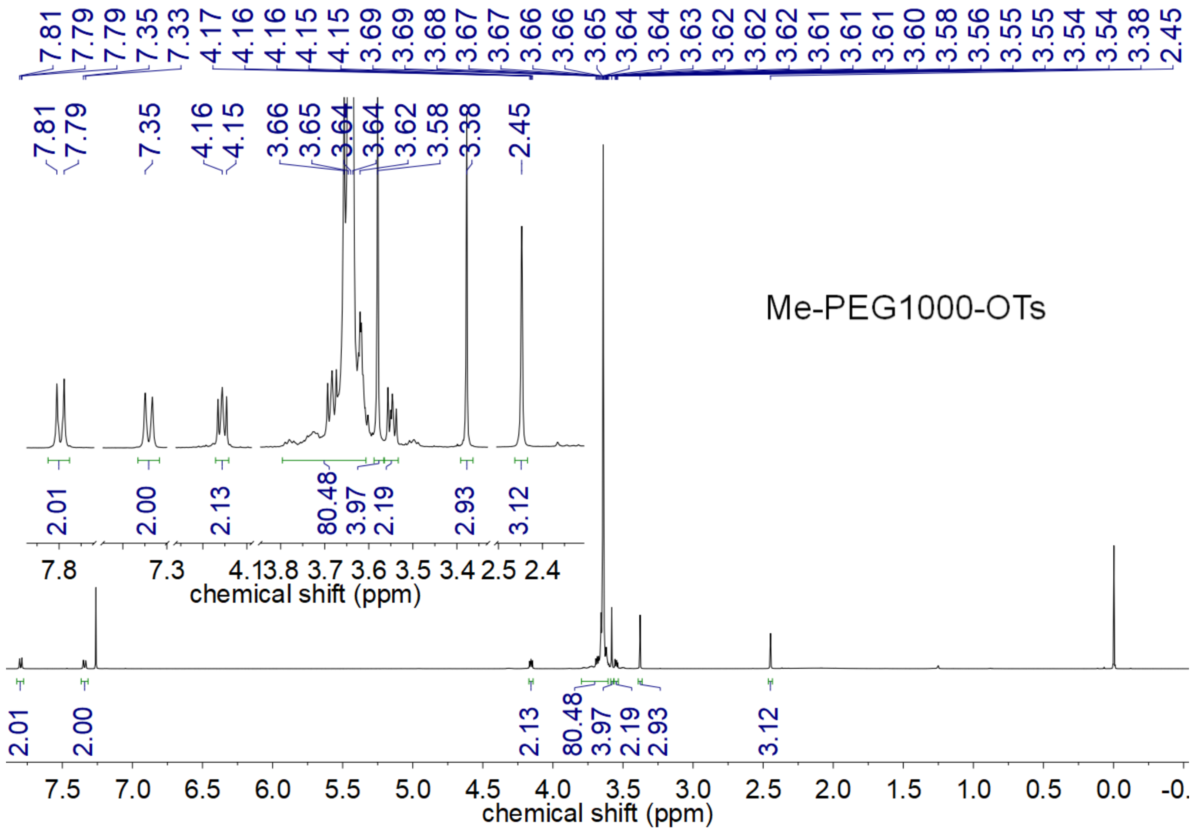

Figure S74. ^{1}H NMR spectrum of **Me-PEG1000-OTs**.

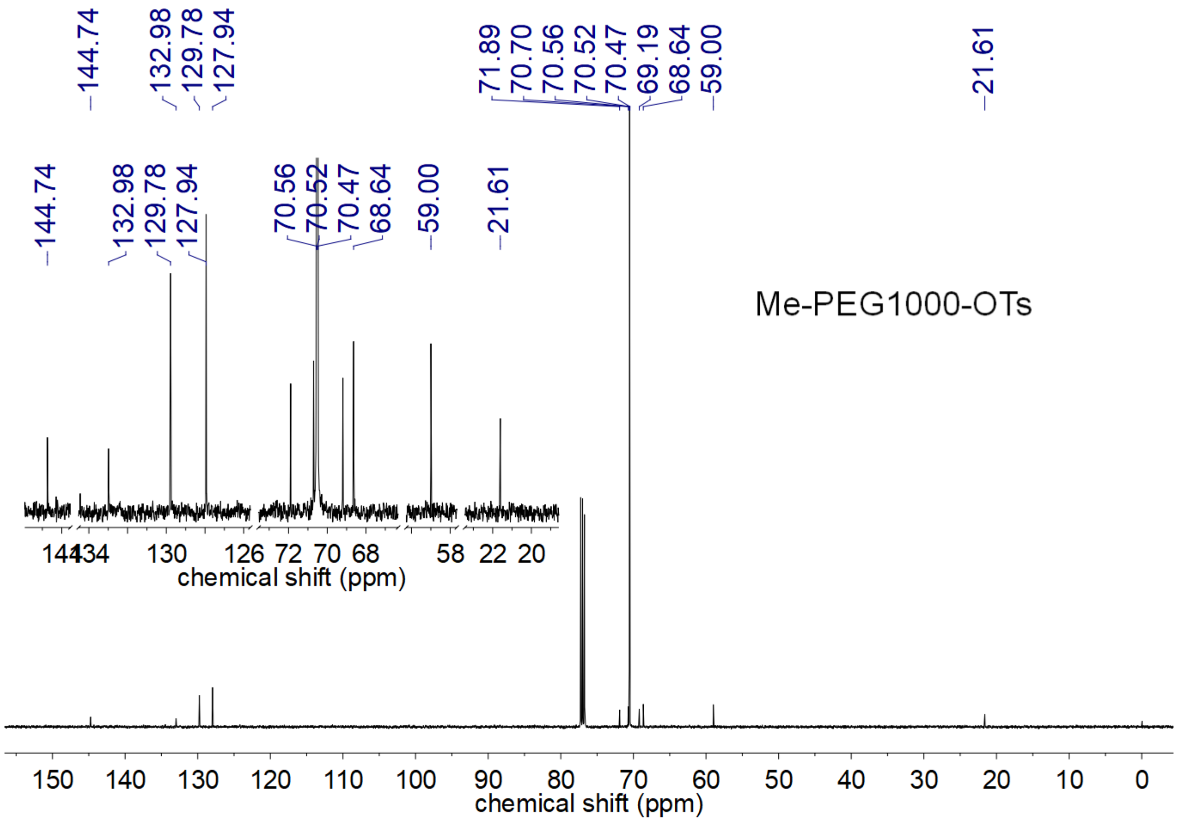

Figure S75. ^{13}C NMR spectrum of **Me-PEG1000-OTs**.

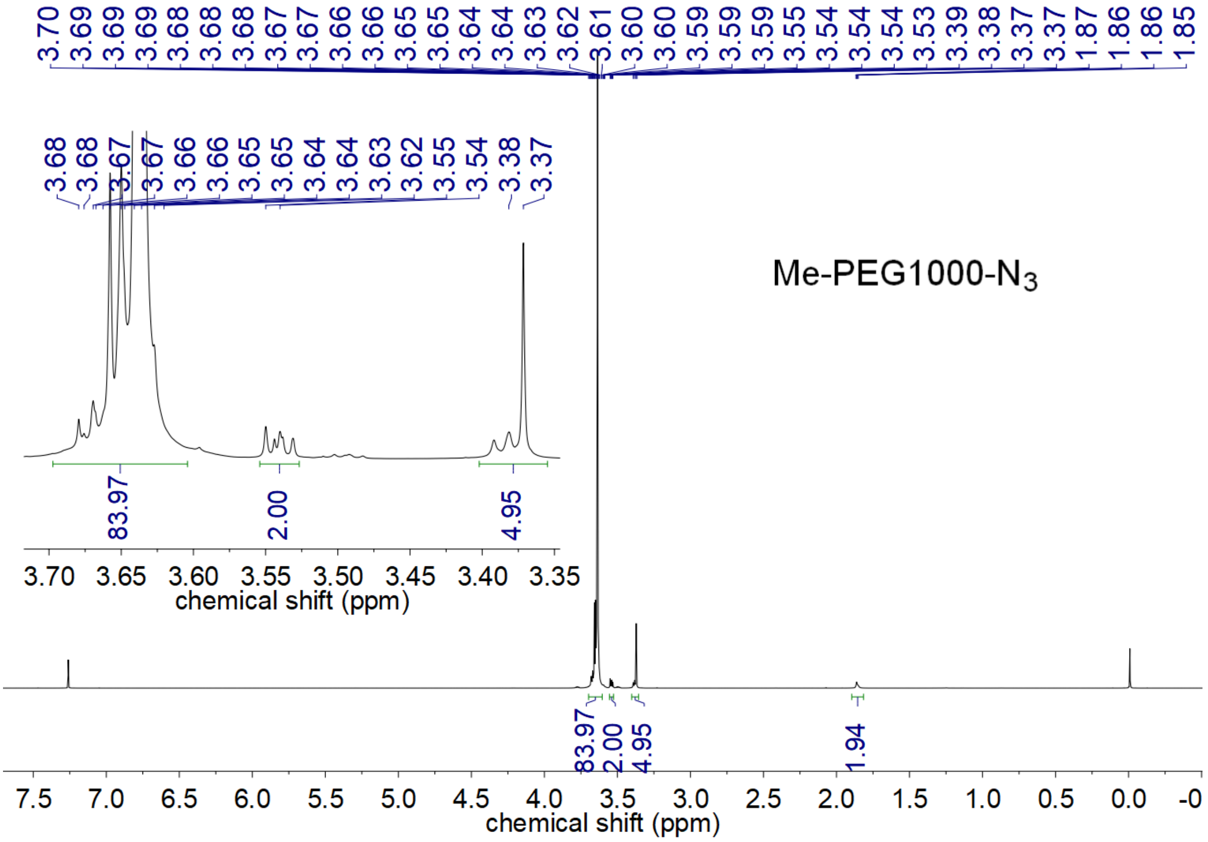

Figure S76. ^{1}H NMR spectrum of **Me-PEG1000-N$_3$**.

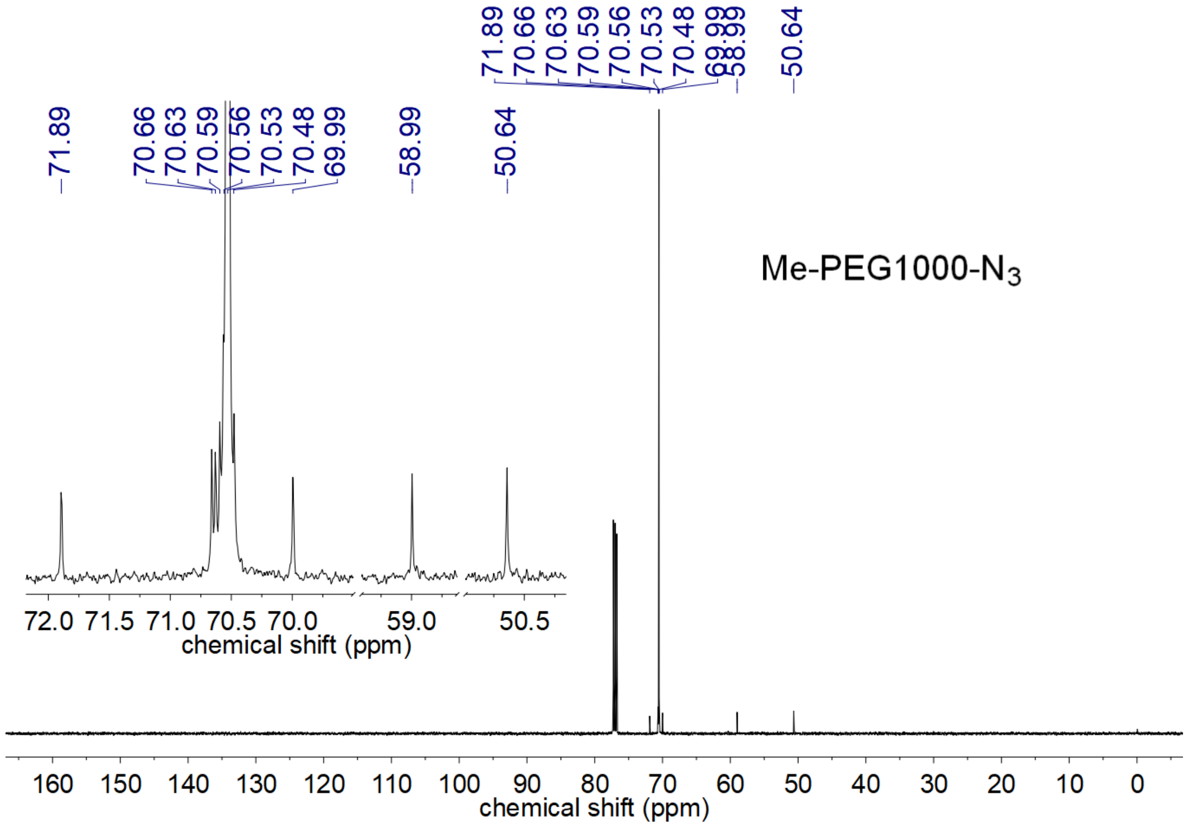

Figure S77. ^{13}C NMR spectrum of **Me-PEG1000-N$_3$**.

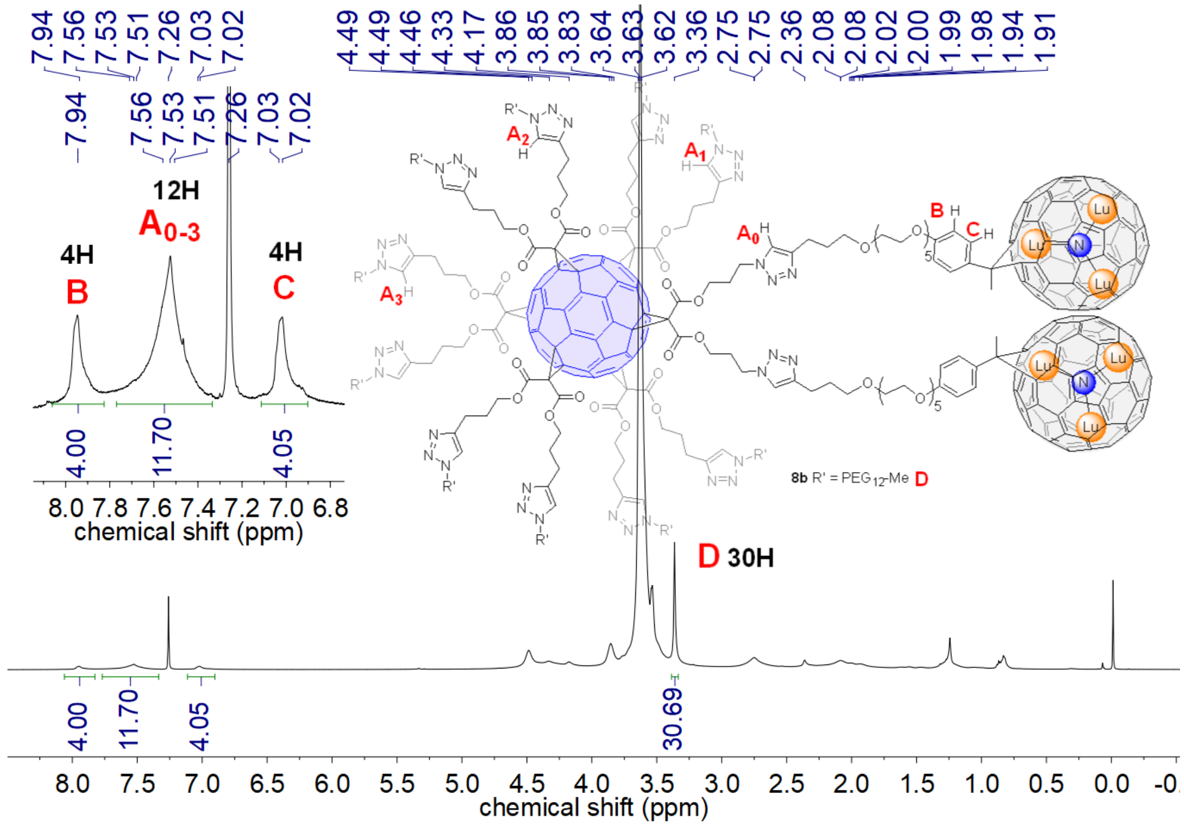

Figure S78. ^{1}H NMR spectrum of **8b**.

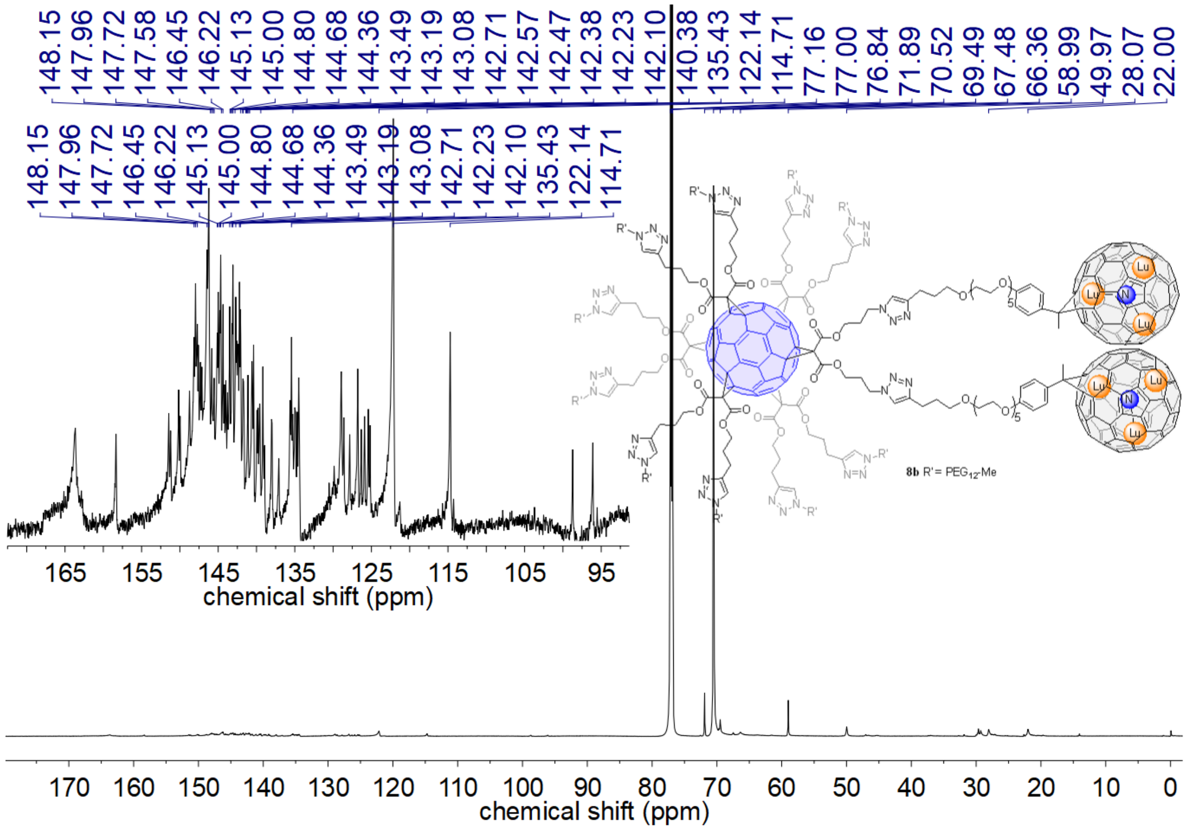

Figure S79. ^{13}C NMR spectrum of **8b**.

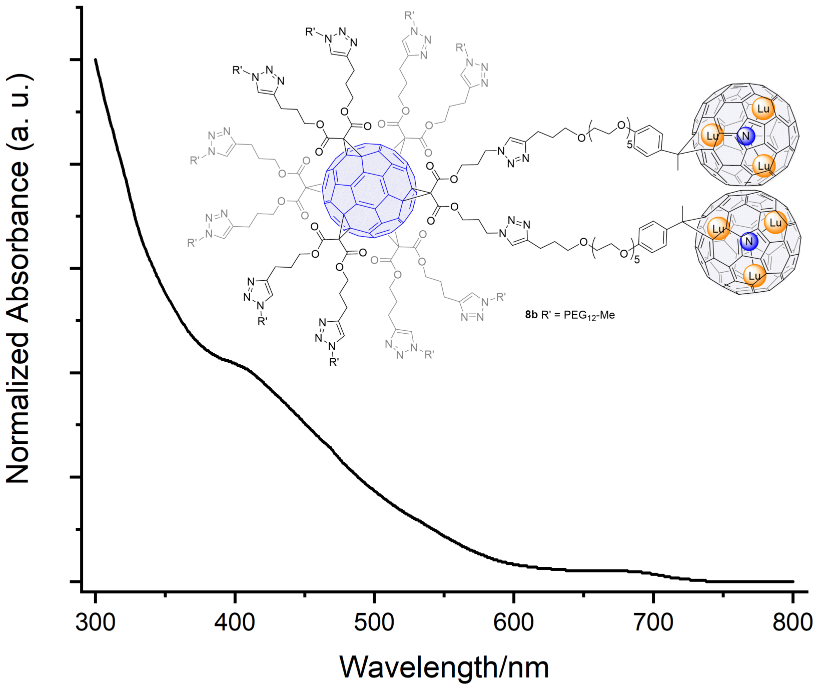

Figure S80. UV-vis spectrum of **8b** in $CHCl_3$.

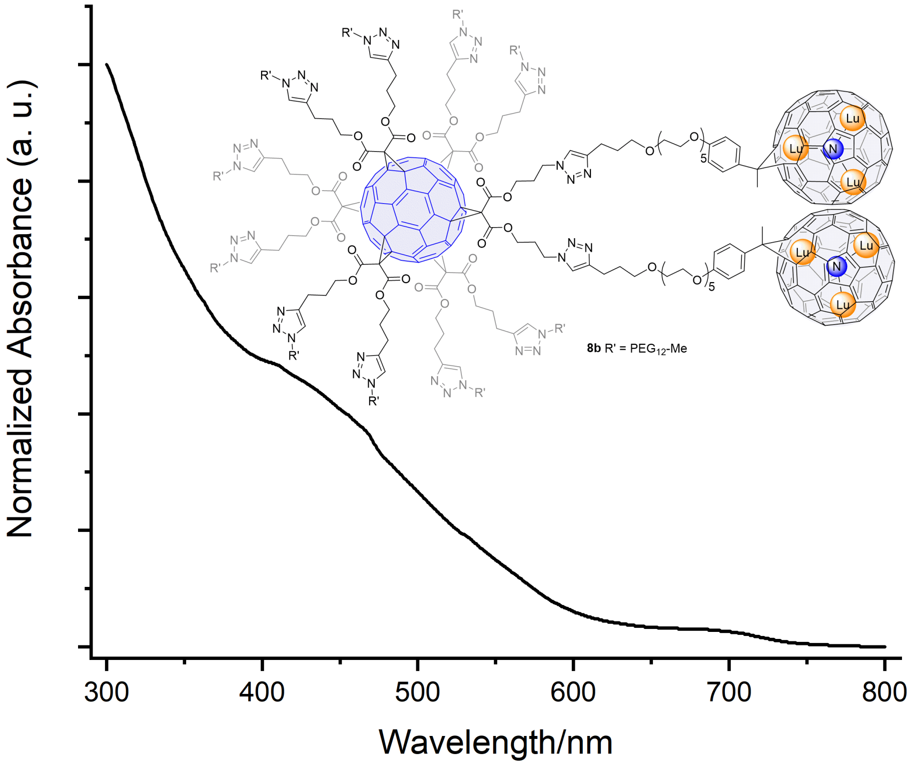

Figure S81. UV-vis spectrum of **8b** in water.

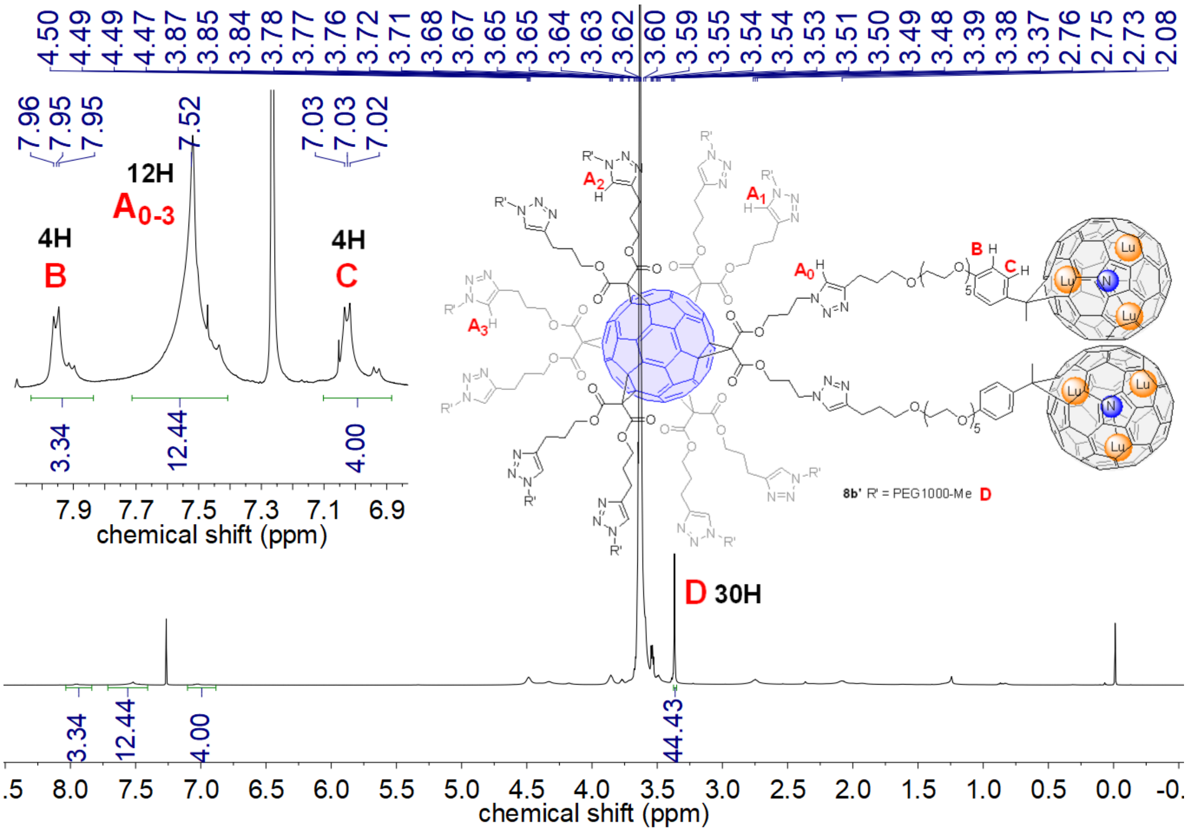

Figure S82. ^{1}H NMR spectrum of **8b′**.

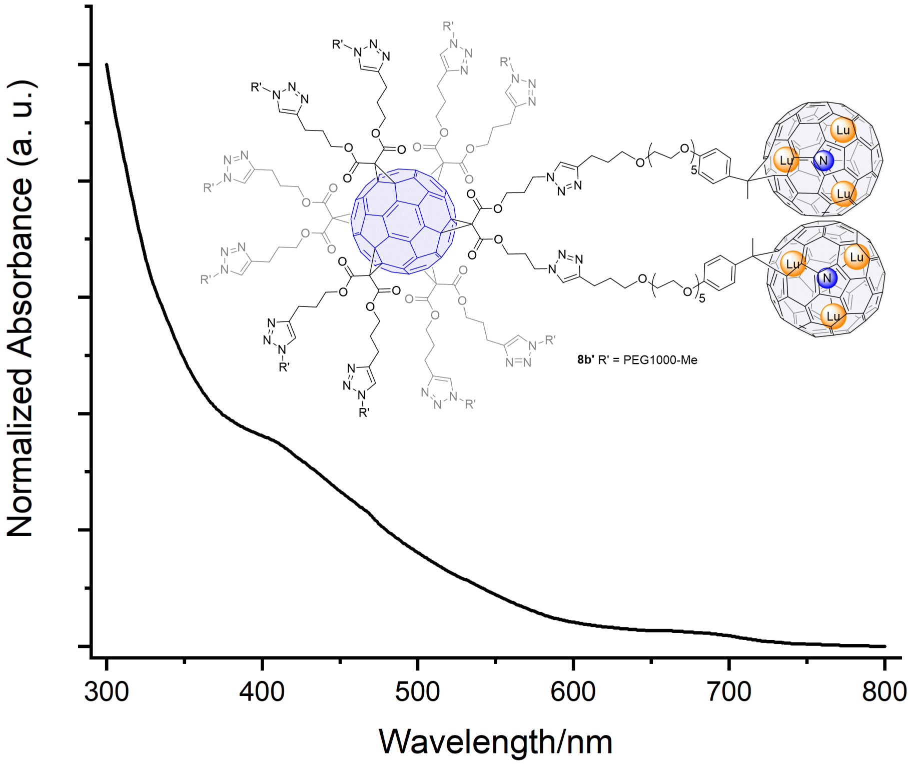

Figure S83. UV-vis spectrum of **8b′** in $CHCl_3$.

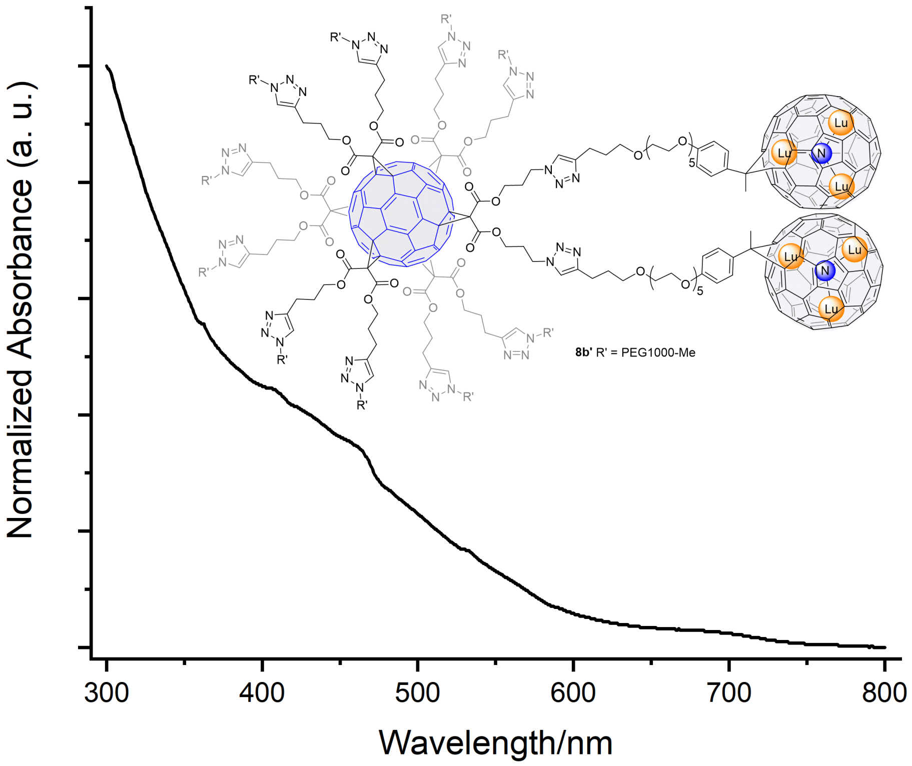

Figure S84. UV-vis spectrum of **8b′** in water.

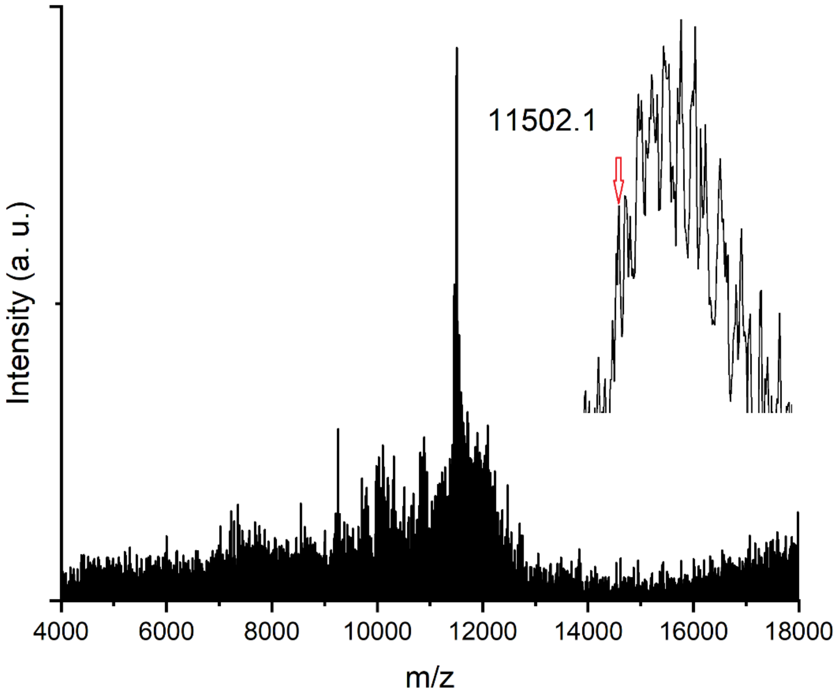

Figure S85. MALDI-TOF mass spectrometry of **8c** (molecular ion peak, m/z calculated 11501.9, found 11502.1)

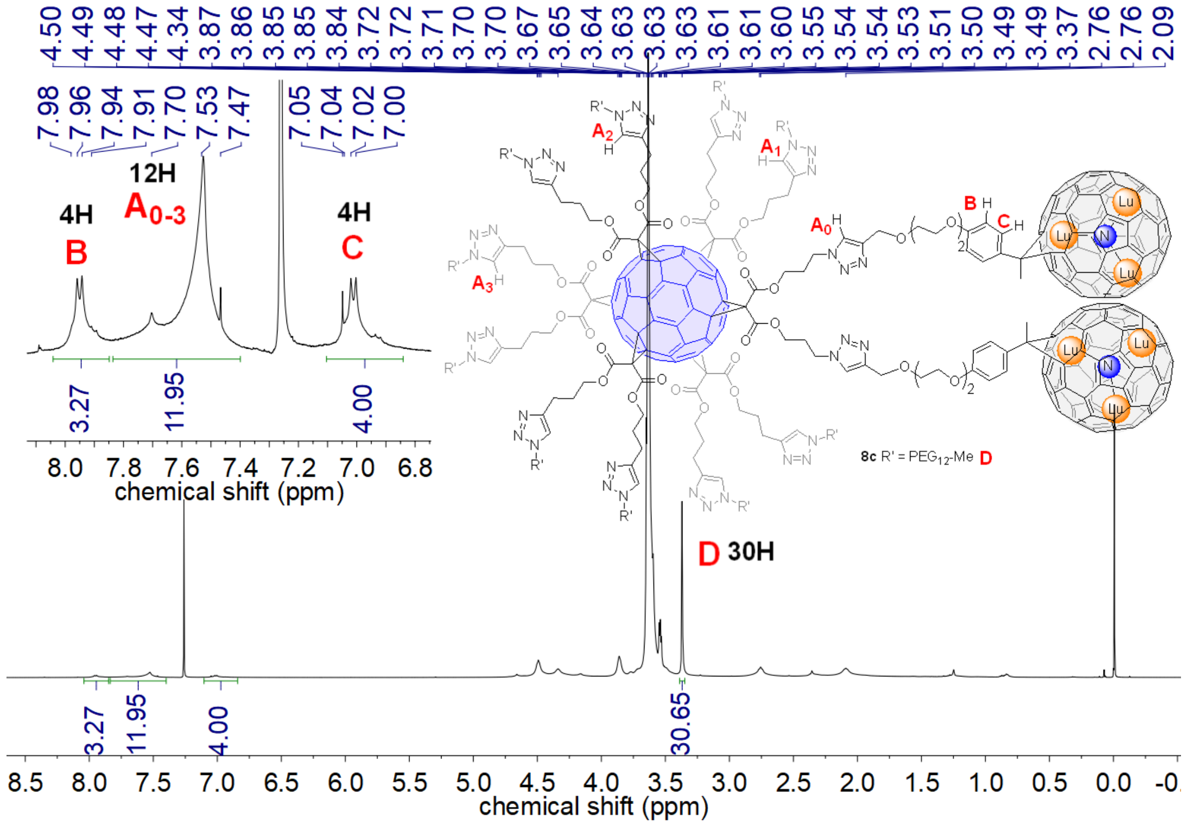

Figure S86. ^{1}H NMR spectrum of **8c**.

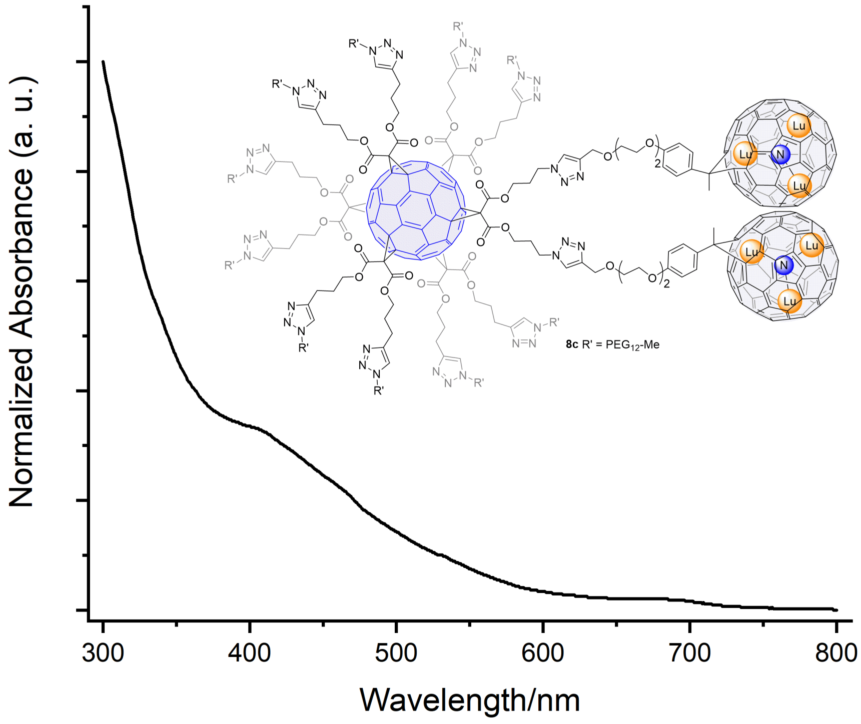

Figure S87. UV-vis spectrum of **8c** in $CHCl_3$.

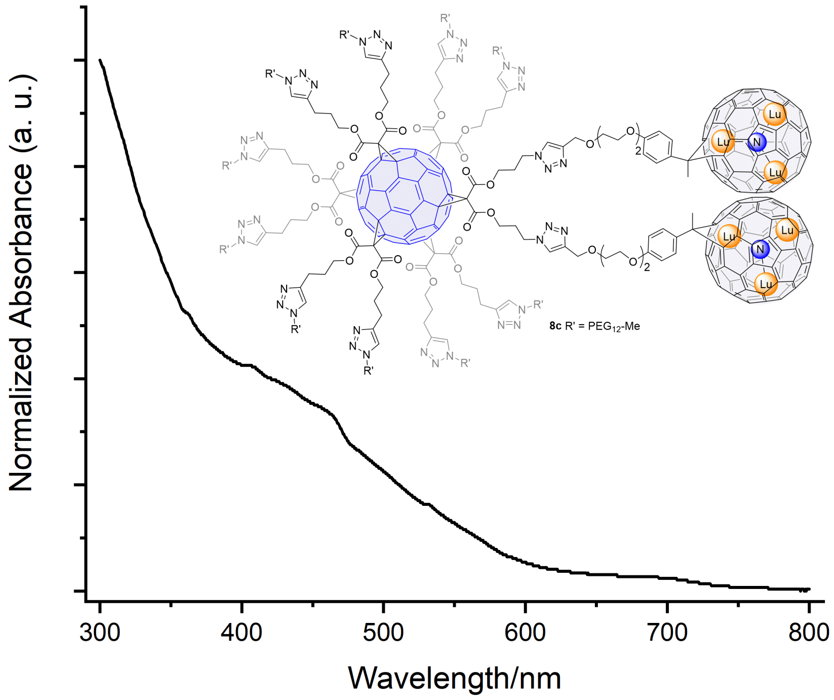

Figure S88. UV-vis spectrum of **8c** in water.

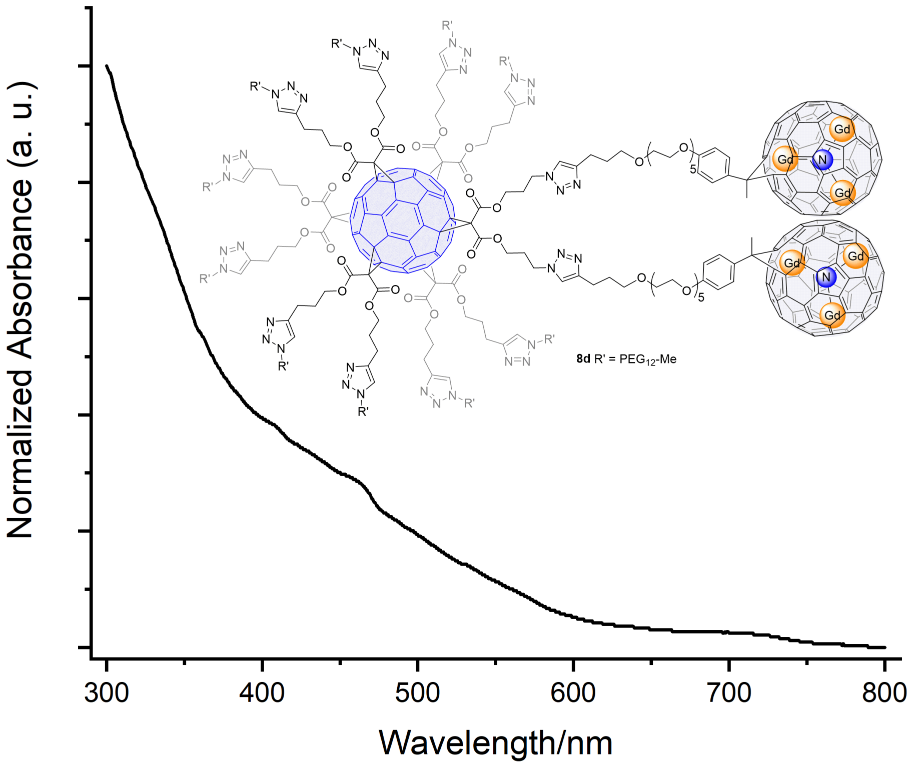

Figure S89. UV-vis spectrum of **8d** in water.

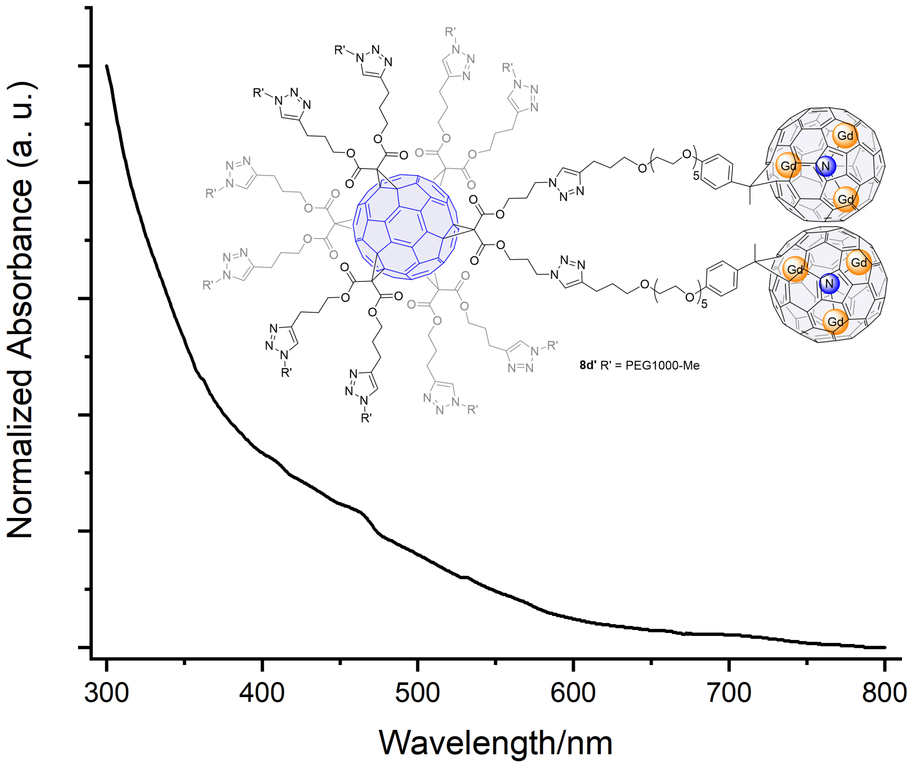

Figure S90. UV-vis spectrum of **8d'** in water.

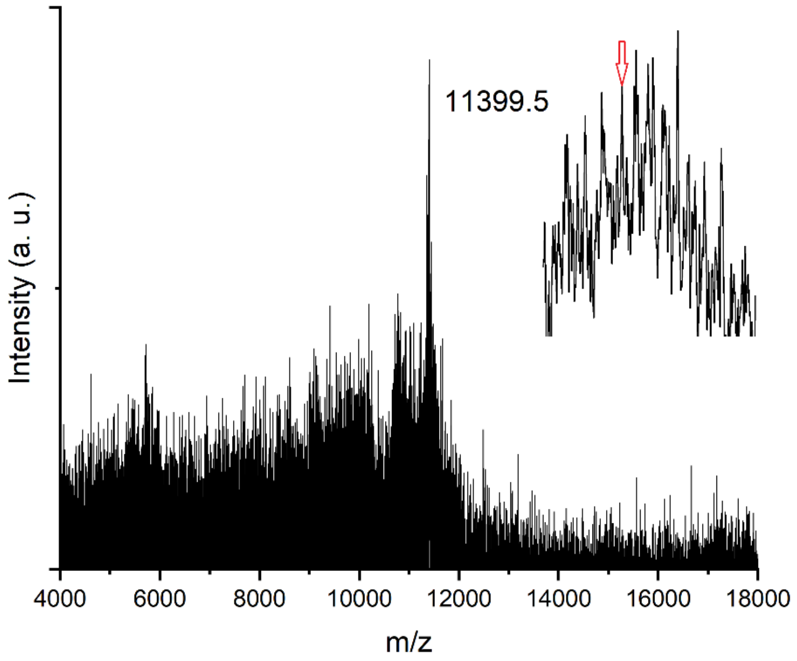

Figure S91. MALDI-TOF mass spectrometry of **8e** (molecular ion peak, m/z calculated 11399.8, found 11399.5)

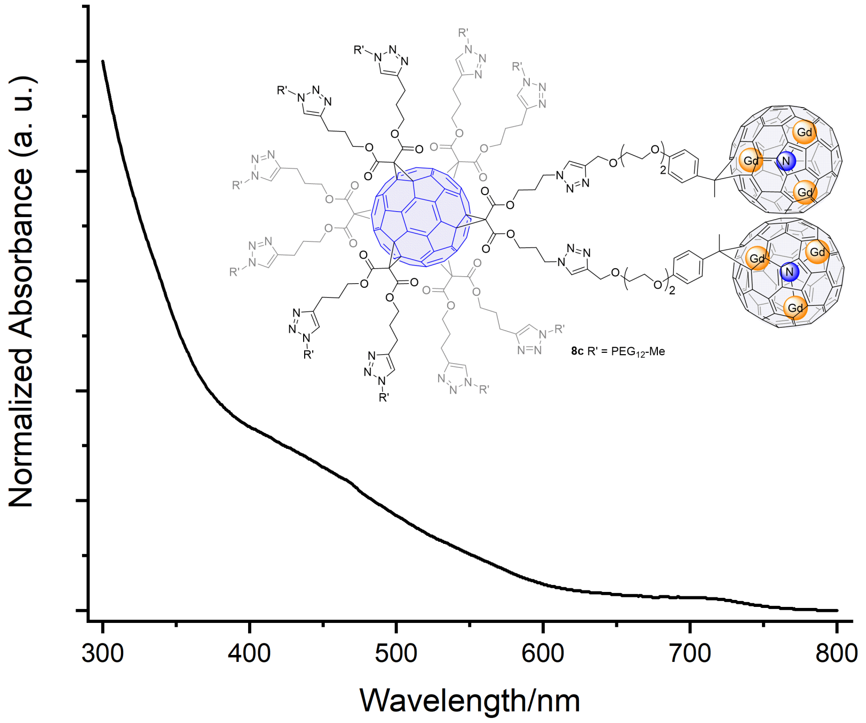

Figure S92. UV-vis spectrum of **8e** in water.

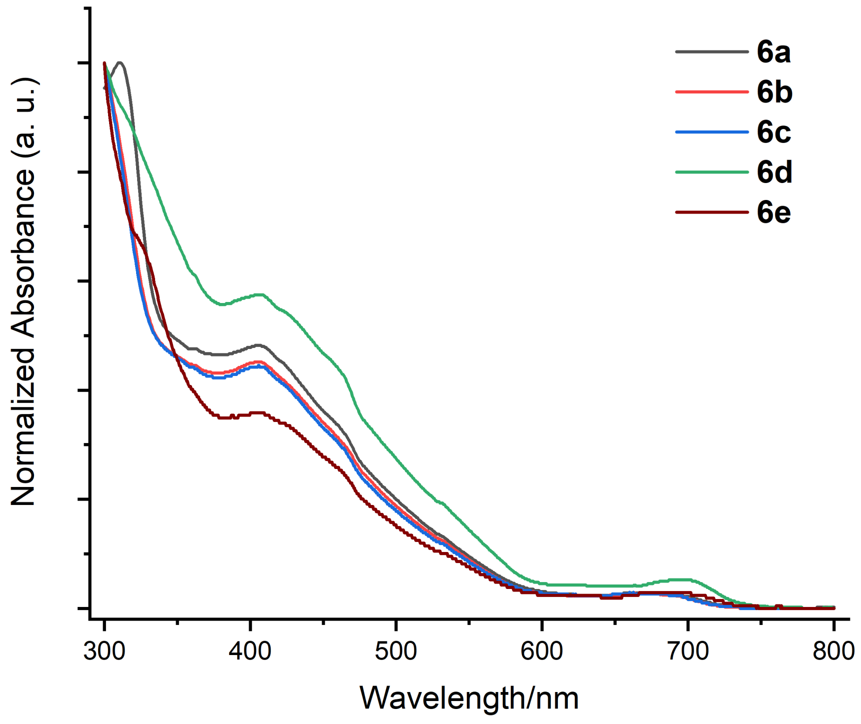

Figure S93. Overlapped UV-vis spectra of **6a-e** in $CHCl_3$.

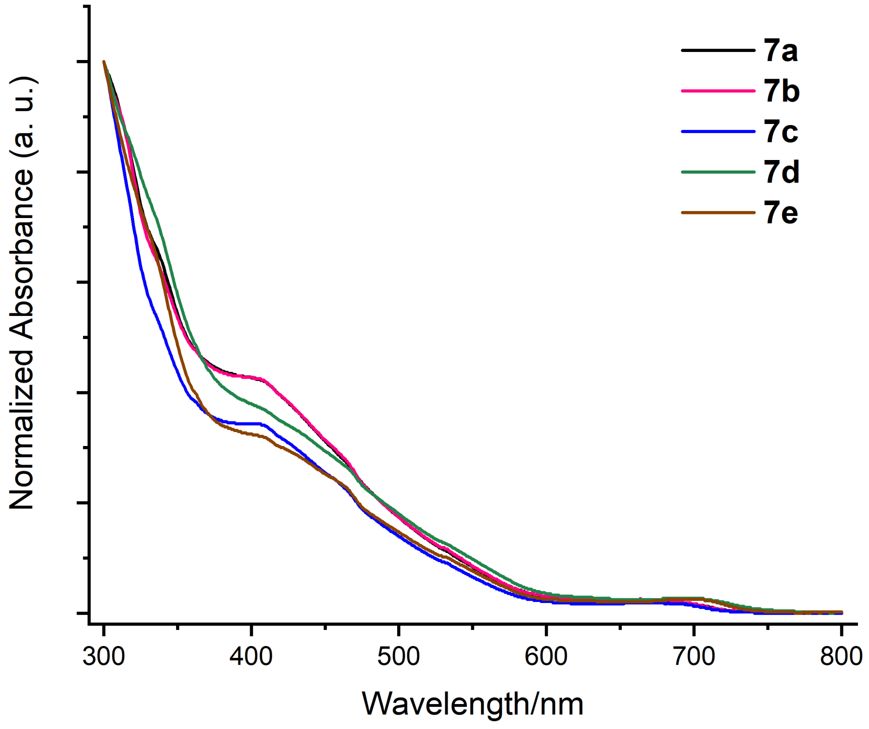

Figure S94. Overlapped UV-vis spectra of **7a-e** in $CHCl_3$.

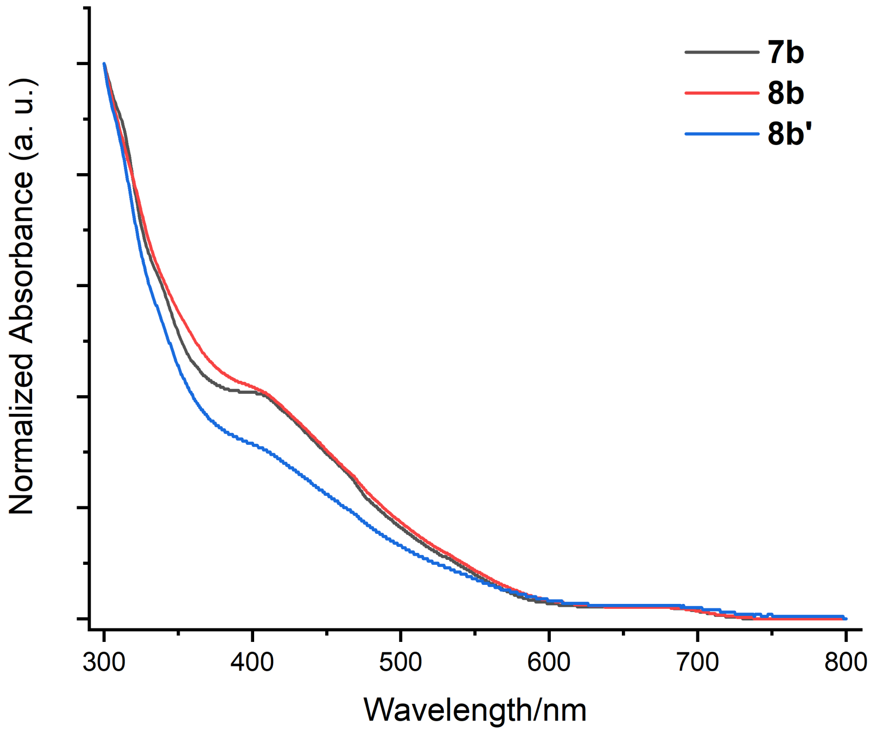

Figure S95. Overlapped UV-vis spectra of **7b**, **8b** and **8b′** in $CHCl_3$.

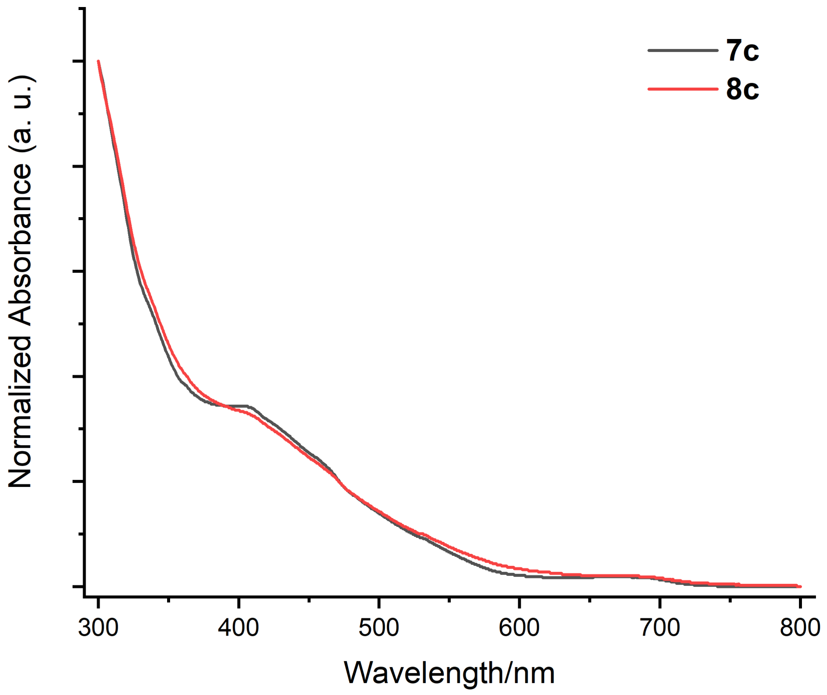

Figure S96. Overlapped UV-vis spectra of **7c** and **8c** in $CHCl_3$.

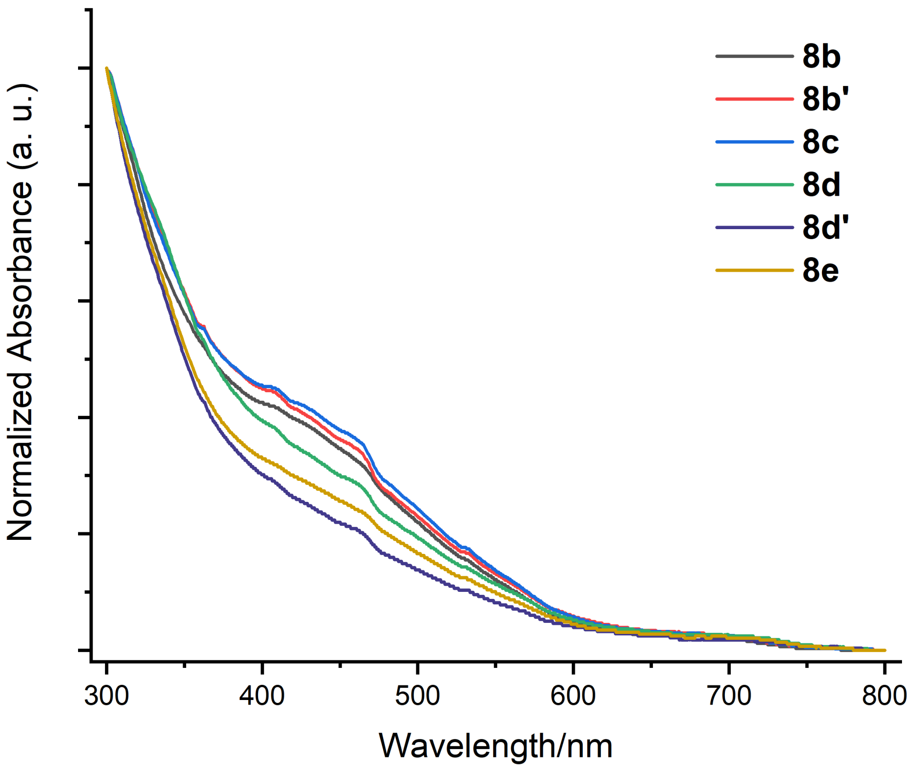

Figure S97. Overalpped UV-vis spectra of **8b**, **8b′**, **8c**, **8d**, **8d′** and **8e** in water.

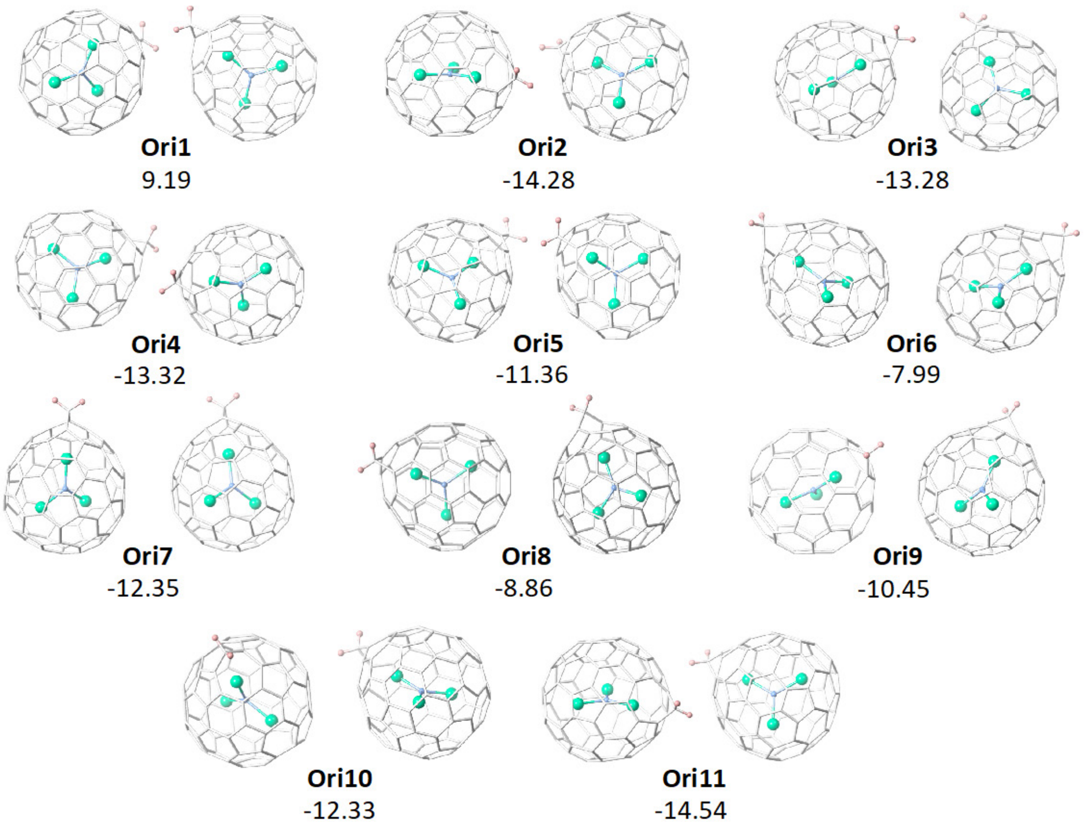

Figure S98. DFT optimized structures of the different orientations (Ori) of $(Lu_3N@C_{80}\text{-}CH_2)_2$ with their corresponding interaction energies (in kcal·mol^{-1}) indicated below of each geometry.

Table S2. Interaction energy (in kcal·mol^{-1}) of models of $(Lu_3N@C_{80}\text{-}CH_2)_2$ varying the shortest C···C distance. N···N distances are also indicated. Distances are in Å.

C···C dist.	E_{int}	N···N dist.
2.5	5.49	10.2
3.26	-12.35	11.0
4.0	-9.49	11.7
5.0	-5.84	12.7
6.0	-4.28	13.7

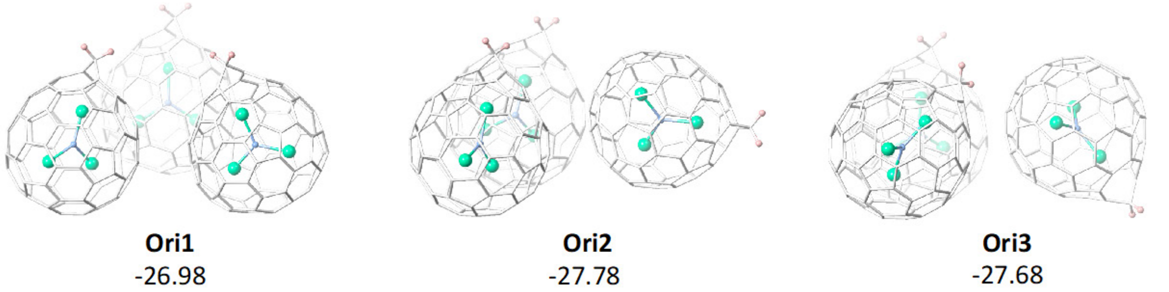

Figure S99. DFT optimized structures of the different orientations (Ori) of $(Lu_3N@C_{80}\text{-}CH_2)_3$ with their corresponding interaction energies (in kcal·mol^{-1}) indicated below of each geometry.

Table S3. Interaction energy (in kcal·mol^{-1}) of models of $(Lu_3N@C_{80}\text{-}CH_2)_3$ varying the shortest C···C distance (in Å).

C···C dist.	E_{int}
Varying the distance of one $Lu_3N@C_{80}\text{-}CH_2$	
3.35	-26.98
4.0	-24.06
5.0	-18.16
6.0	-14.51
10.0	-10.75
Varying the distance of all $Lu_3N@C_{80}\text{-}CH_2$	
5x5x5	-10.29

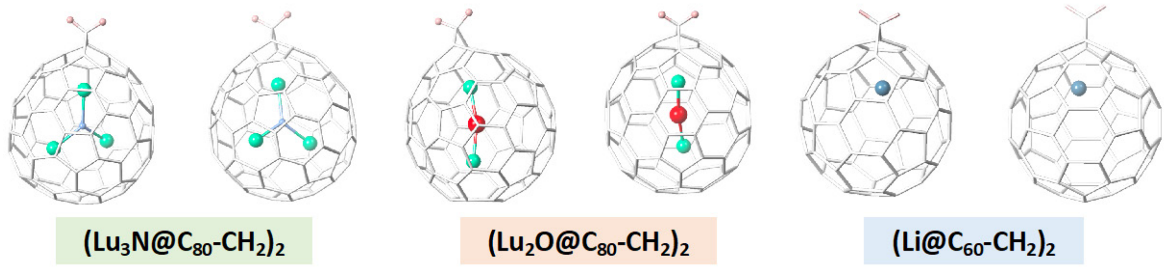

Figure S100. DFT optimized structures of $(Lu_3N@C_{80}\text{-}CH_2)_2$, $(Lu_2O@C_{80}\text{-}CH_2)_2$ and $(Li@C_{60}\text{-}CH_2)_2$.

Table S4. Interaction energy (in kcal·mol^{-1}), shortest C···C distance (in Å) and X···X distance (X=N, O or Li) distance (in Å) for different models of dimers.

Dimer	E_{int}	C···C distance	X···X distance
$(Lu_3N@C_{80}\text{-}CH_2)_2$	-12.35	3.26	11.00
$(Lu_2O@C_{80}\text{-}CH_2)_2$	-10.79	2.92	10.96
$(Li@C_{60}\text{-}CH_2)_2$	-7.83	3.17	9.33
$(Lu_3N@C_{80})_2$	-9.32	3.20	11.09
$(C_{80}\text{-}CH_2)_2$	-9.27	3.05	
$(Li@C_{60})_2$	-8.83	3.06	9.44
$(C_{60}\text{-}CH_2)_2$	-7.10	3.31	

Table S5. Interaction energy (in kcal·mol^{-1}) of $(Lu_3N@C_{80}\text{-}CH_2)_2$ with and without dispersion Grimme D3.

**$(Lu_3N@C_{80}\text{-}CH_2)_2$**	**E_{int}**
With Dispersion	-12.35
Without Dispersion (SP)*	-3.33
Without Dispersion (OPT)**	-4.58

*SP: Single point from the optimized structure with dispersion. **OPT: Optimization from the optimized structure with dispersion.

5. Supplemental references

1) Iehl, J.; Pereira de Freitas, R.; Delavaux-Nicot, B.; Nierengarten, J.-F. *Chem. Commun.* **2008**, 2450-2452.

2) Abellán Flos, M.; García Moreno, M. I.; Ortiz Mellet, C.; García Fernández, J. M.; Nierengarten, J. F.; Vincent, S. P. *Chem. -Eur. J.* **2016**, *22*, 11450-11460.

3) Iehl, J.; Nierengarten, J.-F. *Chem. Eur. J.* **2009**, *15*, 7306-7309.

4) Yu, X.; Zhang, W.-B.; Yue, K.; Li, X.; Liu, H.; Xin, Y.; Wang, C.-L.; Wesdemiotis, C.; Cheng, S. Z. D. *J. Am. Chem. Soc.* **2012**, *134*, 7780-7787.

5) Zhou, W.; Zhang, L.; Jiao, N. *Angew. Chem. Int. Ed.* **2009**, *48*, 7094-7097.

6) Lewis, J. E. M.; Modicom, F.; Goldup, S. M. *J. Am. Chem. Soc.* **2018**, *140*, 4787-4791.

7) Diot, J.; García-Moreno, M. I.; Gouin, S. G.; Ortiz Mellet, C.; Haupt, K.; Kovensky, J. *Org. Biomol. Chem.* **2009**, *7*, 357-363.

8) Tran, F.; Odell, A. V.; Ward, G. E.; Westwood, N. J. *Molecules* **2013**, *18*, 11639-11657.

9) Te Velde, G.; Bickelhaupt, F. M.; Baerends, E. J.; FonsecaGuerra, C.; van Gisbergen, S. J. A.; Snijders, J. G.; Ziegler, T. Chemistry with ADF. *J. Comput. Chem.* **2001**, *22*, 931- 967.

10) J. P. Perdew, K. Burke, M. Ernzerhof, *Phys. Rev. Lett.* 1996, **77**, 3865; J. P. Perdew, K. Burke, M. Ernzerhof, *Phys. Rev. Lett.* 1997, **78**, 1396 (E).

11) A. D. Becke, *J. Chem. Phys.*, 1986, **84**, 4524-4529.

12) J. P. Perdew, *Phys. Rev. B: Condens. Matter Mater. Phys.*, 1986, **33**, 8822-8824.

13) E. van Lenthe, E. J. Baerends and J. G. Snijders , *J. Chem. Phys.*, 1993, **99** , 4597-4610.

14) S. Grimme, J. Antony, S. Ehrlich, H. Krieg, *J. Chem. Phys.*, 2010, **132**, 154104.

15) S. Grimme, S. Ehrlich, L. Goerigk, *J. Comput. Chem.*, 2011, **32**, 1456-1465.
